# Inferring structural variant cancer cell fraction

**DOI:** 10.1038/s41467-020-14351-8

**Published:** 2020-02-05

**Authors:** Marek Cmero, Ke Yuan, Cheng Soon Ong, Jan Schröder, PCAWG Evolution and Heterogeneity Working Group, David J. Adams, Pavana Anur, Rameen Beroukhim, Paul C. Boutros, David D. L. Bowtell, Peter J. Campbell, Shaolong Cao, Elizabeth L. Christie, Yupeng Cun, Kevin J. Dawson, Jonas Demeulemeester, Stefan C. Dentro, Amit G. Deshwar, Nilgun Donmez, Ruben M. Drews, Roland Eils, Yu Fan, Matthew W. Fittall, Dale W. Garsed, Moritz Gerstung, Gad Getz, Santiago Gonzalez, Gavin Ha, Kerstin Haase, Marcin Imielinski, Lara Jerman, Yuan Ji, Clemency Jolly, Kortine Kleinheinz, Juhee Lee, Henry Lee-Six, Ignaty Leshchiner, Dimitri Livitz, Salem Malikic, Iñigo Martincorena, Thomas J. Mitchell, Quaid D. Morris, Ville Mustonen, Layla Oesper, Martin Peifer, Myron Peto, Benjamin J. Raphael, Daniel Rosebrock, Yulia Rubanova, S. Cenk Sahinalp, Adriana Salcedo, Matthias Schlesner, Steven E. Schumacher, Subhajit Sengupta, Ruian Shi, Seung Jun Shin, Paul T. Spellman, Oliver Spiro, Lincoln D. Stein, Maxime Tarabichi, Peter Van Loo, Shankar Vembu, Ignacio Vázquez-García, Wenyi Wang, David C. Wedge, David A. Wheeler, Jeffrey A. Wintersinger, Tsun-Po Yang, Xiaotong Yao, Kaixian Yu, Hongtu Zhu, Niall M. Corcoran, Tony Papenfuss, Christopher M. Hovens, Florian Markowetz, Geoff Macintyre, PCAWG Consortium, Lauri A. Aaltonen, Federico Abascal, Adam Abeshouse, Hiroyuki Aburatani, David J. Adams, Nishant Agrawal, Keun Soo Ahn, Sung-Min Ahn, Hiroshi Aikata, Rehan Akbani, Kadir C. Akdemir, Hikmat Al-Ahmadie, Sultan T. Al-Sedairy, Fatima Al-Shahrour, Malik Alawi, Monique Albert, Kenneth Aldape, Ludmil B. Alexandrov, Adrian Ally, Kathryn Alsop, Eva G. Alvarez, Fernanda Amary, Samirkumar B. Amin, Brice Aminou, Ole Ammerpohl, Matthew J. Anderson, Yeng Ang, Davide Antonello, Pavana Anur, Samuel Aparicio, Elizabeth L. Appelbaum, Yasuhito Arai, Axel Aretz, Koji Arihiro, Shun-ichi Ariizumi, Joshua Armenia, Laurent Arnould, Sylvia Asa, Yassen Assenov, Gurnit Atwal, Sietse Aukema, J. Todd Auman, Miriam R. R. Aure, Philip Awadalla, Marta Aymerich, Gary D. Bader, Adrian Baez-Ortega, Matthew H. Bailey, Peter J. Bailey, Miruna Balasundaram, Saianand Balu, Pratiti Bandopadhayay, Rosamonde E. Banks, Stefano Barbi, Andrew P. Barbour, Jonathan Barenboim, Jill Barnholtz-Sloan, Hugh Barr, Elisabet Barrera, John Bartlett, Javier Bartolome, Claudio Bassi, Oliver F. Bathe, Daniel Baumhoer, Prashant Bavi, Stephen B. Baylin, Wojciech Bazant, Duncan Beardsmore, Timothy A. Beck, Sam Behjati, Andreas Behren, Beifang Niu, Cindy Bell, Sergi Beltran, Christopher Benz, Andrew Berchuck, Anke K. Bergmann, Erik N. Bergstrom, Benjamin P. Berman, Daniel M. Berney, Stephan H. Bernhart, Rameen Beroukhim, Mario Berrios, Samantha Bersani, Johanna Bertl, Miguel Betancourt, Vinayak Bhandari, Shriram G. Bhosle, Andrew V. Biankin, Matthias Bieg, Darell Bigner, Hans Binder, Ewan Birney, Michael Birrer, Nidhan K. Biswas, Bodil Bjerkehagen, Tom Bodenheimer, Lori Boice, Giada Bonizzato, Johann S. De Bono, Arnoud Boot, Moiz S. Bootwalla, Ake Borg, Arndt Borkhardt, Keith A. Boroevich, Ivan Borozan, Christoph Borst, Marcus Bosenberg, Mattia Bosio, Jacqueline Boultwood, Guillaume Bourque, Paul C. Boutros, G. Steven Bova, David T. Bowen, Reanne Bowlby, David D. L. Bowtell, Sandrine Boyault, Rich Boyce, Jeffrey Boyd, Alvis Brazma, Paul Brennan, Daniel S. Brewer, Arie B. Brinkman, Robert G. Bristow, Russell R. Broaddus, Jane E. Brock, Malcolm Brock, Annegien Broeks, Angela N. Brooks, Denise Brooks, Benedikt Brors, Søren Brunak, Timothy J. C. Bruxner, Alicia L. Bruzos, Alex Buchanan, Ivo Buchhalter, Christiane Buchholz, Susan Bullman, Hazel Burke, Birgit Burkhardt, Kathleen H. Burns, John Busanovich, Carlos D. Bustamante, Adam P. Butler, Atul J. Butte, Niall J. Byrne, Anne-Lise Børresen-Dale, Samantha J. Caesar-Johnson, Andy Cafferkey, Declan Cahill, Claudia Calabrese, Carlos Caldas, Fabien Calvo, Niedzica Camacho, Peter J. Campbell, Elias Campo, Cinzia Cantù, Shaolong Cao, Thomas E. Carey, Joana Carlevaro-Fita, Rebecca Carlsen, Ivana Cataldo, Mario Cazzola, Jonathan Cebon, Robert Cerfolio, Dianne E. Chadwick, Dimple Chakravarty, Don Chalmers, Calvin Wing Yiu Chan, Kin Chan, Michelle Chan-Seng-Yue, Vishal S. Chandan, David K. Chang, Stephen J. Chanock, Lorraine A. Chantrill, Aurélien Chateigner, Nilanjan Chatterjee, Kazuaki Chayama, Hsiao-Wei Chen, Jieming Chen, Ken Chen, Yiwen Chen, Zhaohong Chen, Andrew D. Cherniack, Jeremy Chien, Yoke-Eng Chiew, Suet-Feung Chin, Juok Cho, Sunghoon Cho, Jung Kyoon Choi, Wan Choi, Christine Chomienne, Zechen Chong, Su Pin Choo, Angela Chou, Angelika N. Christ, Elizabeth L. Christie, Eric Chuah, Carrie Cibulskis, Kristian Cibulskis, Sara Cingarlini, Peter Clapham, Alexander Claviez, Sean Cleary, Nicole Cloonan, Marek Cmero, Colin C. Collins, Ashton A. Connor, Susanna L. Cooke, Colin S. Cooper, Leslie Cope, Vincenzo Corbo, Matthew G. Cordes, Stephen M. Cordner, Isidro Cortés-Ciriano, Kyle Covington, Prue A. Cowin, Brian Craft, David Craft, Chad J. Creighton, Yupeng Cun, Erin Curley, Ioana Cutcutache, Karolina Czajka, Bogdan Czerniak, Rebecca A. Dagg, Ludmila Danilova, Maria Vittoria Davi, Natalie R. Davidson, Helen Davies, Ian J. Davis, Brandi N. Davis-Dusenbery, Kevin J. Dawson, Francisco M. De La Vega, Ricardo De Paoli-Iseppi, Timothy Defreitas, Angelo P. Dei Tos, Olivier Delaneau, John A. Demchok, Jonas Demeulemeester, German M. Demidov, Deniz Demircioğlu, Nening M. Dennis, Robert E. Denroche, Stefan C. Dentro, Nikita Desai, Vikram Deshpande, Amit G. Deshwar, Christine Desmedt, Jordi Deu-Pons, Noreen Dhalla, Neesha C. Dhani, Priyanka Dhingra, Rajiv Dhir, Anthony DiBiase, Klev Diamanti, Li Ding, Shuai Ding, Huy Q. Dinh, Luc Dirix, HarshaVardhan Doddapaneni, Nilgun Donmez, Michelle T. Dow, Ronny Drapkin, Oliver Drechsel, Ruben M. Drews, Serge Serge, Tim Dudderidge, Ana Dueso-Barroso, Andrew J. Dunford, Michael Dunn, Lewis Jonathan Dursi, Fraser R. Duthie, Ken Dutton-Regester, Jenna Eagles, Douglas F. Easton, Stuart Edmonds, Paul A. Edwards, Sandra E. Edwards, Rosalind A. Eeles, Anna Ehinger, Juergen Eils, Roland Eils, Adel El-Naggar, Matthew Eldridge, Kyle Ellrott, Serap Erkek, Georgia Escaramis, Shadrielle M. G. Espiritu, Xavier Estivill, Dariush Etemadmoghadam, Jorunn E. Eyfjord, Bishoy M. Faltas, Daiming Fan, Yu Fan, William C. Faquin, Claudiu Farcas, Matteo Fassan, Aquila Fatima, Francesco Favero, Nodirjon Fayzullaev, Ina Felau, Sian Fereday, Martin L. Ferguson, Vincent Ferretti, Lars Feuerbach, Matthew A. Field, J. Lynn Fink, Gaetano Finocchiaro, Cyril Fisher, Matthew W. Fittall, Anna Fitzgerald, Rebecca C. Fitzgerald, Adrienne M. Flanagan, Neil E. Fleshner, Paul Flicek, John A. Foekens, Kwun M. Fong, Nuno A. Fonseca, Christopher S. Foster, Natalie S. Fox, Michael Fraser, Scott Frazer, Milana Frenkel-Morgenstern, William Friedman, Joan Frigola, Catrina C. Fronick, Akihiro Fujimoto, Masashi Fujita, Masashi Fukayama, Lucinda A. Fulton, Robert S. Fulton, Mayuko Furuta, P. Andrew Futreal, Anja Füllgrabe, Stacey B. Gabriel, Steven Gallinger, Carlo Gambacorti-Passerini, Jianjiong Gao, Shengjie Gao, Levi Garraway, Øystein Garred, Erik Garrison, Dale W. Garsed, Nils Gehlenborg, Josep L. L. Gelpi, Joshy George, Daniela S. Gerhard, Clarissa Gerhauser, Jeffrey E. Gershenwald, Mark Gerstein, Moritz Gerstung, Gad Getz, Mohammed Ghori, Ronald Ghossein, Nasra H. Giama, Richard A. Gibbs, Bob Gibson, Anthony J. Gill, Pelvender Gill, Dilip D. Giri, Dominik Glodzik, Vincent J. Gnanapragasam, Maria Elisabeth Goebler, Mary J. Goldman, Carmen Gomez, Santiago Gonzalez, Abel Gonzalez-Perez, Dmitry A. Gordenin, James Gossage, Kunihito Gotoh, Ramaswamy Govindan, Dorthe Grabau, Janet S. Graham, Robert C. Grant, Anthony R. Green, Eric Green, Liliana Greger, Nicola Grehan, Sonia Grimaldi, Sean M. Grimmond, Robert L. Grossman, Adam Grundhoff, Gunes Gundem, Qianyun Guo, Manaswi Gupta, Shailja Gupta, Ivo G. Gut, Marta Gut, Jonathan Göke, Gavin Ha, Andrea Haake, David Haan, Siegfried Haas, Kerstin Haase, James E. Haber, Nina Habermann, Faraz Hach, Syed Haider, Natsuko Hama, Freddie C. Hamdy, Anne Hamilton, Mark P. Hamilton, Leng Han, George B. Hanna, Martin Hansmann, Nicholas J. Haradhvala, Olivier Harismendy, Ivon Harliwong, Arif O. Harmanci, Eoghan Harrington, Takanori Hasegawa, David Haussler, Steve Hawkins, Shinya Hayami, Shuto Hayashi, D. Neil Hayes, Stephen J. Hayes, Nicholas K. Hayward, Steven Hazell, Yao He, Allison P. Heath, Simon C. Heath, David Hedley, Apurva M. Hegde, David I. Heiman, Michael C. Heinold, Zachary Heins, Lawrence E. Heisler, Eva Hellstrom-Lindberg, Mohamed Helmy, Seong Gu Heo, Austin J. Hepperla, José María Heredia-Genestar, Carl Herrmann, Peter Hersey, Julian M. Hess, Holmfridur Hilmarsdottir, Jonathan Hinton, Satoshi Hirano, Nobuyoshi Hiraoka, Katherine A. Hoadley, Asger Hobolth, Ermin Hodzic, Jessica I. Hoell, Steve Hoffmann, Oliver Hofmann, Andrea Holbrook, Aliaksei Z. Holik, Michael A. Hollingsworth, Oliver Holmes, Robert A. Holt, Chen Hong, Eun Pyo Hong, Jongwhi H. Hong, Gerrit K. Hooijer, Henrik Hornshøj, Fumie Hosoda, Yong Hou, Volker Hovestadt, William Howat, Alan P. Hoyle, Ralph H. Hruban, Jianhong Hu, Taobo Hu, Xing Hua, Kuan-lin Huang, Mei Huang, Mi Ni Huang, Vincent Huang, Yi Huang, Wolfgang Huber, Thomas J. Hudson, Michael Hummel, Jillian A. Hung, David Huntsman, Ted R. Hupp, Jason Huse, Matthew R. Huska, Barbara Hutter, Carolyn M. Hutter, Daniel Hübschmann, Christine A. Iacobuzio-Donahue, Charles David Imbusch, Marcin Imielinski, Seiya Imoto, William B. Isaacs, Keren Isaev, Shumpei Ishikawa, Murat Iskar, S. M. Ashiqul Islam, Michael Ittmann, Sinisa Ivkovic, Jose M. G. Izarzugaza, Jocelyne Jacquemier, Valerie Jakrot, Nigel B. Jamieson, Gun Ho Jang, Se Jin Jang, Joy C. Jayaseelan, Reyka Jayasinghe, Stuart R. Jefferys, Karine Jegalian, Jennifer L. Jennings, Seung-Hyup Jeon, Lara Jerman, Yuan Ji, Wei Jiao, Peter A. Johansson, Amber L. Johns, Jeremy Johns, Rory Johnson, Todd A. Johnson, Clemency Jolly, Yann Joly, Jon G. Jonasson, Corbin D. Jones, David R. Jones, David T. W. Jones, Nic Jones, Steven J. M. Jones, Jos Jonkers, Young Seok Ju, Hartmut Juhl, Jongsun Jung, Malene Juul, Randi Istrup Juul, Sissel Juul, Natalie Jäger, Rolf Kabbe, Andre Kahles, Abdullah Kahraman, Vera B. Kaiser, Hojabr Kakavand, Sangeetha Kalimuthu, Christof von Kalle, Koo Jeong Kang, Katalin Karaszi, Beth Karlan, Rosa Karlić, Dennis Karsch, Katayoon Kasaian, Karin S. Kassahn, Hitoshi Katai, Mamoru Kato, Hiroto Katoh, Yoshiiku Kawakami, Jonathan D. Kay, Stephen H. Kazakoff, Marat D. Kazanov, Maria Keays, Electron Kebebew, Richard F. Kefford, Manolis Kellis, James G. Kench, Catherine J. Kennedy, Jules N. A. Kerssemakers, David Khoo, Vincent Khoo, Narong Khuntikeo, Ekta Khurana, Helena Kilpinen, Hark Kyun Kim, Hyung-Lae Kim, Hyung-Yong Kim, Hyunghwan Kim, Jaegil Kim, Jihoon Kim, Jong K. Kim, Youngwook Kim, Tari A. King, Wolfram Klapper, Kortine Kleinheinz, Leszek J. Klimczak, Stian Knappskog, Michael Kneba, Bartha M. Knoppers, Youngil Koh, Jan Komorowski, Daisuke Komura, Mitsuhiro Komura, Gu Kong, Marcel Kool, Jan O. Korbel, Viktoriya Korchina, Andrey Korshunov, Michael Koscher, Roelof Koster, Zsofia Kote-Jarai, Antonios Koures, Milena Kovacevic, Barbara Kremeyer, Helene Kretzmer, Markus Kreuz, Savitri Krishnamurthy, Dieter Kube, Kiran Kumar, Pardeep Kumar, Sushant Kumar, Yogesh Kumar, Ritika Kundra, Kirsten Kübler, Ralf Küppers, Jesper Lagergren, Phillip H. Lai, Peter W. Laird, Sunil R. Lakhani, Christopher M. Lalansingh, Emilie Lalonde, Fabien C. Lamaze, Adam Lambert, Eric Lander, Pablo Landgraf, Luca Landoni, Anita Langerød, Andrés Lanzós, Denis Larsimont, Erik Larsson, Mark Lathrop, Loretta M. S. Lau, Chris Lawerenz, Rita T. Lawlor, Michael S. Lawrence, Alexander J. Lazar, Ana Mijalkovic Lazic, Xuan Le, Darlene Lee, Donghoon Lee, Eunjung Alice Lee, Hee Jin Lee, Jake June-Koo Lee, Jeong-Yeon Lee, Juhee Lee, Ming Ta Michael Lee, Henry Lee-Six, Kjong-Van Lehmann, Hans Lehrach, Dido Lenze, Conrad R. Leonard, Daniel A. Leongamornlert, Ignaty Leshchiner, Louis Letourneau, Ivica Letunic, Douglas A. Levine, Lora Lewis, Tim Ley, Chang Li, Constance H. Li, Haiyan Irene Li, Jun Li, Lin Li, Shantao Li, Siliang Li, Xiaobo Li, Xiaotong Li, Xinyue Li, Yilong Li, Han Liang, Sheng-Ben Liang, Peter Lichter, Pei Lin, Ziao Lin, W. M. Linehan, Ole Christian Lingjærde, Dongbing Liu, Eric Minwei Liu, Fei-Fei Fei Liu, Fenglin Liu, Jia Liu, Xingmin Liu, Julie Livingstone, Dimitri Livitz, Naomi Livni, Lucas Lochovsky, Markus Loeffler, Georgina V. Long, Armando Lopez-Guillermo, Shaoke Lou, David N. Louis, Laurence B. Lovat, Yiling Lu, Yong-Jie Lu, Youyong Lu, Claudio Luchini, Ilinca Lungu, Xuemei Luo, Hayley J. Luxton, Andy G. Lynch, Lisa Lype, Cristina López, Carlos López-Otín, Eric Z. Ma, Yussanne Ma, Gaetan MacGrogan, Shona MacRae, Geoff Macintyre, Tobias Madsen, Kazuhiro Maejima, Andrea Mafficini, Dennis T. Maglinte, Arindam Maitra, Partha P. Majumder, Luca Malcovati, Salem Malikic, Giuseppe Malleo, Graham J. Mann, Luisa Mantovani-Löffler, Kathleen Marchal, Giovanni Marchegiani, Elaine R. Mardis, Adam A. Margolin, Maximillian G. Marin, Florian Markowetz, Julia Markowski, Jeffrey Marks, Tomas Marques-Bonet, Marco A. Marra, Luke Marsden, John W. M. Martens, Sancha Martin, Jose I. Martin-Subero, Iñigo Martincorena, Alexander Martinez-Fundichely, Yosef E. Maruvka, R. Jay Mashl, Charlie E. Massie, Thomas J. Matthew, Lucy Matthews, Erik Mayer, Simon Mayes, Michael Mayo, Faridah Mbabaali, Karen McCune, Ultan McDermott, Patrick D. McGillivray, Michael D. McLellan, John D. McPherson, John R. McPherson, Treasa A. McPherson, Samuel R. Meier, Alice Meng, Shaowu Meng, Andrew Menzies, Neil D. Merrett, Sue Merson, Matthew Meyerson, William Meyerson, Piotr A. Mieczkowski, George L. Mihaiescu, Sanja Mijalkovic, Tom Mikkelsen, Michele Milella, Linda Mileshkin, Christopher A. Miller, David K. Miller, Jessica K. Miller, Gordon B. Mills, Ana Milovanovic, Sarah Minner, Marco Miotto, Gisela Mir Arnau, Lisa Mirabello, Chris Mitchell, Thomas J. Mitchell, Satoru Miyano, Naoki Miyoshi, Shinichi Mizuno, Fruzsina Molnár-Gábor, Malcolm J. Moore, Richard A. Moore, Sandro Morganella, Quaid D. Morris, Carl Morrison, Lisle E. Mose, Catherine D. Moser, Ferran Muiños, Loris Mularoni, Andrew J. Mungall, Karen Mungall, Elizabeth A. Musgrove, Ville Mustonen, David Mutch, Francesc Muyas, Donna M. Muzny, Alfonso Muñoz, Jerome Myers, Ola Myklebost, Peter Möller, Genta Nagae, Adnan M. Nagrial, Hardeep K. Nahal-Bose, Hitoshi Nakagama, Hidewaki Nakagawa, Hiromi Nakamura, Toru Nakamura, Kaoru Nakano, Tannistha Nandi, Jyoti Nangalia, Mia Nastic, Arcadi Navarro, Fabio C. P. Navarro, David E. Neal, Gerd Nettekoven, Felicity Newell, Steven J. Newhouse, Yulia Newton, Alvin Wei Tian Ng, Anthony Ng, Jonathan Nicholson, David Nicol, Yongzhan Nie, G. Petur Nielsen, Morten Muhlig Nielsen, Serena Nik-Zainal, Michael S. Noble, Katia Nones, Paul A. Northcott, Faiyaz Notta, Brian D. O’Connor, Peter O’Donnell, Maria O’Donovan, Sarah O’Meara, Brian Patrick O’Neill, J. Robert O’Neill, David Ocana, Angelica Ochoa, Layla Oesper, Christopher Ogden, Hideki Ohdan, Kazuhiro Ohi, Lucila Ohno-Machado, Karin A. Oien, Akinyemi I. Ojesina, Hidenori Ojima, Takuji Okusaka, Larsson Omberg, Choon Kiat Ong, Stephan Ossowski, German Ott, B. F. Francis Ouellette, Christine P’ng, Marta Paczkowska, Salvatore Paiella, Chawalit Pairojkul, Marina Pajic, Qiang Pan-Hammarström, Elli Papaemmanuil, Irene Papatheodorou, Nagarajan Paramasivam, Ji Wan Park, Joong-Won Park, Keunchil Park, Kiejung Park, Peter J. Park, Joel S. Parker, Simon L. Parsons, Harvey Pass, Danielle Pasternack, Alessandro Pastore, Ann-Marie Patch, Iris Pauporté, Antonio Pea, John V. Pearson, Chandra Sekhar Pedamallu, Jakob Skou Pedersen, Paolo Pederzoli, Martin Peifer, Nathan A. Pennell, Charles M. Perou, Marc D. Perry, Gloria M. Petersen, Myron Peto, Nicholas Petrelli, Robert Petryszak, Stefan M. Pfister, Mark Phillips, Oriol Pich, Hilda A. Pickett, Todd D. Pihl, Nischalan Pillay, Sarah Pinder, Mark Pinese, Andreia V. Pinho, Esa Pitkänen, Xavier Pivot, Elena Piñeiro-Yáñez, Laura Planko, Christoph Plass, Paz Polak, Tirso Pons, Irinel Popescu, Olga Potapova, Aparna Prasad, Shaun R. Preston, Manuel Prinz, Antonia L. Pritchard, Stephenie D. Prokopec, Elena Provenzano, Xose S. Puente, Sonia Puig, Montserrat Puiggròs, Sergio Pulido-Tamayo, Gulietta M. Pupo, Colin A. Purdie, Michael C. Quinn, Raquel Rabionet, Janet S. Rader, Bernhard Radlwimmer, Petar Radovic, Benjamin Raeder, Keiran M. Raine, Manasa Ramakrishna, Kamna Ramakrishnan, Suresh Ramalingam, Benjamin J. Raphael, W. Kimryn Rathmell, Tobias Rausch, Guido Reifenberger, Jüri Reimand, Jorge Reis-Filho, Victor Reuter, Iker Reyes-Salazar, Matthew A. Reyna, Sheila M. Reynolds, Esther Rheinbay, Yasser Riazalhosseini, Andrea L. Richardson, Julia Richter, Matthew Ringel, Markus Ringnér, Yasushi Rino, Karsten Rippe, Jeffrey Roach, Lewis R. Roberts, Nicola D. Roberts, Steven A. Roberts, A. Gordon Robertson, Alan J. Robertson, Javier Bartolomé Rodriguez, Bernardo Rodriguez-Martin, F. Germán Rodríguez-González, Michael H. A. Roehrl, Marius Rohde, Hirofumi Rokutan, Gilles Romieu, Ilse Rooman, Tom Roques, Daniel Rosebrock, Mara Rosenberg, Philip C. Rosenstiel, Andreas Rosenwald, Edward W. Rowe, Romina Royo, Steven G. Rozen, Yulia Rubanova, Mark A. Rubin, Carlota Rubio-Perez, Vasilisa A. Rudneva, Borislav C. Rusev, Andrea Ruzzenente, Gunnar Rätsch, Radhakrishnan Sabarinathan, Veronica Y. Sabelnykova, Sara Sadeghi, S. Cenk Sahinalp, Natalie Saini, Mihoko Saito-Adachi, Gordon Saksena, Adriana Salcedo, Roberto Salgado, Leonidas Salichos, Richard Sallari, Charles Saller, Roberto Salvia, Michelle Sam, Jaswinder S. Samra, Francisco Sanchez-Vega, Chris Sander, Grant Sanders, Rajiv Sarin, Iman Sarrafi, Aya Sasaki-Oku, Torill Sauer, Guido Sauter, Robyn P. M. Saw, Maria Scardoni, Christopher J. Scarlett, Aldo Scarpa, Ghislaine Scelo, Dirk Schadendorf, Jacqueline E. Schein, Markus B. Schilhabel, Matthias Schlesner, Thorsten Schlomm, Heather K. Schmidt, Sarah-Jane Schramm, Stefan Schreiber, Nikolaus Schultz, Steven E. Schumacher, Roland F. Schwarz, Richard A. Scolyer, David Scott, Ralph Scully, Raja Seethala, Ayellet V. Segre, Iris Selander, Colin A. Semple, Yasin Senbabaoglu, Subhajit Sengupta, Elisabetta Sereni, Stefano Serra, Dennis C. Sgroi, Mark Shackleton, Nimish C. Shah, Sagedeh Shahabi, Catherine A. Shang, Ping Shang, Ofer Shapira, Troy Shelton, Ciyue Shen, Hui Shen, Rebecca Shepherd, Ruian Shi, Yan Shi, Yu-Jia Shiah, Tatsuhiro Shibata, Juliann Shih, Eigo Shimizu, Kiyo Shimizu, Seung Jun Shin, Yuichi Shiraishi, Tal Shmaya, Ilya Shmulevich, Solomon I. Shorser, Charles Short, Raunak Shrestha, Suyash S. Shringarpure, Craig Shriver, Shimin Shuai, Nikos Sidiropoulos, Reiner Siebert, Anieta M. Sieuwerts, Lina Sieverling, Sabina Signoretti, Katarzyna O. Sikora, Michele Simbolo, Ronald Simon, Janae V. Simons, Jared T. Simpson, Peter T. Simpson, Samuel Singer, Nasa Sinnott-Armstrong, Payal Sipahimalani, Tara J. Skelly, Marcel Smid, Jaclyn Smith, Karen Smith-McCune, Nicholas D. Socci, Heidi J. Sofia, Matthew G. Soloway, Lei Song, Anil K. Sood, Sharmila Sothi, Christos Sotiriou, Cameron M. Soulette, Paul N. Span, Paul T. Spellman, Nicola Sperandio, Andrew J. Spillane, Oliver Spiro, Jonathan Spring, Johan Staaf, Peter F. Stadler, Peter Staib, Stefan G. Stark, Lucy Stebbings, Ólafur Andri Stefánsson, Oliver Stegle, Lincoln D. Stein, Alasdair Stenhouse, Chip Stewart, Stephan Stilgenbauer, Miranda D. Stobbe, Michael R. Stratton, Jonathan R. Stretch, Adam J. Struck, Joshua M. Stuart, Henk G. Stunnenberg, Hong Su, Xiaoping Su, Ren X. Sun, Stephanie Sungalee, Hana Susak, Akihiro Suzuki, Fred Sweep, Monika Szczepanowski, Holger Sültmann, Takashi Yugawa, Angela Tam, David Tamborero, Benita Kiat Tee Tan, Donghui Tan, Patrick Tan, Hiroko Tanaka, Hirokazu Taniguchi, Tomas J. Tanskanen, Maxime Tarabichi, Roy Tarnuzzer, Patrick Tarpey, Morgan L. Taschuk, Kenji Tatsuno, Simon Tavaré, Darrin F. Taylor, Amaro Taylor-Weiner, Jon W. Teague, Bin Tean Teh, Varsha Tembe, Javier Temes, Kevin Thai, Sarah P. Thayer, Nina Thiessen, Gilles Thomas, Sarah Thomas, Alan Thompson, Alastair M. Thompson, John F. F. Thompson, R. Houston Thompson, Heather Thorne, Leigh B. Thorne, Adrian Thorogood, Grace Tiao, Nebojsa Tijanic, Lee E. Timms, Roberto Tirabosco, Marta Tojo, Stefania Tommasi, Christopher W. Toon, Umut H. Toprak, David Torrents, Giampaolo Tortora, Jörg Tost, Yasushi Totoki, David Townend, Nadia Traficante, Isabelle Treilleux, Jean-Rémi Trotta, Lorenz H. P. Trümper, Ming Tsao, Tatsuhiko Tsunoda, Jose M. C. Tubio, Olga Tucker, Richard Turkington, Daniel J. Turner, Andrew Tutt, Masaki Ueno, Naoto T. Ueno, Christopher Umbricht, Husen M. Umer, Timothy J. Underwood, Lara Urban, Tomoko Urushidate, Tetsuo Ushiku, Liis Uusküla-Reimand, Alfonso Valencia, David J. Van Den Berg, Steven Van Laere, Peter Van Loo, Erwin G. Van Meir, Gert G. Van den Eynden, Theodorus Van der Kwast, Naveen Vasudev, Miguel Vazquez, Ravikiran Vedururu, Umadevi Veluvolu, Shankar Vembu, Lieven P. C. Verbeke, Peter Vermeulen, Clare Verrill, Alain Viari, David Vicente, Caterina Vicentini, K. VijayRaghavan, Juris Viksna, Ricardo E. Vilain, Izar Villasante, Anne Vincent-Salomon, Tapio Visakorpi, Douglas Voet, Paresh Vyas, Ignacio Vázquez-García, Nick M. Waddell, Nicola Waddell, Claes Wadelius, Lina Wadi, Rabea Wagener, Jeremiah A. Wala, Jian Wang, Jiayin Wang, Linghua Wang, Qi Wang, Wenyi Wang, Yumeng Wang, Zhining Wang, Paul M. Waring, Hans-Jörg Warnatz, Jonathan Warrell, Anne Y. Warren, Sebastian M. Waszak, David C. Wedge, Dieter Weichenhan, Paul Weinberger, John N. Weinstein, Joachim Weischenfeldt, Daniel J. Weisenberger, Ian Welch, Michael C. Wendl, Johannes Werner, Justin P. Whalley, David A. Wheeler, Hayley C. Whitaker, Dennis Wigle, Matthew D. Wilkerson, Ashley Williams, James S. Wilmott, Gavin W. Wilson, Julie M. Wilson, Richard K. Wilson, Boris Winterhoff, Jeffrey A. Wintersinger, Maciej Wiznerowicz, Stephan Wolf, Bernice H. Wong, Tina Wong, Winghing Wong, Youngchoon Woo, Scott Wood, Bradly G. Wouters, Adam J. Wright, Derek W. Wright, Mark H. Wright, Chin-Lee Wu, Dai-Ying Wu, Guanming Wu, Jianmin Wu, Kui Wu, Yang Wu, Zhenggang Wu, Liu Xi, Tian Xia, Qian Xiang, Xiao Xiao, Rui Xing, Heng Xiong, Qinying Xu, Yanxun Xu, Hong Xue, Shinichi Yachida, Sergei Yakneen, Rui Yamaguchi, Takafumi N. Yamaguchi, Masakazu Yamamoto, Shogo Yamamoto, Hiroki Yamaue, Fan Yang, Huanming Yang, Jean Y. Yang, Liming Yang, Lixing Yang, Shanlin Yang, Tsun-Po Yang, Yang Yang, Xiaotong Yao, Marie-Laure Yaspo, Lucy Yates, Christina Yau, Chen Ye, Kai Ye, Venkata D. Yellapantula, Christopher J. Yoon, Sung-Soo Yoon, Fouad Yousif, Jun Yu, Kaixian Yu, Willie Yu, Yingyan Yu, Ke Yuan, Yuan Yuan, Denis Yuen, Christina K. Yung, Olga Zaikova, Jorge Zamora, Marc Zapatka, Jean C. Zenklusen, Thorsten Zenz, Nikolajs Zeps, Cheng-Zhong Zhang, Fan Zhang, Hailei Zhang, Hongwei Zhang, Hongxin Zhang, Jiashan Zhang, Jing Zhang, Junjun Zhang, Xiuqing Zhang, Xuanping Zhang, Yan Zhang, Zemin Zhang, Zhongming Zhao, Liangtao Zheng, Xiuqing Zheng, Wanding Zhou, Yong Zhou, Bin Zhu, Hongtu Zhu, Jingchun Zhu, Shida Zhu, Lihua Zou, Xueqing Zou, Anna deFazio, Nicholas van As, Carolien H. M. van Deurzen, Marc J. van de Vijver, L. van’t Veer, Christian von Mering

**Affiliations:** 1 Department of Surgery, Division of Urology, Royal Melbourne Hospital and University of Melbourne, Parkville, VIC 3050 Australia; 2 The Epworth Prostate Centre, Epworth Hospital, Richmond, VIC 3121 Australia; 3 Department of Computing and Information Systems, University of Melbourne, Parkville, VIC 3010 Australia; 4 Bioinformatics Division, The Walter and Eliza Hall Institute of Medical Research, Parkville, VIC Australia; 5 Murdoch Children’s Research Institute, Parkville, VIC 3052 Australia; 6 School of Computing Science, University of Glasgow, Sir Alwyn Williams Building, Glasgow, G12 8RZ UK; 7 Cancer Research UK Cambridge Institute, University of Cambridge, Cambridge, CB2 0RE UK; 8 Electrical and Electronic Engineering, University of Melbourne, Parkville, VIC 3010 Australia; 9 Machine Learning Research Group, Data61, Canberra, ACT 2601 Australia; 10 Research School of Computer Science, Australian National University, Canberra, ACT 2601 Australia; 11 Wellcome Sanger Institute, Wellcome Genome Campus, Hinxton, Cambridge, CB10 1SA UK; 12 Molecular and Medical Genetics, Oregon Health and Science University, Portland, OR 97201 USA; 13 Broad Institute of MIT and Harvard, Cambridge, MA 02142 USA; 14 Department of Medical Oncology, Dana-Farber Cancer Institute, Boston, MA 02115 USA; 15 Harvard Medical School, Boston, MA 02115 USA; 16 Computational Biology Program, Ontario Institute for Cancer Research, Toronto, ON M5G 0A3 Canada; 17 Department of Medical Biophysics, University of Toronto, Toronto, ON M5S 1A8 Canada; 18 Department of Pharmacology, University of Toronto, Toronto, ON M5S 1A8 Canada; 19 University of California Los Angeles, Los Angeles, CA 90095 USA; 20 Peter MacCallum Cancer Centre, Melbourne, VIC 3000 Australia; 21 Sir Peter MacCallum Department of Oncology, University of Melbourne, Melbourne, VIC 3052 Australia; 22 Department of Haematology, University of Cambridge, Cambridge, CB2 2XY UK; 23 Department of Bioinformatics and Computational Biology, The University of Texas MD Anderson Cancer Center, Houston, TX 77030 USA; 24 University of Cologne, 50931 Cologne, Germany; 25 University of Leuven, B-3000 Leuven, Belgium; 26 The Francis Crick Institute, London, NW1 1AT UK; 27 Big Data Institute, Li Ka Shing Centre, University of Oxford, Oxford, OX3 7LF UK; 28 The Edward S. Rogers Sr. Department of Electrical and Computer Engineering, University of Toronto, Toronto, ON M5S 3G4 Canada; 29 Simon Fraser University, Burnaby, BC V5A 1S6 Canada; 30 Vancouver Prostate Centre, Vancouver, BC V6H 3Z6 Canada; 31 Cancer Research UK Cambridge Institute, University of Cambridge, Cambridge, CB2 0RE UK; 32 Division of Theoretical Bioinformatics, German Cancer Research Center (DKFZ), 69120 Heidelberg, Germany; 33 Heidelberg University, 69120 Heidelberg, Germany; 34 Institute of Pharmacy and Molecular Biotechnology and BioQuant, Heidelberg University, 69120 Heidelberg, Germany; 35 New BIH Digital Health Center, Berlin Institute of Health (BIH) and Charité - Universitätsmedizin Berlin, 10117 Berlin, Germany; 36 Sir Peter MacCallum Department of Oncology, The University of Melbourne, Melbourne, VIC 3052 Australia; 37 European Molecular Biology Laboratory, European Bioinformatics Institute (EMBL-EBI), Wellcome Genome Campus, Hinxton, Cambridge, CB10 1SD UK; 38 Genome Biology Unit, European Molecular Biology Laboratory (EMBL), 69117 Heidelberg, Germany; 39 Center for Cancer Research, Massachusetts General Hospital, Boston, MA 02129 USA; 40 Department of Pathology, Massachusetts General Hospital, Boston, MA 02115 USA; 41 New York Genome Center, New York, NY 10013 USA; 42 Weill Cornell Medicine, New York, NY 10065 USA; 43 University of Ljubljana, 1000 Ljubljana, Slovenia; 44 Research Institute, NorthShore University HealthSystem, Evanston, IL 60201 USA; 45 Department of Public Health Sciences, The University of Chicago, Chicago, IL 60637 USA; 46 Department of Statistics, University of California Santa Cruz, Santa Cruz, CA 95064 USA; 47 University of Cambridge, Cambridge, CB2 1TN UK; 48 Cambridge University Hospitals NHS Foundation Trust, Cambridge, CB2 0QQ UK; 49 University of Toronto, Toronto, ON M5G 2M9 Canada; 50 Vector Institute, Toronto, ON M5G 0A3 Canada; 51 Department of Computer Science, University of Helsinki, 00014 Helsinki, Finland; 52 Institute of Biotechnology, University of Helsinki, 00014 Helsinki, Finland; 53 Organismal and Evolutionary Biology Research Programme, University of Helsinki, 00014 Helsinki, Finland; 54 Department of Computer Science, Carleton College, Northfield, MN 55057 USA; 55 Molecular and Medical Genetics, Oregon Health & Science University, Portland, OR 97239 USA; 56 Department of Computer Science, Princeton University, Princeton, NJ 08540 USA; 57 Department of Computer Science, University of Toronto, Toronto, ON M5S 1A8 Canada; 58 Indiana University, Bloomington, IN 47405 USA; 59 Bioinformatics and Omics Data Analytics, German Cancer Research Center (DKFZ), 69120 Heidelberg, Germany; 60 Department of Cancer Biology, Dana-Farber Cancer Institute, Boston, MA 02215 USA; 61 Center for Psychiatric Genetics, NorthShore University HealthSystem, Evanston, IL 60201 USA; 62 Korea University, Seoul, 02481 South Korea; 63 Molecular and Medical Genetics, Knight Cancer Institute, Oregon Health & Science University, Portland, OR 97219 USA; 64 Department of Molecular Genetics, University of Toronto, Toronto, ON M5S 1A8 Canada; 65 Argmix Consulting, North Vancouver, BC V7M 2J5 Canada; 66 Department of Applied Mathematics and Theoretical Physics, Centre for Mathematical Sciences, University of Cambridge, Cambridge, CB3 0WA UK; 67 Department of Epidemiology and Biostatistics, Memorial Sloan Kettering Cancer Center, New York, NY 10065 USA; 68 Department of Statistics, Columbia University, New York, NY 10027 USA; 69 Oxford NIHR Biomedical Research Centre, University of Oxford, Oxford, OX4 2PG UK; 70 Department of Molecular and Human Genetics, Baylor College of Medicine, Houston, TX 77030 USA; 71 Human Genome Sequencing Center, Baylor College of Medicine, Houston, TX 77030 USA; 72 The Donnelly Centre, University of Toronto, Toronto, ON M5S 3E1 Canada; 73 Department of Computer Science, University of Toronto, Toronto, ON M5S 2E4 Canada; 74 Tri-institutional PhD Program of Computational Biology and Medicine, Weill Cornell Medicine, New York, NY 10065 USA; 75 Department of Biostatistics, The University of Texas MD Anderson Cancer Center, Houston, TX 77030 USA; 76 Department of Biostatistics, University of North Carolina at Chapel Hill, Chapel Hill, NC 27599 USA; 77 The University of Texas MD Anderson Cancer Center, Houston, TX 77030 USA; 200 Applied Tumor Genomics Research Program, Research Programs Unit, University of Helsinki, Helsinki, Finland; 201 Wellcome Sanger Institute, Wellcome Genome Campus, Hinxton, UK; 202 Memorial Sloan Kettering Cancer Center, New York, NY USA; 203 Genome Science Division, Research Center for Advanced Science and Technology, University of Tokyo, Tokyo, Japan; 204 Department of Surgery, University of Chicago, Chicago, IL USA; 205 Department of Surgery, Division of Hepatobiliary and Pancreatic Surgery, School of Medicine, Keimyung University Dongsan Medical Center, Daegu, South Korea; 206 Department of Oncology, Gil Medical Center, Gachon University, Incheon, South Korea; 207 Hiroshima University, Hiroshima, Japan; 208 Department of Bioinformatics and Computational Biology, The University of Texas MD Anderson Cancer Center, Houston, TX USA; 209 University of Texas MD Anderson Cancer Center, Houston, TX USA; 210 King Faisal Specialist Hospital and Research Centre, Al Maather, Riyadh, Saudi Arabia; 211 Bioinformatics Unit, Spanish National Cancer Research Centre (CNIO), Madrid, Spain; 212 Bioinformatics Core Facility, University Medical Center Hamburg, Hamburg, Germany; 213 Heinrich Pette Institute, Leibniz Institute for Experimental Virology, Hamburg, Germany; 214 Ontario Tumour Bank, Ontario Institute for Cancer Research, Toronto, ON Canada; 215 Department of Pathology, The University of Texas MD Anderson Cancer Center, Houston, TX USA; 216 Laboratory of Pathology, Center for Cancer Research, National Cancer Institute, Bethesda, MD USA; 217 Department of Cellular and Molecular Medicine and Department of Bioengineering, University of California San Diego, La Jolla, CA USA; 218 UC San Diego Moores Cancer Center, San Diego, CA USA; 219 Canada’s Michael Smith Genome Sciences Centre, BC Cancer, Vancouver, BC Canada; 220 Sir Peter MacCallum Department of Oncology, Peter MacCallum Cancer Centre, University of Melbourne, Melbourne, VIC Australia; 221 Centre for Research in Molecular Medicine and Chronic Diseases (CiMUS), Universidade de Santiago de Compostela, Santiago de Compostela, Spain; 222 Department of Zoology, Genetics and Physical Anthropology, (CiMUS), Universidade de Santiago de Compostela, Santiago de Compostela, Spain; 223 The Biomedical Research Centre (CINBIO), Universidade de Vigo, Vigo, Spain; 224 Royal National Orthopaedic Hospital - Bolsover, London, UK; 225 Department of Genomic Medicine, The University of Texas MD Anderson Cancer Center, Houston, TX USA; 226 Quantitative and Computational Biosciences Graduate Program, Baylor College of Medicine, Houston, TX USA; 227 The Jackson Laboratory for Genomic Medicine, Farmington, CT USA; 228 Genome Informatics Program, Ontario Institute for Cancer Research, Toronto, ON Canada; 229 Institute of Human Genetics, Christian-Albrechts-University, Kiel, Germany; 230 Institute of Human Genetics, Ulm University and Ulm University Medical Center, Ulm, Germany; 231 Queensland Centre for Medical Genomics, Institute for Molecular Bioscience, University of Queensland, St. Lucia, Brisbane, QLD Australia; 232 Salford Royal NHS Foundation Trust, Salford, UK; 233 Department of Surgery, Pancreas Institute, University and Hospital Trust of Verona, Verona, Italy; 234 Molecular and Medical Genetics, OHSU Knight Cancer Institute, Oregon Health and Science University, Portland, OR USA; 235 Department of Molecular Oncology, BC Cancer Research Centre, Vancouver, BC Canada; 236 The McDonnell Genome Institute at Washington University, St. Louis, MO USA; 237 University College London, London, UK; 238 Division of Cancer Genomics, National Cancer Center Research Institute, National Cancer Center, Tokyo, Japan; 239 DLR Project Management Agency, Bonn, Germany; 240 Tokyo Women’s Medical University, Tokyo, Japan; 241 Center for Molecular Oncology, Memorial Sloan Kettering Cancer Center, New York, NY USA; 242 Los Alamos National Laboratory, Los Alamos, NM USA; 243 Department of Pathology, University Health Network, Toronto General Hospital, Toronto, ON Canada; 244 Nottingham University Hospitals NHS Trust, Nottingham, UK; 245 Epigenomics and Cancer Risk Factors, German Cancer Research Center (DKFZ), Heidelberg, Germany; 246 Computational Biology Program, Ontario Institute for Cancer Research, Toronto, ON Canada; 247 Department of Molecular Genetics, University of Toronto, Toronto, ON Canada; 248 Vector Institute, Toronto, ON Canada; 249 Hematopathology Section, Institute of Pathology, Christian-Albrechts-University, Kiel, Germany; 250 Department of Pathology and Laboratory Medicine, School of Medicine, University of North Carolina at Chapel Hill, Chapel Hill, NC USA; 251 Department of Cancer Genetics, Institute for Cancer Research, Oslo University Hospital, The Norwegian Radium Hospital, Oslo, Norway; 252 Pathology, Hospital Clinic, Institut d’Investigacions Biomèdiques August Pi i Sunyer (IDIBAPS), University of Barcelona, Barcelona, Spain; 253 Department of Veterinary Medicine, Transmissible Cancer Group, University of Cambridge, Cambridge, UK; 254 Alvin J. Siteman Cancer Center, Washington University School of Medicine, St. Louis, MO USA; 255 Wolfson Wohl Cancer Research Centre, Institute of Cancer Sciences, University of Glasgow, Glasgow, UK; 256 Lineberger Comprehensive Cancer Center, University of North Carolina at Chapel Hill, Chapel Hill, NC USA; 257 Broad Institute of MIT and Harvard, Cambridge, MA USA; 258 Dana-Farber/Boston Children’s Cancer and Blood Disorders Center, Boston, MA USA; 259 Department of Pediatrics, Harvard Medical School, Boston, MA USA; 260 Leeds Institute of Medical Research @ St. James’s, University of Leeds, St. James’s University Hospital, Leeds, UK; 261 Department of Pathology and Diagnostics, University and Hospital Trust of Verona, Verona, Italy; 262 Department of Surgery, Princess Alexandra Hospital, Brisbane, QLD Australia; 263 Surgical Oncology Group, Diamantina Institute, University of Queensland, Brisbane, QLD Australia; 264 Department of Population and Quantitative Health Sciences, Case Western Reserve University School of Medicine, Cleveland, OH USA; 265 Research Health Analytics and Informatics, University Hospitals Cleveland Medical Center, Cleveland, OH USA; 266 Gloucester Royal Hospital, Gloucester, UK; 267 European Molecular Biology Laboratory, European Bioinformatics Institute (EMBL-EBI), Cambridge, UK; 268 Diagnostic Development, Ontario Institute for Cancer Research, Toronto, ON Canada; 269 Barcelona Supercomputing Center (BSC), Barcelona, Spain; 270 Arnie Charbonneau Cancer Institute, University of Calgary, Calgary, AB Canada; 271 Departments of Surgery and Oncology, University of Calgary, Calgary, AB Canada; 272 Department of Pathology, Oslo University Hospital, The Norwegian Radium Hospital, Oslo, Norway; 273 PanCuRx Translational Research Initiative, Ontario Institute for Cancer Research, Toronto, ON Canada; 274 Department of Oncology, Sidney Kimmel Comprehensive Cancer Center at Johns Hopkins University School of Medicine, Baltimore, MD USA; 275 University Hospital Southampton NHS Foundation Trust, Southampton, UK; 276 Royal Stoke University Hospital, Stoke-on-Trent, UK; 277 Genome Sequence Informatics, Ontario Institute for Cancer Research, Toronto, ON Canada; 278 Human Longevity Inc, San Diego, CA USA; 279 Olivia Newton-John Cancer Research Institute, La Trobe University, Heidelberg, VIC Australia; 280 Computer Network Information Center, Chinese Academy of Sciences, Beijing, China; 281 Genome Canada, Ottawa, ON Canada; 282 CNAG-CRG, Centre for Genomic Regulation (CRG), Barcelona Institute of Science and Technology (BIST), Barcelona, Spain; 283 Universitat Pompeu Fabra (UPF), Barcelona, Spain; 284 Buck Institute for Research on Aging, Novato, CA USA; 285 Duke University Medical Center, Durham, NC USA; 286 Department of Human Genetics, Hannover Medical School, Hannover, Germany; 287 Center for Bioinformatics and Functional Genomics, Cedars-Sinai Medical Center, Los Angeles, CA USA; 288 Department of Biomedical Sciences, Cedars-Sinai Medical Center, Los Angeles, CA USA; 289 The Hebrew University Faculty of Medicine, Jerusalem, Israel; 290 Barts Cancer Institute, Barts and the London School of Medicine and Dentistry, Queen Mary University of London, London, UK; 291 Department of Computer Science, Bioinformatics Group, University of Leipzig, Leipzig, Germany; 292 Interdisciplinary Center for Bioinformatics, University of Leipzig, Leipzig, Germany; 293 Transcriptome Bioinformatics, LIFE Research Center for Civilization Diseases, University of Leipzig, Leipzig, Germany; 294 Department of Medical Oncology, Dana-Farber Cancer Institute, Boston, MA USA; 295 Department of Cancer Biology, Dana-Farber Cancer Institute, Boston, MA USA; 296 Harvard Medical School, Boston, MA USA; 297 USC Norris Comprehensive Cancer Center, University of Southern California, Los Angeles, CA USA; 298 Department of Diagnostics and Public Health, University and Hospital Trust of Verona, Verona, Italy; 299 Department of Mathematics, Aarhus University, Aarhus, Denmark; 300 Department of Molecular Medicine (MOMA), Aarhus University Hospital, Aarhus N, Denmark; 301 Instituto Carlos Slim de la Salud, Mexico City, Mexico; 302 Department of Medical Biophysics, University of Toronto, Toronto, ON Canada; 303 Cancer Division, Garvan Institute of Medical Research, Kinghorn Cancer Centre, University of New South Wales (UNSW Sydney), Sydney, NSW Australia; 304 South Western Sydney Clinical School, Faculty of Medicine, University of New South Wales (UNSW Sydney), Liverpool, NSW Australia; 305 West of Scotland Pancreatic Unit, Glasgow Royal Infirmary, Glasgow, UK; 306 Center for Digital Health, Berlin Institute of Health and Charitè - Universitätsmedizin Berlin, Berlin, Germany; 307 Heidelberg Center for Personalized Oncology (DKFZ-HIPO), German Cancer Research Center (DKFZ), Heidelberg, Germany; 308 The Preston Robert Tisch Brain Tumor Center, Duke University Medical Center, Durham, NC USA; 309 Massachusetts General Hospital, Boston, MA USA; 310 National Institute of Biomedical Genomics, Kalyani, West Bengal India; 311 Institute of Clinical Medicine and Institute of Oral Biology, University of Oslo, Oslo, Norway; 312 University of North Carolina at Chapel Hill, Chapel Hill, NC USA; 313 ARC-Net Centre for Applied Research on Cancer, University and Hospital Trust of Verona, Verona, Italy; 314 The Institute of Cancer Research, London, UK; 315 Centre for Computational Biology, Duke-NUS Medical School, Singapore, Singapore; 316 Programme in Cancer and Stem Cell Biology, Duke-NUS Medical School, Singapore, Singapore; 317 Division of Oncology and Pathology, Department of Clinical Sciences Lund, Lund University, Lund, Sweden; 318 Department of Pediatric Oncology, Hematology and Clinical Immunology, Heinrich-Heine-University, Düsseldorf, Germany; 319 Laboratory for Medical Science Mathematics, RIKEN Center for Integrative Medical Sciences, Yokohama, Japan; 320 RIKEN Center for Integrative Medical Sciences, Yokohama, Japan; 321 Department of Internal Medicine/Hematology, Friedrich-Ebert-Hospital, Neumünster, Germany; 322 Departments of Dermatology and Pathology, Yale University, New Haven, CT USA; 323 Centre for Genomic Regulation (CRG), The Barcelona Institute of Science and Technology, Barcelona, Spain; 324 Radcliffe Department of Medicine, University of Oxford, Oxford, UK; 325 Canadian Center for Computational Genomics, McGill University, Montreal, QC Canada; 326 Department of Human Genetics, McGill University, Montreal, QC Canada; 327 Department of Human Genetics, University of California Los Angeles, Los Angeles, CA USA; 328 Department of Pharmacology, University of Toronto, Toronto, ON Canada; 329 Faculty of Medicine and Health Technology, Tampere University and Tays Cancer Center, Tampere University Hospital, Tampere, Finland; 330 Haematology, Leeds Teaching Hospitals NHS Trust, Leeds, UK; 331 Translational Research and Innovation, Centre Léon Bérard, Lyon, France; 332 Fox Chase Cancer Center, Philadelphia, PA USA; 333 International Agency for Research on Cancer, World Health Organization, Lyon, France; 334 Earlham Institute, Norwich, UK; 335 Norwich Medical School, University of East Anglia, Norwich, UK; 336 Department of Molecular Biology, Faculty of Science, Radboud Institute for Molecular Life Sciences, Radboud University, Nijmegen, HB The Netherlands; 337 CRUK Manchester Institute and Centre, Manchester, UK; 338 Department of Radiation Oncology, University of Toronto, Toronto, ON Canada; 339 Division of Cancer Sciences, Manchester Cancer Research Centre, University of Manchester, Manchester, UK; 340 Radiation Medicine Program, Princess Margaret Cancer Centre, Toronto, ON Canada; 341 Department of Pathology, Brigham and Women’s Hospital, Harvard Medical School, Boston, MA USA; 342 Department of Surgery, Division of Thoracic Surgery, The Johns Hopkins University School of Medicine, Baltimore, MD USA; 343 Division of Molecular Pathology, The Netherlands Cancer Institute, Oncode Institute, Amsterdam, CX The Netherlands; 344 Department of Biomolecular Engineering, University of California Santa Cruz, Santa Cruz, CA USA; 345 UC Santa Cruz Genomics Institute, University of California Santa Cruz, Santa Cruz, CA USA; 346 Division of Applied Bioinformatics, German Cancer Research Center (DKFZ), Heidelberg, Germany; 347 German Cancer Consortium (DKTK), German Cancer Research Center (DKFZ), Heidelberg, Germany; 348 National Center for Tumor Diseases (NCT) Heidelberg, Heidelberg, Germany; 349 Center for Biological Sequence Analysis, Department of Bio and Health Informatics, Technical University of Denmark, Lyngby, Denmark; 350 Novo Nordisk Foundation Center for Protein Research, University of Copenhagen, Copenhagen, Denmark; 351 Institute for Molecular Bioscience, University of Queensland, St. Lucia, Brisbane, QLD Australia; 352 Biomedical Engineering, Oregon Health and Science University, Portland, OR USA; 353 Division of Theoretical Bioinformatics, German Cancer Research Center (DKFZ), Heidelberg, Germany; 354 Institute of Pharmacy and Molecular Biotechnology and BioQuant, Heidelberg University, Heidelberg, Germany; 355 Federal Ministry of Education and Research, Berlin, Germany; 356 Melanoma Institute Australia, University of Sydney, Sydney, NSW Australia; 357 Pediatric Hematology and Oncology, University Hospital Muenster, Muenster, Germany; 358 Department of Pathology, Johns Hopkins University School of Medicine, Baltimore, MD USA; 359 McKusick-Nathans Institute of Genetic Medicine, Sidney Kimmel Comprehensive Cancer Center at Johns Hopkins University School of Medicine, Baltimore, MD USA; 360 Foundation Medicine, Inc, Cambridge, MA USA; 361 Department of Biomedical Data Science, Stanford University School of Medicine, Stanford, CA USA; 362 Department of Genetics, Stanford University School of Medicine, Stanford, CA USA; 363 Bakar Computational Health Sciences Institute and Department of Pediatrics, University of California, San Francisco, CA USA; 364 Institute of Clinical Medicine, Faculty of Medicine, University of Oslo, Oslo, Norway; 365 National Cancer Institute, National Institutes of Health, Bethesda, MD USA; 366 Royal Marsden NHS Foundation Trust, London and Sutton, UK; 367 Genome Biology Unit, European Molecular Biology Laboratory (EMBL), Heidelberg, Germany; 368 Department of Oncology, University of Cambridge, Cambridge, UK; 369 Li Ka Shing Centre, Cancer Research UK Cambridge Institute, University of Cambridge, Cambridge, UK; 370 Institut Gustave Roussy, Villejuif, France; 371 Cambridge University Hospitals NHS Foundation Trust, Cambridge, UK; 372 Department of Haematology, University of Cambridge, Cambridge, UK; 373 Anatomia Patológica, Hospital Clinic, Institut d’Investigacions Biomèdiques August Pi i Sunyer (IDIBAPS), University of Barcelona, Barcelona, Spain; 374 Spanish Ministry of Science and Innovation, Madrid, Spain; 375 University of Michigan Comprehensive Cancer Center, Ann Arbor, MI USA; 376 Department for BioMedical Research, University of Bern, Bern, Switzerland; 377 Department of Medical Oncology, Inselspital, University Hospital and University of Bern, Bern, Switzerland; 378 Graduate School for Cellular and Biomedical Sciences, University of Bern, Bern, Switzerland; 379 University of Pavia, Pavia, Italy; 380 University of Alabama at Birmingham, Birmingham, AL USA; 381 UHN Program in BioSpecimen Sciences, Toronto General Hospital, Toronto, ON Canada; 382 Department of Urology, Icahn School of Medicine at Mount Sinai, New York, NY USA; 383 Centre for Law and Genetics, University of Tasmania, Sandy Bay Campus, Hobart, TAS Australia; 384 Faculty of Biosciences, Heidelberg University, Heidelberg, Germany; 385 Department of Biochemistry, Microbiology and Immunology, Faculty of Medicine, University of Ottawa, Ottawa, ON Canada; 386 Division of Anatomic Pathology, Mayo Clinic, Rochester, MN USA; 387 Division of Cancer Epidemiology and Genetics, National Cancer Institute, National Institutes of Health, Bethesda, MD USA; 388 Illawarra Shoalhaven Local Health District L3 Illawarra Cancer Care Centre, Wollongong Hospital, Wollongong, NSW Australia; 389 BioForA, French National Institute for Agriculture, Food, and Environment (INRAE), ONF, Orléans, France; 390 Department of Biostatistics, Bloomberg School of Public Health, Johns Hopkins University, Baltimore, MD USA; 391 University of California San Diego, San Diego, CA USA; 392 Division of Experimental Pathology, Mayo Clinic, Rochester, MN USA; 393 Centre for Cancer Research, The Westmead Institute for Medical Research, University of Sydney, Sydney, NSW Australia; 394 Department of Gynaecological Oncology, Westmead Hospital, Sydney, NSW Australia; 395 PDXen Biosystems Inc, Seoul, South Korea; 396 Korea Advanced Institute of Science and Technology, Daejeon, South Korea; 397 Electronics and Telecommunications Research Institute, Daejeon, South Korea; 398 Institut National du Cancer (INCA), Boulogne-Billancourt, France; 399 Department of Genetics, Informatics Institute, University of Alabama at Birmingham, Birmingham, AL USA; 400 Division of Medical Oncology, National Cancer Centre, Singapore, Singapore; 401 Medical Oncology, University and Hospital Trust of Verona, Verona, Italy; 402 Department of Pediatrics, University Hospital Schleswig-Holstein, Kiel, Germany; 403 Hepatobiliary/Pancreatic Surgical Oncology Program, University Health Network, Toronto, ON Canada; 404 School of Biological Sciences, University of Auckland, Auckland, New Zealand; 405 Department of Surgery, University of Melbourne, Parkville, VIC Australia; 406 The Murdoch Children’s Research Institute, Royal Children’s Hospital, Parkville, VIC Australia; 407 Walter and Eliza Hall Institute, Parkville, VIC Australia; 408 Vancouver Prostate Centre, Vancouver, Canada; 409 Lunenfeld-Tanenbaum Research Institute, Mount Sinai Hospital, Toronto, ON Canada; 410 University of East Anglia, Norwich, UK; 411 Norfolk and Norwich University Hospital NHS Trust, Norwich, UK; 412 Victorian Institute of Forensic Medicine, Southbank, VIC Australia; 413 Department of Biomedical Informatics, Harvard Medical School, Boston, MA USA; 414 Department of Chemistry, Centre for Molecular Science Informatics, University of Cambridge, Cambridge, UK; 415 Ludwig Center at Harvard Medical School, Boston, MA USA; 416 Human Genome Sequencing Center, Baylor College of Medicine, Houston, TX USA; 417 Peter MacCallum Cancer Centre, University of Melbourne, Melbourne, VIC Australia; 418 Physics Division, Optimization and Systems Biology Lab, Massachusetts General Hospital, Boston, MA USA; 419 Department of Medicine, Baylor College of Medicine, Houston, TX USA; 420 University of Cologne, Cologne, Germany; 421 International Genomics Consortium, Phoenix, AZ USA; 422 Genomics Research Program, Ontario Institute for Cancer Research, Toronto, ON Canada; 423 Barking Havering and Redbridge University Hospitals NHS Trust, Romford, UK; 424 Children’s Hospital at Westmead, University of Sydney, Sydney, NSW Australia; 425 Department of Medicine, Section of Endocrinology, University and Hospital Trust of Verona, Verona, Italy; 426 Computational Biology Center, Memorial Sloan Kettering Cancer Center, New York, NY USA; 427 Department of Biology, ETH Zurich, Zürich, Switzerland; 428 Department of Computer Science, ETH Zurich, Zurich, Switzerland; 429 SIB Swiss Institute of Bioinformatics, Lausanne, Switzerland; 430 Weill Cornell Medical College, New York, NY USA; 431 Academic Department of Medical Genetics, University of Cambridge, Addenbrooke’s Hospital, Cambridge, UK; 432 MRC Cancer Unit, University of Cambridge, Cambridge, UK; 433 Departments of Pediatrics and Genetics, University of North Carolina at Chapel Hill, Chapel Hill, NC USA; 434 Seven Bridges Genomics, Charlestown, MA USA; 435 Annai Systems, Inc, Carlsbad, CA USA; 436 Department of Pathology, General Hospital of Treviso, Department of Medicine, University of Padua, Treviso, Italy; 437 Department of Computational Biology, University of Lausanne, Lausanne, Switzerland; 438 Department of Genetic Medicine and Development, University of Geneva Medical School, Geneva, CH Switzerland; 439 Swiss Institute of Bioinformatics, University of Geneva, Geneva, CH Switzerland; 440 The Francis Crick Institute, London, UK; 441 University of Leuven, Leuven, Belgium; 442 Institute of Medical Genetics and Applied Genomics, University of Tübingen, Tübingen, Germany; 443 Computational and Systems Biology, Genome Institute of Singapore, Singapore, Singapore; 444 School of Computing, National University of Singapore, Singapore, Singapore; 445 Big Data Institute, Li Ka Shing Centre, University of Oxford, Oxford, UK; 446 Biomedical Data Science Laboratory, Francis Crick Institute, London, UK; 447 Bioinformatics Group, Department of Computer Science, University College London, London, UK; 448 The Edward S. Rogers Sr. Department of Electrical and Computer Engineering, University of Toronto, Toronto, ON Canada; 449 Breast Cancer Translational Research Laboratory JC Heuson, Institut Jules Bordet, Brussels, Belgium; 450 Department of Oncology, Laboratory for Translational Breast Cancer Research, KU Leuven, Leuven, Belgium; 451 Institute for Research in Biomedicine (IRB Barcelona), The Barcelona Institute of Science and Technology, Barcelona, Spain; 452 Research Program on Biomedical Informatics, Universitat Pompeu Fabra, Barcelona, Spain; 453 Division of Medical Oncology, Princess Margaret Cancer Centre, Toronto, ON Canada; 454 Department of Physiology and Biophysics, Weill Cornell Medicine, New York, NY USA; 455 Institute for Computational Biomedicine, Weill Cornell Medicine, New York, NY USA; 456 Department of Pathology, UPMC Shadyside, Pittsburgh, PA USA; 457 Independent Consultant, Wellesley, USA; 458 Department of Cell and Molecular Biology, Science for Life Laboratory, Uppsala University, Uppsala, Sweden; 459 Department of Medicine and Department of Genetics, Washington University School of Medicine, St. Louis, St. Louis, MO USA; 460 Hefei University of Technology, Anhui, China; 461 Translational Cancer Research Unit, GZA Hospitals St.-Augustinus, Center for Oncological Research, Faculty of Medicine and Health Sciences, University of Antwerp, Antwerp, Belgium; 462 Simon Fraser University, Burnaby, BC Canada; 463 University of Pennsylvania, Philadelphia, PA USA; 464 Faculty of Science and Technology, University of Vic—Central University of Catalonia (UVic-UCC), Vic, Spain; 465 The Wellcome Trust, London, UK; 466 The Hospital for Sick Children, Toronto, ON Canada; 467 Department of Pathology, Queen Elizabeth University Hospital, Glasgow, UK; 468 Department of Genetics and Computational Biology, QIMR Berghofer Medical Research Institute, Brisbane, QLD Australia; 469 Department of Oncology, Centre for Cancer Genetic Epidemiology, University of Cambridge, Cambridge, UK; 470 Department of Public Health and Primary Care, Centre for Cancer Genetic Epidemiology, University of Cambridge, Cambridge, UK; 471 Prostate Cancer Canada, Toronto, ON Canada; 472 University of Cambridge, Cambridge, UK; 473 Department of Laboratory Medicine, Translational Cancer Research, Lund University Cancer Center at Medicon Village, Lund University, Lund, Sweden; 474 Heidelberg University, Heidelberg, Germany; 475 New BIH Digital Health Center, Berlin Institute of Health (BIH) and Charité - Universitätsmedizin Berlin, Berlin, Germany; 476 CIBER Epidemiología y Salud Pública (CIBERESP), Madrid, Spain; 477 Research Group on Statistics, Econometrics and Health (GRECS), UdG, Barcelona, Spain; 478 Quantitative Genomics Laboratories (qGenomics), Barcelona, Spain; 479 Icelandic Cancer Registry, Icelandic Cancer Society, Reykjavik, Iceland; 480 State Key Laboratory of Cancer Biology, and Xijing Hospital of Digestive Diseases, Fourth Military Medical University, Shaanxi, China; 481 Department of Medicine (DIMED), Surgical Pathology Unit, University of Padua, Padua, Italy; 482 Rigshospitalet, Copenhagen, Denmark; 483 Center for Cancer Genomics, National Cancer Institute, National Institutes of Health, Bethesda, MD USA; 484 Department of Biochemistry and Molecular Medicine, University of Montreal, Montreal, QC Canada; 485 Australian Institute of Tropical Health and Medicine, James Cook University, Douglas, QLD Australia; 486 Department of Neuro-Oncology, Istituto Neurologico Besta, Milano, Italy; 487 Bioplatforms Australia, North Ryde, NSW Australia; 488 Department of Pathology (Research), University College London Cancer Institute, London, UK; 489 Department of Surgical Oncology, Princess Margaret Cancer Centre, Toronto, ON Canada; 490 Department of Medical Oncology, Josephine Nefkens Institute and Cancer Genomics Centre, Erasmus Medical Center, Rotterdam, CN The Netherlands; 491 The University of Queensland Thoracic Research Centre, The Prince Charles Hospital, Brisbane, QLD Australia; 492 CIBIO/InBIO - Research Center in Biodiversity and Genetic Resources, Universidade do Porto, Vairão, Portugal; 493 HCA Laboratories, London, UK; 494 University of Liverpool, Liverpool, UK; 495 The Azrieli Faculty of Medicine, Bar-Ilan University, Safed, Israel; 496 Department of Neurosurgery, University of Florida, Gainesville, FL USA; 497 Department of Pathology, Graduate School of Medicine, University of Tokyo, Tokyo, Japan; 498 University of Milano Bicocca, Monza, Italy; 499 BGI-Shenzhen, Shenzhen, China; 500 Department of Pathology, Oslo University Hospital Ulleval, Oslo, Norway; 501 Center for Biomedical Informatics, Harvard Medical School, Boston, MA USA; 502 Department Biochemistry and Molecular Biomedicine, University of Barcelona, Barcelona, Spain; 503 Office of Cancer Genomics, National Cancer Institute, National Institutes of Health, Bethesda, MD USA; 504 Cancer Epigenomics, German Cancer Research Center (DKFZ), Heidelberg, Germany; 505 Department of Cancer Biology, The University of Texas MD Anderson Cancer Center, Houston, TX USA; 506 Department of Surgical Oncology, The University of Texas MD Anderson Cancer Center, Houston, TX USA; 507 Department of Computer Science, Yale University, New Haven, CT USA; 508 Department of Molecular Biophysics and Biochemistry, Yale University, New Haven, CT USA; 509 Program in Computational Biology and Bioinformatics, Yale University, New Haven, CT USA; 510 Center for Cancer Research, Massachusetts General Hospital, Boston, MA USA; 511 Department of Pathology, Massachusetts General Hospital, Boston, MA USA; 512 Department of Pathology, Memorial Sloan Kettering Cancer Center, New York, NY USA; 513 Division of Gastroenterology and Hepatology, Mayo Clinic, Rochester, MN USA; 514 University of Sydney, Sydney, NSW Australia; 515 University of Oxford, Oxford, UK; 516 Department of Surgery, Academic Urology Group, University of Cambridge, Cambridge, UK; 517 Department of Medicine II, University of Würzburg, Wuerzburg, Germany; 518 Sylvester Comprehensive Cancer Center, University of Miami, Miami, FL USA; 519 Institut Hospital del Mar d’Investigacions Mèdiques (IMIM), Barcelona, Spain; 520 Genome Integrity and Structural Biology Laboratory, National Institute of Environmental Health Sciences (NIEHS), Durham, NC USA; 521 St. Thomas’s Hospital, London, UK; 522 Osaka International Cancer Center, Osaka, Japan; 523 Department of Pathology, Skåne University Hospital, Lund University, Lund, Sweden; 524 Department of Medical Oncology, Beatson West of Scotland Cancer Centre, Glasgow, UK; 525 National Human Genome Research Institute, National Institutes of Health, Bethesda, MD USA; 526 Centre for Cancer Research, Victorian Comprehensive Cancer Centre, University of Melbourne, Melbourne, VIC Australia; 527 Department of Medicine, Section of Hematology/Oncology, University of Chicago, Chicago, IL USA; 528 German Center for Infection Research (DZIF), Partner Site Hamburg-Borstel-Lübeck-Riems, Hamburg, Germany; 529 Bioinformatics Research Centre (BiRC), Aarhus University, Aarhus, Denmark; 530 Department of Biotechnology, Ministry of Science and Technology, Government of India, New Delhi, Delhi India; 531 National Cancer Centre Singapore, Singapore, Singapore; 532 Brandeis University, Waltham, MA USA; 533 Department of Urologic Sciences, University of British Columbia, Vancouver, BC Canada; 534 Department of Internal Medicine, Stanford University, Stanford, CA USA; 535 The University of Texas Health Science Center at Houston, Houston, TX USA; 536 Imperial College NHS Trust, Imperial College, London, INY UK; 537 Senckenberg Institute of Pathology, University of Frankfurt Medical School, Frankfurt, Germany; 538 Department of Medicine, Division of Biomedical Informatics, UC San Diego School of Medicine, San Diego, CA USA; 539 Center for Precision Health, School of Biomedical Informatics, The University of Texas Health Science Center, Houston, TX USA; 540 Oxford Nanopore Technologies, New York, NY USA; 541 Institute of Medical Science, University of Tokyo, Tokyo, Japan; 542 Howard Hughes Medical Institute, University of California Santa Cruz, Santa Cruz, CA USA; 543 Wakayama Medical University, Wakayama, Japan; 544 Department of Internal Medicine, Division of Medical Oncology, Lineberger Comprehensive Cancer Center, University of North Carolina at Chapel Hill, Chapel Hill, NC USA; 545 University of Tennessee Health Science Center for Cancer Research, Memphis, TN USA; 546 Department of Histopathology, Salford Royal NHS Foundation Trust, Salford, UK; 547 Faculty of Biology, Medicine and Health, University of Manchester, Manchester, UK; 548 BIOPIC, ICG and College of Life Sciences, Peking University, Beijing, China; 549 Peking-Tsinghua Center for Life Sciences, Peking University, Beijing, China; 550 Children’s Hospital of Philadelphia, Philadelphia, PA USA; 551 Department of Bioinformatics and Computational Biology and Department of Systems Biology, The University of Texas MD Anderson Cancer Center, Houston, TX USA; 552 Karolinska Institute, Stockholm, Sweden; 553 The Donnelly Centre, University of Toronto, Toronto, ON Canada; 554 Department of Medical Genetics, College of Medicine, Hallym University, Chuncheon, South Korea; 555 Department of Experimental and Health Sciences, Institute of Evolutionary Biology (UPF-CSIC), Universitat Pompeu Fabra, Barcelona, Spain; 556 Health Data Science Unit, University Clinics, Heidelberg, Germany; 557 Massachusetts General Hospital Center for Cancer Research, Charlestown, MA USA; 558 Hokkaido University, Sapporo, Japan; 559 Department of Pathology and Clinical Laboratory, National Cancer Center Hospital, Tokyo, Japan; 560 Department of Genetics, University of North Carolina at Chapel Hill, Chapel Hill, NC USA; 561 Computational Biology, Leibniz Institute on Aging - Fritz Lipmann Institute (FLI), Jena, Germany; 562 University of Melbourne Centre for Cancer Research, Melbourne, VIC Australia; 563 University of Nebraska Medical Center, Omaha, NE USA; 564 Syntekabio Inc, Daejeon, South Korea; 565 Department of Pathology, Academic Medical Center, Amsterdam, AZ The Netherlands; 566 China National GeneBank-Shenzhen, Shenzhen, China; 567 Division of Molecular Genetics, German Cancer Research Center (DKFZ), Heidelberg, Germany; 568 Division of Life Science and Applied Genomics Center, Hong Kong University of Science and Technology, Clear Water Bay, Hong Kong, China; 569 Icahn School of Medicine at Mount Sinai, New York, NY USA; 570 Geneplus-Shenzhen, Shenzhen, China; 571 School of Computer Science and Technology, Xi’an Jiaotong University, Xi’an, China; 572 AbbVie, North Chicago, IL USA; 573 Institute of Pathology, Charité – University Medicine Berlin, Berlin, Germany; 574 Centre for Translational and Applied Genomics, British Columbia Cancer Agency, Vancouver, BC Canada; 575 Edinburgh Royal Infirmary, Edinburgh, UK; 576 Berlin Institute for Medical Systems Biology, Max Delbrück Center for Molecular Medicine, Berlin, Germany; 577 Department of Pediatric Immunology, Hematology and Oncology, University Hospital, Heidelberg, Germany; 578 German Cancer Research Center (DKFZ), Heidelberg, Germany; 579 Heidelberg Institute for Stem Cell Technology and Experimental Medicine (HI-STEM), Heidelberg, Germany; 580 Institute for Computational Biomedicine, Weill Cornell Medical College, New York, NY USA; 581 New York Genome Center, New York, NY USA; 582 Department of Urology, James Buchanan Brady Urological Institute, Johns Hopkins University School of Medicine, Baltimore, MD USA; 583 Department of Preventive Medicine, Graduate School of Medicine, The University of Tokyo, Tokyo, Japan; 584 Department of Molecular and Cellular Biology, Baylor College of Medicine, Houston, TX USA; 585 Department of Pathology and Immunology, Baylor College of Medicine, Houston, TX USA; 586 Michael E. DeBakey Veterans Affairs Medical Center, Houston, TX USA; 587 Technical University of Denmark, Lyngby, Denmark; 588 Department of Pathology, College of Medicine, Hanyang University, Seoul, South Korea; 589 Academic Unit of Surgery, School of Medicine, College of Medical, Veterinary and Life Sciences, University of Glasgow, Glasgow Royal Infirmary, Glasgow, UK; 590 Department of Pathology, Asan Medical Center, College of Medicine, Ulsan University, Songpa-gu, Seoul South Korea; 591 Science Writer, Garrett Park, MD USA; 592 International Cancer Genome Consortium (ICGC)/ICGC Accelerating Research in Genomic Oncology (ARGO) Secretariat, Ontario Institute for Cancer Research, Toronto, ON Canada; 593 University of Ljubljana, Ljubljana, Slovenia; 594 Department of Public Health Sciences, University of Chicago, Chicago, IL USA; 595 Research Institute, NorthShore University HealthSystem, Evanston, IL USA; 596 Department for Biomedical Research, University of Bern, Bern, Switzerland; 597 Centre of Genomics and Policy, McGill University and Génome Québec Innovation Centre, Montreal, QC Canada; 598 Carolina Center for Genome Sciences, University of North Carolina at Chapel Hill, Chapel Hill, NC USA; 599 Hopp Children’s Cancer Center (KiTZ), Heidelberg, Germany; 600 Pediatric Glioma Research Group, German Cancer Research Center (DKFZ), Heidelberg, Germany; 601 Cancer Research UK, London, UK; 602 Indivumed GmbH, Hamburg, Germany; 603 Genome Integration Data Center, Syntekabio, Inc, Daejeon, South Korea; 604 University Hospital Zurich, Zurich, Switzerland; 605 Clinical Bioinformatics, Swiss Institute of Bioinformatics, Geneva, Switzerland; 606 Institute for Pathology and Molecular Pathology, University Hospital Zurich, Zurich, Switzerland; 607 Institute of Molecular Life Sciences, University of Zurich, Zurich, Switzerland; 608 MRC Human Genetics Unit, MRC IGMM, University of Edinburgh, Edinburgh, UK; 609 Women’s Cancer Program at the Samuel Oschin Comprehensive Cancer Institute, Cedars-Sinai Medical Center, Los Angeles, CA USA; 610 Department of Biology, Bioinformatics Group, Division of Molecular Biology, Faculty of Science, University of Zagreb, Zagreb, Croatia; 611 Department for Internal Medicine II, University Hospital Schleswig-Holstein, Kiel, Germany; 612 Genetics and Molecular Pathology, SA Pathology, Adelaide, SA Australia; 613 Department of Gastric Surgery, National Cancer Center Hospital, Tokyo, Japan; 614 Department of Bioinformatics, Division of Cancer Genomics, National Cancer Center Research Institute, Tokyo, Japan; 615 A.A. Kharkevich Institute of Information Transmission Problems, Moscow, Russia; 616 Oncology and Immunology, Dmitry Rogachev National Research Center of Pediatric Hematology, Moscow, Russia; 617 Skolkovo Institute of Science and Technology, Moscow, Russia; 618 Department of Surgery, The George Washington University, School of Medicine and Health Science, Washington, DC USA; 619 Endocrine Oncology Branch, Center for Cancer Research, National Cancer Institute, National Institutes of Health, Bethesda, MD USA; 620 Melanoma Institute Australia, Macquarie University, Sydney, NSW Australia; 621 MIT Computer Science and Artificial Intelligence Laboratory, Massachusetts Institute of Technology, Cambridge, MA USA; 622 Tissue Pathology and Diagnostic Oncology, Royal Prince Alfred Hospital, Sydney, NSW Australia; 623 Cholangiocarcinoma Screening and Care Program and Liver Fluke and Cholangiocarcinoma Research Centre, Faculty of Medicine, Khon Kaen University, Khon Kaen, Thailand; 624 Controlled Department and Institution, New York, NY USA; 625 Englander Institute for Precision Medicine, Weill Cornell Medicine, New York, NY USA; 626 National Cancer Center, Gyeonggi, South Korea; 627 Department of Biochemistry, College of Medicine, Ewha Womans University, Seoul, South Korea; 628 Health Sciences Department of Biomedical Informatics, University of California San Diego, La Jolla, CA USA; 629 Research Core Center, National Cancer Centre Korea, Goyang-si, South Korea; 630 Department of Health Sciences and Technology, Sungkyunkwan University School of Medicine, Seoul, South Korea; 631 Samsung Genome Institute, Seoul, South Korea; 632 Breast Oncology Program, Dana-Farber/Brigham and Women’s Cancer Center, Boston, MA USA; 633 Department of Surgery, Memorial Sloan Kettering Cancer Center, New York, NY USA; 634 Division of Breast Surgery, Brigham and Women’s Hospital, Boston, MA USA; 635 Integrative Bioinformatics Support Group, National Institute of Environmental Health Sciences (NIEHS), Durham, NC USA; 636 Department of Clinical Science, University of Bergen, Bergen, Norway; 637 Center For Medical Innovation, Seoul National University Hospital, Seoul, South Korea; 638 Department of Internal Medicine, Seoul National University Hospital, Seoul, South Korea; 639 Institute of Computer Science, Polish Academy of Sciences, Warsawa, Poland; 640 Functional and Structural Genomics, German Cancer Research Center (DKFZ), Heidelberg, Germany; 641 Laboratory of Translational Genomics, Division of Cancer Epidemiology and Genetics, National Cancer Institute, , National Institutes of Health, Bethesda, MD USA; 642 Institute for Medical Informatics Statistics and Epidemiology, University of Leipzig, Leipzig, Germany; 643 Morgan Welch Inflammatory Breast Cancer Research Program and Clinic, The University of Texas MD Anderson Cancer Center, Houston, TX USA; 644 Department of Hematology and Oncology, Georg-Augusts-University of Göttingen, Göttingen, Germany; 645 Institute of Cell Biology (Cancer Research), University of Duisburg-Essen, Essen, Germany; 646 King’s College London and Guy’s and St. Thomas’ NHS Foundation Trust, London, UK; 647 Center for Epigenetics, Van Andel Research Institute, Grand Rapids, MI USA; 648 The University of Queensland Centre for Clinical Research, Royal Brisbane and Women’s Hospital, Herston, QLD Australia; 649 Department of Pediatric Oncology and Hematology, University of Cologne, Cologne, Germany; 650 University of Düsseldorf, Düsseldorf, Germany; 651 Department of Pathology, Institut Jules Bordet, Brussels, Belgium; 652 Institute of Biomedicine, Sahlgrenska Academy at University of Gothenburg, Gothenburg, Sweden; 653 Children’s Medical Research Institute, Sydney, NSW Australia; 654 ILSbio, LLC Biobank, Chestertown, MD USA; 655 Division of Genetics and Genomics, Boston Children’s Hospital, Harvard Medical School, Boston, MA USA; 656 Institute for Bioengineering and Biopharmaceutical Research (IBBR), Hanyang University, Seoul, South Korea; 657 Department of Statistics, University of California Santa Cruz, Santa Cruz, CA USA; 658 National Genotyping Center, Institute of Biomedical Sciences, Academia Sinica, Taipei, Taiwan; 659 Department of Vertebrate Genomics/Otto Warburg Laboratory Gene Regulation and Systems Biology of Cancer, Max Planck Institute for Molecular Genetics, Berlin, Germany; 660 McGill University and Genome Quebec Innovation Centre, Montreal, QC Canada; 661 biobyte solutions GmbH, Heidelberg, Germany; 662 Gynecologic Oncology, NYU Laura and Isaac Perlmutter Cancer Center, New York University, New York, NY USA; 663 Division of Oncology, Stem Cell Biology Section, Washington University School of Medicine, St. Louis, MO USA; 664 Department of Systems Biology, The University of Texas MD Anderson Cancer Center, Houston, TX USA; 665 Harvard University, Cambridge, MA USA; 666 Urologic Oncology Branch, Center for Cancer Research, National Cancer Institute, National Institutes of Health, Bethesda, MD USA; 667 University of Oslo, Oslo, Norway; 668 University of Toronto, Toronto, ON Canada; 669 Peking University, Beijing, China; 670 School of Life Sciences, Peking University, Beijing, China; 671 Leidos Biomedical Research, Inc, McLean, VA USA; 672 Hematology, Hospital Clinic, Institut d’Investigacions Biomèdiques August Pi i Sunyer (IDIBAPS), University of Barcelona, Barcelona, Spain; 673 Second Military Medical University, Shanghai, China; 674 Chinese Cancer Genome Consortium, Shenzhen, China; 675 Department of Medical Oncology, Beijing Hospital, Beijing, China; 676 Laboratory of Molecular Oncology, Key Laboratory of Carcinogenesis and Translational Research (Ministry of Education), Peking University Cancer Hospital and Institute, Beijing, China; 677 School of Medicine/School of Mathematics and Statistics, University of St. Andrews, St, Andrews, Fife UK; 678 Institute for Systems Biology, Seattle, WA USA; 679 Department of Biochemistry and Molecular Biology, Faculty of Medicine, University Institute of Oncology-IUOPA, Oviedo, Spain; 680 Institut Bergonié, Bordeaux, France; 681 Cancer Unit, MRC University of Cambridge, Cambridge, UK; 682 Department of Pathology and Laboratory Medicine, Center for Personalized Medicine, Children’s Hospital Los Angeles, Los Angeles, CA USA; 683 John Curtin School of Medical Research, Canberra, ACT Australia; 684 MVZ Department of Oncology, PraxisClinic am Johannisplatz, Leipzig, Germany; 685 Department of Information Technology, Ghent University, Ghent, Belgium; 686 Department of Plant Biotechnology and Bioinformatics, Ghent University, Ghent, Belgium; 687 Institute for Genomic Medicine, Nationwide Children’s Hospital, Columbus, OH USA; 688 Computational Biology Program, School of Medicine, Oregon Health and Science University, Portland, OR USA; 689 Department of Surgery, Duke University, Durham, NC USA; 690 Institució Catalana de Recerca i Estudis Avançats (ICREA), Barcelona, Spain; 691 Institut Català de Paleontologia Miquel Crusafont, Universitat Autònoma de Barcelona, Barcelona, Spain; 692 University of Glasgow, Glasgow, UK; 693 Institut d’Investigacions Biomèdiques August Pi i Sunyer (IDIBAPS), Barcelona, Spain; 694 Division of Oncology, Washington University School of Medicine, St. Louis, MO USA; 695 Department of Surgery and Cancer, Imperial College, London, INY UK; 696 Applications Department, Oxford Nanopore Technologies, Oxford, UK; 697 Department of Obstetrics, Gynecology and Reproductive Services, University of California San Francisco, San Francisco, CA USA; 698 Department of Biochemistry and Molecular Medicine, University California at Davis, Sacramento, CA USA; 699 STTARR Innovation Facility, Princess Margaret Cancer Centre, Toronto, ON Canada; 700 Discipline of Surgery, Western Sydney University, Penrith, NSW Australia; 701 Yale School of Medicine, Yale University, New Haven, CT USA; 702 Department of Genetics, Lineberger Comprehensive Cancer Center, University of North Carolina at Chapel Hill, Chapel Hill, NC USA; 703 Departments of Neurology and Neurosurgery, Henry Ford Hospital, Detroit, MI USA; 704 Precision Oncology, OHSU Knight Cancer Institute, Oregon Health and Science University, Portland, OR USA; 705 Institute of Pathology, University Medical Center Hamburg-Eppendorf, Hamburg, Germany; 706 Department of Health Sciences, Faculty of Medical Sciences, Kyushu University, Fukuoka, Japan; 707 Heidelberg Academy of Sciences and Humanities, Heidelberg, Germany; 708 Department of Clinical Pathology, University of Melbourne, Melbourne, VIC, Australia; 709 Department of Pathology, Roswell Park Cancer Institute, Buffalo, NY USA; 710 Department of Computer Science, University of Helsinki, Helsinki, Finland; 711 Institute of Biotechnology, University of Helsinki, Helsinki, Finland; 712 Organismal and Evolutionary Biology Research Programme, University of Helsinki, Helsinki, Finland; 713 Department of Obstetrics and Gynecology, Division of Gynecologic Oncology, Washington University School of Medicine, St. Louis, MO USA; 714 Penrose St. Francis Health Services, Colorado Springs, CO USA; 715 Institute of Pathology, Ulm University and University Hospital of Ulm, Ulm, Germany; 716 National Cancer Center, Tokyo, Japan; 717 Genome Institute of Singapore, Singapore, Singapore; 718 32Program in Computational Biology and Bioinformatics, Yale University, New Haven, CT USA; 719 German Cancer Aid, Bonn, Germany; 720 Programme in Cancer and Stem Cell Biology, Centre for Computational Biology, Duke-NUS Medical School, Singapore, Singapore; 721 The Chinese University of Hong Kong, Shatin, NT, Hong Kong China; 722 Fourth Military Medical University, Shaanxi, China; 723 The University of Cambridge School of Clinical Medicine, Cambridge, UK; 724 St. Jude Children’s Research Hospital, Memphis, TN USA; 725 University Health Network, Princess Margaret Cancer Centre, Toronto, ON Canada; 726 Center for Biomolecular Science and Engineering, University of California Santa Cruz, Santa Cruz, CA USA; 727 Department of Medicine, University of Chicago, Chicago, IL USA; 728 Department of Neurology, Mayo Clinic, Rochester, MN USA; 729 Cambridge Oesophagogastric Centre, Cambridge University Hospitals NHS Foundation Trust, Cambridge, UK; 730 Department of Computer Science, Carleton College, Northfield, MN USA; 731 Institute of Cancer Sciences, College of Medical Veterinary and Life Sciences, University of Glasgow, Glasgow, UK; 732 Department of Epidemiology, University of Alabama at Birmingham, Birmingham, AL USA; 733 HudsonAlpha Institute for Biotechnology, Huntsville, AL USA; 734 O’Neal Comprehensive Cancer Center, University of Alabama at Birmingham, Birmingham, AL USA; 735 Department of Pathology, Keio University School of Medicine, Tokyo, Japan; 736 Department of Hepatobiliary and Pancreatic Oncology, National Cancer Center Hospital, Tokyo, Japan; 737 Sage Bionetworks, Seattle, WA USA; 738 Lymphoma Genomic Translational Research Laboratory, National Cancer Centre, Singapore, Singapore; 739 Department of Clinical Pathology, Robert-Bosch-Hospital, Stuttgart, Germany; 740 Department of Cell and Systems Biology, University of Toronto, Toronto, ON Canada; 741 Department of Biosciences and Nutrition, Karolinska Institutet, Stockholm, Sweden; 742 Center for Liver Cancer, Research Institute and Hospital, National Cancer Center, Gyeonggi, South Korea; 743 Division of Hematology-Oncology, Samsung Medical Center, Sungkyunkwan University School of Medicine, Seoul, South Korea; 744 Samsung Advanced Institute for Health Sciences and Technology, Sungkyunkwan University School of Medicine, Seoul, South Korea; 745 Cheonan Industry-Academic Collaboration Foundation, Sangmyung University, Cheonan, South Korea; 746 NYU Langone Medical Center, New York, NY USA; 747 Department of Hematology and Medical Oncology, Cleveland Clinic, Cleveland, OH USA; 748 Department of Radiation Oncology, University of California San Francisco, San Francisco, CA USA; 749 Department of Health Sciences Research, Mayo Clinic, Rochester, MN USA; 750 Helen F. Graham Cancer Center at Christiana Care Health Systems, Newark, DE USA; 751 Heidelberg University Hospital, Heidelberg, Germany; 752 CSRA Incorporated, Fairfax, VA USA; 753 Research Department of Pathology, University College London Cancer Institute, London, UK; 754 Department of Research Oncology, Guy’s Hospital, King’s Health Partners AHSC, King’s College London School of Medicine, London, UK; 755 Faculty of Medicine and Health Sciences, Macquarie University, Sydney, NSW Australia; 756 University Hospital of Minjoz, INSERM UMR 1098, Besançon, France; 757 Spanish National Cancer Research Centre, Madrid, Spain; 758 Center of Digestive Diseases and Liver Transplantation, Fundeni Clinical Institute, Bucharest, Romania; 759 Cureline, Inc, South San Francisco, CA USA; 760 St. Luke’s Cancer Centre, Royal Surrey County Hospital NHS Foundation Trust, Guildford, UK; 761 Cambridge Breast Unit, Addenbrooke’s Hospital, Cambridge University Hospital NHS Foundation Trust and NIHR Cambridge Biomedical Research Centre, Cambridge, UK; 762 East of Scotland Breast Service, Ninewells Hospital, Aberdeen, UK; 763 Department of Genetics, Microbiology and Statistics, University of Barcelona, IRSJD, IBUB, Barcelona, Spain; 764 Department of Obstetrics and Gynecology, Medical College of Wisconsin, Milwaukee, WI USA; 765 Hematology and Medical Oncology, Winship Cancer Institute of Emory University, Atlanta, GA USA; 766 Department of Computer Science, Princeton University, Princeton, NJ USA; 767 Vanderbilt Ingram Cancer Center, Vanderbilt University, Nashville, TN USA; 768 Ohio State University College of Medicine and Arthur G. James Comprehensive Cancer Center, Columbus, OH USA; 769 Department of Surgery, Yokohama City University Graduate School of Medicine, Kanagawa, Japan; 770 Division of Chromatin Networks, German Cancer Research Center (DKFZ) and BioQuant, Heidelberg, Germany; 771 Research Computing Center, University of North Carolina at Chapel Hill, Chapel Hill, NC USA; 772 School of Molecular Biosciences and Center for Reproductive Biology, Washington State University, Pullman, WA USA; 773 Finsen Laboratory and Biotech Research and Innovation Centre (BRIC), University of Copenhagen, Copenhagen, Denmark; 774 Department of Laboratory Medicine and Pathobiology, University of Toronto, Toronto, ON Canada; 775 Department of Pathology, Human Oncology and Pathogenesis Program, Memorial Sloan Kettering Cancer Center, New York, NY USA; 776 University Hospital Giessen, Pediatric Hematology and Oncology, Giessen, Germany; 777 Oncologie Sénologie, ICM Institut Régional du Cancer, Montpellier, France; 778 Institute of Clinical Molecular Biology, Christian-Albrechts-University, Kiel, Germany; 779 Institute of Pathology, University of Wuerzburg, Wuerzburg, Germany; 780 Department of Urology, North Bristol NHS Trust, Bristol, UK; 781 SingHealth, Duke-NUS Institute of Precision Medicine, National Heart Centre Singapore, Singapore, Singapore; 782 Department of Computer Science, University of Toronto, Toronto, ON Canada; 783 Bern Center for Precision Medicine, University Hospital of Bern, University of Bern, Bern, Switzerland; 784 Englander Institute for Precision Medicine, Weill Cornell Medicine and New York Presbyterian Hospital, New York, NY USA; 785 Meyer Cancer Center, Weill Cornell Medicine, New York, NY USA; 786 Pathology and Laboratory, Weill Cornell Medical College, New York, NY USA; 787 Vall d’Hebron Institute of Oncology: VHIO, Barcelona, Spain; 788 General and Hepatobiliary-Biliary Surgery, Pancreas Institute, University and Hospital Trust of Verona, Verona, Italy; 789 National Centre for Biological Sciences, Tata Institute of Fundamental Research, Bangalore, India; 790 Indiana University, Bloomington, IN USA; 791 Department of Pathology, GZA-ZNA Hospitals, Antwerp, Belgium; 792 Analytical Biological Services, Inc, Wilmington, DE USA; 793 Sydney Medical School, University of Sydney, Sydney, NSW Australia; 794 cBio Center, Dana-Farber Cancer Institute, Harvard Medical School, Boston, MA USA; 795 Department of Cell Biology, Harvard Medical School, Boston, MA USA; 796 Advanced Centre for Treatment Research and Education in Cancer, Tata Memorial Centre, Navi Mumbai, Maharashtra India; 797 School of Environmental and Life Sciences, Faculty of Science, The University of Newcastle, Ourimbah, NSW Australia; 798 Department of Dermatology, University Hospital of Essen, Essen, Germany; 799 Bioinformatics and Omics Data Analytics, German Cancer Research Center (DKFZ), Heidelberg, Germany; 800 Department of Urology, Charité Universitätsmedizin Berlin, Berlin, Germany; 801 Martini-Clinic, Prostate Cancer Center, University Medical Center Hamburg-Eppendorf, Hamburg, Germany; 802 Department of General Internal Medicine, University of Kiel, Kiel, Germany; 803 German Cancer Consortium (DKTK), Partner site Berlin, Berlin, Germany; 804 Cancer Research Institute, Beth Israel Deaconess Medical Center, Boston, MA USA; 805 University of Pittsburgh, Pittsburgh, PA USA; 806 Department of Ophthalmology and Ocular Genomics Institute, Massachusetts Eye and Ear, Harvard Medical School, Boston, MA USA; 807 Center for Psychiatric Genetics, NorthShore University HealthSystem, Evanston, IL USA; 808 Van Andel Research Institute, Grand Rapids, MI USA; 809 Laboratory of Molecular Medicine, Human Genome Center, Institute of Medical Science, University of Tokyo, Tokyo, Japan; 810 Japan Agency for Medical Research and Development, Tokyo, Japan; 811 Korea University, Seoul, South Korea; 812 Murtha Cancer Center, Walter Reed National Military Medical Center, Bethesda, MD USA; 813 Human Genetics, University of Kiel, Kiel, Germany; 814 Department of Oncologic Pathology, Dana-Farber Cancer Institute, Harvard Medical School, Boston, MA USA; 815 Oregon Health and Science University, Portland, OR USA; 816 Center for RNA Interference and Noncoding RNA, The University of Texas MD Anderson Cancer Center, Houston, TX USA; 817 Department of Experimental Therapeutics, The University of Texas MD Anderson Cancer Center, Houston, TX USA; 818 Department of Gynecologic Oncology and Reproductive Medicine, The University of Texas MD Anderson Cancer Center, Houston, TX USA; 819 University Hospitals Coventry and Warwickshire NHS Trust, Coventry, UK; 820 Department of Radiation Oncology, Radboud University Nijmegen Medical Centre, Nijmegen, GA The Netherlands; 821 Institute for Genomics and Systems Biology, University of Chicago, Chicago, IL USA; 822 Clinic for Hematology and Oncology, St.-Antonius-Hospital, Eschweiler, Germany; 823 Computational and Systems Biology Program, Memorial Sloan Kettering Cancer Center, New York, NY USA; 824 University of Iceland, Reykjavik, Iceland; 825 Division of Computational Genomics and Systems Genetics, German Cancer Research Center (DKFZ), Heidelberg, Germany; 826 Dundee Cancer Centre, Ninewells Hospital, Dundee, UK; 827 Department for Internal Medicine III, University of Ulm and University Hospital of Ulm, Ulm, Germany; 828 Institut Curie, INSERM Unit 830, Paris, France; 829 Department of Gastroenterology and Hepatology, Yokohama City University Graduate School of Medicine, Kanagawa, Japan; 830 Department of Laboratory Medicine, Radboud University Nijmegen Medical Centre, Nijmegen, GA The Netherlands; 831 Division of Cancer Genome Research, German Cancer Research Center (DKFZ), Heidelberg, Germany; 832 Department of General Surgery, Singapore General Hospital, Singapore, Singapore; 833 Cancer Science Institute of Singapore, National University of Singapore, Singapore, Singapore; 834 Department of Medical and Clinical Genetics, Genome-Scale Biology Research Program, University of Helsinki, Helsinki, Finland; 835 East Anglian Medical Genetics Service, Cambridge University Hospitals NHS Foundation Trust, Cambridge, UK; 836 Irving Institute for Cancer Dynamics, Columbia University, New York, NY USA; 837 Institute of Molecular and Cell Biology, Singapore, Singapore; 838 Laboratory of Cancer Epigenome, Division of Medical Science, National Cancer Centre Singapore, Singapore, Singapore; 839 Universite Lyon, INCa-Synergie, Centre Léon Bérard, Lyon, France; 840 Department of Urology, Mayo Clinic, Rochester, MN USA; 841 Royal National Orthopaedic Hospital - Stanmore, Stanmore, Middlesex UK; 842 Department of Biochemistry, Genetics and Immunology, University of Vigo, Vigo, Spain; 843 Giovanni Paolo II / I.R.C.C.S. Cancer Institute, Bari, BA Italy; 844 Neuroblastoma Genomics, German Cancer Research Center (DKFZ), Heidelberg, Germany; 845 Fondazione Policlinico Universitario Gemelli IRCCS, Rome, Italy, Rome, Italy; 846 University of Verona, Verona, Italy; 847 Centre National de Génotypage, CEA - Institute de Génomique, Evry, France; 848 CAPHRI Research School, Maastricht University, Maastricht, ER The Netherlands; 849 Department of Biopathology, Centre Léon Bérard, Lyon, France; 850 Université Claude Bernard Lyon 1, Villeurbanne, France; 851 Core Research for Evolutional Science and Technology (CREST), JST, Tokyo, Japan; 852 Department of Biological Sciences, Laboratory for Medical Science Mathematics, Graduate School of Science, University of Tokyo, Yokohama, Japan; 853 Department of Medical Science Mathematics, Medical Research Institute, Tokyo Medical and Dental University (TMDU), Tokyo, Japan; 854 Cancer Ageing and Somatic Mutation Programme, Wellcome Sanger Institute, Hinxton, UK; 855 University Hospitals Birmingham NHS Foundation Trust, Birmingham, UK; 856 Centre for Cancer Research and Cell Biology, Queen’s University, Belfast, UK; 857 Breast Medical Oncology, The University of Texas MD Anderson Cancer Center, Houston, TX USA; 858 Department of Surgery, Johns Hopkins University School of Medicine, Baltimore, MD USA; 859 Department of Oncology-Pathology, Science for Life Laboratory, Karolinska Institute, Stockholm, Sweden; 860 School of Cancer Sciences, Faculty of Medicine, University of Southampton, Southampton, UK; 861 Department of Gene Technology, Tallinn University of Technology, Tallinn, Estonia; 862 Genetics and Genome Biology Program, SickKids Research Institute, The Hospital for Sick Children, Toronto, ON Canada; 863 Departments of Neurosurgery and Hematology and Medical Oncology, Winship Cancer Institute and School of Medicine, Emory University, Atlanta, GA USA; 864 Department of Clinical and Molecular Medicine, Faculty of Medicine and Health Sciences, Norwegian University of Science and Technology, Trondheim, Norway; 865 Argmix Consulting, North Vancouver, BC Canada; 866 Department of Information Technology, Ghent University, Interuniversitair Micro-Electronica Centrum (IMEC), Ghent, Belgium; 867 Nuffield Department of Surgical Sciences, John Radcliffe Hospital, University of Oxford, Oxford, UK; 868 Institute of Mathematics and Computer Science, University of Latvia, Riga, LV Latvia; 869 Discipline of Pathology, Sydney Medical School, University of Sydney, Sydney, NSW Australia; 870 Department of Applied Mathematics and Theoretical Physics, Centre for Mathematical Sciences, University of Cambridge, Cambridge, UK; 871 Department of Epidemiology and Biostatistics, Memorial Sloan Kettering Cancer Center, New York, NY USA; 872 Department of Statistics, Columbia University, New York, NY USA; 873 Department of Immunology, Genetics and Pathology, Science for Life Laboratory, Uppsala University, Uppsala, Sweden; 874 School of Electronic and Information Engineering, Xi’an Jiaotong University, Xi’an, China; 875 Department of Histopathology, Cambridge University Hospitals NHS Foundation Trust, Cambridge, UK; 876 Oxford NIHR Biomedical Research Centre, University of Oxford, Oxford, UK; 877 Georgia Regents University Cancer Center, Augusta, GA USA; 878 Wythenshawe Hospital, Manchester, UK; 879 Department of Genetics, Washington University School of Medicine, St.Louis, MO USA; 880 Department of Biological Oceanography, Leibniz Institute of Baltic Sea Research, Rostock, Germany; 881 Wellcome Centre for Human Genetics, University of Oxford, Oxford, UK; 882 Department of Molecular and Human Genetics, Baylor College of Medicine, Houston, TX USA; 883 Thoracic Oncology Laboratory, Mayo Clinic, Rochester, MN USA; 884 Institute for Genomic Medicine, Nationwide Children’s Hospital, Columbus, OH USA; 885 Department of Obstetrics and Gynecology, Division of Gynecologic Oncology, Mayo Clinic, Rochester, MN USA; 886 International Institute for Molecular Oncology, Poznań, Poland; 887 Poznan University of Medical Sciences, Poznań, Poland; 888 Genomics and Proteomics Core Facility High Throughput Sequencing Unit, German Cancer Research Center (DKFZ), Heidelberg, Germany; 889 NCCS-VARI Translational Research Laboratory, National Cancer Centre Singapore, Singapore, Singapore; 890 Edison Family Center for Genome Sciences and Systems Biology, Washington University, St. Louis, MO USA; 891 MRC-University of Glasgow Centre for Virus Research, Glasgow, UK; 892 Department of Medical Informatics and Clinical Epidemiology, Division of Bioinformatics and Computational Biology, OHSU Knight Cancer Institute, Oregon Health and Science University, Portland, OR USA; 893 School of Electronic Information and Communications, Huazhong University of Science and Technology, Wuhan, China; 894 Department of Applied Mathematics and Statistics, Johns Hopkins University, Baltimore, MD USA; 895 Department of Cancer Genome Informatics, Graduate School of Medicine, Osaka University, Osaka, Japan; 896 Institute of Computer Science, Heidelberg University, Heidelberg, Germany; 897 School of Mathematics and Statistics, University of Sydney, Sydney, NSW Australia; 898 Ben May Department for Cancer Research, University of Chicago, Chicago, IL USA; 899 Department of Human Genetics, University of Chicago, Chicago, IL USA; 900 Tri-Institutional PhD Program in Computational Biology and Medicine, Weill Cornell Medicine, New York, NY USA; 901 The First Affiliated Hospital, Xi’an Jiaotong University, Xi’an, China; 902 Department of Medicine and Therapeutics, The Chinese University of Hong Kong, Shatin, NT, Hong Kong China; 903 Department of Biostatistics, The University of Texas MD Anderson Cancer Center, Houston, TX USA; 904 Duke-NUS Medical School, Singapore, Singapore; 905 Department of Surgery, Ruijin Hospital, Shanghai Jiaotong University School of Medicine, Shanghai, China; 906 School of Computing Science, University of Glasgow, Glasgow, UK; 907 Division of Orthopaedic Surgery, Oslo University Hospital, Oslo, Norway; 908 Eastern Clinical School, Monash University, Melbourne, VIC Australia; 909 Epworth HealthCare, Richmond, VIC Australia; 910 Department of Biostatistics and Computational Biology, Dana-Farber Cancer Institute and Harvard Medical School, Boston, MA USA; 911 Department of Biomedical Informatics, College of Medicine, The Ohio State University, Columbus, OH USA; 912 The Ohio State University Comprehensive Cancer Center (OSUCCC – James), Columbus, OH USA; 913 The University of Texas School of Biomedical Informatics (SBMI) at Houston, Houston, TX USA; 914 Department of Biostatistics, University of North Carolina at Chapel Hill, Chapel Hill, NC USA; 915 Department of Biochemistry and Molecular Genetics, Feinberg School of Medicine, Northwestern University, Chicago, IL USA; 916 Faculty of Medicine and Health, University of Sydney, Sydney, NSW Australia; 917 Department of Pathology, Erasmus Medical Center Rotterdam, Rotterdam, GD The Netherlands; 918 Division of Molecular Carcinogenesis, The Netherlands Cancer Institute, Amsterdam, CX The Netherlands; 919 Institute of Molecular Life Sciences and Swiss Institute of Bioinformatics, University of Zurich, Zurich, Switzerland

**Keywords:** Cancer, Computational biology and bioinformatics, Machine learning

## Abstract

We present SVclone, a computational method for inferring the cancer cell fraction of structural variant (SV) breakpoints from whole-genome sequencing data. SVclone accurately determines the variant allele frequencies of both SV breakends, then simultaneously estimates the cancer cell fraction and SV copy number. We assess performance using in silico mixtures of real samples, at known proportions, created from two clonal metastases from the same patient. We find that SVclone’s performance is comparable to single-nucleotide variant-based methods, despite having an order of magnitude fewer data points. As part of the Pan-Cancer Analysis of Whole Genomes (PCAWG) consortium, which aggregated whole-genome sequencing data from 2658 cancers across 38 tumour types, we use SVclone to reveal a subset of liver, ovarian and pancreatic cancers with subclonally enriched copy-number neutral rearrangements that show decreased overall survival. SVclone enables improved characterisation of SV intra-tumour heterogeneity.

## Abstract (web summary)

The authors present SVclone, a computational method for inferring the cancer cell fraction of structural variants from whole-genome sequencing data.

## Introduction

The clonal theory of cancer evolution^1^ posits that cancers arise from a single progenitor cell that has acquired mutations conferring selective advantage, resulting in the expansion of a genetically identical cell population or clone. As a cancer grows, a process akin to Darwinian species evolution emerges with subsequent genetically distinct populations arising from the founding clone via the continual acquisition of advantageous genomic aberrations. Consequently, tumours are likely to consist of a genetically heterogeneous combination of multiple cell populations, the extent of which has been revealed through the use of whole-genome sequencing^2,3^. As clones can respond differently to therapy^4^, understanding this cellular diversity has important clinical implications^5^.

The mutations belonging to each clone in a tumour can be interrogated using bulk whole-genome sequencing, with mutation detection subject to factors such as sequencing depth and quality, tumour cellularity and mutation copy number^6^. The expansion of each clone over the life of a tumour is encoded in the allele frequency of somatic mutations^7^. To characterise the clonal composition of a tumour, the variant allele frequency (VAF) must be converted to a cancer cell fraction (CCF), the fraction of cancer cells within which the variant is present. Events appearing in all cancer cells (CCF = 100%) are considered clonal and due to a pervasive expansion. Events appearing in a subset of cells (CCF < 100%) are considered subclonal and part of an ongoing expansion. Estimating the cancer cell fraction of events is challenging, as the observed variant allele frequency depends on the amount of normal cell admixture (purity) and local copy number.

Given these challenges, previous computational approaches for estimating CCF have focused on individual facets of this complexity, commonly limiting their view to single-nucleotide variants (SNVs)^8–13^ or somatic copy-number aberrations (SCNAs)^14–16^. This has left the clonality of balanced rearrangements largely unexplored, despite their implication in oncogenic fusions^17^ and subclonal translocations conferring drug-resistant phenotypes^18^. While SNV-based approaches have provided solutions to the problem of downstream inference of mutation CCF, they cannot be used for structural variant (SV) breakpoint data as: (i) no complete and robust methodology exists yet to calculate VAFs from SVs (Fan et al.^19^ provides a limited framework that does not correct for DNA-gains or support all SV types), (ii) SVs themselves can cause copy-number changes (background copy numbers must therefore be inferred differently), (iii) SVs are composed of two ends, each with a potentially different VAF, and (iv) due to the relatively small number of data points (on average compared with SNVs), false-positive SVs greatly diminish clustering performance, hence a robust filtering methodology is required to consider only high-confidence SVs.

To address this gap, we present SVclone, an algorithmic approach that infers CCFs of SV breakpoints. It considers all types of large-scale structural variation (SV), including copy-number aberrant and copy-number neutral variation. The Pan-Cancer Analysis of Whole Genomes (PCAWG) Consortium has aggregated whole-genome sequencing data from 2658 cancers across 38 tumour types generated by the ICGC and TCGA projects. These sequencing data were re-analysed with standardised, high-accuracy pipelines to align to the human genome (reference build hs37d5) and identify germline variants and somatically acquired mutations, as described in^20^. Here we apply SVclone to these large-scale data to generate insight into patterns of clonality of structural variation across a large number of cancer types, and identify functionally important and clinically relevant observations.

## Results

### Algorithm overview

**Fig. 1:**
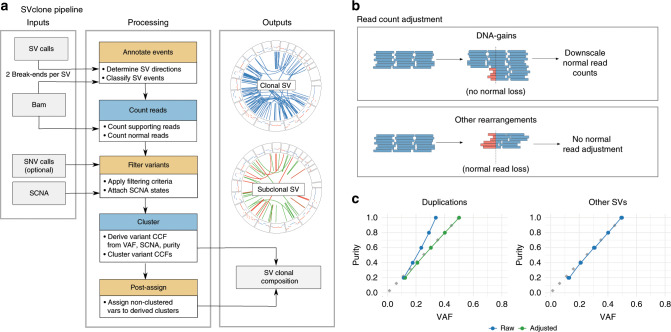
Pipeline schematic and VAF calculation adjustment. **a** A flow-chart of the SVclone pipeline. **b** A schematic showing the adjustments required for DNA-gains (top) and all other rearrangements (bottom). From left to right, each segment shows an unaffected locus, the effect of the variant type on reads at the breakpoint, and the resulting adjustment strategy required to normalise the allele frequency. Red portions of the reads show soft-clips, i.e. the portion of the reads mapping to the other end of the breakpoint. **c** The effect of adjusting raw VAFs in duplications (left), and unadjusted VAFs for other SVs (right), at purity levels at 20–100% in 20% increments, where the expected VAF is half the purity level (dotted line).

The SVclone algorithm consists of five steps: annotate, count, filter, cluster and post-assign. A graphical representation of the SVclone pipeline can be found in Fig. 1a. Here we briefly summarise each step with detailed explanations appearing in the Methods section.

Annotate: SV calls are required as input into the annotate step (single-nucleotide resolution paired SV loci), and the corresponding whole-genome sequencing file in BAM format. The annotate step determines the read directionality of SVs and classifies the SV type.

Count: The count step estimates the supporting and normal (non-supporting) read counts and computes SV VAFs.

Filter: The filter step removes low-quality SVs and those with missing information, and, given copy-number calls, infers the background copy number for each break-end.

Cluster: The cluster step simultaneously estimates the mutated copy number of SVs, the number of clusters and their respective CCF means. Allele frequencies from both break-ends of each SV are used to perform inference.

Post assign: The post-assign step (re)assigns variants a most-likely mutated copy number and CCF, given the previously obtained clustering configuration.

### Estimation of SV allele frequency

SV variant allele frequencies can be estimated in the same way as SNVs: the number of variant reads divided by the total number of reads observed at the SV breakpoint. The challenge for SVs is that many reads are split across the breakpoint making extracting accurate estimates for these read counts difficult. To explore how best to deal with this challenge we simulated reads from SVs with known allele frequency, at varying tumour purity. We then implemented an optimised approach for computing a VAF from these read counts (see Methods). The simulations revealed that the VAF estimates were accurate, independent of purity, except for duplications (Fig. 1c). Duplications showed an increased normal read count due to DNA gains showing no loss of normal DNA (Fig. 1b). To account for this bias, we introduced a scaling factor that incorporates tumour purity to calibrate the supporting read counts. This corrected for the bias and showed accurate estimation of the underlying VAF (Fig. 1c).

### In silico subclonal mixing of tumours for validation

**Fig. 2:**
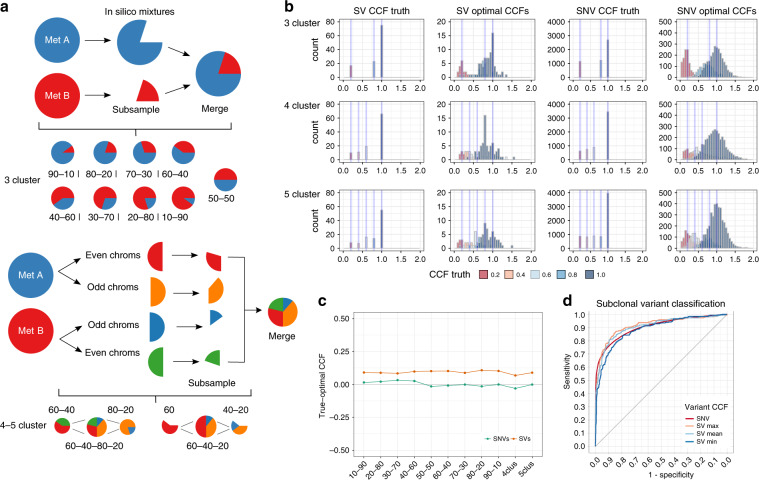
In silico mixing strategy and optimal CCF calculation metrics. **a** A schematic illustrating the subsampling and merging process used to create in silico mixtures of real tumour samples. The top diagram shows the three-cluster in silico mixtures, created by mixing the two metastasis samples in different proportions. The bottom diagram shows the methodology for creating the four- and five-cluster mixtures, which separates each mixture sample into even and odd chromosomes, then subsamples these samples to create additional clusters. The resultant CCFs are based on the subsampling percentage of each odd or even chromosome sample, rather than the sample proportion (as in the three-cluster mixtures). **b** The CCF ground truth (based on sample membership) versus optimal SV and SNV results (based on transformed variant allele frequencies from the true cluster mean) for a representative three-cluster mixture and the four- and five-cluster mixtures. **c** Mean per-variant CCF error of optimal SNV and SV CCFs compared with the expected, ground truth CCF. **d** ROC curves for classifying variants as clonal or subclonal based on optimal variant CCFs.

Recently, a number of efforts have been made to simulate datasets with known subclonal structure to assess the performance of algorithms that infer the CCF of mutations^21,22^. However, these have been limited to simulating SNVs and copy-number changes. To date, a gold standard dataset to test the performance SV cancer cell fraction inference does not exist. Therefore, we created a dataset of tumour samples with known SV subclonal structure. Rather than simulate SVs, we opted to mix two whole-genome sequenced samples from the same patient^23^, in silico, at known subclonal proportions (Fig. 2a). By mixing tumour sequence data, we maintained many of the noise characteristics of real sequence data. Our samples consisted of a set of three-cluster mixtures with SV and SNVs subsampled with known clonal frequencies at 10% increments, as well as four and five-cluster mixtures created by subsampling odd and even chromosomes at different frequencies (Fig. 2a). The prostate cancer samples used to create the mixtures had no evidence of subclonality (Supplementary Fig. 2d from Hong et al.^23^), and had similar read coverage and tumour purity.

### Optimal cancer cell fraction versus ground truth

Our in silico mixtures allowed us to explore some of the fundamental noise properties of CCF distributions. As the read counts supporting the SVs and SNVs in our mixed samples were subject to noise (approximately binomially distributed), we hypothesised that the resulting CCF estimates must also be noisy (approximately normally distributed). To observe this, we estimated an ‘optimal’ CCF for each variant, which was calculated using the VAF and inferring multiplicity from the true mixture proportion (see Methods). This estimate allowed us to observe the optimal CCF distributions (Fig. 2c). Indeed, we observed that subclonal populations were approximately normally distributed, and those with similar CCFs had overlapping distributions (Fig. 2b).

These optimal CCF estimates also allowed us to explore any differences between SV and SNV CCF estimation. Overall, 234 high-confidence SVs and 9810 SNVs were called across both metastasis samples. The lower number of SVs resulted in the optimal CCF estimates having less clear CCF peaks than SNVs (Fig. 2b). These data highlight the difficulty in estimating CCFs for SVs as compared with SNVs. In addition, at the variant level, CCFs of SVs had a slightly higher mean error (ME) compared with SNVs (0.0461 vs. 0.015 across all cluster mixtures; per mixture results are shown in Fig. 2c).

Given the ground truth, these data also allowed us to determine the optimal per-variant cutoff for determining whether a variant was subclonal (Fig. 2d). We found that taking the max or mean CCF of both SV ends resulted in the highest AUC (~0.90), which was also approximately equal to the AUC obtained by classifying SNVs. The optimal CCF cutoffs for determining subclonality were 0.69 and 0.72 for SVs (using mean CCF) and SNVs respectively—to simplify this, we used a cutoff of 0.7 for both variant types for all downstream analyses.

### Performance assessment

SVclone is chiefly designed to determine the CCF of SVs in a single, whole-genome sequenced tumour sample. Common downstream analyses of these data include analysing the number of subclonal populations in a sample^24^ and observing which SVs are clonal or subclonal^25^. As such, we designed performance metrics to interrogate such variables including: cluster number error, mean cluster CCF error, mean variant CCF error, and sensitivity and specificity for calling a variant subclonal. As one of the key features of any CCF inference algorithm is to estimate the number of chromosome copies of a variant (known as multiplicity), we also observed the mean multiplicity error.

**Fig. 3:**
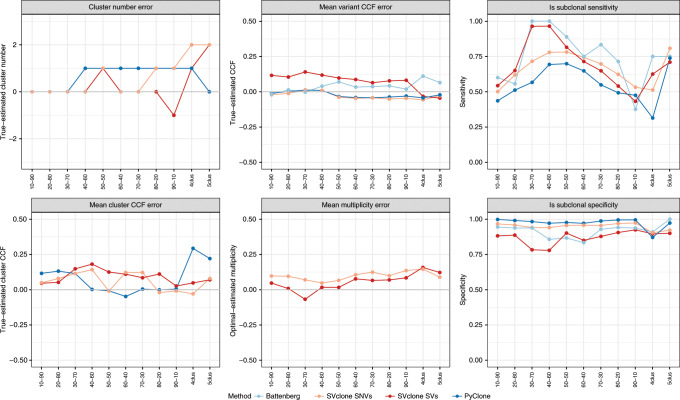
Clustering performance metrics versus existing methods. Performance of SVclone’s SV and SNV models, compared with Battenberg (SCNAs) and PyClone (SNVs) run on the in silico mixtures. The first column shows the cluster number error (three-inferred cluster number), and the mean CCF error, where true and inferred clusters are matched based on their order (see Methods). The second column shows the mean variant CCF and multiplicity error compared with the ground truth CCF. The third column shows the subclonal classification sensitivity and specificity using sample membership of the variant (i.e. a variant is classified as clonal if present in both samples of the mixture, and subclonal otherwise).

To our knowledge, no other method for estimating SV CCF exists for direct comparison. Instead we opted to compare to two representative, state of the art methods for estimating the CCF of SNVs, PyClone^10^, and copy number, Battenberg^15^, from single samples. In addition, we also ran SVclone in SNV clustering mode, which uses Ccube’s clustering model^26^. Performance is summarised in Fig. 3, and a breakdown of the performance under each measure can be found below.

Cluster number error: This metric indicates how effective the given clustering algorithms were at inferring the correct number of clusters. SVclone applied to the in silico mixtures was able to identify the correct number of clusters in 7 of 11 cases (Fig. 3). SVclone’s SNV clustering found the correct number of clusters in 5/11 cases, compared with PyClone’s 4/11, suggesting that SVs may have a slight advantage in identifying the correct number of underlying clusters.

Mean cluster CCF error: Mean cluster CCF error was generally higher in the SV data, with an average mean error of 0.0913, compared with 0.0412 and 0.0756 observed in the SNV data by SVclone and PyClone respectively. This is likely due to the variant number differences, as the comparatively larger number of SNVs is likely to lead to more accurate cluster CCF estimates.

Mean variant CCF error: Similarly, mean variant CCF error was slightly higher in the SV data than other methods. SV CCFs had an average mean error of 0.0873, compared with −0.034 for SVclone SNVs, −0.0213 for PyClone, and 0.0375 for Battenberg. Slightly higher error rates for SV CCFs are expected, given that the optimal (i.e. best obtainable given knowledge of cluster means) CCF mean errors averaged 0.0408 and 0.002 for SVs and SNVs respectively (Fig. 2c). Notably, while Battenberg performed on average better than SVclone in terms of mean variant CCF error for the three-cluster mixes, SVclone performed better on the four- and five-cluster mixtures, demonstrating SVclone’s advantage in being able to consider >2 subclones. SVclone’s SNV clustering and PyClone displayed similar mean error trends across the mixtures. Given the relatively smaller number of variants used in the clustering compared with SNVs, and the fewer data points used to infer fraction compared with SCNAs, SV CCF mean errors were in general comparable to other methodologies, with <0.05 absolute difference, on average, across the mixtures.

Sensitivity and specificity for calling a variant subclonal: SVclone’s SV estimates demonstrated similar sensitivity to SNVs when classifying a variant as subclonal, with an average sensitivity of 0.670 (compared with an SNV sensitivity of 0.6643). The SVs had a lower specificity (0.8852 vs. 0.952 with SNVs). PyClone displayed a lower sensitivity, but higher specificity than the other methods at 0.577 and 0.9687 respectively. Battenberg had the highest average sensitivity and specificity (sens = 0.747, spec = 0.9175), which is expected given the number of data points (germline SNVs) used by Battenberg to infer each copy-number fraction.

Multiplicity error: Multiplicity error represents the difference in the multiplicity inferred from clustering, compared with the inferred multiplicity given the ‘true’ CCF cluster mean (as multiplicity cannot directly be observed). As PyClone averages across all possible multiplicities, and does not directly estimate multiplicity, we did not consider PyClone for this metric. Average multiplicity errors were −0.0391 for SVs and 0.1029 for SNVs. The lower multiplicity error rate in SVs is likely due to the subclonal copy-number inference model (only SNVs with clonal copy numbers were considered), which allows for non-integer copy numbers. The mean multiplicity error for clonal SVs across the three-cluster mixtures was −0.1239, similar in absolute terms to the SNV multiplicity error (0.1029).

SVclone’s comparable performance to SNV-based clustering indicates that clonal structure can be effectively reconstructed with high concordance and accuracy, despite the relative deficit in variant number. This means that the clonal structure of a tumour can be inferred from SNVs and SVs independently and their results compared. However, if it is assumed that the clonal populations in a sample share the same SNVs and SVs, we have also provided an option to cluster both SVs and SNVs using the same clustering framework. This is particularly powerful when considering model-based post-assignment. SNV CCF posterior can be integrated with SV read counts’ likelihood to make assignment calls and vice versa (see Supplementary Fig. 1). By combining these data types overall performance can be increased.

Two of SVclone’s unique design features also warranted further performance assessment: (1) SVclone incorporates background SCNA states from both breakpoint ends into its clustering model; and (2) SVclone clusters variants in clonal and subclonal copy-number regions. Here, we sought to quantify the advantages of both approaches over ‘naive’ approaches which considered only one breakpoint for each SV, or used only variants in clonal copy-number regions.

**Fig. 4:**
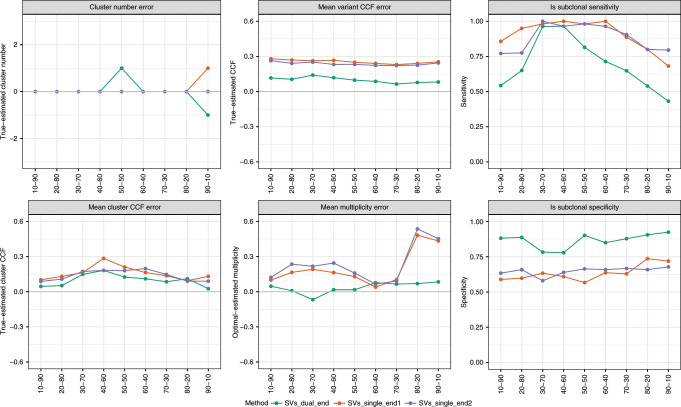
SV clustering performance for dual versus single-end models. Performance of SVclone run on three-cluster in silico mixtures using either both breakends of an SV, or a single end. The first column shows the cluster number error (three-inferred cluster number), and the mean CCF error, where true and inferred clusters are matched based on their order (see Methods). The second column shows the mean variant CCF and multiplicity error compared with the ground truth CCF. The third column shows the subclonal classification sensitivity and specificity using sample membership of the variant (i.e. a variant is classified as clonal if present in both samples of the mixture, and subclonal otherwise).

To compare the performance of SVclone’s dual-end clustering model, to a single end, we ran the respective SV sides from the three-cluster in silico mixtures through SVclone’s single-end (SNV) model, and compared the clustering performance to the dual-end model. Performance is summarised in Fig. 4. Figure 4 shows that dual-end model outperforms the single-end model across mean variant CCF error, mean multiplicity error, and mean cluster CCF error across almost all mixes. Only the cluster number of the 50–50 mix was incorrectly inferred, compared with the single-end model which was correct, However, we would expect only two clusters given the 50–50 mixture split and thus the dual-end model’s result is likely more parsimonious with the data. Interestingly, the single-end model showed a higher subclonal classification sensitivity, but a lower specificity than the dual-end model. Given that this metric represents a sensitivity and specificity trade-off, we generated a ROC curve (Supplementary Fig. 2). Considering the AUC indicates that the dual-end model is preferable (AUC of 0.8234 vs. 0.8095 for the dual and single-end models respectively).

We further hypothesised that the dual-end model was more robust to SCNA noise. To investigate this point, we selected the 70–30 in silico mixture due to its low variant CCF error, and perturbed copy number in the following ways: (i) major allele copy number − 1 (CN − 1 for short), (ii) major allele copy number + 1 (CN + 1), and (iii) subclonal copy-number fraction +/−0.3, where, 0.3 is added to the copy-number fraction if the resulting fraction is <0.9, otherwise we subtract 0.3 (see Methods for further details). We performed these experiments for the dual-end model, perturbing one side and both sides in separate runs. As expected, we found that the CN-perturbed runs showed slightly worse performance across the measured metrics compared with the unperturbed runs (Supplementary Fig. 3). In general, variant-level metrics were more severely affected than cluster-level metrics. All perturbations performed similarly, with CN − 1 (experiment i) on both sides being the most affected scenario. Mean variant CCF was most significantly affected with a 0.27 error in the CN − 1 scenario on both sides (compared with 0.07 in the unperturbed model). Mean cluster CCF error was only mildly affected, but was also most significant for the CN − 1 on both sides scenario (0.16 vs. 0.11 ME in the unperturbed data). The CN − 1 experiments were the only ones that caused an error in the cluster number. Supplementary Fig. 4 shows the effects of the perturbation experiments on the single-end model versus the dual-end model (where only one side is perturbed). The dual-end model was more robust to perturbation across all metrics for all perturbations except for cluster number with the CN − 1 experiment (where one extra cluster was called), subclonal classification sensitivity in the CN − 1 experiment and a slightly worse mean multiplicity error in the CN + 1 scenario. Interestingly, mean cluster CCF error was still lower in the over-clustered case. Importantly, the mean variant CCF error and mean cluster CCF error were lower in all cases when considering the perturbed dual-end model versus the perturbed single-end. In summary, these data show that the dual-end model is more robust to copy-number noise than the single end. Copy-number addition errors were better tolerated than subtraction errors, and a mis-estimation of copy-number fraction resulted in errors somewhere between the two. However, mean cluster CCF error and cluster number were minimally affected, suggesting that poor CN estimation effects are largely restricted to errors in variant-level estimates.

**Fig. 5:**
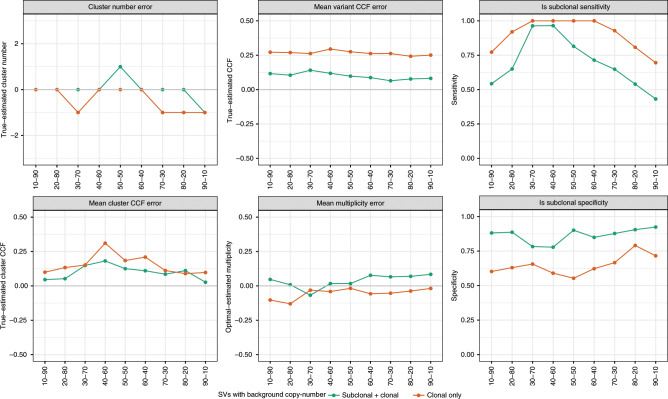
SV clustering performance incorporating background subclonal copy-number states. Performance of SVclone run across the three-cluster in silico mixtures using either clonal background copy-number states, or clonal plus subclonal states. The first column shows the cluster number error (three-inferred cluster number), and the mean CCF error, where true and inferred clusters are matched based on their order (see Methods). The second column shows the mean variant CCF and multiplicity error compared with the ground truth CCF. The third column shows the subclonal classification sensitivity and specificity using sample membership of the variant (i.e. a variant is classified as clonal if present in both samples of the mixture, and subclonal otherwise).

Finally, we compared SVclone’s performance using SVs in both clonal and subclonal copy-number regions, to clonal only. Performance is summarised in Fig. 5. Utilising all available SVs improved the performance significantly across all metrics (apart from subclonal classification specificity) compared with clustering SVs with clonal background copy numbers states only.

### Clonality analysis of 1705 whole-genome sequenced tumours

We applied SVclone to 1705 WGS samples from the pan-cancer analysis of whole genomes (PCAWG) project (dcc.icgc.org/pcawg)^20,27^, clustering both SVs and SNVs separately. An analysis of the clonality of putative driver SV events can be found in Dentro, et al.^30^ Here, we sought to observe any differences in the clonal structure of SVs compared with SNVs. Downstream analysis was performed on 23 tumour types showing ≥20 samples, with >10 SVs, and >10 SNVs, and sufficient power to detect subclonality (total *n* = 1169, see Methods).

**Fig. 6:**
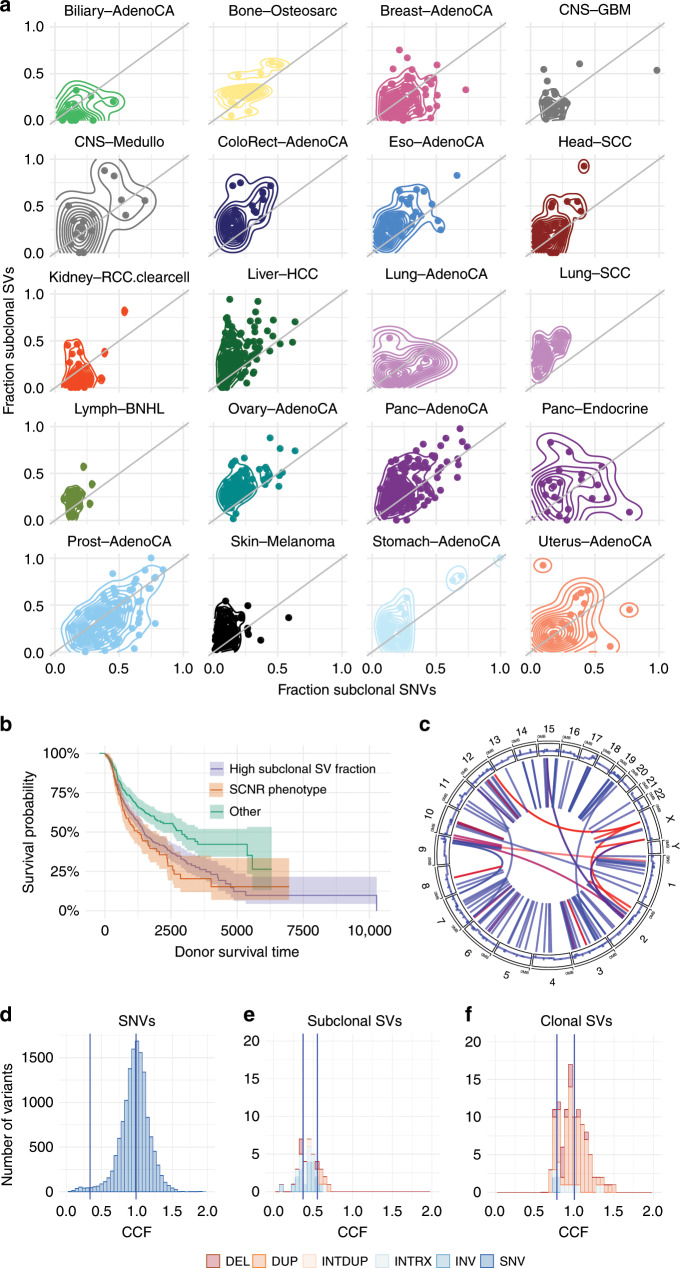
Application of SVclone to PCAWG cohort. **a** A 2D density plot of the fraction of subclonal SVs versus SNVs for PCAWG samples (*n* = 1169) (a variant under 0.7 CCF was considered subclonal). **b** Survival curves representing patients divided into those with a SCNR pattern, those with high subclonal SV fraction, or neither. **c** A circos plots for an example SCNR pattern tumour (Liver Hepatocellular carcinoma, tumour WGS aliquot 2bff30d5-be79-4686-8164-7a7d9619d3c0). The outside track represents the copy number across the genome and the inner lines indicate SVs. Blue lines represent clonal SVs and red lines represent subclonal SVs. **d** A CCF histogram of sample 2bff30d5-be79-4686-8164-7a7d9619d3c0’s SNVs. **e** A CCF histogram of 2bff30d5-be79-4686-8164-7a7d9619d3c0’s subclonal SV’s colour coded by SV category. **f** A CCF histogram of 2bff30d5-be79-4686-8164-7a7d9619d3c0’s clonal SVs.

A comparison of the fraction of subclonal SVs versus SNVs showed different patterns across tumour types (Fig. 6a). Tumour types showing a greater proportion of subclonal SVs versus SNVs included 100% of lung squamous cell carcinomas, and 92% of both colorectal adenomas and ovarian adenocarcinomas. In contrast, 23% of biliary adenocarcinomas had a greater proportion of subclonal SNVs versus SVs (Supplementary Table 1). Some cancers also contained subsets of samples with distinct patterns of clonality, for instance, liver cancers contained a cluster of 19 samples with high SV subclonality (≥50%) and low SNV subclonality (<30%).

One unique feature of SVclone is that it determines the clonality of copy-number neutral rearrangements (inversions and inter-chromosomal translocations). We applied a test for enrichment of subclonal copy-number neutral rearrangements across the PCAWG cohort. A total of 177 samples across 28 cancer types exhibited a subclonal copy-number neutral rearrangement (SCNR) pattern (e.g. Fig. 6c–f, see Supplementary Fig. 5 for the distribution of the pattern across histologies), with ovarian (*n* = 29, 25.7% of total ovarian), liver hepatocellular carcinoma (*n* = 26, 10.4% of liver samples) and pancreatic cancers (*n* = 18, 7.5% of total pancreatic) overrepresented in this set.

To test for potential clinical relevance of this SCNR pattern, we compared the overall survival of SCNR cases (*n* = 177), with high SV heterogeneity cases (*n* = 650), and all remaining cases (*n* = 447) for which overall survival was recorded, stratified on age, tumour histological subtype, and number of SVs. These groups showed significantly different survival probabilities (*p* = 0.006, likelihood-ratio test), with median survival times of 1236, 1470 and 2907 days, respectively (Fig. 6b). This resulted in a hazard ratio of 1.930 for SCNR cases, significantly higher compared with the baseline cohort (*p* = 0.0014, Z-test). In contrast, the high SV heterogeneity cases had a hazard ratio of 1.302 (*p* = 0.084, Z-test). Given the high number of ovarian samples within the SCNR cohort, we also considered whether fold-back inversions (FBI) were enriched, as they have been previously associated with poor prognosis^28^. We found no evidence for enrichment of FBIs (see Supplementary Fig. 6 and methods for further details), suggesting that the SCNR genotype might arise from an independent mechanism.

To test if these SCNR events were the result of a single complex rearrangement event (such as chromothripsis), or were simply a set of unrelated rearrangements, we looked for clustered events, and where possible, attempted to walk the derivative chromosome (i.e. Korbel’s^29^ fourth statistical criterion for chromothripsis, indicating that the fragments of a given chromosome form a ‘walkable’ chain of segments through consistent orientation). SCNR events were ~26% more likely to be part of a complex event, compared with background (39% vs. 31% in absolute terms; *p* = 0.002, two-sided *t* test on proportion of linked SVs between SCNR and other samples). Overall, 50% of these clustered SCNR events were linked by at least one inter-chromosomal translocation, compared with only 22% of other samples (see Supplementary Table 2), suggesting these events can span multiple chromosomes. We found a slight increase between the fraction of chromosomes that could be walked between SCNR (2.7%) and other samples (2.1%), however this was not significant (*p* = 0.4029, two-sided *t* test). Overall, these data suggest that subclonal events present in SCNR samples are likely enriched in complex, interrelated rearrangements.

To provide some insights into the aetiology of the SCNR genotype, we looked for an enrichment of SNVs/INDELs in known cancer drivers, which may cause the SCNR genotype. Specifically, we considered clonal (CCF > 0.7) mutations as they may reveal predisposing drivers to an SCNR genotype. We found an enrichment of *TP53* mutations (40.11% of SCNR samples) compared with background (14.54% of other samples; FDR < 0.0001, hypergeometric test). The enrichment of *TP53* is consistent with the reported link between TP53 mutations and complex rearrangements^30^. However, an enrichment of *TP53* SNVs/INDELs was also observed in the high SV heterogeneity cohort (36.77% of high SV heterogeneity samples), along with KRAS and CTNNB1 (all FDR < 0.001), suggesting that *TP53* may be necessary but not sufficient for an SCNR genotype.

Finally, to determine whether the enrichment of subclonal neutral SVs within SCNR samples harboured functional consequences, we identified all driver genes with candidate bi-allelic hits involving an SCNR. We considered a candidate bi-allelic hit as two separate mutation events affecting the same gene (copy-number loss, an SV within the gene body and/or an SNV/INDEL). We found that 62.15% of SCNR samples had at least one subclonal balanced rearrangement affecting a driver gene that was also affected by another mutation, almost double the rate found in the high SV heterogeneity cohort (32.15%) (Supplementary Table 3). This indicated that functionally-relevant consequences of the SCNR genotype are likely.

## Discussion

Here we have presented an integrated method for inferring the cancer cell fraction of structural variation breakpoints, and have demonstrated the importance of considering the clonality of neutral rearrangements. In cancers where copy-number neutral rearrangements are common, a significant portion of the clonal landscape has remained, until now, unexplored.

Despite the successful applications of SVclone demonstrated here, it is important to consider some of its limitations. In this work, our clustering model considers all SVs as independent events despite the fact that in some cases these SVs may be part of the same complex rearrangement. Complex rearrangements are not identified by SVclone’s classification framework, however, users may specify their own types, if known. As more sophisticated methods for classifying complex SV events become available, this could be integrated into the algorithm framework. Another limitation to consider is that all CCF clustering-based methods are affected by the power to detect variants and accurately estimate their VAFs. We present an extensive analysis investigating the effects of tumour purity, coverage and copy number (for SNVs) on the power to detect clones and subclonal mutations in Dentro et al.^25^, which is also applicable to SVs.

Inferring the evolutionary history of SVs from whole-genome sequence data is a challenging problem. One of the key goals in the field is to derive a clone tree that depicts the acquisition of SVs over time and their relationship to clonal expansions during tumour evolution. To achieve this, a number of key variables must be inferred from the data: variant allele frequencies of SV breakpoints; number of DNA copies harbouring SV breakpoints (also known as multiplicity), the cancer cell fraction of SVs, cancer cell fraction of clones, and a clone phylogeny. No one method exists that can simultaneously infer all variables, but rather existing methods tackle subsets: Fan et al.: VAF^31^, WEAVER: VAF + clonal multiplicity^32^, TUSV: subclonal multiplicity + clone CCF + phylogeny + (additionally) clone copy number^33^, Meltos: VAF + phylogeny^34^, and SVclone: VAF + subclonal multiplicity + approximate clone CCF + SV CCF. At present these methods need to be combined to achieve a more complete picture of the evolution of SVs (e.g. WEAVER + TUSV^33^ or SVclone + Meltos^34^). Thus, there remains an opportunity for future development of an algorithm that can simultaneously infer all variables.

Inferring the evolution of all variant classes, including SVs, SNVs, SCNAs, indels, and their respective clonality will ultimately be required to gain a more complete picture of the tumour heterogeneity landscape. We have presented an integrated software package for modelling the cancer cell fraction of structural variation breakpoints using single sample whole-genome sequencing data and have demonstrated its application by identifying patterns of subclonal variation. This software enables further exploration and quantification of tumour heterogeneity, and moves us closer to an integrated approach to modelling tumour heterogeneity.

## Methods

### Data input

The SVclone algorithm requires, at a minimum, a list of SV breakpoints and associated tumour BAM file. SV breakpoints can be provided as a VCF or as a tab-delimited file of paired single-nucleotide resolution break-ends. Using an SV caller with directionality of each break-end is recommended. The Socrates^24^ output format is natively supported and allows additional filtering by repeat type and average MAPQ. An associated paired-end, indexed whole-genome sequencing BAM file is required. In the filter step, copy-number information can be added in Battenberg^15^, ASCAT^35^ or PCAWG consensus copy-number formats to aid in correcting VAFs. SNV input is also supported in multiple VCF formats (sanger, mutect, mutect call-stats and PCAWG consensus). Further details of input formats can be found in the repository README file.

### SV annotation

To accurately calculate variant allele frequency (VAF) of structural variants, the following information is required: (i) the single-nucleotide location of loci comprising each breakpoint; (ii) the direction in which the break faces, i.e. whether the breakpoint is on the left (−) or the right side (+) of a locus that connects to the distant locus; and (iii) the classification of the SV. SV directionality affects read counting, as only reads on one side of each break-end will correspond to a specific breakpoint.

SVclone incorporates basic methodology to infer the breakpoint direction (ii) and classification (iii) of the SV, however, we recommend using the information provided by the SV caller if it is available. SVclone will infer the directionality of each break-end by determining which side of the break-end has soft-clipped reads. If SVclone finds evidence of soft-clips lying on both sides of the break-end (i.e. at least 10% of soft-clipped reads support the opposite directionality), we consider the directionality for this break-end as mixed (i.e. multiple break-end pairs are involved for this event). If only one break-end of a pair has mixed directionality, the SV will be split into two events, one where the mixed-evidence locus is (−), and the other end is (+). If both ends have mixed directionality, we attempt to resolve this by searching the SV input for other SV events matching the SV break-ends, considering the following scenarios (see Supplementary Table 4 for a summary). We denote each SV as *j* = 1..*J*; *i* ∈ [*l*, *u*] where *l* = lower break-end locus and *u* = upper break-end locus, *l* < *u* if the chromosome is the same, or the lower of the chromosomes for inter-chromosomal translocations.

### SV directionality inference

Directionality is determined for each SV as follows: (i) neither *l* nor *u* matches any other event: the SV breakpoint is considered to be (−, −), and a new SV breakpoint is created with directionality (+, +); (ii) both *l* and *u* match (within a threshold): we consider one pair’s directionality as (−, −) and the other pair’s as (+, +); (iii) two matching breakpoints are found, each break-end matching one locus of each partner only: if the positional rankings of the three SV breakpoints (on a single chromosome) are [(1, 2), (2, 3), (1, 3)], we consider this a translocation event, and assign the directions of [(+, −), (+, −), (−, +)]; (iv) more than two matching breakpoints are found: the SV breakpoint is considered a complex event, and is discarded at the count step.

The directionality inference does not utilise local realignment of reads. The functionality is not intended to provide comprehensive and robust annotation. We recommend that directionality be inferred from the SV caller of choice.

### SV classification

After resolving directionality, we employ a decision-tree based approach to classify SV events into categories if this information is unavailable from the SV caller (see Supplementary Fig. 7). We consider six simplified categories of rearrangements: inversions, deletions, tandem duplications, interspersed duplications and intra- and inter-chromosomal translocations. Inversions refer to a flipping of a segment of DNA, where the head of one segment joins the tail of another at both ends. Deletions are considered a loss of DNA at a locus where the flanking non-deleted segments join directly, without the intervening deleted sequence. Duplications are split into two categories: tandem and interspersed. The former category consists of a duplication joining tail to head immediately one after another. In the latter case, the duplication may be interspersed anywhere within the same chromosome. An intra-chromosomal translocation is similar to interspersed duplications, except that the original mobile element is deleted rather than retained. Inter-chromosomal translocations are defined as any joining event involving different chromosomes.

The classification heuristics are shown in Supplementary Fig. 8 and are summarised as: (i) inversion (INV): (*l*, *u*) directionality matches, i.e. (+, +) or (−, −), and there are 1 or 2 breakpoints corresponding to the inversion event; (ii) deletion (DEL): (*l*, *u*) directionality is (+, −), where *l* < *u*; (iii) tandem duplication (DUP) - breakpoint directionality is (−, +), where *l* < *u*; (iv) interspersed duplication (INTDUP): requires two breakpoints, (*l*, *u*)_1_ and (*l*, *u*)_2_ where *l*_1_ ≈ *l*_2_ (within 100 bp) and *u*_1_ ≠ *u*_2_ (one breakpoint has a tandem duplication signature and the other a deletion signature, i.e. (*l*, *u*)_1_ = (−, +) and (*l*, *u*)_2_ = (+, −)); (v) intra-chromosomal translocation (TRX): the same as an interspersed duplications, except with the presence of a third breakpoint (*l*, *u*)_3_, classified as a deletion that spans the mobile element: *l*_3_ ≈ *u*_2_, *u*_3_ ≈ *u*_1_ and (*l*, *u*)_3_ = (+, −). To successfully classify a translocation, the deletion ends must be within 6 bp (by default) of both ends of the mobile element; and (vi) inter-chromosomal translocation (INTRX) - the only criteria is that the chromosomes of *l* and *u* do no match, no directionality is considered.

### Read counting

We consider three types of reads that cross the respective break-ends (*l*, *u*) (within 6 bp):

*s*_*i*_ = *s*_*l*_ + *s*_*u*_: supporting split reads at *l* and *u* respectively. These are variant reads (supporting the break) where one of the read-pairs lies across the break-end by a specified number of base-pairs, which must be greater than the soft-clip threshold (10 by default for 100 bp reads).

*c*_*j*_: supporting discordant (spanning) reads, i.e. reads that span across the (*l*, *u*) breakpoint, where each read of the pair lies on one side of the break, effectively spanning the breakpoint (see Supplementary Fig. 9). The insert distance is calculated by both reads’ distance from their respective breakpoint at both ends. One of the reads may also be soft-clipped at the breakpoint, and still be counted as a supporting discordant read (these reads are counted under the spanning read category). In addition, the read orientation of both reads is also checked to ensure both reads are oriented towards the break (this is always the case for a true spanning read supporting the breakpoint).

(*o*_*l*_, *o*_*u*_): normal read counts at *l* and *u* respectively. Either the read or the insert between the reads must lie across the breakpoint locus. These are reads derived from alleles *not supporting* the breakpoint. The outside ends of each read pair must overlap the breakpoint boundary by at least the specified base-pairs (10 by default for 100 bp reads) to be counted. Reads must not be soft-clipped above a small threshold (6 bp by default).

### Supporting read calculation

Supporting reads are only counted if reads match the specified break-end directionality; this avoids double-counting of reads for events where reads are present at both sides of the breakpoint, such as inversions and translocations (these events consist of ≥2 breakpoints per event). All reads that are counted towards the supporting or normal read totals must have an insert size (fragment size) < *μ*_*ins*_ + (3·*σ*_*ins*_), where *μ*_*ins*_ = the mean of the insert size and *σ*_*ins*_ = the standard deviation of the insert size. (The insert size for supporting spanning reads is considered the adjusted insert size for this criterion.) This is a quality-checking measure to ensure only high-confidence reads are counted. We consider both spanning and split reads together as the total supporting read count: *b*_*i,j*_ = *s*_*i,j*_ + *c*_*i,j*_.

SV breakpoints where at any break-end the average read depth exceeds *λ*·*maxn*_*j*_ are considered high depth regions and are ignored, where *λ* is the expected number of reads per locus and *maxn*_*j*_ is the maximum expected copy-number value (coverage and maximum expected copy number can be defined by the user). These breakpoints are likely caused by repetitive regions, rather than true copy-number amplifications, and are not suitable for inference of clonal composition. Bed filtering has been incorporated to automatically ignore breaks falling within specified regions (to accommodate blacklists such as DAC—www.encodeproject.org/annotations/ENCSR636HFF/).

In order to determine whether micro-homology was likely to play a large role in the read counting process, we analysed the distribution of breaks containing micro-homologies across the PCAWG samples used in the paper analysis (using PCAWG’s consensus SVs v1.6). We found that the mean and median micro-homology lengths were 1 and 2.4 respectively. Micro-homologies ≤ 6 bp in length are handled by the variable threshold used by the read counting step. We found that 6.17% of SVs had micro-homologies greater than 6 bp and <1% of SVs had micro-homologies greater than 20 bp. Given the minority of SVs affected, handling of longer micro-homologies is outside the scope of this work, and such SVs should be filtered out.

### Non-supporting read calculation

For each SV, normal reads are counted at the break-ends resulting in two normal read count totals (*o*_*l*_*, o*_*u*_). In the case where the SV results in a gain of DNA (interspersed and tandem duplications), the normal read count must be adjusted. We consider the SV classification *κ*_j_ for an SV *j*, where *κ*_j_ ∈ {DEL, DUP, INTDUP, INV, TRX, INTRX}(respectively: deletions, duplications, interspersed duplications, inversions, translocations and inter-chromosomal translocations). We define two subsets *κ*_gain_ = {DUP, INTDUP} where normal reads at the variant population's break-ends are unaffected at the variant allele, and *κ*_non-gain _= {DEL, INV, TRX} where the normal reads at the variant population’s breakends are replaced by supporting reads. We compute an adjustment factor,

1 $${{{\mathrm{AF}}}}_{norm}\,=\,1 \,- \,\left( {\frac{t}{{n_p}}} \right),$$

where *t* is the tumour content and *n*_*p*_ the tumour ploidy. The normal read counts of all DNA-gain events are then multiplied by this adjustment factor (*o*_*i,j*_ = *o*_*i,j*_·AF_*norm*_ if *κ*_*j* _∈ *κ*_gain_), while events that are not DNA-gains remain unadjusted.

### Anomalous reads

Reads that cross the SV boundary but do not meet the requirements for split, spanning or normal reads are considered anomalous and do not contribute to read counts. Reads can be considered anomalous for numerous reasons: (i) the insert distance is greater than *μ*_*ins*_ + (3·*σ*_*ins*_), (ii) discordant reads do not face the break, (iii) the read is soft-clipped at both ends, (iv) the read is soft-clipped but is either not in the vicinity of the breakpoint, boundary or the soft-clip is below the threshold, or (v) the reads support the break in the opposite direction, but have not been called by the SV calling algorithm. To investigate points i-iv, we investigated anomalous reads in the 100% purity deletion simulations, and flagged an average of 8.74 anomalous reads per breakpoint per 246.18 considered (3.57%) from the extracted regions around both break-ends of a breakpoint (these reads are proximal to the breakpoint and may not directly cross it). Upon manual inspection, we found that anomalous reads largely fell in the (iv) category, i.e. insufficiently long soft-clips or the reads genuinely did not cross the breakpoint boundary. Manual analysis uncovered no consistent under-counting of supporting reads.

### Filtering variants

While tumours may contain several thousand unique mutations, typically SVs number in the dozens to low-hundreds (for instance, in breast cancers^36^). With typically 10-fold fewer variants, each variant utilised in clustering has a higher influence on the clustering results. A conservative approach to filtering is therefore required to minimise noise propagated through variants with spurious read counts. The following filtering criteria have been implemented, with default values, to provide a baseline for minimising noise. These variants may be adjusted in cases by the user to tailor their noise thresholds to the samples under consideration. We filter on the following criteria:

### Germline variants

The output from the count step for the corresponding patient’s germline sample can be supplied to filter out any events where there is at least one supporting read in the germline for breakpoints that are considered the same event (both break-ends match directionality and are within 6 bp of each other).

### SV size

If a breakpoint is on the same chromosome, SV size (*u* − *l*) must be larger than the fragment size (by default) as otherwise supporting and normal reads may be difficult to distinguish. This criterion is only considered for intra-chromosomal events.

### Minimum support

The SV breakpoint must have at least one split and one spanning read supporting the break (*s*_*i,j*_ ≥ 1, *c*_*i,j* _≥ 1). Custom minimum values can be specified.

### Minimum depth

The minimum supporting + normal reads must be greater than the minimum depth for each break-end: (*b*_*i,j*_ + *o*_*l*_) > *b*_*min*_ and (*b*_*i,j*_ + *o*_*u*_) > *b*_*min*_. (Default *b*_*min*_ = 2).

### Copy-number state

If copy-number input is provided, either *l* or *u* must have a valid copy-number state for each variant. The major + minor copy numbers must be at least 1 for a state to be considered valid.

Optionally, in some instances it may be appropriate to filter on several further criteria:

### Copy-number neutral regions

Filters out variants with copy-number states that are not 1, 1 for major, minor alleles. Used if copy-number calls are unreliable and sufficient regions of neutral copy-number exist.

### Subclonal copy-number regions

This filter may be invoked to remove any variants with subclonal copy-number states. This reduces the copy-number search space, which is useful for clustering high numbers of variants.

### Assigning background copy-number states

Allele-specific copy-number variation can be supplied as input to SVclone in order to attach copy-number states to break-ends. We assign the estimated copy-number state that occurred before the SV occurred. For intra-chromosomal SVs, this involves obtaining the copy-number state upstream of the lower break-end and downstream of the upper break-end. For inter-chromosomal translocations, we obtain the copy number in the opposite direction of the break-end directionality. See Supplementary Fig. 10 for a conceptual schematic and Supplementary Table 5 for the mathematical representation.

Battenberg^15^ output format is preferential to capture subclonal CNAs, however, ASCAT^35^ is also supported. If no CNA information is supplied, the algorithm assumes that the total tumour copy number $( {n_{tot_t}})$ matches the normal copy number $( {n_{tot_n}})$, with no subclonality. For robustness of the algorithm results, it is recommended that copy-number information be supplied if available. If Battenberg input is defined, the first solution set of segmentations in the input is considered. We define the total copy number as the sum of each clone’s copy number, weighted by the clonal fraction:

2 $$n_{tot_t,i,j} \,= \,\mathop {\sum}\limits_{r = 1}^2 {\rho _{r,i,j}n_{tot_t,r,i,j}},$$

where $n_{tot_t,r,i,j}$ and $\rho _{r,i,j}$ are the total copy number and copy-number fraction per (copy number) clone $r \in 1,2$.

### Clustering

The clustering step of SVclone simultaneously computes SV CCFs and clusters SVs of similar CCF, based on purity, ploidy and copy-number status of the normal, reference and tumour populations. SVclone uses a bespoke clustering algorithm that takes read counts and copy-number states at both break-ends of the same SV as input, and utilises a Bayesian mixture model, implemented using variational inference, to approximate posterior distributions for unknown parameters. The algorithm determines the number of clusters automatically and infers average CCF per cluster, as well as the multiplicity of each variant (the number of mutated chromosomal copy). The model extends our previous method, Ccube^26^, for estimating and cluster CCFs for SNVs by allowing it to deal with additional read and copy-number profiles from the two break-ends. This is achieved by assigning the two break-ends of an SV to the same CCF cluster. Below is a detailed description of our clustering method.

### Read distribution

Let $i \in 1,2$ and $j \in 1,2,...,J$ be the indexes of break-ends and breakpoints respectively. We assume the supporting read counts from both breakpoints are independently distributed following two different Binomial distributions. The joint probability mass function of the supporting read counts is the following:

3 $$\begin{array}{*{20}{c}} {p\left( {{{{\boldsymbol{b}}}}_{{{\boldsymbol{j}}}}{{{\mathrm{|}}}}{{{\boldsymbol{d}}}}_{{{\boldsymbol{j}}}},{{{\boldsymbol{f}}}}_{{{\boldsymbol{j}}}}} \right) \,= \,\mathop {\prod}\limits_{i = 1}^2 {Binomial} \left( {b_{i,j}{{{\mathrm{|}}}}d_{i,j},f_{i,j}} \right)} \end{array},$$

where *b*_*i,j*_, *d*_*i,j*_, and *f*_*i,j*_ denote the number of supporting reads, the number of normal reads, and the probability of observing one support read. The bold font variable are collections of these across both breakpoints, $b_j = [b_{1,j},b_{2,j}]$, $d_j = [d_{1,j},d_{2,j}]$, and $f_j = [f_{1,j},f_{2,j}]$.

We model the probability of sampling a variant read given variant locus *j* at break-end *i* as coming from a binomial distribution with trials *d* (read depth *b*_*j*_ + *o*) and probability *f*_*i,j,k*_:

4 $$\begin{array}{*{20}{c}} {b_{i,j}|d_{i,j},f_{i,j,k}\sim Binomial\left( {b_{i,j}{{{\mathrm{|}}}}d_{i,j},f_{i,j,k}} \right)} \end{array},$$

where *b*_*i,j*_ = *s*_*i,j*_ *+* *c*_*i,j*_, (*s*_*i,j*_ is the number of split reads and *c*_*i,j*_ the number of spanning reads supporting the break). We assume the two breakpoints are conditionally independent of each other given the same CCF. In order to calculate *f*_*i,j,k*_ we require the tumour purity estimate *t* and copy-number information:

5 $$\begin{array}{*{20}{c}} {f_{i,j,k} \,= \,w_{i,j}\phi _k \,+ \,{\it{\epsilon }},with\,w_{i,j} \,= \,\frac{{t\left( {m_{i,j}\left( {1 \,- \,{\it{\epsilon }}} \right) \,-\, n_{tot_ti,j}{\it{\epsilon }}} \right)}}{{\left( {1 \,- \,t} \right)n_{tot_ni,j} \,+ \,tn_{tot_ti,j}}}} \end{array},$$

where $n_{tot_ni,j}$ and $n_{tot_ti,j}$ are the total copy number of the normal and tumour population respectively and ε is the sequencing error constant. $\phi _k,k \in 1,...,K$ represents the unknown CCF, and is indexed by *k*, representing the *k*th cluster. The other unknown parameter is *m*_*i*,*j*_, the number of mutated chromosomal copies, also known as the multiplicity of the variant. See below for how these are inferred.

To test the appropriateness of the binomial distribution for SV allele frequencies, we studied the distribution of clonal SV VAFs from the two samples used in the in silico mixtures (see below for more details). A likelihood-ratio test was performed to compare the goodness of fit of each SV using a binomial and a beta-binomial distribution. We found that the binomial distribution adequately fit most (89%) SVs. In addition, our variational formulation mitigates potential overdispersion by producing a similar effect to a beta-binomial model. The assignment probability is computed as an expectation of the binomial distribution with respect to the posterior CCF distribution; therefore, the uncertainty within the probability of success is integrated out when making assignments. Uncertainty is normally distributed in our model, while being beta-distributed in a beta-binomial model—the benefit of our choice is a fully tractable variational approximation in which all its parameters can be efficiently estimated. In the beta-binomial case, the key overdispersion parameter is difficult to estimate at typical depths obtained in whole-genome sequencing (~50x). This difficulty is evident in the high variance and lack of clear convergence observed in PyClone’s MCMC traces of its overdispersion parameter (Supplementary Fig. 11).

### Posterior inference

We estimate the unknown *ϕ*_*k*_ and *m*_*i,j*_ in Eq. (5) by variational inference (VI). Specifically, the algorithm obtains a posterior distribution over *ϕ*_*k*_ and a point estimate of *m*_*i,j*_. For *ϕ*_*k*_, we specify a Gaussian distribution as its prior. The choice is motivated by its convenience in deriving a fully trackable VI method. As a result, we obtain a maximum pseudo marginal likelihood estimator for *m*_*i,j*_. The model employs a finite mixture model, hence, we introduce additional parameters such as the mixing coefficient *π*_*k*_ and the cluster assignment variable *z*_*j,k*_, which have the standard Dirichlet and Categorical prior respectively. We use this formulation for both clonal and subclonal copy-number settings. In regions of clonal copy number, the mapping is exact. In the presence of subclonal copy number, the mapping is an approximation, in which $n_{tot_t}$ is replaced by the weighted average total tumour copy number. Here we provide a detailed description of the inference.

The variational inference method maximises the evidence lower bound (ELBO) of the marginal likelihood of the model:

6 $${logp(B|M,H)} \hfill \, =	 \, \, {log\smallint p(B|Z,\phi ,M,H)p(Z|\pi )p(\phi )p(\pi )dZd\phi d\pi } \hfill \\ {} \hfill \, =	 \, \, {log\smallint p(B,Z,\phi ,\pi |M,H)dZd\phi d\pi } \hfill \\ \ge	 \, \, {E_{q\left( {Z,\phi ,\pi } \right)}\left[ {logp\left( {B,Z,\phi ,\pi |M,H} \right)} \right] - E_{q\left( {Z,\phi ,\pi } \right)}\left[ {logq\left( {Z,\phi ,\pi } \right)} \right]} \hfill,$$

where *B* = {*b*_*i*,*j*_}, *Z* = {*Z*_*i*,*k*_}, *ϕ* = {*ϕ*_*k*_}, *π* = {*π*_*k*_}, *M* = {*m*_*i*,*j*_}. We use *H* to represent all fixed variables.

Assuming independence among the unknowns, $q(Z,\phi ,\pi ) = q(Z)q(\phi )q(\pi )$, the ELBO is maximised by the following solution:

7 $$\begin{array}{l}q\left( Z \right) \propto exp\left( {E_{q\left( {\phi ,\pi } \right)}\left[ {logp\left( {B,Z,\phi ,\pi {{{\mathrm{|}}}}M,H} \right)} \right]} \right)\\ q(\phi ) \propto exp\left(E_{q(Z,\pi )}\left[ {logp(B,Z,\phi ,\pi |M,H)} \right]\right)\\ \begin{array}{*{20}{c}} {q\left( \pi \right) \propto exp\left( {E_{q\left( {Z,\phi } \right)}\left[ {logp\left( {B,Z,\phi ,\pi {{{\mathrm{|}}}}M,H} \right)} \right]} \right)} \end{array}\end{array}$$

further details about these approximations can be found in^26^.

The multiplicities are estimated by the following maximisation formula:

8 $$\begin{array}{*{20}{c}} {\widehat m_{i,j} = argmax_{m_{i,j} \in \Xi _{i,j}}\mathop {\sum }\limits_{k = 1}^K E_{q\left( {z_{i,k},\phi _k} \right)}\left[ {logp\left( {b_{i,j}{{{\mathrm{|}}}}d_{i,j},f_{i,j,k},z_{i,k} = 1} \right)} \right]} \end{array}$$

The difference between clonal and subclonal copy number is reflected in the set of possible multiplicities:

9 $$\begin{array}{*{20}{c}} {\Xi _{i,j} = \left\{ {\begin{array}{*{20}{l}} {\left\{ {1, \ldots n_{maj_t,i,j}} \right\},{{{\mathrm{if}}}}\,{{{\mathrm{tumour}}}}\,{{{\mathrm{copy}}}}\,{{{\mathrm{number}}}}\,{{{\mathrm{at}}}}\,{{{\mathrm{SV}}}}\,{{i}},{{j}} \, \, {{{\mathrm{is}}}}\,{{{\mathrm{clonal}}}}} \hfill \\ {\left\{ {\mathop {\sum}\nolimits_{r = 1}^2 {\rho _{r,i,j}} x_{i,j}:x_{i,j} \in 0, \ldots n_{maj_t,r,i,j}} \right\},{{{\mathrm{if}}}}\,{{{\mathrm{tumour}}}}\,{{{\mathrm{copy}}}}\,{{{\mathrm{number}}}}\,{{{\mathrm{at}}}}\,{{{\mathrm{SV}}}}\,{{i}},{{j}}\, \, {{{\mathrm{is}}}}\,{{{\mathrm{subclonal}}}}.} \hfill \end{array}} \right.} \end{array}$$

with $\Xi _{i,j} = \{ 1, \ldots n_{maj_t,i,j}\}$ if the tumour copy-number segment at SV_*i,j*_ is clonal, and $\Xi _{i,j} = \left\{ {\mathop {\sum }\nolimits_{r = 1}^2 \rho _{r,i,j}m_{i,j}:m_{i,j} \in 0,...n_{maj_t,r,i,j}} \right\}$ if the tumour copy-number segment at SV_*i,j*_ is subclonal. Where $n_{maj_t,i,j}$ is the tumour major copy number at SV_*i,j*_. $\rho _{r,i,j}$ and $n_{maj_t,r,i,j}$ are the fraction and major copy number of the tumour subclonal *r* at SV_*i,j*_.

The variational inference algorithm is run over a range of possible cluster numbers (by default 1..6) and multiple repeats (by default 5). The solution with the best ELBO is selected.

### Calculating variant CCFs

Given the estimated multiplicity, *m*_*i*,*j*_, we infer CCF at individual variant level. In our Binomial model, the probability of observing a variant supporting read is specified as $f_{i,j,k} = w_{i,j}\phi _k + {\it{\epsilon }}$. We replace the cluster-level CCF parameter *ϕ*_*k*_ with variant-level CCF parameter *ϕ*_*i,j*_. As a result, we have $\phi _{i,j} = \frac{{f_{i,j} - {\it{\epsilon }}}}{{w_{i,j}}}$. The removal of subscript *k* in *f*_*i,j*_ reflects the change in CCF levels. Under the Binomial distribution assumption for variant supporting read counts, $f_{i,j} = E[VAF_{i,j}]$, $VAF_{i,j}$ is an unbiased estimator of *f*_*i,j*_. Therefore, *ϕ*_*i,j*_ can be estimated as $\frac{{VAF_{i,j} - {\it{\epsilon }}}}{{w_{i,j}}}$. Note that, the linear relationship doesn’t support a natural bound on CCF. We cap the maximum of CCF at 2. Finally, we have

10 $$\begin{array}{*{20}{c}} {CCF_{i,j} = min\left( {2,\frac{{VAF_{i,j} - {\it{\epsilon }}}}{{w_{i,j}}}} \right)} \end{array}$$

For SVs, the mean of these two CCFs is used as the representative CCF per SV.

### Post-assignment of variants to clusters

In some cases, the number of filtered SVs in a sample is too small ⪅ 10 to perform reliable clustering. To estimate the CCF of these SVs, we leverage clustering results from SNV data. To demonstrate this approach, we use the subscript $_{{{{\mathrm{post}}}}}$ to denote variables of the post-assigned SVs, e.g. *B*_post_. The strategy is to use *q*(*ϕ*_SNV_) (the subscript $_{{{{\mathrm{SNV}}}}}$ emphasises that the distribution is constructed from SNVs) from Ccube results as a reference model, then assign SVs to clusters in *q*(*ϕ*_SNV_). Algorithmically, given that Ccube and SVclone both assume *q*(*ϕ*) to be Gaussian, one can use the SNV-based *q*(*ϕ*_SNV_) to compute *q*(*Z*_post_) and *q*(*π*_post_) for the SVs.

Here, we set out to update *q*(*π*_post_) in addition to the assignment variable *q*(*Z*_post_). The reason for this is to avoid the post-assignment mimicking the mixing proportions in SNV results. The dimensions of *q*(*Z*_post_) and *q*(*π*_post_) are set to be compatible with the number of clusters in *q*(*ϕ*_SNV_). More precisely,

11 $$\begin{array}{l}q(Z_{{{{\mathrm{post}}}}}) \propto exp\left( {E_{q\left( {\phi _{{{{\mathrm{SNV}}}}},\pi _{{{{\mathrm{post}}}}}} \right)}\left[ {logp\left( {B_{{{{\mathrm{post}}}}},Z_{{{{\mathrm{post}}}}},\phi _{{{{\mathrm{SNV}}}}},\pi _{{{{\mathrm{post}}}}}{{{\mathrm{|}}}}M,H_{{{{\mathrm{post}}}}}} \right)} \right]} \right)\hfill\\ \begin{array}{*{20}{c}} {q\left( {\pi _{{{{\mathrm{post}}}}}} \right) \propto exp\left( {E_{q\left( {Z_{{{{\mathrm{post}}}}},\phi _{{{{\mathrm{SNV}}}}}} \right)}\left[ {logp\left( {B_{{{{\mathrm{post}}}}},Z_{{{{\mathrm{post}}}}},\phi _{{{{\mathrm{SNV}}}}},\pi _{{{{\mathrm{post}}}}}|M_{{{{\mathrm{post}}}}},H_{{{{\mathrm{post}}}}}} \right)} \right]} \right)} \end{array}\end{array}$$

The multiplicities of the post-assigned, $\widehat m_{i,j,{{{\mathrm{post}}}}}$, are estimated as:

12 $$\begin{array}{*{20}{c}} {argmax_{m_{i,j,{{{\mathrm{post}}}}} \in \Xi _{i,j}}\mathop {\sum}\limits_{k = 1}^K {E_{q\left( {z_{i,k,{{{\mathrm{post}}}}},\phi _{k,{{{\mathrm{SNV}}}}}} \right)}} \left[ {logp\left( {b_{i,j,{{{\mathrm{post}}}}}{{{\mathrm{|}}}}d_{i,j,{{{\mathrm{post}}}}},f_{i,j,k},z_{i,k,{{{\mathrm{post}}}}} = 1} \right)} \right]} \end{array}$$

where the set of possible states $\Xi _{i,j}$ is of the same form with the settings in the main clustering algorithm, Eq. (9). Similar to above, and SVs that were initially filtered can be post-assigned to the SNV or SV clusters.

### Quality control

We use the same quality-checking steps for both clustering and post-assign. They are made of three steps: (1) remove empty clusters, (2) remove small clusters with less than 1% of the data assigned, (3) merge clusters with cluster means less than 10% apart. In each step, the model parameters are refined with the same variational inference procedures.

### SV simulation

SVs were simulated by first rearranging the reference genome to create an artificial genome containing SVs, and then simulating reads with SimSeq^37^ from this rearranged reference. The reads were then mapped back to the original, unmodified reference genome using bowtie2 with the local alignment flag^38^. SV size was randomly chosen among the size categories 300–1 kb, 2–10 kb and 20–100 kb with equal probability for each category.

We simulated a single, heterozygous chromosome 12 (being roughly representative of genome-wide GC-content) with SVs of a single type at every 100 kb interval. Samples containing only deletions, translocations, inversions and duplications were generated at the tumour purity levels of 100, 80, 60, 40 and 20%. We generated 100 bp paired-end reads with an average fragment size of 300 bp and an insert-size standard deviation of 20 bp. The SV events were assumed to always occur in the heterozygous fashion, hence the ‘true’ VAF was always considered to be half of the simulated purity value. To achieve the effect of differing purities, simulated normal reads were mixed with tumour samples with coverage equivalent to $\frac{{\lambda \cdot t}}{2}$ and normal read coverage of $\frac{{\lambda \cdot (1 - t)}}{2}$ where *λ* represents the expected total read count at a locus. We ran simulations at 50x coverage, typical for WGS data by simulating $\frac{{50L}}{{300}}$ total reads per simulation where *L* is the chromosome length (post rearrangement) and 300 is the fragment length. The number of reads generated for genomes with variants that changed the total size of the genome (deletions and duplications) was adjusted by the new genome length.

The list of SV breakpoints for each simulation run was collated, directions were inferred and each breakpoint was classified using SVclone’s annotate step. Any breakpoints where the direction could not be inferred correctly, or their classification was incorrect were discarded. The resulting set was run through SVclone’s count step with default parameters. SVs were then filtered through the filter step, and adjusted VAF field was used to compare variant frequencies at corresponding purity levels.

### Prostate sample mixing

The metastatic samples bM (A) and gM (B) from Patient 001^23^ were chosen due to their similar coverage (51.5x and 58.9x) and purity (49 and 46%). Previous analysis by Hong et al.^23^ showed that these metastases shared a common ancestral clone, had no evidence of subclonality, and contained a number of private SVs and SNVs. Mixing two clonal metastases from the same patient has many advantages over spike-in approaches including: realistic sequencing noise, realistic subclonal mixing of SVs, SCNAs and SNVs, and a natural branching clonal architecture with both clonal and subclonal mutations present. We generated a total of nine samples with subclonal mixes of reads sampled at percentages 10–90, 20–80, 30–70, 40–60, 50–50, 60–40, 70–30, 80–20, and 90–10 for metastasis A and B, respectively. Three clusters are expected to be revealed upon mixing: shared variants present at 100% CCF, one cluster at bM’s mixture frequency and one cluster at gM’s mixture frequency. We also generated mixtures of four and five clusters each. The four cluster mixture was constructed by subsampling bMʼs odd and even chromosomes separately at 20 and 60% respectively, and then mixing this with a 40% subsampled mixture from gMʼs odd chromosomes only (effectively creating a mixture where odd and even chromosomes comprise 60% of either one or both samples, with CCFs of 20%, 40 and 60%). Similarly, the five-cluster mixture was constructed by subsampling bMʼs odd and even chromosomes separately at 80 and 60% respectively, and gMʼs odd and even chromosomes at 20 and 40% respectively (effectively creating a mixture where odd and even chromosomes comprise 100% of both samples, with CCFs of 20, 40 and 60 and 80%).

A merged variant list was created for SVs and SNVs, containing both the individual sample’s high-confidence calls. SV breakpoints were then run through SVclone’s complete pipeline, and SNVs were counted at each variant locus using Samtool’s mpileup^39^ and pileup2base (https://github.com/riverlee/pileup2base) for each mixture. Battenberg was run on each mixture to obtain SCNA data and purity estimates (which were used as the purity values for both SVclone and PyClone). A truth set was created for benchmarking purposes, constructed for SV, SNV and SCNA by determining whether the variant was unique to one sample, or shared in both.

In silico mixtures were created using the subsample and merge functions from SAMtools v1.2. copy numbers were obtained from Battenberg on each merged sample with default parameters. To construct the breakpoint list for input into SVclone’s annotate step, Socrates was run on the individual bM and gM samples, then run through SVclone’s annotate and count steps (using Socrates’ directions, filtered on simple and satellite repeats using the repeat-masker track (repeatmasker.org) and a minimum average MAPQ of 20). The resulting bM and gM SVs were then merged and filtered against the germline. copy numbers were matched using corresponding Battenberg subclonal copy-number output. The merged SV list was used as the set of SV calls for the annotate step for each mix.

The reference and variant alleles were counted at each of the 9810 SNVs across the different mixture proportion BAM files (Mutect variant calls from Hong et al. were used with alleles recounted using Samtool’s mpileup and pileup2base (https://github.com/riverlee/pileup2base) using a minimum quality and MAPQ cutoffs of 20 to count a base. Battenberg was run on each mixture and was used to provide copy-number information for each variant locus, as well as the purity estimate for both SVclone and PyClone. For SNV clustering, we filtered out any variants in regions of subclonal copy number (PyClone does not support subclonal copy-number handling). To improve performance, cluster labels from the PyClone traces were obtained using the mpear function from the mcclust R package https://cran.r-project.org/web/packages/mcclust. Variant and cluster CCFs were calculated from the mean MCMC trace values. We subsampled 5000 variants from the resulting SNV output per mixture and ran these variants through the PyClone algorithm for 2500 iterations with a burn-in of 1000.

Set ownership of SVs (whether the SV is present in bM only, gM only or shared between the two), was determined by running the union list of variants through SVclone against each 001 sample. If there were any supporting reads for both samples, the SV was considered shared, otherwise it was considered unique to the sample that it exclusively appeared in. For the SNVs, a variant was considered exclusive to a sample if it was called in one individual sample’s consensus SNV list only, and shared if it appeared in both lists. To test set ownership of SCNAs, Battenberg calls run on bM and gM were analysed. Any SCNAs present in both bM and gM (where start and end coordinates had to be at least within 5 kb of each other) that contained the same allelic copy numbers were considered shared SCNAs. Any SCNAs with partial matches (only one end matched, or copy numbers differed) were discarded. All other SCNAs from were considered unique to their respective samples. The mixture SCNAs were then compared with this list (where both ends must match within a 5 kb boundary). The SCNA fraction that matched the given sample’s allelic copy number was used as the SCNA’s CCF estimate. To calculate mean cluster CCF error, we compared: (i) highest ground truth CCF to highest derived cluster CCF, (ii) lowest ground truth CCF to lowest derived cluster CCF, (iii) second-highest ground truth CCF etc. alternating between highest and lowest in ranked order until either there are no more derived clusters or no more truth clusters.

### Determining optimal variant multiplicities and CCFs

To calculate the optimal CCF per variant across all mixes, we took the true mixture state of the variant, and inferred the best multiplicity. For example, in the 70–30 mixture, if an SV was present only in bM and not in gM, the true cluster CCF of 0.7 was used as the *ϕ*_*k*_ when calculating *f*_*i*,*j*,*k*_ (the binomial probability of sampling a variant read for the given locus). The multiplicity was then inferred using Eq. (6). An adjusted variant CCF could then be determined using the same method outlined in the section titled calculating variant CCFs.

### Testing the distribution fit of SV allele frequencies

To test whether a binomial model was appropriate for modelling SV VAFs, SVclone’s annotate to filter steps were run on the bM and gM metastases from patient 001, retaining only variants with copy-number neutral states (1/1 for major/minor allelic copy numbers). For each variant, a binomial likelihood for the observed number of supporting reads was calculated using *p*_*j*_ = *t*/2 (the theoretical heterozygous variant frequency) and *d*_*j*_ equivalent to the observed variant’s adjusted read depth. A beta-binomial likelihood was also calculated where the *β* value was empirically estimated from the data as:

13 $$\begin{array}{*{20}{c}} {\beta \,= \,\frac{{\left( {\mu \,- \,n} \right)\left( {\mu ^2 \,- \,\mu n \,+ \,\sigma ^2} \right)}}{{\mu ^2 \,- \,\mu n \,+ \,n\sigma ^2}}}, \end{array}$$

where $\mu = \mu _d(t/2)$ (mean adjusted depth multiplied by the theoretical heterozygous variant frequency) and *σ* is the standard deviation of the observed supporting reads. *α* was then estimated as:

14 $$\begin{array}{*{20}{c}} {\alpha = - \frac{{\mu \beta }}{{\mu - d_j}}} \end{array}$$

A likelihood-ratio test was then applied with one degree of freedom to each variant with the binomial distribution as the null model, and the beta-binomial as the alternative. Of 55 SVs tested in the bM sample, 6 SVs rejected the null model (10.9%); 2 of 44 SVs (4.5%) rejected the null model in the gM sample. Therefore, >89% SVs of moderate purity and coverage appeared to be consistent with the binomial model, indicating that the binomial model is a reasonable choice of distribution for SV data given the moderate coverage and purity of the analysed datasets.

### Copy-number perturbation experiments

We selected the 70–30 in silico mixture due to its low mean variant CCF error for the SCNA perturbation experiments. In order to perform experiments representative of the background copy-number heterogeneity prevalence, we quantified the per-sample fraction of SVs that had different background copy-number states across PCAWG. Supplementary Table 1 includes the medians of this measure cohort-wide and by histology group. We observed a range of background copy-number heterogeneity across the cohort, with a minimum (median) of 34% in non-Hodgkin lymphoma and a maximum (median) of 74% in colorectal adenoma. The median across all cancer types was 0.53. Given this result, we investigated the same measure in the three-cluster in silico mixtures and identified a lower background SCNA heterogeneity rate (potentially due to SCNA averaging as background SCNA heterogeneity was higher in the pure 001 bM and 001 gM samples) (see Supplementary Table 6). We therefore randomly removed SVs with homogeneous background SCNAs until the rate of heterogeneity was 50%, resulting in 45 SVs with homogeneous and heterogeneous background SCNA states, and used these data for downstream experiments (see the SVclone_Rmarkdown notebook under code availability to replicate this analysis).

SCNAs were perturbed as follows: (i) CN − 1: major alleles were subtracted by one in the fraction A subclone in Battenberg. If the copy number was 1, subtracting one from the minor allele was attempted. If the copy-number state was 1-0, no modification was performed (only two SVs were unable to be changed); (ii) CN + 1: major alleles were incremented by one for the fraction A clone; (iii) Frac ± 0.3: 0.3 was added to the SCNA fraction for subclonal SVs, unless that modified fraction were to exceed 0.9, in which case 0.3 was subtracted. The new SCNA fraction of clone B was calculated as one minus the new fraction of clone A.

The above experiments perturbed the *l* side of each SV for the one-side experiments, and both the *l* and *u* sides for both-side experiments. Performance metrics were calculated as usual with the two single-end metrics averaged out between the two runs (Supplementary Fig. 3).

### Analysis of ICGC/TCGA pan-cancer samples

We utilised the pan-cancer analysis of whole genomes (PCAWG) October 12th 2016 consensus SNV call set, the v1.6 consensus SVs and the consensus subclonal copy numbers (19th of January 2017). For a detailed explanation on how these were generated, see^25^. Annotate and count were run using each sample’s associated mini-bam. Consensus purity and ploidy estimates (January 9th 2017) were used. Sample SVs and SNVs were run separately through SVclone’s SV and SNV clustering model with default parameters.

We considered only white-listed PCAWG samples that had sufficient power to detect subclonality (number of reads per chromosome copy or NRPCC > 10; *n* = 1705, see Supplementary Note 1 for a list of samples). As a QC measure, we tested the association of SV number with sample purity (Supplementary Fig. 12), and found the variables to be uncorrelated (*R*^2^ = 0.001). We also tested the rate at which SVclone called single non-clonal clusters in PCAWG samples. Using a cutoff of <0.7 cluster CCF, and considering only samples with >10 variants (of the clustered variant type) the SV clustering reported one sample with single non-clonal clusters across 1220 PCAWG samples with (0.0008%), and the SNV clustering reported four samples across 1362 (0.0029%) samples. These results indicated that rates of under-clustering were low, and were similar across the SV and SNV clustering models.

We tested each PCAWG sample for the enrichment of balanced rearrangements (inversions and inter-chromosomal translocations) below the CCF cutoff (0.7) using a hypergeometric test, with the alternative hypothesis of P(X ≥ x), where ${{{\mathrm{x}}}} = \mathop{\sum}\limits_{j = 1}^J = 1[ {\kappa _j = \kappa _{bal}}]$ and *κ*_*j*_ refers to a given SV’s classification. Survival analysis was undertaken using the *survival* CRAN package (cran.r-project.org/package = survival). Hazard ratios were calculated using the Cox proportional hazards regression model, stratified by tier 4 tumour histology, age and the number of SVs in 1–100, 101–200 etc. bins. We used a hypergeometric test to determine whether any ICGC/TCGA contributors were overrepresented for SCNR samples within each histology type and found no evidence of any significant over-representation (using an FDR < 0.05 significance threshold). To determine whether SCNR samples were overrepresented for fold-back inversions (FBI) in the ovarian samples, we used a one-sided *t* test to compare SCNR samples to the high SV heterogeneity and other subsets, and found no significance (*p* = 0.8056 and *p* = 0.4671, respectively). Supplementary Fig. 6 shows a boxplot of amplified FBI fraction across the three subsets. For the clustering of breakpoints criteria, SVs were tested on a per-chromosome basis (inter-chromosomal SVs were removed). The ability to walk each derivative chromosome was tested using criteria for chromothripsis tests A and F^29^. Chromosomes were only tested if they contained at least four clonal and four subclonal rearrangements per chromosome.

A list of consensus coding driver genes was obtained from the curated PCAWG coding driver genes (29th of September 2016). Patient-centric coding point mutations were obtained from Sabarinathan et al. Table S2^40^. CCFs were matched using SVclone’s clustering results, and variants with a CCF < 0.7 were considered subclonal. Enrichment of driver genes was computed using a hypergeometric test for the SCNR and high SV heterogeneity cohorts using the driver mutations from all 1705 samples as the complete sample set. Copy numbers were obtained from the PCAWG annotated consensus clonal copy numbers (19th of January 2017). Loss of heterozygosity was defined as any region where the minor allele was zero (X and Y chromosomes in males were only considered in cases of complete loss). Copy-number gains or amplifications were not considered. SV driver hits were defined as any SV that affected at least one exon of a driver gene, and was not completely outside the gene (i.e. at least one SV break-end must fall within the gene body. Any regions with deletions (called as structural variants) that were also affected by copy-number loss (called from copy number) were considered as one variant only to avoid redundancy.

### Reporting summary

Further information on research design is available in the Nature Research Reporting Summary linked to this article.

## Supplementary information

Supplementary Information

Peer Review File

Reporting Summary

## Data Availability

In silico sample mixtures were generated from patient data derived from patient 001 from the Hong et al. study^23^. The data are available in the EGA Sequence Read Archive under accession EGAS00001000942. Somatic and germline variant calls, mutational signatures, subclonal reconstructions, transcript abundance, splice calls and other core data generated by the ICGC/TCGA Pan-cancer Analysis of Whole Genomes Consortium is described in ref ^20^ and available for download at https://dcc.icgc.org/releases/PCAWG. Additional information on accessing the data, including raw read files, can be found at https://docs.icgc.org/pcawg/data/. In accordance with the data access policies of the ICGC and TCGA projects, most molecular, clinical and specimen data are in an open tier which does not require access approval. To access potentially identification information, such as germline alleles and underlying sequencing data, researchers will need to apply to the TCGA Data Access Committee (DAC) via dbGaP (https://dbgap.ncbi.nlm.nih.gov/aa/wga.cgi?page=login) for access to the TCGA portion of the dataset, and to the ICGC Data Access Compliance Office (DACO; http://icgc.org/daco) for the ICGC portion. In addition, to access somatic single-nucleotide variants derived from TCGA donors, researchers will also need to obtain dbGaP authorisation. Derived datasets described specifically in this manuscript can be found at these locations: https://www.synapse.org/#!Synapse:syn7596712 (consensus SVs) https://www.synapse.org/#!Synapse:syn7357330 (consensus SNVs and INDELs) https://www.synapse.org/#!Synapse:syn8042880 (consensus copy-numbers) All the other data supporting the findings of this study are available within the article and its supplementary information files and from the corresponding author upon reasonable request. A reporting summary for this article is available as a Supplementary Information file.

## Code availability

The SVclone software, user documentation, and example data can be downloaded from https://github.com/mcmero/SVclone. Ccube clustering code can be found under https://github.com/keyuan/ccube. Code for generating all figures in the manuscript and the in silico mixture samples can be found under https://github.com/mcmero/SVclone_Rmarkdown. Code for simulating SVs can be found under https://github.com/mcmero/sv_simu_pipe.

The core computational pipelines used by the PCAWG Consortium for alignment, quality control and variant calling are available to the public at https://dockstore.org/search?search=pcawg under the GNU General Public License v3.0, which allows for reuse and distribution.
