# Supplementary material for: Inferring structural variant cancer cell fraction
Source: Nat Commun. 2020 Feb 5;11:730. doi: 10.1038/s41467-020-14351-8 (PMC7002525; doi:10.1038/s41467-020-14351-8)
Supplement: Supplementary file 1 — Supplementary Information [file 41467_2020_14351_MOESM1_ESM.pdf]

Inferring structural variant cancer cell fraction

Cmero et al.

Supplementary Figures

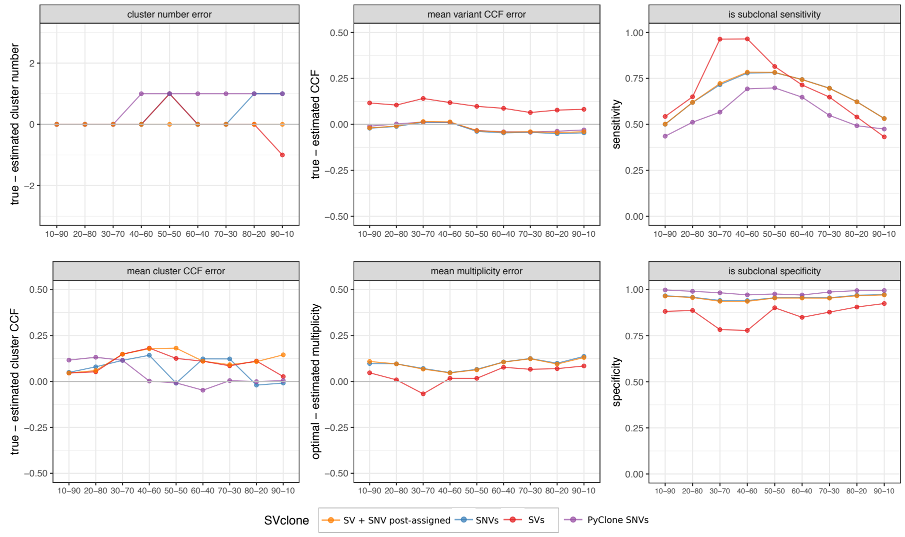

Supplementary Figure 1: Performance assessment of post assignment of variants. The first column shows the cluster number error (3 - inferred cluster number), and the mean CCF error, where true and inferred clusters are matched based on their order (see Methods). The second column shows the mean variant CCF and multiplicity error compared to the ground truth CCF. The third column shows the subclonal classification sensitivity and specificity using sample membership of the variant (i.e. a variant is classified as clonal if present in both samples of the mixture, and subclonal otherwise). Results are split into four categories: SNV and SVs post assigned by SVclone, SNVs post assigned by SVclone, SVs post assigned by SVclone, and SNVs post-assigned by Pyclone. Post assigning SVs and SNVs to a joint model yields improvements in mean variant CCF, multiplicity error and subclonal classification sensitivity and specificity. Utilising both SNV and SV data, the correct cluster number is inferred for the 50-50 mix.

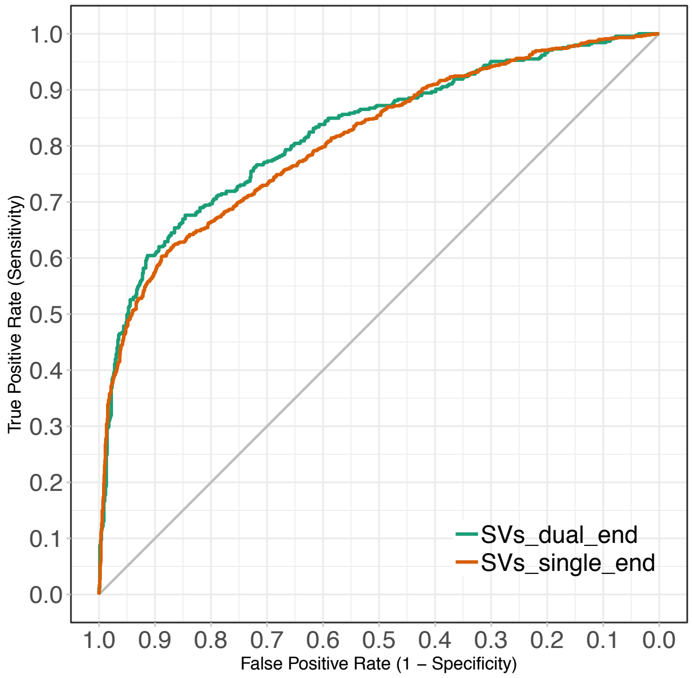

Supplementary Figure 2: Performance of subclonal classification of variants. The ROC curves shows classification of an SV as subclonal or clonal for the dual-end and single-end models, using the 3-cluster *in silico* mixture data. The respective AUCs for the dual and single-end models are 0.8234 and 0.8095.

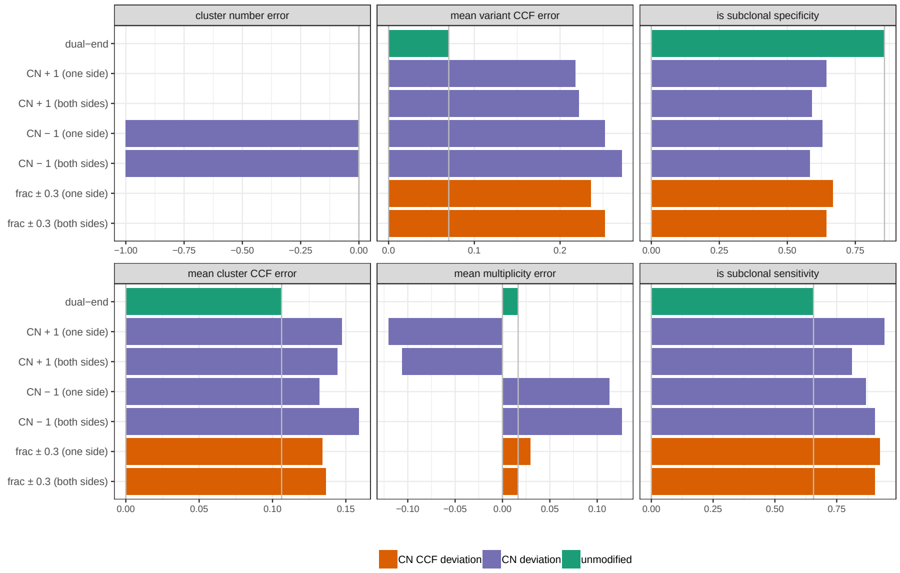

Supplementary Figure 3: Assessment of the effect of background copy-number perturbation. Comparison of clustering metrics in the unmodified dual-end model under various background copy-number perturbations (+1 and -1 from major allele copy-number, and +0.3 or -0.3 from the copy-number fraction for subclonal copy-numbers). Metrics for cluster number error, mean cluster CCF error and mean variant CCF error are all measured as the true minus estimated value. Multiplicity error is measured as the ‘optimal’ minus estimated value.

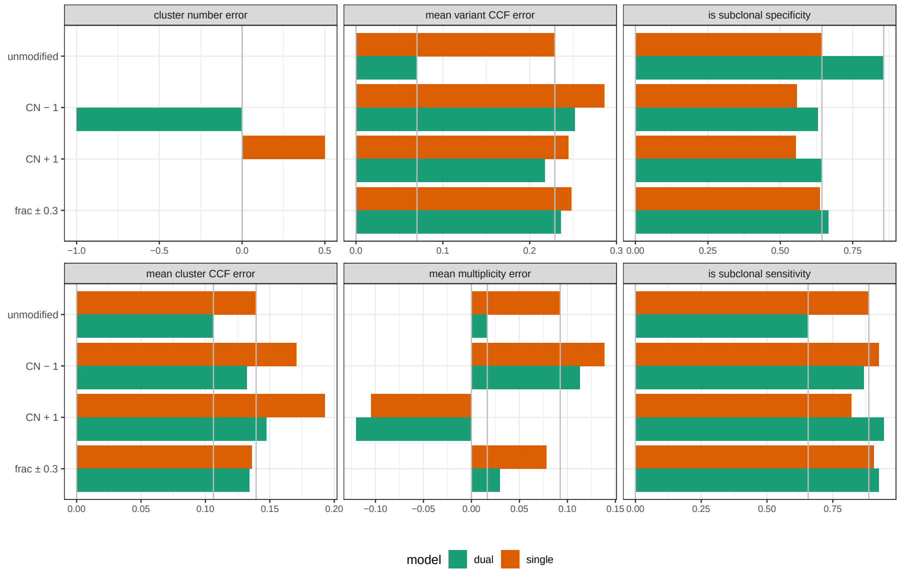

Supplementary Figure 4: Assessment of the effect of copy-number perturbations on the dual versus single-end models. Metrics for the single-end model are averaged across runs from the l and u sides (which were run separately). The dual-end model perturbations were perturbed on the l side only. Metrics are the described in Supplementary Figure 3.

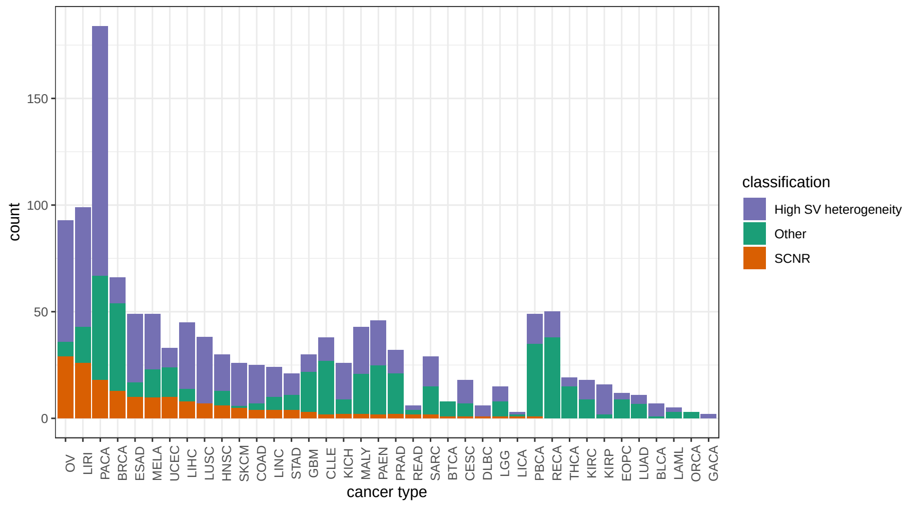

Supplementary Figure 5: Number of samples per histotype classified as SCNR, high fraction of SV subclonality, or other.

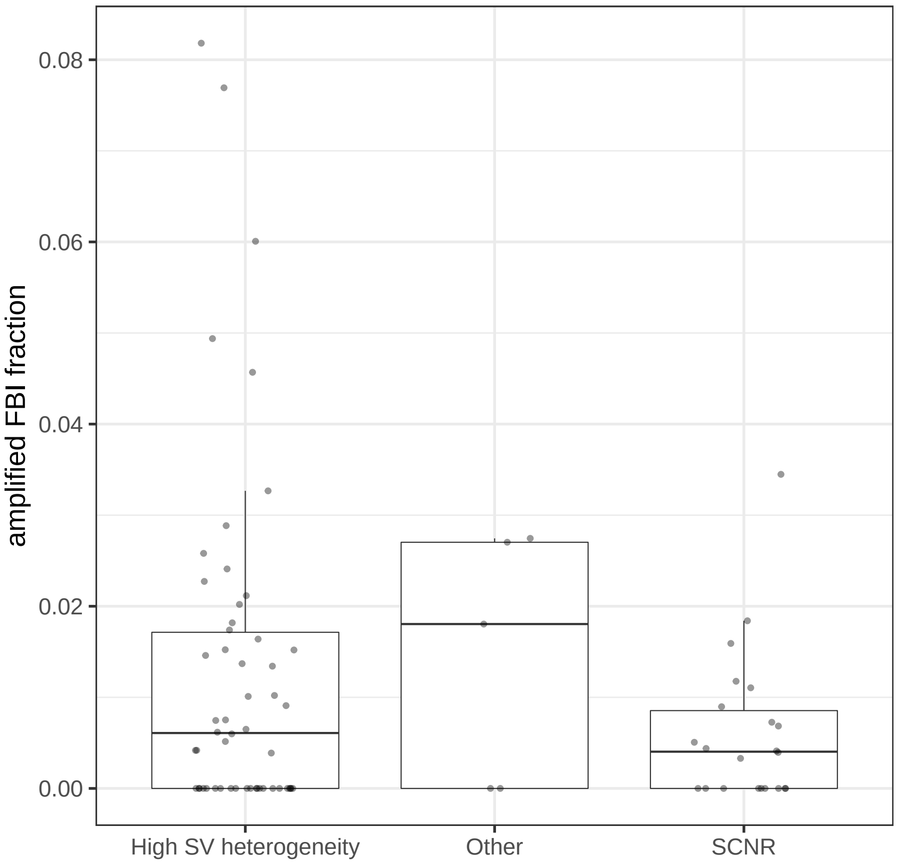

Supplementary Figure 6: Fold-back inversion (FBI) fraction for high-SV heterogeneity, other and SCNR phenotypes. No correlation between FBI and the SCNR phenotype is apparent.

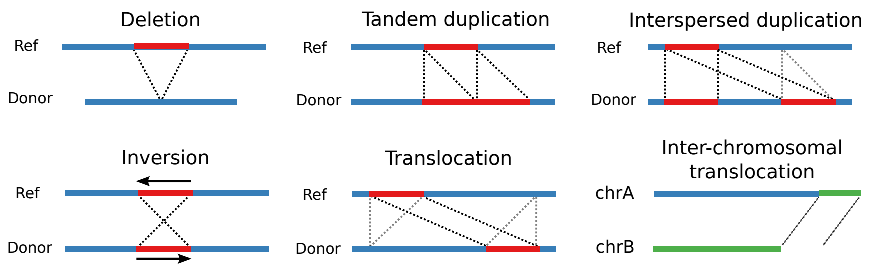

Supplementary Figure 7: A schematic of the classes of structural variation identified by SVclone. Ref denotes the reference genome and donor to the modified genome.

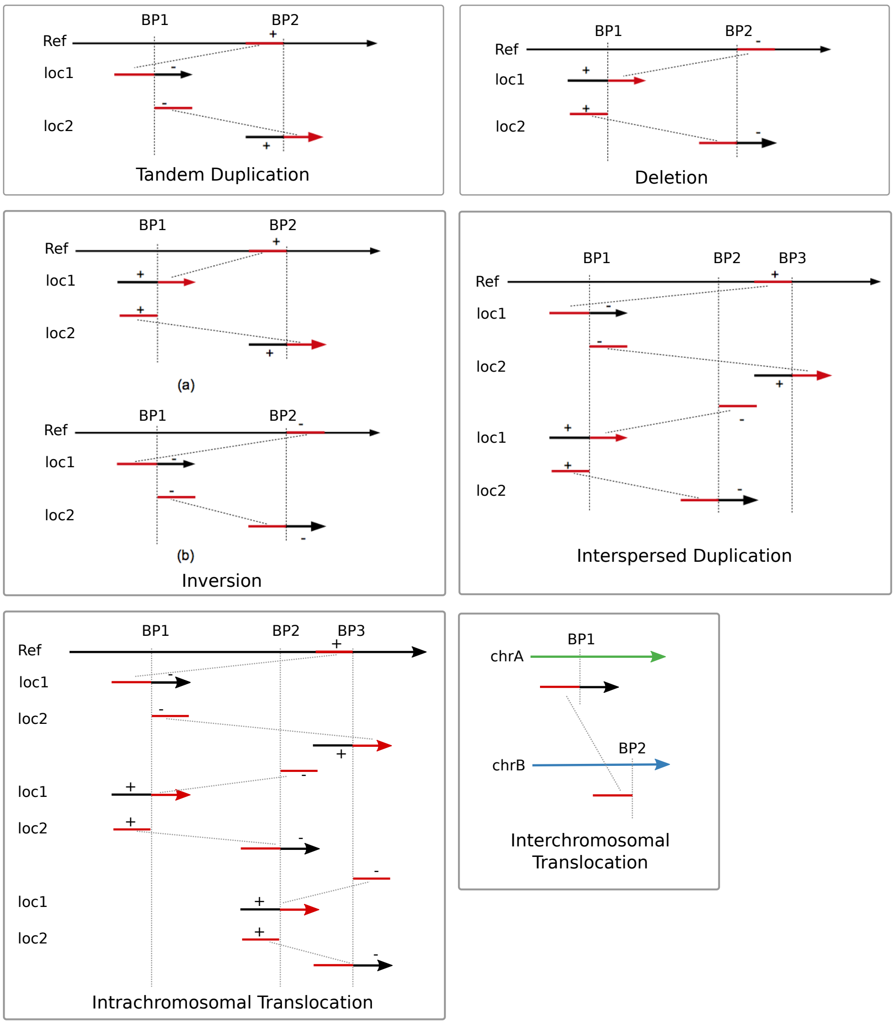

Supplementary Figure 8: SV patterns used by SVclone to classify SV events. SVs are classified from one or more breakpoints that are part of the same SV event. Tandem duplications and deletions consist of one breakpoint, where the directionality of $(-,+)$ (where $-$ indicates that the break occurs on the left side, and $+$ the right) for duplications and $(+,-)$ for deletions. Inversions consist of two breakpoints with $(+,+)$ and $(-,-)$ directionality. Interspersed duplications consist of two breakpoints and 3 break-points with a duplication pattern at breakpoints BP1 and BP3, and a deletion pattern at breakpoints BP1 and BP2. Intra-chromosomal translocations consist of 3 breakpoints consisting of an interspersed duplication pattern, in addition to a deletion at BP2 and BP3. We consider any breakpoints where BP1 and BP2 lie on different chromosomes to be inter-chromosomal translocations.

Break-end 1	
split reads	1
spanning reads	2
normal reads	2
Raw VAF	0.6

Break-end 2	
split reads	1
spanning reads	2
normal reads	3
Raw VAF	0.5

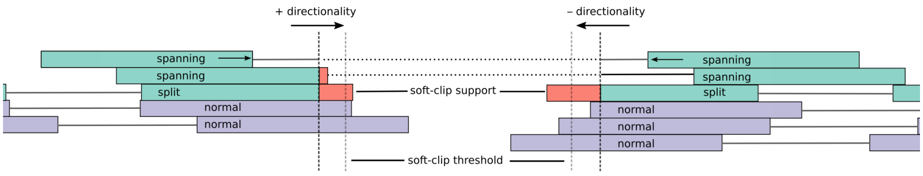

tumour reads
normal reads

Read counting criteria

Read	Crosses break	SC > threshold	Facing break	Valid insert
Spanning	-	N	Y	Y
Split	Y	Y	-	Y
Normal	Y	N	-	Y

Supplementary Figure 9: Schematic showing how reads align across SV breakpoints. Reads crossing SV break-end loci are counted as supporting split reads, supporting spanning reads, normal reads or are skipped and considered anomalous. All supporting and normal reads must have a valid insert size. Insert sizes for discordant/spanning reads are calculated as the summed distance to each break-end. Split reads must be soft-clipped above the soft-clip threshold and cross the break. Normal reads must cross the break but not be soft-clipped. Spanning reads must also be oriented towards each break-end. Reads crossing the break but failing to pass the supporting or normal criteria are considered anomalous.

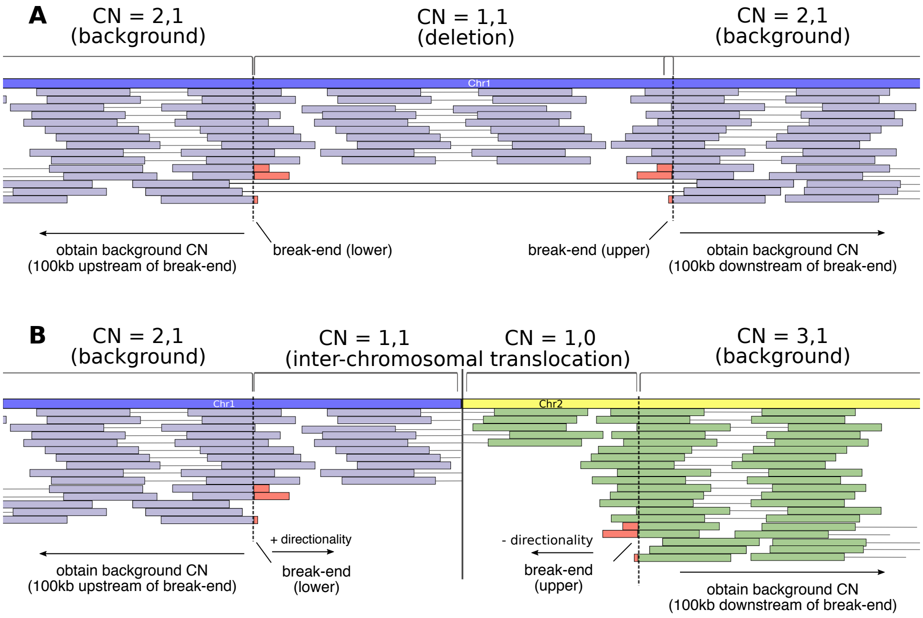

Supplementary Figure 10: Schematic showing an example for how background copy numbers are inferred for (**A**) intra-chromosomal variants (upstream of the lower SV break-end and downstream of the upper SV breakpoint), and (**B**) inter-chromosomal translocations (upstream for + directionality break-ends and upstream for − directionality break-ends).

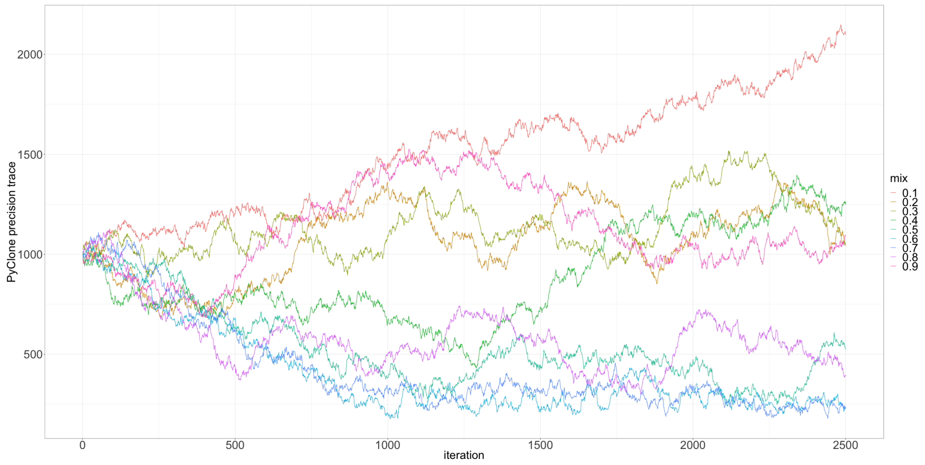

Supplementary Figure 11: PyClone precision traces for the three-cluster *in silico* mixtures. The large variance of the trace values and lack of clear convergence shows the difficulty of estimating dispersion in moderate coverage ($\tilde{6}$0x) and purity ($\tilde{5}$0%) data.

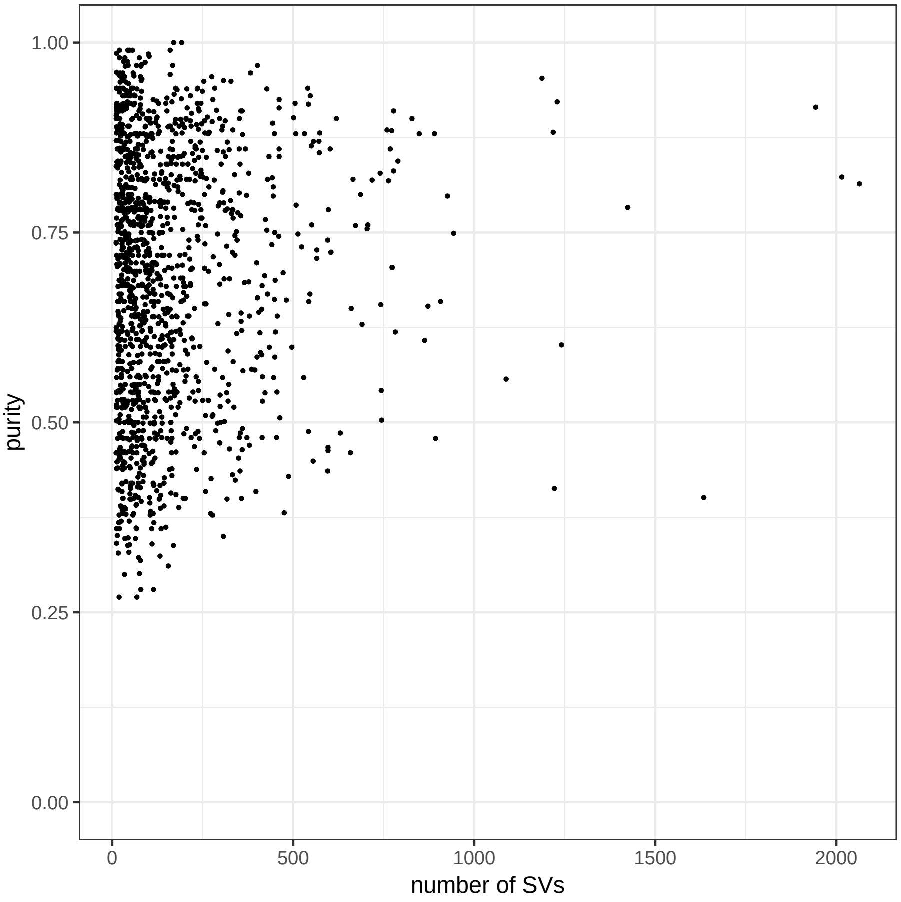

Supplementary Figure 12: Number of SVs versus purity for 2436 PCAWG samples with > 0 SVs, showing that SV number is uncorrelated with purity ($R^2 = 0.0001$).

1 Supplementary Tables

Supplementary Table 1: PCAWG SV vs. SNV statistics per histology group. High SV heterogeneity is defined as > 50% subclonal SVs. SVs with different background copy-number states at their two ends are considered to have heterogeneous CN states. Mean and median values were calculated across the whole cohort.

Histology abbreviation	Samples	High SV het. (samp.)	High SV het. (prop.)	Median Het. CN fraction	Mean # of SVs	Mean # of SNVs
Lung-SCC	37	37	1	0.65	176	46045
Ovary-AdenoCA	93	86	0.92	0.52	217	8674
ColoRect-AdenoCA	26	24	0.92	0.49	63	112812
Liver-HCC	156	133	0.85	0.5	76	13243
Eso-AdenoCA	48	41	0.85	0.62	204	32317
Skin-Melanoma	75	61	0.81	0.58	149	107468
Bone-Osteosarc	18	14	0.78	0.75	234	5878
Panc-AdenoCA	179	132	0.74	0.61	84	6820
Lymph-BNHL	26	19	0.73	0.35	55	15163
Head-SCC	33	23	0.7	0.48	92	18407
Stomach-AdenoCA	23	16	0.7	0.42	129	19605
Uterus-AdenoCA	26	17	0.65	0.55	172	10043
CNS-Medullo	17	10	0.59	0.61	26	1836
Panc-Endocrine	21	12	0.57	0.38	40	4234
Breast-AdenoCA	98	40	0.41	0.61	242	7108
Prost-AdenoCA	123	48	0.39	0.4	93	3576
CNS-GBM	29	11	0.38	0.57	93	15293
Lung-AdenoCA	11	4	0.36	0.56	104	28624
Kidney-RCC.clearcell	33	8	0.24	0.55	57	7223
Biliary-AdenoCA	13	3	0.23	0.64	84	24829
**TOTAL**	1085	739	0.68			
**MEAN**				0.52	125	20956
**MEDIAN**				0.53	76	7425

Supplementary Table 2: SCNR SV clustering and linkage statistics vs. non-SCNR, non-SV-enriched cohort.

	**SCNR**	**Other**
**Clustered SVs (all)**	0.9704	0.8712
**Clonal clustered SVs**	0.2694	0.1649
**Subclonal clustered SVs**	0.3911	0.3132
**Chromosomes walkable**	0.02734	0.0209
**Samples with clustered SVs linked by INTRXs**	0.5031	0.2159

Supplementary Table 3: Variants involved in candidate bi-allelic hits of driver genes in the high SV heterogeneity (HSVH) and subclonal neutral rearrangement (SCNR) cohorts. Percentages indicate fraction of cohort affected by at least one such event (N = 650 for HSVH and N = 177 for SCNR). Hits columns indicate the number of clonal and subclonal genes affected per cohort by the variant type.

**Variant**	**Cohort**	**Samples affected by clonal var**	**Samples affected by subcl. var**	**Clonal hits**	**Subcl. hits**
Balanced SV	HSVH	280 (43.08%)	209 (32.15%)	554	344
Unbalanced SV	HSVH	377 (58%)	243 (37.38%)	963	487
SNV/INDEL	HSVH	511 (78.62%)	100 (15.38%)	1084	124
(clonal) LoH	HSVH	634 (97.54%)	–	86535	–
Balanced SV	SCNR	118 (66.67%)	110 (62.15%)	318	238
Unbalanced SV	SCNR	156 (88.14%)	97 (54.8%)	773	242
SNV/INDEL	SCNR	138 (77.97%)	18 (10.17%)	275	20
(clonal) LoH	SCNR	177 (100%)	–	28067	–

Supplementary Table 4: Directionality assignment summary. # SV pairs is the total number of SV breakpoints with matching loci, # matching is the number of loci (of 2) per break-end that match. Ranks considers how the SV breakpoints rank against each other by locus on the same chromosome. SV1-3 show the outcome assignments. D indicates the original break-end direction; * indicates a new SV breakpoint is created.

# Mixed	# SV pairs	# Matching # matching break-ends	Break-end ranks	SV1	SV2	SV3
1	–	–	–	$(D, -)$	$(D, +)$*	–
2	1	0	–	$(-, -)$	$(+, +)$*	–
2	2	2	–	$(-, -)$	$(+, +)$	–
2	3	1	$[(1, 2), (2, 3), (1, 3)]$	$(+, -)$	$(+, -)$	$(-, +)$

Supplementary Table 5: SV offsets for matching SCNA states to SV loci. l and u represent two break-ends of a breakpoint, where $l < u$ (intra-chromosomal rearrangements, in inter-chromosomal rearrangements l is break-end of the lower chromosome) and dir_l and dir_u representing the directionality of each respective break-end ($dir_x \in \{+, -\}$); f is a pre-specified offset (100kb by default).

Translocation type	l offset	u offset
inter-chromosomal	$l - (dir_l \cdot f)$	$u - (dir_u \cdot f)$
intra-chromosomal	$l - f$	$u + f$

Supplementary Table 6: Fraction of SVs where background copy-numbers vary between ends in 001bM, 001gM and all 3-cluster mixtures.

**Mixture**	**CN heterogeneity**
001bM	0.31
001gM	0.38
10-90	0.26
20-80	0.25
30-70	0.27
40-60	0.25
50-50	0.25
60-40	0.24
70-30	0.24
80-20	0.25
90-10	0.25

2 Supplementary Notes

Supplementary Note 1

SVclone was run on the following PCAWG samples (all these samples were multi-sample representative and had a number of reads per chromosome copy of at least 10):

```
003819bc-c415-4e76-887c-931d60ed39e7
008aef39-0c97-48ce-9dfd-f12d67116c59
008bad10-d41b-4bbb-86fa-9976ecea46b1
00db1b95-8ca3-4cc4-bb46-6b8c8019a7c7
0385961e-ea99-40b2-ad79-6872bc30d8a1
05da3161-1444-4188-914d-19762de3a443
06ecd127-6c24-422d-a7fb-bf5aee1b8b7b
07f16397-71bb-4594-ad4d-caa7d2baeabd
089be98d-c3c9-4fb4-aef2-7a8578a91d51
0972bfcf-c6c6-48cc-b820-cdfa6279a4f3
097a7d36-905b-72be-e050-11ac0d482c9a
0a6be23a-d5a0-4e95-ada2-a61b2b5d9485
0bfd1043-70fb-d2dc-e050-11ac0c4860cb
0bfd1043-8170-e3e4-e050-11ac0c4860c5
0bfd1043-8177-e3e4-e050-11ac0c4860c5
0bfd1043-8181-e3e4-e050-11ac0c4860c5
0bfd1043-8183-e3e4-e050-11ac0c4860c5
0bfd1043-8187-e3e4-e050-11ac0c4860c5
0bfd1068-3fc5-a95b-e050-11ac0c4860c3
0bfd1068-3fcf-a95b-e050-11ac0c4860c3
0bfd1068-3fd8-a95b-e050-11ac0c4860c3
0c8f7e63-7a3e-42e8-9408-c5cbd2f52f9d
108749d2-5c62-4ef1-92df-aec6941ba53b
108b67d4-5d66-46da-8675-6acae643b76f
11473a98-d953-45f9-abf5-709b68a4bae8
1193a9c4-5aab-4cd7-a690-60c96bd1172d
120f01d1-8884-4aca-a1cb-36b207b2aa3a
122bf0a9-8052-49da-a9d7-0e5de9ab7032
134c9a92-e91e-4347-a9c4-727279edebb1
140d6ef7-0217-4d16-9add-215d0d14ba06
141918cd-3e23-45bc-96e1-b208c80f69e7
142b6dbf-c943-4a7d-8ab6-13a975f48d7a
145f2b89-8878-4390-b0f6-f09b02fb138a
145f6dbb-9744-44f7-8485-e440689de3fd
14c5b81d-da49-4db1-9834-77711c2b1d38
15563a10-47a8-401d-a507-2c7101f7ae60
16d33e09-2e21-4da2-8e57-e78ce28c4408
17a66d24-de77-4f2a-a8ad-d2c6a69e7671
183b04f5-107f-496c-8e9a-8263dd00cffa
19233fd1-5229-466e-acf3-5882165758e0
1924d200-6929-4eef-8a7f-317f8fbab915
19cd4360-8392-4bc2-ae88-fdc1335d886b
```

19def402-fb8d-4b27-90dd-ce9135a3c0a2
1a319682-f55e-4e0b-8476-48a0bea6c265
1c300960-e51c-4477-8fdd-026c7e545dc4
1cd0acf2-3116-4dfa-a063-0a435b9f6da3
1d325d84-a3a0-4802-9842-76cb21cecb1c
1d91f9c7-67ba-4606-9f0a-01ec6fc08262
228fb827-c05e-494c-8a21-e1d925e100cb
28839c75-90a8-493f-b658-8c63e0ebd324
289790a5-77bd-49a9-a1ec-478a8ecacd7f
2b41746a-95c0-4875-afb5-c6d3d3ad30d9
2b78de4e-4c8b-4adf-a058-3dae797e7881
2bd9ccca-3fae-4b66-a762-6f30d6276222
2c9c50ff-01d9-4703-931b-04119178706f
2e76891c-b620-4cc0-9315-6f1217b09b1e
2ea2294d-fab9-43ae-a222-370487495b06
305a5074-5384-45a0-985d-289b178d09f6
31bc44b9-35ff-43fd-8a01-a834f3b1ce46
32d8c373-b5c8-420b-9808-8812b5501649
335c721a-3653-471f-b0ec-6144fe868c6a
33bcd589-80db-4a58-8f83-a48cb60e0fda
33bf46dd-16b3-49c6-80d7-76caf27aa0f4
34ab4c57-5240-4af7-a329-a5ab55934fd4
35553150-e4ef-4539-b220-259f2d634bd7
368e592d-260c-4f58-81c4-a9f1e6177a4b
36962459-e81f-4b8c-a08d-df60ef3283a3
3933c60d-73d6-4f74-ae02-fd545fc1f092
3afb3f57-545a-4ed5-9461-2fcb28d89399
3b02a7a7-e5bc-4e77-b691-521603630b00
3b4ff158-c6d8-4a26-9568-0634ddb4a609
3b7810f7-f8ff-4d62-b766-3ba06170194c
3bb4c27b-4d0d-4d13-b910-41a9ebf95880
3e86e68b-d083-4450-b056-4cd0f8d4a084
41027c0b-85af-4c78-bba0-0da63cd75fa9
42f00950-09c9-461a-8423-d5b04a96af10
43206cb7-787d-4f68-8019-55e8585c4b4d
43b675e0-22e0-42d6-a060-afc93e22ac95
44083f54-0953-48e3-a704-11ad0988ad2e
46e166fe-3b20-49ad-98db-42c854c61c93
47d77750-c622-11e3-bf01-24c6515278c0
4aa7eabc-0419-4897-b7af-5dcccfd6ac2f
4c4aa1b1-fda3-4c5b-b588-68aa727500ad
4eac784b-084a-4106-9a76-2d9fbec57033
5042b417-d14e-42bb-b6f3-de303346a252
5187e77d-f412-4303-8049-11d1aa1a0235
51893d3f-e7f3-43f9-9fd0-c0f25ae96804
51e5a835-eb48-4640-87d0-f40f5e2bff18
526b3796-2cbd-4eec-8273-064b41456279
52f2d49e-f24d-41d2-9f73-33bd1cc51bba
532bec86-b8d3-41a1-ba17-913a7d378f66
54195db3-94a9-4538-8bb8-9953d936acd4

561fd34c-7c7d-4df0-bbfc-3d31147ca562
58e2453c-bb58-43a3-9642-705ec0e0bdbd
59938f45-3e57-43d6-af3b-bb4968249e5a
5d9ff58c-5702-48fc-a66a-d1276327fd49
5ead73fe-1c34-48ed-b20d-89fc3c82dbd6
5ee1dda9-2e84-4617-af09-2ae02873b44d
603594e3-e427-494c-b9ee-e872d6629563
6218f366-d5d2-4289-a95e-eeedfae7523a
6239e340-d50c-45b1-a7ca-e1135671ae0f
62ac7c91-da99-4144-b927-e90528dcd8d4
631ab9d0-5400-400c-b0bc-c3e576bcfe06
64b80154-c623-11e3-bf01-24c6515278c0
66b4a1f3-480f-4050-96ac-920c1c080864
67a2e34f-454e-4253-8f22-262fc74d312c
681e2301-2057-40e4-ab63-934775d63faa
68b7fdb1-22b5-4152-9e69-a41151640cd8
6c8e9197-fd16-4fed-bfda-e349cab26314
6ca5c1bb-275b-4d05-948a-3c6c7d03fab9
6cfce053-bfd6-4ca0-b74b-b2e4549e4f1f
6d0851ba-271a-408f-bcc3-c104b452c9e5
6d936ef9-b5df-44d3-831f-528bf8ddc131
6f25ca85-4b4a-420f-b0cc-5a4f65a97da3
7117854b-6f0a-4a43-b66c-506a3e29377d
712ba532-fb1a-43fa-a356-b446b509ceb7
715f59dd-a80f-422d-988c-f73f4b987baf
7181dd10-1371-4cb6-9219-a9542c62b854
72f82fbd-9838-4082-b605-bc3d80226f16
74b57d2c-a83f-4798-b86b-a533975e4cc0
759d30b2-ddb8-49e2-a604-57ff9222d3a0
759e20be-6a2b-4c54-aca0-6d358598d6f5
767517f2-f94c-44bb-9af6-36b662b166ef
77bf9514-88da-473b-9a91-f669ef3cd343
7a6cb147-a576-446d-a605-fa846ea3d1ad
7a95af21-ca7c-4596-9c83-66d11ca0c417
7c5c2293-3f40-4ae3-8346-6a327b2abf4d
7d04f401-3505-410c-8def-0348ecd8aae2
7d6fb1a7-7742-4b8c-9903-abff706240b5
7dd2dc62-0eb4-4d45-86f1-e9e9377181ca
7ef9fd9b-d349-4ec3-ab4d-1a1d11c0204b
8689b53c-8883-479c-85f6-d13b86272b5d
873ed251-fa44-465f-99e0-c90a42ea13d9
887616c5-06a7-4e83-948c-3546202349fb
8a2580c3-9c89-4cb0-b94d-3eca15dac1c3
8b0c4c91-2d07-4a9b-97a1-d2bda003437e
8c0a7dfa-6ec3-4262-b718-c3605722df37
8ca665f8-fe78-48bf-8c0f-c606d92885d4
8cf713ff-256b-4411-a1b4-5bf6f445b747
8fa7549d-d0a6-4216-9763-74b0f21c0fd7
9078333d-73d3-496a-9fc3-a94353b7e107
91b6fd6b-547a-47e4-8ecb-a6f1826ccfd3

924613ef-8732-4703-a387-136d37b2ba6b
924bcc4a-c982-43bf-8bbb-641dc983d65e
94652d14-2e4d-4f4a-a4f7-8df77df788c0
94cf1801-a720-4b2f-aab5-0b76c8ccb242
96cca60e-17dc-44c3-90f7-1b57b0e08e5f
9749a9a5-cb7f-4038-a5f9-5e2d6b97c689
97c1a300-978d-4df1-9e97-ce2f92b9eff1
97c46ede-b280-4344-8dbb-e860c6472239
99e7016a-c3c1-431c-8838-beb78a3cc017
9bb9164e-ea1b-49ab-bbcc-f59cf8c32345
9c00828e-e9ae-4b9c-959e-34837ee2b230
9de495d1-55b2-4535-9b0a-a999df35977c
9f298806-77b6-4c76-bd38-3343e1a3c6f0
a08c196e-4f96-4b16-94b4-aa6a0153ac50
a13ee768-1423-4fcc-b663-caaf6694acb3
a1af7c42-47af-435c-bb51-b932a3cd7b91
a3210fd0-344c-468e-8ff2-2d0869a2fb75
a6045753-60bb-4e65-bc89-1ef0b47aab35
a65bcd07-c47d-4bda-a2bf-21f68cef2f3b
a6d8467e-88e3-4ea5-8583-61c5ab9747ae
a6ebe0c0-8aab-4b9f-8328-4b795895a77d
a83e247a-8f65-4be4-9c6e-a72c4abffae8
a89a2341-263d-4e7f-87fa-18d9692d6b2b
ad92f17f-11e6-49bd-9835-6b2f47053485
add0b0f6-b4a8-4b73-b634-c3600dc567d5
aeeb01b4-790e-4929-bd59-6aad837603b1
aff5793b-3197-4d1d-bf0a-9b0ded5f2937
b1753e9c-29d0-4955-89d2-949cdabd1211
b2afbcba-d67d-43f1-be3b-05f3a5836ec0
b30dfb8b-8288-4e5a-afc2-3d5bd7bfa26c
b3befa40-8f44-4eb6-ada0-ec395f460656
b41c63e8-bb85-4f83-9bc6-e611fd9ef075
b5f90cb8-7304-48fb-a1d3-ff459c7d79d1
b8270565-9e02-4d46-9534-08206bfd027a
ba67c02a-180b-43f3-b53c-2b77d84fbe3c
bb659e7c-0b97-41b5-ac10-a66bb1f6f283
bb66787e-e0b2-45b6-b452-98a6c32132e7
bb91765e-4c55-4e7d-93e8-77face8c91f4
bc395326-1656-4ef2-bb19-0cb29194b91c
bc9f105f-6d99-44ac-9871-67defd5e85cd
bdc039d6-4817-4e6f-b8c6-a40b1e8e1a06
beafb7c6-ef39-478e-aa47-0f1ad39f95a3
c13fb736-614c-4d5f-83bf-2d7586f4fb53
c14b2c74-447b-4526-a6e9-415d2728c7ca
c5bc0826-8037-4fe6-ad4e-b1b675e771fb
c642b9cc-bdb1-4796-9692-8be92398be17
c75cc75a-7496-420f-b526-ea63c77e9839
ca004926-f3ac-4824-8ed5-d84e62efdfae
caf2e99d-8f1f-4043-b567-c3fe28dc33ac
cb753e6f-5ea3-4b58-9db3-7a62d15c8b2e

cc7af465-22ed-485d-a78c-282b840bf7c9
cd0c3b1f-c73a-4991-8724-4e74381900cb
cdf045a6-ab6a-4dc5-b0b0-d72fbb26b0bb
d1875ee6-6677-4117-a7a8-930ab5cde775
d1cebf4a-4127-47ee-85cb-465fca2ac445
d2620dab-4319-499a-b8f1-9575aaf8f4f3
d3b72600-a888-4ef6-ac5d-661c105405dd
d4c6061b-5019-4564-806d-4e75910a4690
d5423a93-0a72-43cf-a7ae-9011f47553c7
d707940e-0f9d-4da3-9380-90a422e44efd
d9e66fc5-9018-4568-b388-c5eb756f7823
da10f77f-4414-4125-b359-de596421cca6
dccddb92-bb26-4dc9-a935-2320d089a54a
deb9fbb6-656b-41ce-8299-554efc2379bd
decfb419-63b3-47b0-9dfc-baae180566a0
e1df938f-1f23-4291-98ef-13db6debb796
e4c8c7f0-5bac-4d59-91c4-c98207150548
e592e8db-b3d1-4ab5-ae0d-de4b5b4c0f1a
e6eda5db-4d4f-418e-b0d4-ed9b3e5259d3
eb1792ee-6946-4e0d-bdd1-c924328dba5d
eba6d269-27f8-4e50-b59a-5d639bdd5cff
ed7be9ae-e603-4731-8d91-a8285abaee33
eeddf701-93f9-4f10-85cb-9dce1760bae8
f1534b2a-5350-442c-bb9d-8c7142668d31
f1fdacf3-2bb5-400c-895d-ec7f472fa0d4
f269134a-e3d5-4020-bcf5-01fb42f914b2
f2e639cc-c30c-459f-8afc-7ace7d0209c5
f37971bd-ec65-4840-8d4f-678692cee695
f7cc504a-37c9-45f5-b50d-599c54dca157
f8a165b0-51df-413c-b7c6-c7fc80547db4
f8fae6e5-93b5-7233-e040-11ac0c4830e7
f9031d03-def1-24b4-e040-11ac0c486570
f92268fe-7a92-51e8-e040-11ac0d48733a
f938b6f4-a457-c624-e040-11ac0c486fad
f93abbbf-5e13-9b85-e040-11ac0d485718
f9c52187-2e82-d58a-e040-11ac0d484fc4
f9c6f4ca-4bb8-26b4-e040-11ac0d485600
f9c70e38-dd99-3fe2-e040-11ac0d4862f2
fa718a69-7d09-424b-90a3-4839ba7dc9b2
fab0be4b-c84a-45cd-a76f-44d9a8bf1846
fc93369d-3ffb-1b53-e040-11ac0c4852ba
fc9404ed-1ba3-2638-e040-11ac0c484da2
fc9d5fc6-719b-0525-e040-11ac0d485f83
fc9d93b6-92e8-acb7-e040-11ac0d487dee
ff870342-f0d6-4450-8f9c-344c046a0baf
ffad9288-c622-11e3-bf01-24c6515278c0
0009b464-b376-4fbc-8a56-da538269a02f
0040b1b6-b07a-4b6e-90ef-133523eaf412
00493087-9d9d-40ca-86d5-936f1b951c93
00508f2b-36bf-44fc-b66b-97e1f3e40bfa

005794f1-5a87-45b5-9811-83ddf6924568
005e85a3-3571-462d-8dc9-2babfc7ace21
007aab66-2f07-459d-8952-3041d6ea24a8
00aa769d-622c-433e-8a8a-63fb5c41ea42
00b9d0e6-69dc-4345-bffd-ce32880c8eef
00c27940-c623-11e3-bf01-24c6515278c0
00db4dc2-3ec7-4ff9-9233-d69c8c8a607f
01658141-8398-4585-9f0f-8355dd9b0604
0168a2a6-c3af-4d58-a51c-d33f0fc7876d
0176cf1d-0760-4769-a493-277f4bb7585e
0192d529-7340-45d8-a5f0-249cbb11ca19
01b0848c-ae36-4ade-a3da-d297b003c99f
01c8e465-2b4e-4519-bdef-c3ac06b43eeb
01dc6872-c623-11e3-bf01-24c6515278c0
01df36af-3617-40fc-9892-f54ce433cf71
020fab36-c7de-4933-b2bf-dc7b019a1326
0292a46f-a282-4b7f-a7d6-ac55cc7324fb
02c97e2b-914e-4afc-bf50-78f0cfbfa67b
02e5c36f-5bec-45e2-a048-875653b85ca1
030695f6-c623-11e3-bf01-24c6515278c0
0363fe2c-d765-450e-8b1a-d13a23d9af2a
03ad38a6-0902-4aaa-84a3-91ea88fa9883
03c3c692-8a86-4843-85ae-e045f0fa6f88
03c88506-d72e-4a44-a34e-a7f0564f1799
03ced0ce-186a-4349-8d98-572c2bc90382
04339769-517c-448d-a7ca-951f83608c60
0448206f-3ade-4087-b1a9-4fb2d14e1367
046d7386-95c8-4501-9e55-c85bec272a7a
047f9e4d-86b5-4943-aef5-68199bf29e8c
04b570c2-3224-4e9b-81cc-089b4a7ff07a
04c09376-8756-41d9-bbca-f464170a8cfb
05070acd-3ec1-44bb-9c32-26d3a483a72a
051cb117-16fd-459e-81e5-002eca2b7eea
052665d1-ab75-4f40-be5a-b88154c8beed
05486349-24a4-4931-bf5f-c9fefe369af2
0554ffe5-31f7-43f5-8372-2b73c9cf3527
05616329-e7ba-4efd-87b1-d79cd0f7af3d
0567d3e6-6278-4d0a-81ae-c084d73c6dd3
05c487aa-72d8-42e6-aa2b-b9b5ce273f5c
0609b182-0396-4755-b463-10f949c5b8bb
063cfacf-9933-4a2d-8921-0aeb76e12779
06dad93a-4b67-49b3-8fa5-f2546e22bae7
074fd614-5d9c-4bcc-96ad-351aa14387e3
079bcb09-a075-4313-9a3e-dd67734a8c5d
07b5141f-ac79-439b-883b-b0183bbe7ff5
07d20658-3db4-47e7-877b-66536266edfc
07e0e938-f61f-477a-92a2-5b7e74b78943
0831e45e-c623-11e3-bf01-24c6515278c0
08ac57ec-0036-4134-a9bb-f22eaa27ab0d
08b5d0e4-4661-460e-a9f7-f2e687414711

08e1d976-6c39-428e-a4c2-f655b675683e
09508a0d-ebe0-4fa1-b7b2-1710814181cd
0980e7fd-051d-45e9-9ca6-2baf073da4e8
09cb8bc5-13ac-44ac-9b7d-6de143373570
09e1fe3e-bfd8-4175-ac42-0e1bf0ba5523
0ab4d782-9a50-48b9-96e4-6ce42b2ea034
0ae2193f-0d68-485a-b8c2-7568cbcce33e
0b19bee7-5281-4915-9d98-c20eb3e84ecf
0b29c893-03bf-4131-b192-c14a2788d411
0b811c6a-8f05-44bd-ac33-fb720d189e71
0bafd9ae-64b3-492d-a6fd-12aa8edefcbe
0bbc7ede-5022-4084-925c-d65baaf7abc2
0c18a8b7-bad1-4aa4-a6df-472f89d9761c
0c39c03a-ec84-42c6-be69-8e98bc7a9f5a
0c9a5dbc-f7f3-43e7-83d1-77b9fb4b8b54
0cb4356c-b5d6-4541-b048-ff6d4b3a5d26
0cf9bbc2-cbd5-4b64-8d90-cfa416307b39
0d0793c1-df1b-4db1-ba36-adcb960cc0f5
0d259ac2-03d1-4814-9b0b-d05e3a6029b7
0d31a44f-1fc5-43a2-9633-36c4e650d3d2
0db9309e-7b95-4ae3-b0ed-dfd4f38d11b0
0dd39eca-c623-11e3-bf01-24c6515278c0
0e1e628b-823a-4861-aa95-bed2fada7349
0e6ca8dc-0f31-4675-95b1-5920576daeff
0e7ac212-0469-4f17-9432-205ab1831257
0e7f46ca-6f5c-4538-b6d6-00af65d57fcf
0e872e0d-4711-4364-a5d0-6beeb6fd3ff2
0ed2e2e1-2fe3-43eb-8cad-34f3f21a7169
0ed63d84-d3fe-4289-9255-35f4a03b703b
0ef92ff8-829f-425a-91d8-c594b6e22a2b
0f53e757-1823-409f-a0ea-249270728e15
0fa9cfba-0f49-47d0-ab54-9dd2144e0a73
0fbd94b1-bb34-4620-841b-861a0b5e0c12
1021b60d-f7b2-43b0-b2cc-f282d619d533
105a51c4-cc7e-4d0f-9cf8-e4d64a31d14d
10ad692b-4c3d-42de-9b5e-4968441388b3
10bb1a92-901e-4a14-80f4-5e88f997754b
1157a548-6770-4c89-a84c-bcf6f0e96ea8
1168944c-5451-4a18-8758-88bf5723bdd9
11ae45fb-05f6-425a-9295-d50d13407006
123c9d1c-72ba-4d82-8e57-d5caa52a40ba
126ee433-d345-4cac-882a-c91831a24690
127b0f7d-d24e-48b7-ac25-d3f14a43952d
12874085-a596-41ae-b4cd-817cb1ebb278
1292e13b-d7c6-447b-a227-9a8113215580
129459c1-38be-45da-bb87-18848c97c544
1295947c-c623-11e3-bf01-24c6515278c0
129de5b2-d9b0-4762-9ef8-72d98231fb50
12ae2ba1-9484-47fe-8a3f-bba94a6abe0c
12f038e1-00af-4c64-a2e0-9e63323492ef

13446fc4-9fa6-4bb3-a00a-b9265b41eb12
13642cf8-9840-4a7d-a26a-95f65b2c70c3
13c62975-9f5e-4d40-a461-dceab08a31ec
13c7065e-6780-4571-978e-a7c13078ff50
13d05f7a-e16a-40ef-9a15-21257da2cf89
140d5fa9-afbe-444e-a7e7-6a4cb4ab2923
14334aea-2dae-428c-9d4a-1a37878dfc3f
1447c8cb-25d4-4092-8919-4df08f898d2d
147e3496-514e-4da7-be42-7cd9be78ed04
148536ce-ee2a-4952-a19d-10d6f44146b9
14b8bbf2-310b-459b-b52d-a7ef510ce1cf
14cbcbb1-028b-471a-bb4f-8b29cfa5e4eb
14ed7388-41ed-43d4-afb2-04cd6410d5d2
154f80bd-984c-4792-bb89-20c4da0c08e0
155873b2-e5de-405f-87f6-6de0d238b160
15895218-c623-11e3-bf01-24c6515278c0
15a93657-0521-430a-a816-bf0842374b75
15d597de-d26c-4ce1-b0cb-077e39b55f30
15daadb0-6b24-4248-9225-470dc08f1552
15e7d981-8c27-4b2b-b4f8-626e22021895
15f90ef0-831b-40a3-98bd-ec226a9e8b26
15fd8dc8-c622-11e3-bf01-24c6515278c0
16c6f975-48fd-4cda-946a-777cb922949d
16df7888-2480-4394-8856-d57a6ef371d2
170ad241-9e24-469b-b7ca-3fd783a89b21
1776f326-b12f-4355-8107-80fa6fcd2159
178d0486-cb0d-4979-8bdf-bb3fe99149bd
17de5b12-c623-11e3-bf01-24c6515278c0
186cacaf-4f20-4ad2-a07a-a7dc942a70b0
18ec066e-8510-4921-9e35-45d85fb01e38
18f5e75e-c623-11e3-bf01-24c6515278c0
18f9240e-7acd-41eb-a154-c2a67a1cbe9b
19085f7d-e6a6-406f-bd09-520a27e0283d
190eb791-383d-4e27-870d-e470630843e7
199bbb0f-996c-40c1-b06d-2066f04be778
19aa8223-ee84-4b5c-b11a-6ca2a2680b3c
19b6b9d6-6912-4b85-a4bf-52014bcb170b
19c1c97f-a3ec-44a8-8a20-6f97caed1a4f
19d6cf34-1cd7-4242-a4d1-5d3e11f428aa
19fe27d8-c623-11e3-bf01-24c6515278c0
1a0ef226-41a5-4b56-9078-4b7834f0c976
1a5bf97e-3d49-4d3f-b385-ffa7a295be9c
1a841850-fca0-48ab-8c78-db0bd5a64435
1ac15380-04a2-42dd-8ade-28556a570e80
1ac54c6e-a1e4-4f3b-b4a5-cdb327e0dca6
1aff91a6-1b0f-4575-8f4b-4e064a50b886
1b0fe1d5-b286-4f49-ae92-94d9c6db9657
1b1780d5-06da-40ee-9e15-02631a68027b
1b7838c9-c685-41c3-b184-a2e40c7c4dc7
1bb2bdb7-8a89-4e7d-903d-03a6f8018efb

1bbb522d-e224-4b3f-9e10-3a47d06b2b05
1bbfe6f2-ad19-4d29-895b-973f5a91c707
1bd74f92-47f6-427e-af6f-299c3a7fe023
1c00925b-7328-4db0-b930-04aab2d80719
1c0e384f-7254-4afe-93c0-b3fc6c6a7894
1c10ab52-01a3-11e4-8395-af1f6b7ba88c
1c188bf5-2c99-4eb4-a774-59c75d53e643
1c28e44a-6e6c-44ed-b58a-e3262c0e6759
1c3df485-8e75-4378-87f6-c6463a520624
1cb25b72-3726-4f12-9e22-f5c7e4b05681
1d0617e8-2725-4411-b50f-e46ea1d43242
1d1eeba2-4d7b-4380-b0b6-26d246bc4158
1d4a091d-fe65-49c0-8810-5a95243b108a
1db83e80-bdf7-4d8d-8008-bd8406d641fc
1dc9e7fd-fc62-4b32-9619-4e02a266a385
1de83d45-8492-4b83-9055-a9f43b823f91
1e181878-c640-4e91-a620-3fc4b08a4de1
1e27cc8a-5394-4958-9af6-5ece1fe24516
1e2dcbcc-771c-43c5-8c8d-e0eb77cb3494
1e8ae8dd-74c1-44d5-a74e-bfdce801c736
1eb1bc62-18f6-4bcb-a16d-aa6a91914031
1f2a8889-4374-4037-bb56-0f20733380e3
1f8d8720-30ca-4057-90f7-a34b8383eafe
1f967003-82d6-4932-a445-24fc25723594
1ff155dd-deb4-44e0-b3a2-e4875b618435
1fff8b62-534b-4d71-a65f-e5f93b8b50ed
2009e5e7-1796-445b-8677-46b3804fe0bf
2045c788-9ea8-4ea5-a5e3-65fc16a62adb
207f8a42-5b05-4876-b0ae-ebfaeea27844
209a9b10-7129-48fe-a899-d14ba17efe6f
20d1b88b-3ff6-4201-a748-6a993500c652
20e02396-e676-412d-9724-44a428919cdb
2102558b-87b0-447b-bb9a-e4ae9df8b75e
21205681-a0c7-48d0-81d4-0b7ef31158cf
2182ce2c-5941-4b65-9419-fc7966d5e6d5
22578b9f-78dc-464c-9bb9-382f033283d0
2290b078-6a5b-4c83-9dfb-b525bbf14e4e
22d67778-61fc-4f15-95b8-7e7c6cc7112b
2321c412-7730-4694-bc8f-199d7e51a7f8
2399ab13-abfa-480e-9fda-7947edc420be
23cc70c4-b744-4fa0-a0de-b25c0fdf212f
23d86395-9e20-4cf9-bf7e-f76544a1f772
23eb72d8-c622-11e3-bf01-24c6515278c0
23fa90d5-b273-4e2c-9c88-68aa57b60e97
241abdfd-e6de-4830-a233-4bbc5f622725
2425a532-f562-423a-88f5-228642f53875
248fd0ed-f14c-40b8-9f14-e9c7adf16e22
24badf07-8615-48a3-8d6e-45c3d783f18d
24f887e1-ce82-40f3-9674-11102bd076c0
25103371-28ac-4f43-b0e2-2d376d0dcb84

25c76a8f-77c0-4650-bddf-45ed0c10a2e6
25cf7449-8c5b-403e-aa75-e2af158598e8
25e20393-752b-4796-9001-0e22ee04c586
25e4cad7-2403-4992-9cbc-433574673549
25e68b09-7d62-4449-a14a-8d09edd96db6
2660825c-68f0-4631-948e-6da158edbe9e
267a9862-17bd-4056-ba4a-1f373736ffbb
274723ac-2bc1-4e2c-b31a-1907217b9f84
278b2498-1d64-493b-ac43-3489ec86f313
2790b964-63e3-49aa-bf8c-9a00d3448c25
27b56bf2-7a9d-4061-98d4-61fe2761578c
27fcccdc-c622-11e3-bf01-24c6515278c0
28f41a20-b6d6-4ecc-888f-72f779ad9af7
28f8d45f-4763-472e-9c4d-6787ba00164b
29127cde-548f-4c42-96cf-6f0020c3db9a
293a2f0a-4846-4c7c-b8b1-bb741c8d3275
29578759-9ccb-4a24-b5b8-c45ebd4339d9
295aac88-c623-11e3-bf01-24c6515278c0
29a00d78-b9bb-4c6b-b142-d5b8bfa63455
29b92b51-7ba3-42a9-97d3-6a9b5e43f928
29fc38aa-f367-46a3-a14d-a5f657ad0caa
2a8d63eb-0174-4213-9214-413f391f512c
2abe2e53-ad9b-4fbc-b9a7-93444f1bea87
2b000af3-2c9d-4eaa-af3f-8101b7425c37
2b36ac4b-eed3-4591-8b4f-b44049dfc5ed
2b3837b3-ee29-4a08-848e-1206404da986
2b40a733-7a63-4bb8-a953-95a4ee28f962
2b4feb84-89e4-4c38-8561-5ffab02c8132
2b6d4d66-7f0b-4bc0-b3d6-171956a937c5
2bf5b018-9f19-4fbd-9e1f-7d958aabe5d1
2bff30d5-be79-4686-8164-7a7d9619d3c0
2c316b7e-6a82-4ba1-9f34-d8bfcf82d41e
2c3ada8b-9650-4859-9857-43209c0f7cea
2c48eefe-2a08-47bf-8e4d-cbaab6777150
2c6f1862-bb82-4e7e-9cb3-338bdf022ff4
2c9dc04b-e9ec-4cf1-ab2c-a18edb30dd37
2ccd028d-e7e0-4f77-a512-f658a31819a4
2cdc4cce-0877-4ec1-85f5-20c909912e65
2ce48f01-2f61-49d9-a56a-7438bf4a37d7
2d0e4b82-c623-11e3-bf01-24c6515278c0
2da2b745-068d-408c-9163-3b3a78d4aaed
2dd5eea1-42a1-4e8b-99ac-20399cc7e15c
2deb8f9a-9e0a-46d1-bcb5-902db3b5c8ac
2df17728-33e8-45ab-833b-ef68a5482daa
2dfe70fd-2650-49c0-b6eb-67df585d063b
2e43e0ca-54ea-482e-acf2-0048d9187a5c
2e8ad1cd-d5d5-4bb4-8bbd-e9bef45088da
2ecd8358-c622-11e3-bf01-24c6515278c0
2f0a660c-ba3c-49bb-90f7-39b0034cea82
2f2eaecc-6509-423f-b63a-8c3bea1ba4a4

2fdab641-d73b-4f9a-aa4c-c1944f131a69
2fe2433b-9a55-431f-a869-2b5a63e8e8fc
301d6ce3-4099-4c1d-8e50-c04b7ce91450
303abbe5-4155-4a0d-bc3b-f8995261ca52
304c3e10-583c-421d-ab01-15c937d5014a
30600994-c1ec-44c9-a620-3f6c617eb928
306ef5bc-97d0-4312-ae0e-343c4e31d869
30a8f292-c623-11e3-bf01-24c6515278c0
30dbe353-86d5-40ed-84c2-dbddf7beb17b
3166f1ae-678d-42ae-9d44-d3d25d6860c7
31c0c8f8-c623-11e3-bf01-24c6515278c0
31c75873-abb4-4d88-9e2f-07497a6c892d
31e63f89-a6a9-40fb-823d-f41587bd73d8
322f0b01-2118-4dbe-aba1-3875a54ee71b
3232f77f-b745-4232-a802-6699b6356efd
3269608c-5dd7-452a-976a-31ac971aac2f
32be40fa-c623-11e3-bf01-24c6515278c0
32fa6457-c8bd-4d26-8106-925baf13df68
33061bf5-9b99-4735-a810-f2d5eeef6873
33992441-4f34-42f2-ba87-6ddd227f2250
33b7e799-ad10-498f-9948-8ce433311539
33ea81f2-db2c-4567-bd7b-4cb9aadfef88
343fcbc2-f30c-4ffa-a0b3-f5bb21e5e70b
345a06d6-fa5c-4674-a847-88a6b537cf3c
34739bd6-b3be-441d-8cdb-e4233bf14080
34881c6b-f59f-42c0-9f29-9c5c47262ae6
34b0c838-bf6c-4d0f-8cd2-d2928adbb539
351db483-a70e-496d-b70a-7449875121ee
352fbbb4-88a5-4354-b1fa-3a01da3fbfa7
3585e133-b3c1-4d90-b5f2-2b867e0ae0ec
35a74e53-16ff-4764-8397-6a9b02dfe733
35b951c0-edb0-47c4-9076-ede346ea7cb1
35cca3b8-c623-11e3-bf01-24c6515278c0
35ceba07-0759-4fbe-b076-af821a528cf0
35dc84c1-476c-4d48-ae23-622bf6e043ac
35f82a33-bde2-47e2-8aba-ff1fd5920219
363d3a59-dce3-408d-a736-5c29ba9f9b6d
367fdef3-d7af-4996-932f-cc5087e843dc
369c06f2-8904-49cb-99d1-dd297ed0cd0c
36d1a85e-a09b-4537-86e0-eaf1eb03aed8
36e1d9cc-32ec-4a0a-8fb1-c46f058a6fb8
3728982b-4547-4249-bd42-72a91d3fda8c
374cbd87-428e-4509-85c1-b7d3302c30a0
37522f18-77b2-4414-8df8-3c2c8048adba
37c913e6-9ce5-4338-8849-b5bf7862d36a
387c6519-6529-4074-a5ab-00f8052a5732
388a8875-c3f5-494e-8456-28be8d3626e1
38d8e626-c622-11e3-bf01-24c6515278c0
3914c02e-44ad-4c96-8464-61aa95b42c49
39aee9fc-fa27-4b71-b9e8-43ff34bd3930

39c476fd-273b-4136-874c-53352f1be008

39c55051-e1bc-4081-962a-17205645de45

39c6c3b3-4683-4d11-984c-ed589188da65

39d38c06-3779-4ca4-b103-1d096f93dae8

3a49a936-c623-11e3-bf01-24c6515278c0

3a5eaed6-fcfd-437a-b61c-fd3725c64717

3a6bce45-0431-49d8-82df-b1d9a738e5a6

3a81f733-b3a3-4578-b925-a663612bc92c

3ace181a-23bd-424e-a87e-e0c01a2a2cb8

3ad44032-01fb-4f84-b880-f01bb13a4359

3ae3ce4a-54ec-4c3b-83f2-845696ea0fa1

3afae3fb-2d87-4724-a589-d31cce1f4c28

3b41cb48-c623-11e3-bf01-24c6515278c0

3b526846-72d6-4e10-b7cd-8cdb45a92a1c

3b55c58d-f95a-4a1d-9aa1-8d2c026656a9

3b590d75-ccfb-4da8-a2e9-10fb07e02cea

3bacc189-01b8-46cc-a442-f393c0f428c6

3baecfba-6977-4374-8bc9-22240abad9b5

3bb25b72-ceb1-4169-891e-339f22cb0f03

3bfbc33d-804b-4bb3-97ac-97563d77b13d

3c019b2f-52ec-40a8-99b5-98c1423ce627

3c2b1509-1eb9-4b79-9569-57810f291499

3c963890-6e79-4b16-a4aa-bac04938b4d7

3d400522-c623-11e3-bf01-24c6515278c0

3d477ac1-3bdf-4410-9594-1a3a1412c6ae

3da169e8-844a-4eee-b794-a2eb81db779a

3db6e6cc-1a06-49b9-834e-b6611cde4c4b

3de261ea-6478-4f96-8ca1-30be130e945f

3e1180a5-149c-4e6c-9a29-696e3ba567e3

3e604a1c-b95f-44ff-9723-e2fac845da3b

3e68233a-c623-11e3-bf01-24c6515278c0

3e7ccab5-5b1d-4147-b907-77cab8f0837e

3e94aa64-7dd3-4d63-a5de-b4050d3dfafa

3f039593-91d1-43cb-a06b-41e92f7e8d20

3f2ecbcd-5c9e-4d57-8aa5-962e2e86a83c

3f72f750-5666-44e7-acaf-a912d89475be

3feac02a-c99f-4dd9-9eff-e4f5e87f7dc5

4019c219-c51e-479f-8a9d-cfa6816ed696

40378b7a-a65d-4510-aaba-a34c4058fac7

408ed640-75f6-4346-bdd0-3b93c51adae5

40b1e928-c623-11e3-bf01-24c6515278c0

41840dc1-5ea2-4f01-a0d4-8b65add641c8

418e916b-7a4e-4fab-8616-15dcec4d79f8

41a7b031-d928-4a1f-891b-82fb3f6d548f

41c565a9-d44a-447f-85c0-98f23bfee1b3

41d50a3c-49fc-4d8a-9c25-bbbcd3a5da6f

41de507a-c623-11e3-bf01-24c6515278c0

41f140b8-72b9-4ae3-8b25-6144d9f94d0f

42465bbd-289b-4e96-98fe-76809c5e1520

42629ae0-9410-4fac-8df1-2c276467deb5

42af8f74-fd4b-486d-bc11-db53cc471d62
42de1441-6f3c-4b4d-b8b8-ad91e4b1dbe2
42e92d78-c623-11e3-bf01-24c6515278c0
42f88b95-fa12-47c7-93f1-cf72f207291c
436af904-65b2-4196-ad8c-a4e98081ee41
43aa4123-0fe2-4071-8a81-cbdd0410b917
43be43cb-a367-4a74-94e4-973acafcf576
43bf37e0-3dbc-4360-818c-fc1356d77495
43d630aa-3890-401c-bf56-e3a9f325734f
43dadc68-c623-11e3-bf01-24c6515278c0
441d2f21-b448-4fc8-8c54-9b85aca56237
44406493-37f4-48c7-961b-8714be50773a
446437de-c622-11e3-bf01-24c6515278c0
448af8b4-e071-48b0-a65b-b4ad17afdc0c
44eebc04-c027-45ae-beca-c4012b494f29
450e8eba-a3a9-4dcb-b423-e33dfcd5c34e
45348eff-4b09-4776-825e-b18de9dba937
45614404-2149-4468-848c-0796e3757d62
45aedf22-c622-11e3-bf01-24c6515278c0
45d0ccb2-641f-4348-b3a8-61f4113cd85b
45e16b70-c3ec-493e-86d1-505ffdf5056c
461df2ae-fcf1-4b93-be0a-c14954fe7c42
463bf060-8ba6-4a73-8173-172025f7b527
4652ae03-b096-42d0-bdfa-3f6281d4f023
46693a2b-5105-4770-a9e1-031dfedeb694
4679f37a-4f09-449b-a1d8-1f02847996da
467ef778-f481-44f3-9768-b9900252d2ba
468083cb-95c3-47c1-9d96-79bcf7bf3f26
46d35b82-e1b7-4d35-be5e-3a70fd47e421
46f19b5c-3eba-4b23-a1ab-9748090ca4e5
47050918-c623-11e3-bf01-24c6515278c0
4729fe4b-c1ac-489b-a67e-91e5c5e3ff8c
473fe833-fcc6-485e-93d2-95e662c3ef53
47b5d831-5287-4f62-b17a-6e5eff2e4184
47e5ff56-9534-4fcf-a593-33027e877f93
4808f9e1-452d-45eb-9b87-2f87c67d3452
483bb781-0179-42e1-bf9c-487b240769b8
484a41c8-fcaf-488d-97dc-bfe6a4b88a95
4853fd17-7214-4f0c-984b-1be0346ca4ab
48b15122-b1e9-4c30-8fc5-70a07686779a
48f68f73-3ac0-44ac-ba12-b8889fcb7f38
4904d56f-0fe7-4482-8f6d-9e54d261f399
492694a7-bb41-4128-a909-c424e7fc0c9e
492b4e58-12e2-47b2-bd47-28f8607072fa
493e7008-551c-4c0e-b567-a0f31868629e
494701ce-05d4-440b-a65e-d925902b9d42
49795e42-c623-11e3-bf01-24c6515278c0
49ad3c8d-88cd-43cd-a273-1623fe2aec0b
49b26ab5-2ca9-49b1-ae83-573fa41af2cd
49dec0c2-8e75-4f44-a253-82b2ea605890

49fa7e44-c86f-4b1f-8849-e5fe5b9de734
4a88981e-e409-440f-9bf0-71ca1bab8b4e
4adc846a-42cb-4ebd-a954-e31eb29d7572
4b5d9d8c-ff95-45f4-9287-2804e8a98ade
4b7a704b-e516-4275-be11-45cc935237e2
4b8c6b0c-0d0c-4834-a2f3-691e0919b902
4bae2f08-da75-4991-acb8-5ba9912f9131
4c027164-12de-456f-b933-6bc91f70b46f
4c183106-c622-11e3-bf01-24c6515278c0
4c2ad4a0-5d57-4e27-9f35-058b2f205f50
4c3840df-9824-40db-879e-6d24adc8c155
4c5228b5-bf31-4abd-a47c-d088e16dba13
4c8afa82-c623-11e3-bf01-24c6515278c0
4cbe411b-b05e-46bd-bea8-126289a0866c
4ceeb025-2f16-4f80-b9b4-0151346349c6
4cfb0aee-6661-478a-8694-9d8ba15e46b7
4cff8590-559e-4204-8635-96e11bfeda68
4d121008-0987-4142-a2b8-7dac58f3110a
4d1a4587-adb4-4a65-943e-f19efe8de142
4d2204f1-be84-4f58-b7e7-61ae9fbf6d25
4d409156-3a36-4c63-9ad6-b6af34f27b06
4d4eff82-ede0-47f1-b49a-ada025952114
4d690424-b015-46ae-885d-aa6d9031f66f
4d6bb438-86f0-4414-962f-2b1cebee4524
4d7f2c74-862b-4aad-98e1-fa831f14a905
4daa2708-d0db-40fe-a07a-763f003adcae
4dc9b032-7f7b-486d-a12b-8461af89501d
4de7441f-a609-4a75-9fe7-17a647079ac1
4e361622-f9a8-4e9b-a89e-19bafebe1d6a
4e7e6e1f-c648-446f-bdf6-0b1fcc6dfa83
4e8396f7-9506-4401-96b6-bb2e89557d59
4ecbfd89-393f-4126-aa1c-951a2c23ef89
4ef20a13-f107-4654-adca-fc60b8f5be47
4f3c2c94-fa7f-4945-9dff-e4d4dac95f56
4fdc8980-c623-11e3-bf01-24c6515278c0
50203cff-83f8-4884-8346-b91ef717d00e
5046f1b4-9d95-4841-ba30-691fa44719a2
504cdfc5-6721-43ef-b31e-fdf52f0cd9cd
507620ef-f0fc-4ac3-a7c0-ea2e36c40f77
50a5970d-a0cb-4251-8ab0-74ad0e94be9a
50b61288-1412-4104-92e6-8b1a359ef399
50eb58ba-c623-11e3-bf01-24c6515278c0
50ee360c-e3df-4888-9999-ebb88e271d08
513bafce-375c-49b0-ae75-6c607abd05d8
51458c86-0fdd-470e-b059-1ffbffc92a7f
5174f450-0bf7-4237-a53e-844064f0e701
51800588-c622-11e3-bf01-24c6515278c0
5193a482-6ed9-4504-bd7c-c8b9e4103577
51b25b37-f75c-4380-a0f6-5273e0b7ee33
51dd1a39-7349-442c-b7e1-7850fb800569

522dc834-8367-4f49-b93b-fcba91a2de7e
5255d645-d3d2-422e-8449-046cd21f4935
52f43b72-15ea-40a0-a3b4-4bf22bc0bf25
5310f698-d31a-4628-84b4-dc9ac29722a5
53534b3c-cd15-4d68-a9b1-6902bb234c45
53dec97d-0464-4ffd-8e2e-95b2b9a03af0
53e6a756-bdcc-4217-afe7-27d776159544
541f91bd-7e9d-4348-9e78-45b948d8967e
54319e3d-c28f-4ba1-9c87-299d303e4de3
54354d24-c622-11e3-bf01-24c6515278c0
54426b1a-e0d3-4d8e-9e81-2d8e6b153b22
546b97b0-ab82-42a1-bbd2-241523278ec3
546b982f-60d9-43df-9761-09369afbee2d
54842328-9432-4820-8976-7964bf9bf147
54cfd4b0-c623-11e3-bf01-24c6515278c0
54e78de0-d357-4125-a904-ab35e461572b
551d5082-ab6a-4674-af0d-ce408f589949
5533240d-8fed-440d-b781-6092785a77ce
55345803-83d4-4fdb-b0ee-328753801da1
554b9439-7e11-43ec-8bc3-448c59100b9e
559c30f2-8ae7-43c8-819c-800261065e06
55ada396-0878-4724-8ebe-00da367eed3d
55c82198-5be6-4cc5-b5b3-b707af234107
55e5497a-c623-11e3-bf01-24c6515278c0
56425c2e-6bbf-4b20-ba07-79a526dd4603
5650dc4e-8bce-4716-9199-cd5750d7a888
56681386-d407-4f53-ae4f-6c04437652f7
56cc8740-b132-4cbc-b513-f0b5b2305ca6
56d4ea48-c623-11e3-bf01-24c6515278c0
56ddcb96-e90b-4fce-aa4c-c677bbfee488
5702affd-eafe-42a4-8f56-c1f22f8f184d
5714b52d-7048-41b5-8141-bf42d960abe4
571ce258-b453-4cac-b05e-05755791be84
573ee34b-43a7-40d8-95ee-0ced4b46a7c8
576a1741-8c65-40da-9f0f-b2ca805d70f3
577d5c9e-fbda-41d5-b0b3-cdb733453ea5
578ba169-1d2f-4187-b174-082bbc6a5bd2
57947014-053e-4c34-b6a7-9e6f36f48010
589daa3f-0b76-4c9c-b494-048955e36052
589e0aea-9057-4b8c-8af1-74c74fe46a38
58c7e9c3-1345-48c0-a758-0887358b8696
58f4ff52-c623-11e3-bf01-24c6515278c0
58faf969-bf37-4180-8807-2f44f2cc8eda
59632e7e-c622-11e3-bf01-24c6515278c0
59c18694-8a43-41dd-a6f6-52a20bc8dd54
59e2d6d1-debd-4796-ab0c-6a5673a990fc
5a535c49-d42e-43c6-9d32-dc76f28d4f0f
5b178f44-c622-11e3-bf01-24c6515278c0
5b41e033-6df8-411e-a13b-59eeb8afbf9b
5b560f4c-d2a3-43fa-b394-abef78bdefc1

5b7d5767-2037-4175-aec9-886776343181
5bdca282-c671-48ff-b32b-2380996016c0
5bebc389-de0d-45b8-9d2f-6476988c358a
5c02d399-07af-4573-a568-bc1b256bc8f8
5c156f63-6537-4d93-a6c2-4155618cf638
5c4b369c-51a3-4ca7-8b9d-2a8e49c0dde7
5c8da06c-7964-4ad1-ac9e-d1b5507105d2
5c9b8a2c-c623-11e3-bf01-24c6515278c0
5cc7c966-499e-49e3-b509-4eeb00ee03d4
5cf21488-c622-11e3-bf01-24c6515278c0
5d6ad982-bb01-4233-b8fa-d129460eec79
5d922e48-aa70-454d-9417-c9af686feebc
5dbf3203-ce73-41e4-bf9a-32fc856f73f5
5deeaa45-4c1b-4ab2-a487-93c548838f68
5df489bc-6178-49e4-8a42-506f70300dff
5ecc88f7-8391-4168-af11-07a6bf9b3652
5f00a760-fbc0-41f0-b92b-b3c553eac0e5
5f096d7b-a1ea-4cc2-af73-10108089ae84
5f13e9b8-6c8e-4fd7-aef1-da4e5d5e2a5e
5f53bcda-2fd3-4574-9cc2-5efe353372b2
5f94cb62-4019-47ff-bf6a-eeda8e9e033c
5fa88c3a-dbc1-43f2-82b2-e0592f35ce57
5fd632ea-085e-4e9b-8dcc-ec94e492d3bf
5fd77ba9-5015-4d8b-86a0-582e5c76bdd6
60413de1-6cd2-4f74-8180-3bdd394d6d16
6051f40a-99e5-4461-9255-0e70d757b4d5
60525b38-c34b-479b-86d3-013c7e567b8f
606d7f5e-ff98-4245-a9ac-281f5074f97a
60aff7a0-4960-4ed1-922b-3233ccbfdba1
60e78c80-41a5-456a-9a81-9007d18fd72c
60f81dba-c623-11e3-bf01-24c6515278c0
616ff577-c5c3-432e-a857-f29901de119c
6172ca02-c622-11e3-bf01-24c6515278c0
61973578-4c0d-4a3f-b9c4-f96ceab24629
61cad26a-cbc6-4977-83b9-dc61e49f2861
61d941f1-87df-43af-8a29-b6809c809275
626c9788-d70c-449b-b9dc-5e3f6fc00991
6297aa77-37a0-4f46-987b-32bd8653c0c2
62fceb56-8c6e-4d20-9d2f-5b7042854d23
631eedb3-dbd1-4606-90e1-ea91b9a77a5a
637208b2-d8b6-45a3-a389-6f34db18fea4
63762458-902a-4329-a823-703b54cb5f9d
638e80c7-9a6e-4a32-a621-fc4168e72343
63a907ff-1042-4f9c-a385-55a32b3e103b
63adc09c-e1b1-40dd-9c35-2f8276b656fc
63d0c49d-918d-41fb-808a-1f8001981917
63db50d6-5ef2-44d0-9906-26eae74ecf44
63ea274b-d42b-4787-9c54-8ee95ea376ec
63fef3fe-c622-11e3-bf01-24c6515278c0
6400c324-2c94-477d-acb9-669d4ea46f1e

642e1379-1061-40bc-9a4c-f7c191e84d9d
64446aec-c125-4c81-b9cc-62482cb1aaed
644c669b-937b-4adc-81b9-6a1811cba9da
64623aaa-a274-4baa-8b71-958d9c8df733
649f2c20-5b50-40a8-a6fd-9a51f3d5b9df
64a17d5b-8f40-4ccf-8f2f-e68c5fe6b1ff
64c2e6a0-2341-49c4-a6dd-656e7bb505dd
64dd81e1-35fa-4579-8a4c-f7486adbb949
64f696a6-58b2-40a8-b848-fd775cd52529
654dba9f-cfe5-4121-97c7-868613cdef53
6551ec26-4166-4d4f-bee2-444ea2466978
65723119-bdfe-46f0-b629-c171023abd71
65d2dbc3-a163-4696-b246-47a430e66572
65f9a820-f026-4a10-8bb7-1fbc3b38321e
66701395-5a53-41bb-866b-3bbf08bc33ab
669f0e01-28f6-4ed8-bdb5-73f84ea28f78
66d312fc-809d-428b-98e3-a29d211db35c
66eb4833-1b87-4fd9-a53d-26dc7ad6de29
670f971f-a525-4ef8-91ba-610cfbaeb914
67189cd8-c623-11e3-bf01-24c6515278c0
6726c157-f688-491d-8b56-35628645df89
674819dc-c622-11e3-bf01-24c6515278c0
675a5a32-b405-4f03-bfcd-756343d1dfaf
6764e0a2-7a78-45c1-9189-6001c6518ce4
6797443c-eb4a-4654-b957-c0056e5a4206
67bb70b2-b6f8-4aa9-80ce-48829f9fec56
67f88cd9-ab77-4227-a8d7-5512246f9772
68054218-c623-11e3-bf01-24c6515278c0
6821446e-8e76-46fe-b0b9-47feb46d2585
6847e993-1414-4e6f-a2af-39ebe218dd7c
6867811f-ac89-47da-b5dc-1270033c36e7
68956108-2606-4696-b038-462b6c432398
68ba1105-569a-4209-9b45-0e2d64931806
68c2a355-862c-4657-b296-5776ed8447b0
692dfa4f-45e5-4183-b5da-6650a1fbcabd
6962bc97-fbb8-4220-a600-e3c251e245cb
69c3c88c-d51e-4e03-b96b-ec4f1a0faa2d
69dbe6ef-f5de-4c1f-b2f2-963dcb6ccd3e
69fe982d-6ab8-49b7-9efa-8349d60eb076
6a0d0c42-1884-4c6a-936a-678a822c0936
6a7cdaf6-211e-4eb8-a70e-1153d69c45e4
6aa00162-6294-4ce7-b6b7-0c3452e24cd6
6ad44218-d34e-4126-bf56-1be2140cd3fb
6b20f942-7b89-497c-8a2e-ccce74d20ca9
6b50391f-d890-4ca0-b942-beab0f8bf1c9
6ba5f81a-b7a0-4c18-a112-2e11094eec85
6baee028-c622-11e3-bf01-24c6515278c0
6bd4bca3-e568-4d1b-8b42-401fdf13d751
6bdf00f6-670f-466e-87fb-e853e41f000e
6c306cbe-c623-11e3-bf01-24c6515278c0

6c31d730-1734-44ac-92b3-760981528723
6c65cb6d-8c50-4c7c-bc84-eeebe09fbe01
6c884037-49f9-41c3-b0e5-9cbcd545aeb7
6ce42746-88aa-485f-a1c4-1759aad95914
6d3551d6-b5f4-4fd1-b8d7-8e5931096c19
6d3d5531-16b4-46ec-bd7e-394946855d62
6dac8ca0-f776-4ea2-85c4-aefba4966be7
6dc43234-4f07-4c53-8b07-8d78d9dbad3b
6de41ac1-229b-40b9-a494-5588c284351d
6e516c4b-fee7-4336-8470-494588e645b3
6e599fcc-d79a-4e0f-b451-c20768c989f9
6e6ad1a2-f1eb-44d1-9852-f5752afcf5eb
6e776415-ddf4-4060-953f-08ef22441234
6e839eaf-1dbb-43f5-8846-c980e05540c7
6ec4456e-c622-11e3-bf01-24c6515278c0
6f981023-4269-4e8e-a4ab-2c92bb27273c
6fc0cb07-9713-4355-8543-f85d1c5505c2
6fe135ef-54b4-4941-9bb9-872dc4d18d9f
70422e6d-cb1f-4284-8be9-1d4517ffad60
7087a2b6-7fc4-45b0-8c48-248864f03deb
70990332-7702-45e2-84cb-8bbff773f160
70c2f91e-c622-11e3-bf01-24c6515278c0
70dd8453-11d3-4105-a8d0-6a755c52495d
711c8a16-3cf8-42d8-b29e-fd1e9ef1c82b
7163a0f7-2c69-40b8-8857-8ab3f46f0acf
71982a1b-52a4-440f-bd5d-62a0e6cc7fd9
71a2b4a2-88dd-4911-a67e-70b3309c4a12
71ef8379-03a0-4b81-92c9-1ba666fe4d7a
724bf2b8-a246-4b6f-85f8-95824c03896e
7266ec7b-4824-4b88-910e-e9bfb848d55f
729229fe-92b9-439b-acd2-b49159116a41
72a27124-24b9-41f7-a065-774fdf5f7d19
72a5e569-99d4-47bc-be1b-6296a5025080
72b62805-76f9-476a-b876-a781e69a2c90
72f0a49a-aec8-47e5-846a-956c4da1507c
734a8251-9ecc-47f5-835f-846b67693ca6
7364a112-61b9-41c6-b85f-18c97c54cefd
73936e8b-c893-4afd-bd1f-be90c06a4869
73afad06-c623-11e3-bf01-24c6515278c0
7410c00d-ed0b-48dc-a4bf-eb76ac613980
745b8756-0eab-423f-8cde-e0ff1aaa6596
748d3ff3-8699-4519-8e0f-26b6a0581bff
74e5905f-6350-498a-9454-9ea0593d2f56
75ba6722-1148-4a52-a9ed-68d890238205
75cd90ef-e884-4812-bbf3-5a524d95147e
75fac59c-3346-4c5a-bb9f-fb67fec8d13b
760881cc-c623-11e3-bf01-24c6515278c0
769e2ac9-a121-4b2a-a0fd-5f755b579811
7731ef7f-25bd-42f5-9a20-be249306a711
77c022d3-2f29-49d3-a1df-cc2e9407fd7f

77ebe6d2-6537-49d3-849d-fe93e2cf52af
78100212-65aa-4365-8b64-4b33f77732d5
78103d9b-0b8a-431e-bb58-5c99fbea12e4
7815397b-aa39-4b79-bcaa-6859a3f115f8
783579bd-e29d-457c-8236-1a80b557600c
786fc3e4-e2bf-4914-9251-41c800ebb2fa
79169c46-7049-4576-9885-63973db76ab9
79353875-9d86-4063-a8ea-1d998acb1e81
796a8782-6d7e-47c8-b322-fcfe1dd240c8
79914ce9-04d8-4b0d-9f16-2e528044966e
79b23f34-c623-11e3-bf01-24c6515278c0
79e81ab3-5466-4324-9387-2de00d62315c
79eb7bba-f0d8-462c-add7-20a2fb7843e1
79fa8423-2014-4536-a1f9-cb48aed3c45f
7a4f6014-f7a2-459b-bc43-df164e73cbba
7a5d3b6b-be5a-4e55-9255-32a069b8af3d
7a921087-8e62-4a93-a757-fd8cdbe1eb8f
7ac0b303-d685-4fa5-bfec-34d2ba1dbc8e
7adcea71-1aed-450c-b0ff-b9d9820c6153
7ae3671f-bf98-4693-8f35-3b762c9121d4
7ae9b843-488f-459c-8c0d-c81dcae57f99
7b0b60c7-5fa0-440e-937f-8d82119330d6
7b296706-b535-45a0-9a8e-4a34a8c33bb5
7b318282-5df4-42bc-b1e0-0e4a66ba9204
7bc3209f-b349-49bf-9b3d-44ef870b586f
7bd3d112-d411-4ea8-a9a9-bd965f0fc943
7c405ca0-c622-11e3-bf01-24c6515278c0
7c43c006-d917-4915-b304-65995b6c0d46
7cae6c0b-36fe-411b-bbba-093a4c846d84
7cb71498-6ada-43c6-912b-bad4f93cf580
7ccb9a4d-6f48-41c2-a630-27fde8c67d60
7ccee959-1e87-4a39-b6b3-840ddcb94995
7cdbe0e8-f614-4f54-b864-fd6b39e8ef1c
7d2a22eb-7344-4cba-ad7d-94c3f9ef3d7c
7d2da7ba-f57c-4083-9572-6fa98008083b
7d332cb1-ba25-47e4-8bf8-d25e14f40d59
7d38018a-c622-11e3-bf01-24c6515278c0
7d59205a-65ec-4341-9748-28639d82ed29
7d734d06-f2b1-4924-a201-620ac8084c49
7dc3e035-c7ae-4164-a62b-9c2460812b05
7dc5f8ba-0080-43d3-8426-bd527a970761
7dfcfe29-3900-4d7b-8067-60c51584b0b1
7e22401d-f4cd-44c5-8a01-b08a439e5a31
7e305f31-fbb0-4bce-894b-1dd85e303421
7e4d570e-f26b-41fb-84c5-2a6f7d668bb5
7e584ff6-0b81-4f85-81da-b8117afee326
7eac4710-c622-11e3-bf01-24c6515278c0
7edc42d3-d08e-4360-a3e1-aeb57cfc6640
7f031d71-3cb7-4744-86bd-a3beecfe166e
7f22b0f7-4040-4c84-939e-894c3050ef5d

7f42e118-aab6-438a-9e84-e7451fe12d7e
7f5fd36f-6e30-45ca-92a5-d8a8a8ee1bc2
7f795952-c623-11e3-bf01-24c6515278c0
7f7e007e-4b06-4328-b116-6dcdb2ad8181
7fc7b316-f3d3-4e71-9417-423fe334e6bc
7fcd8af9-4d73-4c97-95cc-b30b09f21bb2
801e67c8-c506-410a-bdf0-856a66e974f4
804ffa2e-158b-447d-945c-707684134c87
8093649a-74d6-4832-9154-8cc0182fa381
80ebcecc-4a7a-40dd-914c-f0648a58095f
80f02aec-c07a-4bcb-b547-e60f8c33a7b1
81598359-24d5-4a8b-a6e6-7a6f94289119
819b4304-c622-11e3-bf01-24c6515278c0
81a8b064-e735-455f-b2db-af7ae11daac4
81b4dd02-34ec-4ddc-8818-ed888a60ad88
81bb13fc-f082-4c0b-8066-1729f11ef617
81bc7f0c-865d-4801-a935-2ab04170df53
8282283d-247a-431d-9421-0fcc52f0a897
82be6087-a491-42d4-9b48-6a10d1bace18
82d7fa2d-19cd-41b7-8ed1-ea9fb9a544c6
836ec5a5-e385-4236-83e7-2641ee35db7f
8404ef5b-7a64-47fe-98f9-3f63619d2d99
841eb82b-347d-4d7f-805f-3f3701a2983d
842df341-d34f-4ed6-928c-eaf15bf7f667
8454fe53-869d-41c8-b0c8-a7929d00eec3
84ae4dd8-f661-46aa-9892-69742e2f3542
84ca6ab0-9edc-4636-9d27-55cdba334d7d
84e601b7-dfa5-4cd5-9fef-07f03967a0d4
84fe77b4-f6cc-49bf-a6b9-1621ec9394f8
850389d4-c622-11e3-bf01-24c6515278c0
85422a8e-2599-4dab-a257-150e380ecba8
85448d11-9f84-44b7-8ae3-a115f24bdb76
857a8354-9589-424e-98f4-98b9bfd6dcb0
85d915b8-0184-4ec7-93ad-e9ffa2f5743b
85e29049-a5e8-49fa-a576-e113c7b2c2e3
85ed2434-da94-4333-90ca-6ca4691cbdd3
86386b36-2950-4479-b79f-4b347053c8bc
8658f4f5-9a50-4195-8ea3-227951977647
866ecfe7-caa6-4565-9418-6b9d6c8a3b43
86874a67-aa86-45f1-be32-3b2722ebedec
8691c87f-2468-44bb-a670-3dccd6da33c6
86ae34f9-e16a-4593-8e55-b1296782bc1f
86f23897-dba0-4e89-8381-d174eaa6fcc1
874f64e9-8a0d-4f88-92c4-7e831842b1d1
87671312-c622-11e3-bf01-24c6515278c0
878a7fe7-20ff-4651-9587-b4d6fd42e929
87e0b332-60f3-4d59-8c4e-691a820c9ee1
880e6970-0131-4966-b13e-8614d33f0df7
88395c0e-4afe-434d-8b20-5a3eef8b7f00
8845fce6-e2b4-4929-942b-57096b0bbafc

8853cbee-7931-49a6-b063-a806943a10ad
889fb525-5055-4408-a12e-17f1225c9d29
88bc38ba-ad1d-431e-a67e-0a5a23678386
88d5a8b2-daba-45ce-90bf-480fac85bf1d
88d82a42-c622-11e3-bf01-24c6515278c0
88e35d25-d98d-4576-8ebe-1cd74010ecd8
88f2c496-3eb4-4c03-b3a5-8d5a55803aaf
890e840c-1d1d-4874-a8eb-f9d9a2b50a1c
897a4500-6fb8-4e81-aa07-021a26d632fb
897ba508-d74b-46bf-8554-1210a66a36d1
89c48e53-c8a9-4518-b3b8-509cdcaff52b
89dad92e-5b3f-479a-a6da-a94ee7df7f8a
8a69f3ca-4e17-4daa-8722-a36316e345ba
8a929c55-35a6-4645-bb70-4b85d281b139
8b28f6d2-4b7d-493b-826e-b119a4fb0cb4
8bbe4006-be0a-4cd5-91f6-529100d4f06e
8be2b0f5-7bad-4720-b13c-e95b32bdb933
8be6b14d-286a-471b-a282-ab98bc6050c3
8c443bd0-7987-44de-b312-5b859e6d13a9
8c46a1df-1c10-4188-992a-a55b782494c9
8c4ebff2-8318-4046-98f5-da8f6b1a9e3b
8c5f9574-c622-11e3-bf01-24c6515278c0
8c619cbc-9e91-4716-9711-5236e55d8f46
8c850df7-d48e-4386-b3a5-cac5da865ea5
8cbd1752-7eea-46d3-8623-09e96dfb1fdf
8cf9b32d-3d6f-4898-8c7a-89511b754021
8d5ffe62-6dd9-4764-b0b7-5e3010eefe6d
8d67c121-37d0-4fc0-a349-2c9452589d65
8d7592e2-c622-11e3-bf01-24c6515278c0
8da641e4-9b12-4163-bd1e-9d5af91e5186
8dd14f0e-8601-4aa1-864c-3c49e768cdd1
8ddcf0d9-312f-4055-8984-55d463face34
8e03e773-5557-4e78-889b-4710c515378f
8e48aa43-6cd1-4aef-83ce-2451b1b86e8e
8e7fe8bb-6b3d-428f-a232-97e6042a0f39
8eb90ea4-c622-11e3-bf01-24c6515278c0
8efb116f-4ecf-4191-8ebf-90a94c1880cf
8f558713-f32b-403b-aedf-c79efeb41c67
8fa0484b-5f95-4ec8-b3de-cea2e8f3c5ca
8fa9575b-b2c9-4bd7-8da1-3c2cdf0e39f6
8fb7fcac-6c1d-40c2-9309-b53821cbef30
8fc11cb6-9d79-41c3-bb45-0dadda890e8d
8ff9b1b6-a498-47fe-a970-ab7cfb64e653
9011cf17-0783-4f9d-b355-4f0fc1a2e78b
9020b6a5-8325-4b0d-84d9-04371c857910
9032b7fe-e38a-4641-a45e-67041668adc4
90611290-e44c-438e-91aa-f3b83dae3b71
90883300-d4b6-45d7-af9a-4e4ce4104500
909f3c5d-89fc-419b-a654-75ac1dbb149f
90ab64be-2229-4a8e-b864-4260b6cb39e9

90b3c237-29a4-48b3-90b3-9f2c0ccb5928
90fd404b-f734-41ed-a601-0f30e43625fc
914421d2-898e-433e-bb66-b1710eaae2c7
915cbb43-9e00-433d-818f-531011bea57e
917564e4-c622-11e3-bf01-24c6515278c0
92126163-00c3-41f2-9ce6-7aad5f621407
926a04de-ef57-4271-91db-e90de8aa8a1a
9321341c-c622-11e3-bf01-24c6515278c0
935b113e-f5ed-4a07-8e1d-1603daba7f40
93a49462-4bd3-4efa-87a5-69006abba5a0
93b51c61-6eea-4228-a102-840a2e118522
93be2990-bceb-45fd-aca5-3358d96e9d5a
93ff786e-0165-4b02-8d27-806d422e93fc
94434272-4cbd-4b4d-bd98-44f18526dd69
945e6808-2887-4f32-ac32-8f9e00353bf6
94b5dc5a-701a-45e3-8f63-8231031a055a
950486ad-14f8-480a-b079-9cc3cd842090
9536f736-63bc-4099-bd54-740f5910f4a8
9563a264-c622-11e3-bf01-24c6515278c0
958d4d1d-25e2-43f2-a18b-59b2c66f0c05
959efb81-1d56-4659-9b7a-09f2b62483f2
95cc9620-7686-4805-a96c-cf8e659847c3
95e8d38a-122e-494c-a304-bef4275fed7e
95fc38ac-2b36-4c46-abbf-8d2d52ff9626
9623d295-c23d-4f2f-827c-69f767a26f1f
9650640f-154d-4696-aa96-3611c6fcee7b
96517058-18d2-4847-8085-768ca8aa1565
968929b0-6bfb-4a2c-bd4d-570bfcdb8a6a
96b67289-9c42-402b-8e30-9efb4d512bef
96ba50eb-3c12-41ad-ac7b-d23b2001fe44
96e3db14-2bb1-4f68-aed6-5e794750c96e
96e62134-b8cc-4931-9c99-1193448c7c93
96e99cf0-707e-49f3-9ec6-cb4f90ecf7e4
96ef40c3-8d4c-4fe1-a33a-99f5b205a0bb
96f337d2-cec4-4484-87cd-ab2109d2bf50
983c14b8-f35b-497b-9fd4-5e5f5f0a7aa7
987528ac-437a-4eb8-a335-4f2076d5c006
987d41ee-6bab-490c-b0f2-bf28c0089de9
9880c3c9-5685-42a7-8fe9-7585ea1a1d37
98bb3025-0637-4106-8621-12df7b5d662f
98d27916-c622-11e3-bf01-24c6515278c0
98f54445-b151-4aeb-b8cf-91d743ba066e
992d9566-b1da-421a-bf36-d23382b115fa
99696731-c88f-427b-975b-1d90013e63ff
9a5bb831-8cb8-4de0-b94b-088cb38def1a
9a90f951-ad96-4883-b97f-ac43395dae5d
9aac83e4-c622-11e3-bf01-24c6515278c0
9ae0744a-9bc1-4cd7-b7cf-c6569ed9e4aa
9ae33a63-6b1a-43bf-9205-463c867bcbc4
9aecfc8f-62ea-4acf-aa00-d1f0fe6c6556

9b3e7a03-cf8c-4da8-bc36-20129d7783ba
9ba2c970-c622-11e3-bf01-24c6515278c0
9be1246b-3dc1-4928-8b89-2d5ed0c866e8
9c181313-5b4c-41c5-9ad5-a5a86fe609aa
9c681cd9-25fb-42ac-aa6b-bb962882fa22
9c70688d-6e43-4520-9262-eaae4e4d597d
9c730cfb-3fd6-428a-a0a4-c061ca5db06c
9c857452-c81d-4b9e-87a8-5b41f4394f7c
9cb80280-c622-11e3-bf01-24c6515278c0
9cfa4e8f-ea4d-433b-8c2c-54f66ecb7e43
9d29543e-8601-4fd0-8e76-3df3de465cab
9d3b0b87-5fc5-4d09-9c63-4d5fc4bc46d0
9d691fa3-5c32-4b98-85c4-f6e0fd4c37e0
9ddf2119-a222-4fa5-a9f3-0bec7eeea36b
9df57388-7de1-4c90-910e-ef63a246dff1
9e0009d1-c993-4247-9706-88ee84591dec
9e272bea-9193-4c10-a802-f7e18b2937a3
9e6806c4-d331-4025-abee-2f576e3bf182
9e7b51fb-2249-451a-bf23-bb2a3d935928
9ebac79d-8b38-4469-837e-b834725fe6d5
9ed8f0b9-d615-44c4-9ec9-e07ecd744922
9fba5db6-c622-11e3-bf01-24c6515278c0
9fc0e0e8-1b0f-400c-b4cf-126193283695
9fc5b5c7-3973-42b4-8710-454de0cb5b50
9ff21093-58d7-4b69-aade-c242a383ea56
9ffe694e-b488-489e-bdbe-0800e505eec4
a0583407-712d-46f3-8c4e-4eeaa0149087
a0bbb3b1-e774-4c75-9301-ba43fb803f20
a0e317b1-3f3b-4d25-b2d9-fcc2211dfda3
a0eb2611-1ca6-44b7-897e-ce18399eb7bc
a0f94565-9169-49ee-8e6a-21bdc43d9de6
a0fe80e0-e3ac-485b-8a1a-4c80c101b874
a1724132-f95c-4622-8987-fe3183b9dce3
a1fc7064-abf5-477d-983e-576a784e1d40
a2034620-e4b5-4d4f-ac05-7fc1c098eb40
a2238b5f-0513-487a-9b1b-7bef85ebf336
a29278af-7ecf-403e-b6a9-623ea7879d05
a2ee5dde-4342-4e78-88c5-3c9a8e10cbf5
a2f143d6-ba07-4ff0-960d-b29c3c716665
a3135834-3af0-4e98-bc6f-ad8ddf33db80
a315b798-f037-48d5-91ed-e733e3bbd6ee
a31b6123-c1b7-4cd0-86d8-b2e640cce9da
a330a96e-9897-4605-b5f1-5b5ef45cd365
a335b03d-41ac-4d41-a2a9-3134b5b0a0a7
a37a825c-d74e-46cc-92ef-b65016857b4a
a3deafcb-b54f-4b7e-89ac-c92c16fb919f
a3edc9cc-f54a-4459-a5d0-097879c811e5
a441697c-4ca0-4eab-9b01-b9cb50223ce2
a47c2012-c13d-48ac-88b6-e09bfd50122b
a492a32b-bf2f-4f8c-b06e-794a4baa2fe7

a4beedc3-0e96-4e1c-90b4-3674dfc01786
a4c838df-6ac4-455a-8096-8ec680064374
a4ca18dc-c622-11e3-bf01-24c6515278c0
a54aac0e-4108-49cf-bf50-687031c7cda6
a592e80a-3b1a-46aa-be30-bcd87d6bca6f
a5d5c238-0447-47fa-9a81-3b823cbdcea6
a64000df-16e5-4b88-865f-5c680adf60eb
a6c14ebc-81a2-44f7-af87-2b7ec85e5696
a6e24c2a-26b9-409c-bbcd-a8fcdd1e437c
a762dd44-a8ac-4269-8163-bbf6d0f8d4b3
a78544d7-65c6-4778-af62-ceec24c14056
a7fb0931-28df-46f4-bc0f-2011fc91f0e1
a8041e86-071e-46cd-8fb5-7ecdc1ab0553
a84915de-6562-4836-86f9-f2a05598296e
a876398c-5b1d-444f-a360-5fe2db697480
a8f86b64-914c-4d89-897b-33bcdd1759f7
a92023de-5c97-4bf2-aa3c-0e768d7c5ece
a9c29edd-2ef1-46ef-93c6-796cf0332a2b
a9d055c6-6c53-4e85-8270-34883a1342a9
a9dbd55c-5dcc-48db-8785-6baef3fdd7db
aa018d90-5250-4313-9f7c-a3f196765e07
aa06ae46-2336-4666-a3af-fc790daf8da4
aa20b9ab-dba9-4002-ad45-cafa2f85cef7
aa215f18-ae1f-4f0e-89dd-ac59ee8c8c44
aa4a868a-df23-4eef-a618-e945aa2ce98a
aa58c09a-7799-4675-8310-d270224b163e
aa847c3d-d3a7-46aa-a81b-db5099a458fb
aabddb20-c622-11e3-bf01-24c6515278c0
aacaba97-ecde-440b-aae0-0e9c608c4b24
ab923db7-54e8-4a50-b7fd-c2b4b300041e
abc94013-71f5-4ac6-88a4-01b4ef9f9d2f
abd2d959-d5ed-4eb3-9759-67eb1aa23325
abe290ed-67c8-42bb-84f7-0d32a5f8f3db
ac02c38e-5fca-4995-b0cc-39b6189976a3
ac1bd179-8285-468c-ab9f-7f91151ca0f2
aca4d810-9540-4ce3-8ceb-72d396c9b704
acb5e263-111b-43d8-9bd1-adb9a54e0fc0
acb9750e-7e17-4cef-bee1-5d710f9bf3a7
acc629cb-ad03-4cec-9b21-922e4932ef3e
accfc45b-eae0-4991-a488-e217cdb46655
acd510de-b732-4a1a-8b72-6d22e763540b
ad007b59-46f6-48b9-958a-2b9d28cca3b7
ad0700e2-048a-4856-a77a-bc0b6f25f0e2
ad41f1f3-e38e-4418-9c16-52bca5719f39
ad4aa4dc-bea4-4f4a-8b1b-8f361112f84f
ad874249-9964-4e48-aa3f-7e828ea28f3f
ad9455e9-7147-489e-9b1f-3540c457c260
ae82fead-2671-4335-a342-67bb69935ad9
aed4363b-19b8-4cb5-aa34-a442410d0796
af96db5a-684f-41d1-a910-5a5193393d9c

af9cfac8-7fbe-49cb-aeca-8d68aea57994
afee5b10-3dff-4e50-9575-bc9fe20c5dea
affa4ef4-02ec-40f1-b6a2-739f8bf2afa9
b02b4bba-6e66-44fb-a48f-38c309aaaac5
b07bad52-d44c-4b27-900a-960985bfadec
b0a83df8-dd2c-4c1b-b238-9081d2c22258
b0d1fde4-928f-4f4c-bff6-33b885582bb5
b19ec4cd-2876-4a2b-bcb9-b3c8db54fc15
b1e62d8e-24d2-4118-8cd0-3142acebdd5b
b24feafa-f2e9-4498-8471-a9e42c80cc9f
b27b569a-2c79-4453-8a2d-41fd0b4dd28d
b27d75ba-5989-4200-bfe9-f1b7d7cf8008
b2d17671-d2e1-4c97-8b01-a976d5abe1d6
b330c50b-c45f-413d-918f-33275d0d8f91
b35d9a68-29f4-49ab-b83e-b5151679e3af
b37d6283-6f95-4975-a794-f3d5c4bbc7b3
b38d0777-4901-48b8-9cdc-33b7f13a424f
b3b3a27c-ee9a-42af-a6d1-9af5970a98b9
b3f1d232-4392-4258-9256-d38ff4b59f94
b42d183c-bc9c-4652-9e56-10c54c5ee96e
b47aa163-eec9-4225-940b-4373e78152e2
b4860b5f-0a80-4fb2-81e1-4bafeb525b1c
b499032c-6cb8-4646-a95b-10af2a21a289
b4e5626b-44fe-4cdb-a7d5-42028e0dbc4d
b4f515ce-5bee-4e40-a66a-01f41e92181e
b51fa117-2c0a-4e1d-8ec4-9f039073a486
b537bc07-9998-49f4-bbbe-5b617152bfd9
b53dcb4d-f894-47b8-8a4e-17c7998d8120
b54b9433-ec10-4cb5-a860-4555da64917b
b5cabba2-30a4-458e-897c-00ec3fefa6d2
b5cc4a6d-93a9-497c-a7bb-a5db82edc176
b5dd0bc9-6c1c-4272-b920-744de37d0aee
b60392fb-43d9-4c9c-b91b-ded40492e61c
b6365bdb-e6d5-4615-95b3-db12a0c2f3d9
b64173fc-abac-4695-902b-f0acf9444e9f
b68f18e1-0117-420c-9405-e8d3a1e8caff
b6b6e1d3-c592-4836-bdad-d53af2dbcc9a
b7008a98-5aa0-4cc9-9bee-af2a422266db
b710b214-5666-4054-97ef-3b9ac2bd90ac
b75b2663-dcc6-411c-bfcc-574aa33cf388
b77084ab-8148-49e0-b3f9-90f7d9ea1862
b7a7d93b-38a7-4fc3-a433-3bb0a8cb7c42
b8068cbb-4523-4ac9-ab17-edd4eb0e2975
b80ca528-2c5f-400c-8df3-9c47869a64c2
b8151614-b08f-49a3-ab6f-2e780f765a17
b88f014e-bc0f-4775-8228-39e0b471c3b0
b8cd6882-be27-4742-bc63-3227d31bf704
b8f3137e-5e92-4a56-90d4-884a4ed2ef9c
b9098b7c-eab0-4ef5-b968-8d3024bf32f7
b97941dd-9844-4db2-9e25-42c725f47d70

b9d1a64e-d445-4174-a5b4-76dd6ea69419
b9d2e29e-8546-4310-aeea-e029f17c0516
b9dbc78e-44ce-427c-bbeb-de8d097bde2c
b9f03936-1de5-4330-8b0f-6d0ab533876e
ba5667da-7688-4931-bdb7-d65f391caf60
ba686386-8113-4885-b20f-6ad09a295604
ba94c29b-b76e-4d67-bf5a-ce6bc45d85f8
bb02202f-d140-4bec-95ce-5c2b117a2167
bb45c6f5-49cb-418d-85bb-3ded165e2d07
bb65d670-0411-4fc5-a6cb-019caec1ab36
bb8176a7-ea7c-42d7-a85a-cec1ca386f2e
bbb2cf2f-8f32-43d4-846c-d1020e6329ec
bbbc531a-dd41-40b7-ae59-e35508aea0c7
bbdd7393-024b-4073-b5bc-ef7a68c15b8a
bbe59385-5f83-43f6-a485-517c860bef6f
bc9b66f5-fcb8-4545-ab2d-438bb810edc0
bcd52bcb-6e35-4220-9775-96dabff926b4
bcefbf6a-80ff-4825-9d3a-492e2dceb35d
bcf76f1a-7109-422d-94c9-5e8364895fa0
bcf858fd-cc3b-4fde-ab10-eb96216f4366
bd2cc59d-f5ce-46ed-beb0-4c80b24cbe71
bd2ec47b-a120-4b3b-b10d-aee09805ab33
bd403458-5154-488b-931a-a7e737a6bf8c
bef21282-c622-11e3-bf01-24c6515278c0
bef23192-57cd-4301-b7a7-5e82ca62482d
bf24366f-fd91-4d84-8b3d-eed80064250d
bf91afc4-aa2b-4365-80c5-b98c9d118e10
bff518fb-6da7-4dfc-ae4c-bd3f641028e2
c00d925a-ca4d-4561-a8f2-d4542f218086
c00de7a0-0b09-4e07-988c-ef2a7f8e932a
c05adc19-2e01-4712-a35a-69eee4d40998
c0812962-a345-48b7-aec0-01336c2d1eed
c082dc34-457e-40ec-8258-e11e8ed362c2
c13d7ee5-2bf0-43e4-9cd3-9326ed9355cf
c14407ad-670e-4d1e-9417-2b76f4810fff
c19a1388-95e6-4708-a24c-3738f1908071
c1b44966-0f72-4c4f-8783-ab3ffe7f17b2
c1d9ed06-7498-4c6c-a0de-dbf28e868109
c20cb5c8-a6d0-4150-b72b-3f7e8909d72b
c2124bb2-30e1-4093-9a8a-3a188a6dc0a9
c21a5d55-9790-429f-a4e6-420293c07b53
c27290e4-6835-448a-abdc-df8ddd5f4630
c285c2fa-24b4-47a1-874d-86e74b002b05
c298ec82-8305-4c89-898f-268308f782b2
c2aafb9c-230a-43df-9a43-b34aa145a7bd
c2ec7f57-8510-4bbf-a2e9-dbd9ce8dcad1
c307688c-b1fa-47f6-a9e2-1ea41f7645b6
c36213e4-c057-46f5-ada5-f1f921855bb4
c3679262-63d7-463e-9501-6ab3f177aee8
c437535b-2acc-4236-beae-94e2c1336830

c488a170-c622-11e3-bf01-24c6515278c0
c48d7f4a-e98a-4077-a749-eb9f56f05b84
c504d5a9-29b0-4b7e-ac7b-5e543449a0f4
c556f81b-8a6c-4bbb-876f-2e2ce570c185
c56505cb-e150-408d-9df6-3af4e608c376
c59188a8-c6bb-433c-97fe-69f79a126d03
c61ded54-f80e-4da8-b1d8-e22551c96118
c6224ab0-74f5-4f0a-9fe0-c083f806c853
c66926a8-474d-482c-bfb7-043029c8b737
c67409b2-ac25-42a0-8543-4636ef132fe4
c6888be2-4517-41e4-a801-9c915f24e737
c691d6b3-8e70-4951-8551-2ee4e4d0d10b
c6a28615-1b92-4085-956e-eeedb2573c03
c6bb32f0-c622-11e3-bf01-24c6515278c0
c6d6f49e-6e95-4e41-8388-71f6fe017d80
c741fb12-8160-43a7-bc70-2c545ea0031d
c767254e-b289-4904-a80f-050cf01ff8ba
c76f7fcc-2748-4b6d-805e-d21652dd5e3a
c79ce3d3-4758-4f52-9da1-38f994c99f4e
c7d798b8-c622-11e3-bf01-24c6515278c0
c8f64a71-e545-4eb6-9da3-2d43ae0c2627
c955387a-16e5-447c-8e69-e4c04a9861d5
c95a2b1b-726c-4608-9fff-d57b6f1aa75a
c9959f68-c385-4c1f-9188-8203844d288e
c9ad6b1c-baa0-45a7-b7c4-733728505b8a
c9e7c629-7b57-4ede-b315-0cea8c97c48e
c9e7cff0-aec2-4ff2-8091-7cbea2bf5991
c9f91ded-3b04-4cd1-8ea6-bbc635a8a4f0
ca284012-bd06-47a3-8a9d-c6b185d87d85
ca2b9fe2-97e0-4d4f-afd7-a5acf638800f
ca5ded1c-c622-11e3-bf01-24c6515278c0
ca8fa9f5-3190-440d-9879-22e33d05ca6c
cafe9603-a804-48fb-9217-e2d1c3c346c5
cb381d49-4546-400c-af02-f877083caa0a
cb56e4c2-2fff-4f09-be34-fbdaa3794b35
cb5e1546-cda6-4991-911c-f3dd9f1a475a
cb783f8b-d092-4104-b809-3f90b19df7df
cb89cea8-f78e-4024-97c2-8f24debee1bc
cba4de91-cce7-4edc-8450-d0a52c959586
cbb788dd-964b-4cfd-80e1-979c7b5b3c52
cc39b534-5ba1-4ea0-956b-676f6732beec
cc4f9175-a8c8-424a-99d7-8bd11cae0e52
cc69fe94-0ecf-45ad-a03f-8fb7198e8657
cd0aeed5-93a1-4287-8a88-fe6b7b5e3983
cd2a045b-fbde-49c6-8be6-c0e3ecee246f
cd9efdef-a7fb-49e5-9515-63606ae8bbfc
cdbbd701-9c05-4f9e-923d-06039dd8a04d
cdc672cc-35df-4f10-88f1-d3e40dabf601
ce6154e9-3f3a-442f-a608-35e1bab66927
cef1fb1c-18fa-453a-b87f-295d3b0bc12b

cf5deb22-f7eb-409d-a0e4-882716199c39
cf6c7066-6611-43ec-aef1-295a976cce5b
cf777ef1-765f-4828-b789-f5aa6722eb35
cfe0bf13-2ff4-4164-9b6a-cd3615bf3ddc
d00625b4-6e0e-4d96-8e4c-8f1ddcc7aded
d0161ca7-3c33-4fe5-80df-0cc4b3c4e8ee
d01710f9-efc8-4b5f-9e53-300b30f9219f
d02597f8-3ac7-4165-a65f-0e134e5d215b
d0469256-c622-11e3-bf01-24c6515278c0
d053f88f-09e6-472e-a691-bb293c461399
d056645f-4dd3-49f3-b0a4-2293e2b4d82c
d05ea63c-86a3-463a-a790-2edaa74b4da7
d0a0d845-32e2-4590-b909-29bd27b92900
d0a33fd2-085c-488b-b843-2ca97c85a5cb
d0e033bc-8e97-4322-b3b1-8626f1e2449b
d0f4dd92-a63b-4766-8889-5327fca1bd91
d1132127-1250-43af-9c16-425798a3d1a7
d113ce88-04ab-4675-b363-92f80c28de34
d12cfd8b-682d-41df-acf8-ee7f68a6241c
d17c2123-d262-43f4-9d06-3547f991cde7
d182b67c-c622-11e3-bf01-24c6515278c0
d18c6e64-9598-488a-8ab3-3652072f9277
d252a54e-0377-4b3e-8228-26108ea704fa
d252f328-4583-4e97-9a71-bb2885f06f73
d25a4c65-9cb4-4611-909e-e68f93408d84
d277e679-9fdd-44cd-9f2c-f9927e44d721
d2ab2555-7288-47a4-a80c-bf62d65b67b8
d2ab7fd4-d1c7-48ae-ba9a-78c5d811465e
d2b7bb0a-c622-11e3-bf01-24c6515278c0
d2e4dc23-992d-4e44-add4-2972718083cb
d31cce9d-1b38-44fd-9378-0d128e12a011
d333b55b-8bac-4a99-9d23-3cc0c25057bf
d3709eea-ca2a-49ac-b79c-871a5473d450
d3aff5d3-23c0-43ae-9c01-8ddd776b530b
d3d65db3-36f9-41c7-8e5e-1683ce94dfcb
d3daf373-1485-455e-a0a1-8e33d215d623
d414e076-dcb6-4edf-adef-0abdc57354ea
d432e99a-67fb-4609-b90f-99438eee7cae
d4615ca0-b5c7-4a5c-8593-bd50034a78ae
d4907a1b-8b06-47c5-8bca-c781d9cddaf8
d4c9e5c6-c622-11e3-bf01-24c6515278c0
d4cf11be-3bd7-403b-9722-91d8e8761e4d
d4ea36e2-1323-4441-8c63-40ce269608f4
d515c7d2-2314-46bf-9736-ff3f3d2ab599
d5372745-95cf-4572-a6e7-e7f5b796911a
d53c2680-89ac-4f87-a7ba-f4c669546b76
d5796835-d6cd-427e-a398-5fbef88b0648
d5c87d16-172f-4790-8867-949dcaa534d4
d5f92ce6-796f-480d-bf98-e1080a279207
d60f54f5-b154-42c4-99fb-cea4e7a33dc7

d60f880a-c622-11e3-bf01-24c6515278c0
d648c804-4eaf-4252-8b6f-13378158a07d
d6738c83-7b52-4224-846a-67bd085aaab8
d67cad13-e849-48b0-926c-10b6046ba0b9
d6ccfd6f-2ea9-499d-a25d-4ba9d4a9258e
d6ee68e5-ceb3-4a63-b3bd-e64fe5398102
d709d214-02ee-46c1-a73a-e7074cb2623e
d72a7338-c144-4c62-a70e-2177a3b4b77e
d7f8e1f3-a9a7-4929-92df-1283bac815d2
d8415cf6-8e4d-4c54-811e-199da14237f1
d88296ac-bb0d-47ab-9051-6c358f0af780
d8995dfa-a780-4fe6-b3f8-fb464f51a1cf
d8c2b4b2-e12b-43d2-bafc-87b29f027797
d8c6d4b8-f279-4edc-aaa3-a1cc266aec4d
d8d5585d-32cd-4ac4-b410-a4122a17a558
d8e2af63-afe0-4f15-88b4-78118cf68160
d8f0becd-fda8-41f4-a424-e082f9eae22c
d8fbb398-d1da-4444-984a-22c8523625da
d91f487e-0895-44ef-aeb1-a62bd576f8b0
d9338d73-1d5e-4808-91f2-b5a09f7342de
d9690a27-bb02-498e-b15c-ee76e31d16ee
d991f23a-dc1b-427a-b798-bd4187d3aa02
d9c5493e-f969-4c04-a646-9a3134011021
da1ed4b9-f428-48aa-834d-1d9673999044
da43386c-47f8-4e03-b6ca-8b94e13792e7
da910f22-126e-41dc-be6b-47c8f69ad106
dabb5a94-9706-4429-8db0-c328e2004340
db198301-6c69-4d56-88d1-c650406423dd
db2a1df8-487d-4dad-a347-5c5ed539d5ad
db4592fb-13c2-4d61-9dc3-a5d43def8d4b
db98d6d5-94a5-4559-8f29-49eb74f83ce3
dbacc1ad-c88a-4bcf-a45f-e9c83c762164
dbbd54db-4470-4df6-b5be-3e175c7133b0
dbfdb5c6-f52e-48f3-87e7-fb922df9e7a9
dc4ba4bc-6333-4fe9-8805-e058cc9e6e18
dc505248-ed04-4f77-a7c6-3fefbc5df27b
dc57c2e7-4c5b-4e2b-93c6-f032fcebc2fa
dc7faf84-4438-447b-abcf-a3af87043654
dc85552c-2488-48d9-9da1-67a380945b85
dce54d09-9827-4fe2-abe1-c5b7d528ba7f
dcf45005-7e6d-4c26-9994-f973da7e814f
dd4fdb6a-8067-4b64-ab74-bbb0fec34ca9
dd5ed858-0623-4387-b1bf-109175c32ef4
dd67dec6-35dd-4efe-b913-ed4884855365
dd7d623b-b9af-4147-9aa6-e09793691f10
ddc422e4-87e2-4fc0-b56b-ddc2d6a92f58
ddc7377d-82c3-480a-be3c-3d1da52c77d4
dddea2e4-b8c3-4157-9d92-6de472e8375a
de4c3f91-d164-47df-9971-feba840d247c
de581588-be15-4bf4-95d1-aa5478b53ab6

de99a4de-e916-4572-ac9e-73e341827229
ded2689b-8e37-480c-b37c-acc538e54ddf
ded8b673-53df-4038-a375-7cd0fd2a1b9f
df291849-4c35-44e1-b013-8f6b7ee36113
df2d41c4-7e79-4a46-aa4a-2a11c051ac40
df4366c4-170f-4233-b577-a8ea277b069c
e053d377-e4f2-4aee-af7d-e61605e0f4f5
e07991a4-0901-4ed1-8704-aa376565d862
e07affb1-b6b2-40a4-9c54-0e72f5ea5045
e0aa815f-668a-4de9-bb32-3f215bb7f843
e1217ebe-1826-41a9-b6c4-702100a66f5e
e127e9e4-c622-11e3-bf01-24c6515278c0
e1b169c3-44e0-43e5-9ee3-2df7a678a5ea
e1f0c565-6ed3-4e42-bc3e-c7b0003e837a
e1fc4226-40ce-43b7-b92e-aa7cecd0cf4d
e23462f2-cb86-4a3e-8521-221f4bec247c
e2774050-1257-4c29-b201-d79ce70fe17e
e2b2357c-da6f-4ca3-9c07-a22f0423e8fe
e2b5c926-491d-430d-8d9c-5c73d7752d1b
e2e6a409-b64d-463c-ac3b-e7987a3124f0
e30c64d5-e796-4cfc-8dc8-6e62bf97822f
e34bcc5e-c622-11e3-bf01-24c6515278c0
e3eb5217-50e4-49e2-a6fa-f847d091f3c9
e3ef5cad-4d6e-4f04-babb-a908ee02644b
e45f3391-2e74-4767-817a-280cebac7c57
e5193d7e-e8b7-4098-bf98-8b3a62781d13
e52ffa79-557a-4024-81f3-f3826c227ec5
e541b5b9-546d-47bc-acd6-4006ce0eec1d
e5593865-5f8e-4a4c-b36f-73fbe64d66da
e55f338f-97e2-4394-ae23-c92606069485
e64e9f76-c622-11e3-bf01-24c6515278c0
e6772001-dffc-4fb4-86e9-6c395edd176d
e6801359-d1d7-4871-b2fb-180674a2e469
e6c78a98-f45b-482b-a551-4f11b8c1ff8b
e6f7344b-951f-4f3f-b140-7bf53164c462
e7048a93-a1ed-4cdc-a1ab-fa507408ca77
e7335a24-e5fc-4558-9eba-511c5a802320
e75fbf08-c622-11e3-bf01-24c6515278c0
e7603fc8-9b73-4c17-86f9-46ca1bceeb1d
e76128b3-8629-4ffd-b02a-e3f0aae8b1a9
e7c90af0-4b03-453e-b44f-e07248415088
e7d74d34-3255-4c20-90fd-b105e6e229c8
e7dc7492-3a84-49c7-8dea-8f508b53dc40
e7ebc6fb-0926-4c8a-a67b-0c6b9c1ffaba
e84debc4-b47d-48ed-a0d0-2859f0ebf987
e9032df3-8692-4146-a867-b9b64c9b310a
e9445993-854d-44fc-a063-eacf70222fdf
e97ea3d7-9fcd-4df1-9ea8-587cb3be75ec
e99ccc0c-c622-11e3-bf01-24c6515278c0
e9cb8877-8e83-46c0-93f0-ea20530c9400

e9d607e8-41c3-4210-870b-a9ab9b1d1c8c
e9d98643-01ee-40c3-a617-e004559625cd
ea121956-77f5-4da3-ab15-0b825469eafe
ea1cac20-88c1-4257-9cdb-d2890eb2e123
ea2055d1-7b8b-4aaf-9a70-8802f4deef9b
ea43434b-197e-48ac-ae2e-46bc7f3776de
ea4a5ca7-86f9-47c7-81be-0c2c669a999f
ea6efcd1-11de-45f0-8bde-f1c06ad27e79
ea7d37ca-0dac-4ae6-ad03-97c6df3d116d
eab71331-53d3-4cf5-96c9-5121a3962c27
eae45fbe-9f7d-4f59-a0a9-1f0f0d69afc4
eb1531b0-8af1-4b2b-9192-644cb47101fc
eb588266-c622-11e3-bf01-24c6515278c0
eb59b79f-bd36-4bf4-83e3-2af73bd1d30a
eb9a8f12-6451-43cc-95b3-2e86cf704c96
ebe0ed67-2d3f-45cd-8f9b-4912595b16a0
ebe80d6c-7513-4f5e-9919-669b6d1fa68c
ec23a354-e339-4466-9a1b-8e886ef627e6
ec31e921-0782-4f77-ac23-4190af1e61ee
ec43c4b5-fb72-4a4a-af03-10c2d05ff159
ec474dfa-527c-44f3-9224-bf1c858cabfc
ec646623-6d33-4fbd-888e-89f55d82eb11
ec77847e-48fd-4ba5-bc3e-3cd1b149b552
ece0f3a4-a204-4c52-bb1f-88d44a875b2e
ecf4e05a-0912-4b93-ad66-323002f0c845
ecfe388a-99f5-44b9-9226-a6ae6d892538
ed1f5a41-921f-469a-bc04-306a5435e067
ed46bb2e-97e3-4914-a086-de80e00d6ee8
ed4b53cb-5473-4bc1-9d84-0b0ad93cbd57
edac1323-2497-45e6-9148-e9c955292ba2
edc070db-b768-478f-ae75-4c4012bdc3fe
ee368301-b2f7-428f-9330-67c054c1a09d
ee5d5e7d-78cf-4a29-a9ee-56aa3da877dd
ee6cc68e-8d2a-41ee-82c6-0fecdf7e6259
ee963667-56b1-49bd-8896-e07b536d3014
ef002c3d-358f-4fc0-98f3-2f8b89de4374
ef3a0ccd-85bc-4e5f-a8c6-5a2e2d9e6717
ef673d3d-2031-4036-ba25-4bc7ef04075b
efacd7d6-56cf-4f6a-87f0-1a7ab6b1a643
efbec43c-0c16-4006-abe8-c3ec2ec42c05
efc39172-083c-4297-b922-3f58df781332
efec3225-de07-4559-9a90-95223495cc61
effb868c-f905-41a0-ace1-b5e508732534
f03330dd-c616-4ad5-abc3-6d6b0445e9e9
f047cb85-8f88-45d5-83c3-0416c9f3fff7
f04aecf0-eb12-4ab9-928e-7bcf201b0b40
f0768c67-f38c-40a8-8f27-81a927fb5dca
f1504811-8363-41e6-b43c-62452b1262d3
f1a4f60a-5228-47cd-883f-ff8d2dfe1633
f1d9124e-dfa2-415e-b8b8-dd872fd3e2cb

f221c897-6ad0-0df9-e040-11ac0c4813ef
f221cbb5-eefa-187f-e040-11ac0c481708
f24688a9-7952-49d8-ba80-2bbad472379c
f266899a-1dab-4226-a965-bc1327c64080
f2b2111c-c622-11e3-bf01-24c6515278c0
f2c08ac1-4755-4c36-a11d-338d8df9fa0d
f38fc6ac-2af8-4d89-a514-4e2c5fdf08de
f393bafd-1baa-e5f4-e040-11ac0d48450b
f393bb01-6ed7-9533-e040-11ac0d484521
f39cc911-36a7-4c06-9e91-4ad86281db3d
f3c1a125-06e9-41c4-9558-275a3e346cc5
f4798483-9e32-4e6a-8bb5-6f45af2a8ac5
f48c3c82-bebe-4b8e-909e-e1a51a7142ec
f4ada7a2-c4ac-4f89-ada7-4645861002cb
f4b9d98f-7b76-4eaa-9595-10b0973d5ff7
f4e673a9-5b85-46e7-bcac-2ce6e1026896
f4e926fd-006e-4c85-b71c-1433c325acb0
f601cf2f-081f-484d-ab0e-21a8ec8d3770
f6114c69-71a1-47d5-9b28-b0227b1872f7
f6189828-eeaa-4d21-b163-53bf3d47a640
f61e794e-c622-11e3-bf01-24c6515278c0
f64e9609-d75d-400c-a92d-d77fd54d6c29
f658c350-fb89-4268-8a59-a07e365f4221
f663ff15-c8d3-4bff-8bf9-bf8e983579e0
f6c811ff-f22e-490b-9b23-b527d20e6e6d
f7187888-f261-4a39-99b4-966fd7207117
f7702c0c-9636-4700-a8ae-7a58afd9fa71
f7b847e5-0f5d-69b4-e040-11ac0c4845fe
f7b84b5d-8f28-e6b7-e040-11ac0c484704
f7b84c09-15d4-3046-e040-11ac0c4847ff
f7c6486a-bb08-f77a-e040-11ac0c482953
f7d128d2-3964-e3bc-e040-11ac0d481667
f7d77a03-28a0-f446-e040-11ac0c4866fb
f7e4a2a8-093d-9e36-e040-11ac0d480c7d
f7e7d61f-e2dc-b523-e040-11ac0c482000
f7f66114-e321-1d4c-e040-11ac0d48541a
f7fdda4f-7bf7-ede7-e040-11ac0c486e57
f80293b8-ddb7-f575-e040-11ac0d48309d
f81693ba-09ee-4201-a389-0ceeda8a4636
f82d213f-9ba5-7b6b-e040-11ac0c486882
f82d213f-bc06-5b51-e040-11ac0c48687e
f82d213f-bc99-5b1d-e040-11ac0c486880
f83f1eb8-b2c8-c3d6-e040-11ac0c48261e
f83fc777-5416-c3e9-e040-11ac0d482c8e
f8407106-8a96-8f85-e040-11ac0d481656
f845ebba-8c56-b559-e040-11ac0c4863d5
f8467ec8-2d61-ba21-e040-11ac0c483584
f848b66f-bd9e-4fba-afd4-eb58848d1ef4
f8515e5a-7de3-6be3-e040-11ac0c480d6d
f856fa85-fdb8-c0b0-e040-11ac0d480b4e

f85add3d-e2e6-31f8-e040-11ac0d48479c
f85ae2b7-cebf-17a2-e040-11ac0c48033a
f860bc4c-f31b-6538-e040-11ac0c48268f
f866123a-9eb0-e856-e040-11ac0c48717e
f86ae246-2492-9785-e040-11ac0d4813b0
f86b7e84-7040-c751-e040-11ac0c485675
f86e2d80-911b-7a19-e040-11ac0d486900
f87348df-7186-4c6b-e040-11ac0c482ba9
f87cdca1-2c85-49d2-e040-11ac0d485c9d
f87d7c27-eeef-920e-e040-11ac0d48388d
f87e94a9-ef37-fcba-e040-11ac0c482332
f8809cee-8f81-28f8-e040-11ac0d4873bf
f89ef95e-6e39-420b-a3c2-ccebcc69ce3b
f8d89ae2-2c7d-40ae-8d40-34b93b16efc9
f8f749b7-547d-49fa-9da2-44eed962b6fd
f90cab09-6797-eccb-e040-11ac0d486aa3
f90d51f8-3bc4-2e66-e040-11ac0d484d19
f91bde65-233d-2218-e040-11ac0c480a33
f920200e-f349-3730-e040-11ac0d4825cc
f92a34fa-014e-4b41-a6d0-3b46b8c8a3ee
f988e698-9b34-45ce-ba4c-74e06e9cae4a
f98de26b-c7d6-435d-81fa-1f1869da9087
f9a81200-5381-496a-8062-099f9e793618
f9c0a08b-d1e5-4c18-e040-11ac0c4864df
f9c0a12c-2651-d9ea-e040-11ac0d483201
f9c1df10-25b8-c8ae-e040-11ac0d486375
f9c23ce8-7f1c-9417-e040-11ac0d482562
f9c39eb7-39a9-6626-e040-11ac0d4870c2
f9c3bc8e-dbc4-1ed0-e040-11ac0d4803a9
f9c3e4e3-1b95-b71a-e040-11ac0d4819e7
f9c3eaad-a0d9-8bf8-e040-11ac0d481d8e
f9c4e06c-e8a6-613b-e040-11ac0d4828ba
f9c51617-3fcb-91c5-e040-11ac0d484abf
f9c52414-385d-8cc7-e040-11ac0d485037
f9c650e7-9053-78eb-e040-11ac0d4874bb
f9c65e3d-f3f7-dd5f-e040-11ac0d487b1f
f9f04e42-63a2-4ee1-ade4-9e5bb5c20cdd
fa2c3620-7e82-4b12-b135-87986f0f2890
fa4d35e3-fc38-4e26-a9e0-521a648acec1
fa54205f-7e51-4e5a-9f0a-5e8a2e40235f
fa5e2900-95e7-4700-9707-a329cd0c77fd
fa676301-902f-473f-8313-5bff34ae549a
fa9996c3-b874-4424-a5a6-f1c7c0f42b9b
fab3d6f4-de1c-4d6c-bdea-ac39a7884657
fae88d21-171c-4df4-b258-3c5948a1034b
fb0c6353-a90c-45e2-9355-7cd16cf756ff
fbd899a0-c622-11e3-bf01-24c6515278c0
fbea74b5-b1a8-4767-af7c-e179ce8da071
fbf937f1-5f30-470c-8a77-46cd1c41e073
fc249113-83d4-4abe-8c80-a4f7305dcd91

fc447d53-24d1-c83d-e040-11ac0c4846a6
fc447d56-0d53-e0c3-e040-11ac0c4846a8
fc639186-8302-798e-e040-11ac0c484cec
fc63cbab-d27a-5ebb-e040-11ac0c48724f
fc68e599-6a40-61dc-e040-11ac0d48700d
fc6d77a9-121b-48ab-a899-713c3d1319a2
fc7eb420-5c13-20bd-e040-11ac0d484574
fc812906-d3b6-4210-a842-96f095280cbf
fc8130df-18fe-c74d-e040-11ac0d485df2
fc8130df-1bc2-c537-e040-11ac0d485de6
fc8130df-1cdd-cade-e040-11ac0d485dec
fc8130df-1e8f-c879-e040-11ac0d485df4
fc8130df-2211-cfcc-e040-11ac0d485dde
fc8130df-24d9-32f1-e040-11ac0d485df0
fc8130df-2628-304a-e040-11ac0d485dfa
fc8130df-297f-34a3-e040-11ac0d485e02
fc8130df-2ca4-39d2-e040-11ac0d485e00
fc8130df-2e39-3814-e040-11ac0d485de0
fc8130df-3147-3e94-e040-11ac0d485df8
fc8130df-3361-3c8a-e040-11ac0d485dea
fc8130df-34f8-22c6-e040-11ac0d485de8
fc8130df-35ac-2304-e040-11ac0d485dda
fc8130df-6977-7704-e040-11ac0d485de4
fc8130df-6bec-7627-e040-11ac0d485e04
fc8130df-897d-5404-e040-11ac0d485e0a
fc8130df-8ec8-5b1e-e040-11ac0d485e06
fc8130df-90ba-5d94-e040-11ac0d485e0c
fc8130e0-095f-b953-e040-11ac0c483287
fc8130e0-0b9c-bbc9-e040-11ac0c483266
fc8130e0-0bfa-bba4-e040-11ac0c48328d
fc8130e0-0c53-b432-e040-11ac0c483283
fc8130e0-0d25-b475-e040-11ac0c483277
fc8130e0-0db6-b568-e040-11ac0c483281
fc8130e0-a774-d834-e040-11ac0c483268
fc8130e0-a94d-d9d0-e040-11ac0c483262
fc8130e0-ad63-b804-e040-11ac0d485e10
fc8130e0-ad8a-b832-e040-11ac0d485e14
fc8130e0-aed2-bb2f-e040-11ac0d485e12
fc8130e3-023d-c7d4-e040-11ac0c483295
fc876f51-0577-3b53-e040-11ac0d48515e
fc876f5c-8339-bc9c-e040-11ac0d485160
fc95d5ce-6899-62f1-e040-11ac0c486011
fc962061-8ab0-42b3-e040-11ac0c4810cc
fc9650a1-9aef-ab3b-e040-11ac0c4829a7
fc968a86-32e3-ee88-e040-11ac0d487adb
fc96e36c-67f1-4c02-e040-11ac0d48393d
fc9d5fc6-7f7d-1313-e040-11ac0d485f85
fc9dbf68-4599-3c64-e040-11ac0d481623
fc9f6cb0-ceb9-790d-e040-11ac0c480ad1
fca07e09-1fd2-9ef5-e040-11ac0d485bbd

fca08ee9-b480-4dc7-be56-f1eb03b56f7c
fccb865d-83ed-4bfe-ad84-0689813ae977
fce8d8c6-f2a0-43a8-9a7a-b9c519a6686c
fd504153-3cf6-44b9-99d5-21961ebac188
fe04d042-a4cc-4a14-8197-415ea40951aa
fe63d42b-d471-45b6-9bdf-1a3b55465d37
fe96d91c-3686-4125-af71-b8703a011ad4
fea5827b-251d-474b-8713-fc76db995fe7
feccc27e-5c2e-4339-8746-0945fee93c1e
feccee20-a62d-4152-b832-b9fdaca87a61
ff3fe4a3-7d19-4d4d-81b6-aaa41ba5bf39
ffb4f42b-58e9-40c3-8963-11804f041375
ffe4bb51-e98a-41a7-a4e1-c3970386889c

Supplementary Note 2

PCAWG Consortium full author list (excluding paper authors).

Steering committee

Peter J Campbell#[1,2], **Gad Getz#**[3,4,5,6], **Jan O Korbel#**[7,8], **Lincoln D Stein#**[9,10] and **Joshua M Stuart#**[11]

Executive committee

Sultan T Al-Sedairy[12], Axel Aretz[13], Cindy Bell[14], Miguel Betancourt[15], Christiane Buchholz[16], Fabien Calvo[17], Christine Chomienne[18], Michael Dunn[19], Stuart Edmonds[20], Eric Green[21], Shailja Gupta[22], Carolyn M Hutter[21], Karine Jegalian[23], Jennifer L Jennings[24,25], Nic Jones[26], Hyung-Lae Kim[27], Youyong Lu[28,29,30], Hitoshi Nakagama[31], Gerd Nettekoven[32], Laura Planko[32], David Scott[26], Tatsuhiro Shibata[33,34], Kiyo Shimizu[35], Lincoln D Stein[9,10], Michael Rudolf Stratton[1], Takashi Yugawa[36], Giampaolo Tortora[37,38], K VijayRaghavan[22], Huanming Yang[39] and Jean C Zenklusen[40]

Ethics and legal working group

Don Chalmers#[41], Yann Joly[42], **Bartha M Knoppers#**[42], Fruzsina Molnár-Gábor[43], Mark Phillips[42], Adrian Thorogood[42] and David Townend[44]

Technical working group

Brice Aminou[45], Javier Bartolome[46], Keith A Boroevich[47,48], Rich Boyce[7], Alvis Brazma[7], Angela N Brooks[3,49,50], Alex Buchanan[51], Ivo Buchhalter[52,53,54], Adam P Butler[1], Niall J Byrne[45], Andy Cafferkey[7], Peter J Campbell[1,2], Zhaohong Chen[55], Sunghoon Cho[56], Wan Choi[57], Peter Clapham[1], Brandi N Davis-Dusenbery[58], Francisco M De La Vega[59,59,60,61,62], Jonas Demeulemeester[63,64], Michelle T Dow[55], Lewis Jonathan Dursi[9,65], Juergen Eils[66,67], Roland Eils[52,54,66,67], Kyle Ellrott[51], Claudiu Farcas[55], Nodirjon Fayzullaev[45], Vincent Ferretti[45,68], Paul Flicek[7], Nuno A Fonseca[7,69], Josep Ll Gelpi[46,70], Gad Getz[3,4,5,6], Bob Gibson[45], Robert L Grossman[71], Olivier Harismendy[72], Allison P Heath[73], Michael C Heinold[52,54], Julian M Hess[3,74], Oliver Hofmann[75], Jongwhi H Hong[76], Thomas J Hudson[77,78], Barbara Hutter[79,80,81], Carolyn M Hutter[21], Daniel Hübschmann[54,66,82,83,84], Seiya Imoto[85,86], Sinisa Ivkovic[87], Seung-Hyup Jeon[57], Wei Jiao[9], Jongsun Jung[88], Rolf Kabbe[52], Andre

Kahles[89,90,91,92,93], Jules NA Kerssemakers[52], Hyung-Lae Kim[27], Hyunghwan Kim[57], Jihoon Kim[94], Youngwook Kim[95,96], Kortine Kleinheinz[52,54], Jan O Korbel[7,8], Michael Koscher[97], Antonios Koures[55], Milena Kovacevic[87], Chris Lawerenz[67], Ignaty Leshchiner[3], Jia Liu[98], Dimitri Livitz[3], George L Mihaiescu[45], Sanja Mijalkovic[87], Ana Mijalkovic Mijalkovic-Lazic[87], Satoru Miyano[86], Naoki Miyoshi[86], Hardeep K Nahal-Bose[45], Hidewaki Nakagawa[48], Mia Nastic[87], Steven J Newhouse[7], Jonathan Nicholson[1], **Brian D O'Connor#**[45,50], David Ocana[7], Kazuhiro Ohi[85], Lucila Ohno-Machado[55], Larsson Omberg[99], BF Francis Ouellette[100,101], Nagarajan Paramasivam[52,80], Marc D Perry[45,102], Todd D Pihl[103], Manuel Prinz[52], Montserrat Puiggròs[46], Petar Radovic[87], Keiran M Raine[1], Esther Rheinbay[3,6,104], Mara Rosenberg[3,104], Romina Royo[46], Gunnar Rätsch[89,92,93,105,106,107], Gordon Saksena[3], Matthias Schlesner[52,108], Solomon I Shorser[9], Charles Short[7], Heidi J Sofia[21], Jonathan Spring[71], **Lincoln D Stein#**[9,10], Adam J Struck[51], Grace Tiao[3], Nebojsa Tijanic[87], David Torrents[46,109], Peter Van Loo[63,64], Miguel Vazquez[46,110], David Vicente[46], Jeremiah A Wala[3,6,49], Zhining Wang[40], Sebastian M Waszak[8], Joachim Weischenfeldt[8,111,112], Johannes Werner[52,113], Ashley Williams[55], Youngchoon Woo[57], Adam J Wright[9], Qian Xiang[114], **Sergei Yakneen#**[8], Liming Yang[40], Denis Yuen[9], **Christina K Yung#**[45] and **Junjun Zhang#**[45]

Annotations working group

Angela N Brooks[3,49,50], Ivo Buchhalter[52,53,54], Peter J Campbell[1,2], Priyanka Dhingra[115,116], Lars Feuerbach[117], Mark Gerstein[118,119,120], Gad Getz[3,4,5,6], Mark P Hamilton[121], Henrik Hornshøj[122], Todd A Johnson[47], Andre Kahles[89,90,91,92,93], Abdullah Kahraman[123,124,125], Manolis Kellis[3,126], **Ekta Khurana#**[115,116,127,128], Jan O Korbel[7,8], Morten Muhlig Nielsen[122], Jakob Skou Pedersen[122,129], Paz Polak[3,4,6], Jüri Reimand[9,130], Esther Rheinbay[3,6,104], Nicola D Roberts[1], Gunnar Rätsch[89,92,93,105,106,107], Richard Sallari[3], Nasa Sinnott-Armstrong[3,61], Alfonso Valencia[46,109], Miguel Vazquez[46,110], Sebastian M Waszak[8], Joachim Weischenfeldt[8,111,112] and Christian von Mering[125,131]

Quality control working group

Sergi Beltran[132,133], Ivo Buchhalter[52,53,54], Peter J Campbell[1,2], Roland Eils[52,54,66,67], Daniela S Gerhard[134], Gad Getz[3,4,5,6], **Ivo G Gut#**[132,133], Marta Gut[132,133], Barbara Hutter[79,80,81], Daniel Hübschmann[54,66,82,83,84], Kortine Kleinheinz[52,54], Jan O Korbel[7,8], Dimitri Livitz[3], Marc D Perry[45,102], Keiran M Raine[1], Esther Rheinbay[3,6,104], Mara Rosenberg[3,104], Gordon Saksena[3], Matthias Schlesner[52,108], Miranda D Stobbe[132,133], Jean-Rémi Trotta[132], Johannes Werner[52,113] and Justin P Whalley[132]

Novel somatic mutation calling methods

Matthew H Bailey[135,136], Beifang Niu[137], Matthias Bieg[80,138], Paul C Boutros[9,130,139,140], Ivo Buchhalter[52,53,54], Adam P Butler[1], Ken Chen[141], Zechen Chong[142], **Li Ding#**[135,136,143], Oliver Drechsel[133,144], Lewis Jonathan Dursi[9,65], Roland Eils[52,54,66,67], Kyle Ellrott[51], Shadrielle MG Espiritu[9], Yu Fan[145], Robert S Fulton[135,136,143], Shengjie Gao[146], Josep Ll Gelpi[46,70], Mark Gerstein[118,119,120], Gad Getz[3,4,5,6], Santiago Gonzalez[7,8], Ivo G Gut[132,133], Faraz Hach[147,148], Michael C Heinold[52,54], Julian M Hess[3,74], Jonathan Hinton[1], Taobo Hu[149], Vincent Huang[9], Yi Huang[150,151], Barbara Hutter[79,80,81], David R Jones[1], Jongsun Jung[88], Natalie Jäger[52], Hyung-Lae Kim[27], Kortine Kleinheinz[52,54], Sushant Kumar[119,120], Yogesh Kumar[149], Christopher M Lalansingh[9], Ignaty Leshchiner[3], Ivica Letunic[152], Dimitri Livitz[3], Eric Z Ma[149], Yosef E Maruvka[3,74,104], R Jay Mashl[136,153], Michael D McLellan[135,136,143], Andrew Menzies[1], Ana Milovanovic[46], Morten Muhlig Nielsen[122], Stephan Ossowski[133,144,154], Nagarajan Paramasivam[52,80], Jakob Skou Pedersen[122,129], Marc D Perry[45,102], Montserrat Puiggròs[46], Keiran M Raine[1], Esther Rheinbay[3,6,104], Romina Royo[46], S Cenk Sahinalp[148,155,156], Gordon Saksena[3], Iman Sarrafi[148,156], Matthias Schlesner[52,108], **Jared T Simpson#**[9,157], Lucy Stebbings[1], Chip Stewart[3], Miranda D Stobbe[132,133], Jon W Teague[1], Grace Tiao[3], David Torrents[46,109], Jeremiah A Wala[3,6,49], Jiayin Wang[136,151,158], Wenyi Wang[145], Sebastian M Waszak[8], Joachim Weischenfeldt[8,111,112], Michael C Wendl[136,159,160], Johannes Werner[52,113], David A Wheeler[161,162], Zhenggang Wu[149], Hong Xue[149], Sergei Yakneen[8], Takafumi N Yamaguchi[9], Kai Ye[158,163], Venkata D Yellapantula[164,165], Christina K Yung[45] and Junjun Zhang[45]

Drivers and functional interpretation

Federico Abascal[1], Samirkumar B Amin[166,167,168], Gary D Bader[10], Pratiti Bandopadhayay[3,169,170], Jonathan Barenboim[9], Rameen Beroukhim[3,6,171], Johanna Bertl[122,172], Keith A Boroevich[47,48], Søren Brunak[173,174], John Busanovich[3,175], Peter J Campbell[1,2], Joana Carlevaro-Fita[176,177,178], Dimple Chakravarty[179], Calvin Wing Yiu Chan[52,180], Ken Chen[141], Jung Kyoon Choi[181], Jordi Deu-Pons[182,183], Priyanka Dhingra[115,116], Klev Diamanti[184], Lars Feuerbach[117], J Lynn Fink[46,185], Nuno A Fonseca[7,69], Joan Frigola[182], Carlo Gambacorti-Passerini[186], Dale W Garsed[187,188], **Mark Gerstein#**[118,119,120], **Gad Getz#**[3,4,5,6], Qianyun Guo[129], Ivo G Gut[132,133], David Haan[11], Mark P Hamilton[121], Nicholas J Haradhvala[3,104], Arif O Harmanci[120,189], Mohamed Helmy[190], Carl Herrmann[52,54,191], Julian M Hess[3,74], Asger Hobolth[129,172], Ermin Hodzic[156], Chen Hong[117,180], Henrik Hornshøj[122], Keren Isaev[9,130], Jose MG Izarzugaza[173], Rory Johnson[177,192], Todd A Johnson[47], Malene Juul[122], Randi Istrup Juul[122], Andre Kahles[89,90,91,92,93], Abdullah Kahraman[123,124,125], Manolis Kellis[3,126], Ekta Khurana[115,116,127,128], Jaegil Kim[3], Jong K Kim[193], Youngwook Kim[95,96], Jan Komorowski[184,194], Jan O Korbel[7,8], Sushant Kumar[119,120], Andrés Lanzós[177,178,192], Erik Larsson[89], **Michael S Lawrence#**[3,47,104], Donghoon Lee[120], Kjong-Van Lehmann[89,91,92,93,195], Shantao Li[120], Xiaotong Li[120], Ziao Lin[3,196], Eric Minwei Liu[115,116,197], Lucas Lochovsky[167,198,199,200], Shaoke Lou[119,120], Tobias Madsen[122], Kathleen Marchal[201,202], Iñigo Martincorena[1], Alexander Martinez-Fundichely[115,116,127], Yosef E Maruvka[3,74,104], Patrick D McGillivray[119], William Meyerson[120,203], Ferran Muiños[183,204], Loris Mularoni[183,204], Hidewaki Nakagawa[48], Morten Muhlig Nielsen[122], Marta Paczkowska[9], Keunchil Park[205,206], Kiejung Park[207], **Jakob Skou Pedersen#**[122,129], Tirso Pons[208], Sergio Pulido-Tamayo[201,202], **Benjamin J Raphael#**[209], Jüri Reimand[9,130], Iker Reyes-Salazar[204], Matthew A Reyna[209], Esther Rheinbay[3,6,104], Mark A Rubin[128,192,210,211,212], Carlota Rubio-Perez[183,204,213], S Cenk Sahinalp[148,155,156], Gordon Saksena[3], Leonidas Salichos[119,120], Chris Sander[49,89,214,215], Steven E Schumacher[3,216], Mark Shackleton[188,217], Ofer Shapira[3,49], Ciyue Shen[215,218], Raunak Shrestha[148], Shimin Shuai[9,10], Nikos Sidiropoulos[111], Lina Sieverling[117,180], Nasa Sinnott-Armstrong[3,61], Lincoln D Stein[9,10], **Joshua M Stuart#**[11], David Tamborero[183,204], Grace Tiao[3], Tatsuhiko Tsunoda[47,219,220,221], Husen M Umer[184,222], Liis Uusküla-Reimand[223,224], Alfonso Valencia[46,109], Miguel Vazquez[46,110], Lieven PC Verbeke[202,225], Claes Wadelius[226], Lina Wadi[9], Jiayin Wang[136,151,158], Jonathan Warrell[119,120], Sebastian M Waszak[8], Joachim Weischenfeldt[8,111,112], **David A Wheeler#**[161,162], Guanming Wu[227], Jun Yu[228, 229], Jing Zhang[120], Xuanping Zhang[151,230], Yan Zhang[120,231,232], Zhongming Zhao[233], Lihua Zou[234] and Christian von Mering[125,131]

Integration of transcriptome and genome

Samirkumar B Amin[166,167,168], Philip Awadalla[9,10], Peter J Bailey[235], **Alvis Brazma#**[7], **Angela N Brooks#**[3,49,50], Claudia Calabrese[7,8], Aurélien Chateigner[45], Isidro Cortés-Ciriano[236,237,238], Brian Craft[239], David Craft[3,240], Chad J Creighton[241], Natalie R Davidson[89,91,92,107,195], Deniz Demircioğlu[242,243], Serap Erkek[8], Nuno A Fonseca[7,69], Milana Frenkel-Morgenstern[244], Mary J Goldman[239], Liliana Greger[7], Jonathan Göke[242,245], Yao He[246], Katherine A Hoadley[247,248], Yong Hou[39,249], Matthew R Huska[250], Andre Kahles[89,90,91,92,93], Ekta Khurana[115,116,127,128], Helena Kilpinen[251], Jan O Korbel[7,8], Fabien C Lamaze[9], Kjong-Van Lehmann[89,91,92,93,195], Chang Li[39,249], Siliang Li[39,249], Xiaobo Li[39,249], Xinyue Li[39], Dongbing Liu[39,249], Fenglin Liu[246,252], Xingmin Liu[39,249], Maximillian G Marin[50], Julia Markowski[250], Matthew Meyerson[3,6,49,177,253], Tannistha Nandi[254], Morten Muhlig Nielsen[122], Akinyemi I Ojesina[255,256,257], BF Francis Ouellette[100,101], Qiang Pan-Hammarström[39,258], Peter J Park[237,259], Chandra Sekhar Pedamallu[3,6,171], Jakob Skou Pedersen[122,129], Marc D Perry[45,102], **Gunnar Rätsch#**[89,92,93,105,106,107], Roland F Schwarz[7,83,250,260], Yuichi Shiraishi[86], Reiner Siebert[261,262], Cameron M Soulette[50], Stefan G Stark[92,195,263,264], Oliver Stegle[7,8,265], Hong Su[39,249], Patrick Tan[254,266,267,268], Bin Tean Teh[266,267,268,269,270], Lara Urban[7,8], Jian Wang[39], Sebastian M Waszak[8], Kui Wu[39,249], Qian Xiang[114], Heng Xiong[39,249], Sergei Yakneen[8], Huanming Yang[39], Chen Ye[39,249], Christina K Yung[45], Fan Zhang[246], Junjun Zhang[45], Xiuqing Zhang[39], Zemin Zhang[246,271], Liangtao Zheng[246], Jingchun Zhu[239] and Shida Zhu[39,249]

Integration of epigenome and genome

Hiroyuki Aburatani[272], **Benjamin P Berman#**[273,274,275], Hans Binder[276,277], **Benedikt Brors#**[81,117,278], Huy Q Dinh[273], Lars Feuerbach[117], Shengjie Gao[146], Ivo G Gut[132,133], Simon C Heath[132,133], Steve Hoffmann[276,277,279,280], Charles D Imbusch[117], Ekta Khurana[115,116,127,128], Helene Kretzmer[277,280], Peter W Laird[281], Jose I Martin-Subero[109,282], Genta Nagae[272,283], **Christoph Plass#**[284], Paz Polak[3,4,6], Hui Shen[285], Reiner Siebert[261,262], Nasa Sinnott-Armstrong[3,61], Miranda D Stobbe[132,133], Qi Wang[97], Dieter Weichenhan[284], Sergei Yakneen[8] and Wanding Zhou[285]

Patterns of structural variations, signatures, genomic correlations, retrotransposons, mobile elements

Kadir C Akdemir[141], Eva G Alvarez[286,287,288], Adrian Baez-Ortega[289], **Rameen Beroukhim#**[3,6,171], Paul C Boutros[9,130,139,140], David D L Bowtell[187,290], Benedikt Brors[81,117,278], Kathleen H Burns[291], **Peter J Campbell#**[1,2], Kin Chan[292], Ken Chen[141], Isidro Cortés-Ciriano[236,237,238], Ana Dueso-Barroso[46], Andrew J Dunford[3], Paul A Edwards[293,294], Xavier Estivill[144,295], Dariush Etemadmoghadam[187,188], Lars Feuerbach[117], J Lynn Fink[46,185], Milana Frenkel-Morgenstern[244], Dale W Garsed[187,188], Mark Gerstein[118,119,120], Dmitry A Gordenin[296], David Haan[11], James E Haber[297], Julian M Hess[3,74], Barbara Hutter[79,80,81], Marcin Imielinski[298,299], David TW Jones[300,301], Young Seok Ju[1,181], Marat D Kazanov[302,303,304], Leszek J Klimczak[305], Youngil Koh[306,307], Jan O Korbel[7,8], Kiran Kumar[3], Eunjung Alice Lee[308], Jake June-Koo Lee[237,259], Yilong Li[1], Andy G Lynch[293,294,309], Geoff Macintyre[293], Florian Markowetz[293,294], Iñigo Martincorena[1], Alexander Martinez-Fundichely[115,116,127], Matthew Meyerson[3,6,49,177,253], Satoru Miyano[86], Hidewaki Nakagawa[48], Fabio CP Navarro[119], Stephan Ossowski[133,144,154], Peter J Park[237,259], John V Pearson[310,311], Montserrat Puiggròs[46], Karsten Rippe[83], Nicola D Roberts[1], Steven A Roberts[312], Bernardo Rodriguez-Martin[286,287,288], Steven E Schumacher[3,216], Ralph Scully[313], Mark Shackleton[188,217], Nikos Sidiropoulos[111], Lina Sieverling[117,180], Chip Stewart[3], David Torrents[46,109], Jose MC Tubio[286,287,288], Izar Villasante[46], Nicola Waddell[310,311], Jeremiah A Wala[3,6,49], Joachim Weischenfeldt[8,111,112], Lixing Yang[314], Xiaotong Yao[298,315], Sung-Soo Yoon[307], Jorge Zamora[1,286,287,288] and Cheng-Zhong Zhang[3,6,49]

Mutation signatures and processes

Ludmil B Alexandrov[1,316], Erik N Bergstrom[317], Arnoud Boot[267,318], Paul C Boutros[9,130,139,140], Kin Chan[292], Kyle Covington[162], Akihiro Fujimoto[48], Gad Getz[3,4,5,6], Dmitry A Gordenin[296], Nicholas J Haradhvala[3,104], Mi Ni Huang[267,318], S. M. Ashiqul Islam[316], Marat D Kazanov[302,303,304], Jaegil Kim[3], Leszek J Klimczak[305], Michael S Lawrence[3,47,104], Iñigo Martincorena[1], John R McPherson[267,318], Sandro Morganella[1], Ville Mustonen[319,320,321], Hidewaki Nakagawa[48], Avlin Wei Tian Ng[322], Serena Nik-Zainal[1,323,324,325], Paz Polak[3,4,6], Stephenie D Prokopec[9], Steven A Roberts[312], **Steven G Rozen#**[267,268,318], Radhakrishnan Sabarinathan[183,204,326], Natalie Saini[296], Tatsuhiro Shibata[33,34], Yuichi Shiraishi[86], **Michael Rudolf Stratton#**[1], **Bin Tean Teh#**[266,267,268,269,270], Ignacio Vázquez-García[1,164,327,328], Yang Wu[267,318], Fouad Yousif[9] and Willie Yu[329]

Germline cancer genome

Ludmil B Alexandrov[1,316], Eva G Alvarez[286,287,288], Adrian Baez-Ortega[289], Matthew H Bailey[135,136], Mattia Bosio[46,133,144], G Steven Bova[330], Alvis Brazma[7], Alicia L Bruzos[286,287,288], Ivo Buchhalter[52,53,54], Carlos D Bustamante[60,61], Atul J Butte[331], Andy Cafferkey[7], Claudia Calabrese[7,8], Peter J Campbell[1,2], Stephen J Chanock[332], Nilanjan Chatterjee[333,334], Jieming Chen[120,335], Francisco M De La Vega[59,59,60,61,62], Olivier Delaneau[336,337,338], German M Demidov[133,144,339], Anthony DiBiase[340], Li Ding[135,136,143], Oliver Drechsel[133,144], Lewis Jonathan Dursi[9,65], Douglas F Easton[341,342], Serap Erkek[8], Georgia Escaramis[144,343,344], **Xavier Estivill#**[144,295], Erik Garrison[1], Mark Gerstein[118,119,120], Gad Getz[3,4,5,6], Dmitry A Gordenin[296], Nina Habermann[8], Olivier Harismendy[72], Eoghan Harrington[345], Shuto Hayashi[86], Seong Gu Heo[346], José María Heredia-Genestar[347], Aliaksei Z Holik[144], Eun Pyo Hong[346], Xing Hua[332], Kuan-lin Huang[136,348], Seiya Imoto[85,86], Sissel Juul[345], Ekta

Khurana[115,116,127,128], Hyung-Lae Kim[27], Youngwook Kim[95,96], Leszek J Klimczak[305], **Jan O Korbel#**[7,8], Roelof Koster[349], Sushant Kumar[119,120], Ivica Letunic[152], Yilong Li[1], Tomas Marques-Bonet[109,132,347,350], R Jay Mashl[136,153], Simon Mayes[351], Michael D McLellan[135,136,143], Lisa Mirabello[332], Francesc Muyas[133,144,339], Hidewaki Nakagawa[48], Arcadi Navarro[109,132,347], Steven J Newhouse[7], Stephan Ossowski[133,144,154], Ji Wan Park[346], Esa Pitkänen[8], Aparna Prasad[133], Raquel Rabionet[133,144,352], Benjamin Raeder[8], Tobias Rausch[8], Steven A Roberts[312], Bernardo Rodriguez-Martin[286,287,288], Vasilisa A Rudneva[353], Gunnar Rätsch[89,92,93,105,106,107], Natalie Saini[296], Matthias Schlesner[52,108], Roland F Schwarz[7,83,250,260], Ayellet V Segre[3,3,354], Tal Shmaya[59], Suyash S Shringarpure[61], Nikos Sidiropoulos[111], Reiner Siebert[261,262], Jared T Simpson[9,157], Lei Song[332], Oliver Stegle[7,8,265], Hana Susak[133,144], Tomas J Tanskanen[355], Grace Tiao[3], Marta Tojo[288], Jose MC Tubio[286,287,288], Daniel J Turner[351], Lara Urban[7,8], Sebastian M Waszak[8], David C Wedge[1,356,357], Joachim Weischenfeldt[8,111,112], David A Wheeler[161,162], Mark H Wright[61], Dai-Ying Wu[59], Tian Xia[358], Sergei Yakneen[8], Kai Ye[158,163], Venkata D Yellapantula[164,165], Jorge Zamora[1,286,287,288] and Bin Zhu[332]

Tumor subtypes and clinical translation

Fatima Al-Shahrour[359], Gurnit Atwal[9,10,360], Peter J Bailey[235], **Andrew V Biankin#**[361,362,363,364], Paul C Boutros[9,130,139,140], Peter J Campbell[1,2], David K Chang[362,364], Susanna L Cooke[364], Vikram Deshpande[104], Bishoy M Faltas[107], William C Faquin[104], **Levi Garraway#**[49], Gad Getz[3,4,5,6], **Sean M Grimmond#**[365], Syed Haider[9], **Katherine A Hoadley#**[247,248], Wei Jiao[9], Vera B Kaiser[366], Rosa Karlić[367], Mamoru Kato[368], Kirsten Kübler[3,6,104], Alexander J Lazar[369], Constance H Li[9,130], David N Louis[104], Adam Margolin[370], Sancha Martin[1,371], Hardeep K Nahal-Bose[45], G Petur Nielsen[104], Serena Nik-Zainal[1,323,324,325], Larsson Omberg[99], Christine P'ng[9], Marc D Perry[45,102], Paz Polak[3,4,6], Esther Rheinbay[3,6,104], Mark A Rubin[128,192,210,211,212], Colin A Semple[366], Dennis C Sgroi[104], Tatsuhiro Shibata[33,34], Reiner Siebert[261,262], Jaclyn Smith[370], **Lincoln D Stein#**[9,10], Miranda D Stobbe[132,133], Ren X Sun[9], Kevin Thai[45], Derek W Wright[372,373], Chin-Lee Wu[104], and Junjun Zhang[45]

Evolution and heterogeneity

David J Adams[1], Pavana Anur[375], Rameen Beroukhim[3,6,171], Paul C Boutros[9,130,139,140], David D L Bowtell[187,290], Peter J Campbell[1,2], Shaolong Cao[145], Elizabeth L Christie[187], Yupeng Cun[379], Kevin J Dawson[1], Jonas Demeulemeester[63,64], Stefan C Dentro[1,64,356], Amit G Deshwar[380], Nilgun Donmez[148,156], Ruben M Drews[293], Roland Eils[52,54,66,67], Yu Fan[145], Matthew W Fittall[64], Dale W Garsed[187,188], Moritz Gerstung[7,8], Gad Getz[3,4,5,6], Santiago Gonzalez[7,8], Gavin Ha[3], Kerstin Haase[64], Marcin Imielinski[298,299], Lara Jerman[8,381], Yuan Ji[382,383], Clemency Jolly[64], Kortine Kleinheinz[52,54], Juhee Lee[384], Henry Lee-Six[1], Ignaty Leshchiner[3], Dimitri Livitz[3], Salem Malikic[148,156], Iñigo Martincorena[1], Thomas J Mitchell[1,294,385], Quaid D Morris[360,386], Ville Mustonen[319,320,321], Layla Oesper[387], Martin Peifer[379], Myron Peto[388], Benjamin J Raphael[209], Daniel Rosebrock[3], Yulia Rubanova[157,360], S Cenk Sahinalp[148,155,156], Adriana Salcedo[9], Matthias Schlesner[52,108], Steven E Schumacher[3,216], Subhajit Sengupta[389], Ruian Shi[386], Seung Jun Shin[264], **Paul T Spellman#**[390], Oliver Spiro[3], Lincoln D Stein[9,10], Maxime Tarabichi[1,64], **Peter Van Loo#**[63,64], Shankar Vembu[386,391], Ignacio Vázquez-García[1,164,327,328], Wenyi Wang[145], **David C Wedge#**[1,356,357], David A Wheeler[161,162], Jeffrey A Wintersinger[190,360,392], Tsun-Po Yang[379], Xiaotong Yao[298,315], Kaixian Yu[393], and Hongtu Zhu[394,395]

Exploratory: portals, visualization and software infrastructure

Fatima Al-Shahrour[359], Elisabet Barrera[7], Wojciech Bazant[7], Alvis Brazma[7], Isidro Cortés-Ciriano[236,237,238], Brian Craft[239], David Craft[3,240], Vincent Ferretti[45,68], Nuno A Fonseca[7,69], Anja Füllgrabe[7], Mary J Goldman[239], **David Haussler#**[239,396], Wolfgang Huber[8], Maria Keays[7], Alfonso Muñoz[7], Brian D O'Connor[45,50], Irene Papatheodorou[7], Robert Petryszak[7], Elena Piñeiro-Yáñez[359], Alfonso Valencia[46,109], **Miguel Vazquez#**[46,110], John N Weinstein[397,398], Qian Xiang[114], Junjun Zhang[45] and **Jingchun Zhu#**[239]

Exploratory: mitochondrial variants and HLA/immunogenicity

Peter J Campbell[1,2], Yiwen Chen[145], Chad J Creighton[241], Li Ding[135,136,143], Akihiro Fujimoto[48], Masashi Fujita[48], Gad Getz[3,4,5,6], Leng Han[230], Takanori Hasegawa[86], Shuto Hayashi[86], Seiya Imoto[85,86], Young Seok Ju[1,181], Hyung-Lae Kim[27], Youngwook Kim[95,96], Youngil Koh[306,307], Mitsuhiro Komura[86], Jun Li[145], **Han Liang#**[399], Iñigo Martincorena[1], Satoru Miyano[86], Shinichi Mizuno[400], **Hidewaki Nakagawa#**[48], Keunchil Park[205,206], Eigo Shimizu[86], Yumeng Wang[145,401], John N Weinstein[397,398], Yanxun Xu[402], Rui Yamaguchi[86], Fan Yang[386], Yang Yang[230], Christopher J Yoon[181], Sung-Soo Yoon[307], Yuan Yuan[145], Fan Zhang[246] and Zemin Zhang[246,271]

Exploratory: pathogens

Malik Alawi[403,404], Ivan Borozan[9], Daniel S Brewer[405,406], Colin S Cooper[406,407,408], Nikita Desai[45], **Roland Eils#**[52,54,66,67], Vincent Ferretti[45,68], Adam Grundhoff[403,409], Murat Iskar[410], Kortine Kleinheinz[52,54], **Peter Lichter#**[79,410], Hidewaki Nakagawa[48], Akinyemi I Ojesina[255,256,257], Chandra Sekhar Pedamallu[3,6,171], Matthias Schlesner[52,108], Xiaoping Su[141] and Marc Zapatka[410]

Tumor Specific Providers – Australia (Ovarian cancer)

Kathryn Alsop[411,412], Australian Ovarian Cancer Study Group[187,310,413], **David D L Bowtell#**[187,290], Timothy JC Bruxner[185], Angelika N Christ[185], Elizabeth L Christie[187], Stephen M Cordner[414], Prue A Cowin[187], Ronny Drapkin[415], Dariush Etemadmoghadam[187,188], Sian Fereday[416], Dale W Garsed[187,188], Joshy George[167], Sean M Grimmond[365], Anne Hamilton[187], Oliver Holmes[310,311], Jillian A Hung[417,418], Karin S Kassahn[185,419], Stephen H Kazakoff[310,311], Catherine J Kennedy[420,421], Conrad R Leonard[310,311], Linda Mileshkin[187], David K Miller[185,362,422], Gisela Mir Arnau[187], Chris Mitchell[187], Felicity Newell[310,311], Katia Nones[310,311], Ann-Marie Patch[310,311], John V Pearson[310,311], Michael C Quinn[310,311], Mark Shackleton[188,217], Darrin F Taylor[185], Heather Thorne[187], Nadia Traficante[187], Ravikiran Vedururu[187], Nick M Waddell[311], Nicola Waddell[310,311], Paul M Waring[253], Scott Wood[310,311], Qinying Xu[310,311] and Anna deFazio[423,424,425]

Tumor Specific Providers – Australia (Pancreatic cancer)

Matthew J Anderson[185], Davide Antonello[426], Andrew P Barbour[427,428], Claudio Bassi[426], Samantha Bersani[429], **Andrew V Biankin#**[361,362,363,364], Timothy JC Bruxner[185], Ivana Cataldo[429,430], David K Chang[362,364], Lorraine A Chantrill[362], Yoke-Eng Chiew[423], Angela Chou[362,431], Angelika N Christ[185], Sara Cingarlini[37], Nicole Cloonan[432], Vincenzo Corbo[430,433, 434], Fraser R Duthie[435,436], J Lynn Fink[46,185], Anthony J Gill[362,437], Janet S Graham[364,438], **Sean M Grimmond#**[365], Ivon Harliwong[185], Oliver Holmes[310,311], Nigel B Jamieson[363,364,439], Amber L Johns[362,422], Karin S Kassahn[185,419], Stephen H Kazakoff[310,311], James G Kench[362,437,440], Luca Landoni[426], Rita T Lawlor[430], Conrad R Leonard[310,311], Andrea Mafficini[430], Neil D Merrett[426,441], David K Miller[185,362,422], Marco Miotto[426], Elizabeth A Musgrove[364], Adnan M Nagrial[362], Felicity Newell[310,311], Katia Nones[310,311], Karin A Oien[253,442], Marina Pajic[362], Ann-Marie Patch[310,311], John V Pearson[310,311], Mark Pinese[362], Andreia V Pinho[362], Michael C Quinn[310,311], Alan J Robertson[185], Ilse Rooman[362], Borislav C Rusev[430], Jaswinder S Samra[426,437], Maria Scardoni[429], Christopher J Scarlett[362,443], Aldo Scarpa[430], Elisabetta Sereni[426], Katarzyna O Sikora[430], Michele Simbolo[433], Morgan L Taschuk[45], Christopher W Toon[362], Giampaolo Tortora[37,38], Caterina Vicentini[430], Nick M Waddell[311], Nicola Waddell[310,311], Scott Wood[310,311], Jianmin Wu[362], Qinying Xu[310,311] and Nikolajs Zeps[444]

Tumor Specific Providers – Australia (Skin cancer)

Lauri A Aaltonen[445], Andreas Behren[446], Hazel Burke[447], Jonathan Cebon[446], Rebecca A Dagg[448], Ricardo De Paoli-Iseppi[449], Ken Dutton-Regester[310], Matthew A Field[450], Anna Fitzgerald[451], Sean M Grimmond[365], **Nicholas K Hayward#**[310,447], Peter Hersey[447],

Oliver Holmes[310,311], Valerie Jakrot[447], Peter A Johansson[310], Hojabr Kakavand[449], Stephen H Kazakoff[310,311], Richard F Kefford[452], Loretta MS Lau[453], Conrad R Leonard[310,311], Georgina V Long[454], **Graham J Mann#**[455,456], Felicity Newell[310,311], Katia Nones[310,311], Ann-Marie Patch[310,311], John V Pearson[310,311], Hilda A Pickett[453], Antonia L Pritchard[310], Gulietta M Pupo[457], Robyn PM Saw[454], Sarah-Jane Schramm[458], **Richard A Scolyer#**[424,454,459,460], Mark Shackleton[188,217], Catherine A Shang[461], Ping Shang[454], Andrew J Spillane[454], Jonathan R Stretch[454], Varsha Tembe[458], John F Thompson[454], Ricardo E Vilain[459], Nick M Waddell[311], Nicola Waddell[310,311], James S Wilmott[454], Scott Wood[310,311], Qinying Xu[310,311] and Jean Y Yang[462]

Tumor Specific Providers – Canada (Pancreatic cancer)

John Bartlett[463,464], Prashant Bavi[465], Ivan Borozan[9], Dianne E Chadwick[466], Michelle Chan-Seng-Yue[465], Sean Cleary[465,467], Ashton A Connor[468,469], Karolina Czajka[470], Robert E Denroche[465], Neesha C Dhani[471], Jenna Eagles[78], Vincent Ferretti[45,68], Steven Gallinger[465,468,469], Robert C Grant[465,472], David Hedley[471], Michael A Hollingsworth[473], **Thomas J Hudson#**[77,78], Gun Ho Jang[465], Jeremy Johns[78], Sangeetha Kalimuthu[465], Sheng-Ben Liang[474], Ilinca Lungu[465,475], Xuemei Luo[9], Faridah Mbabaali[78], **John D McPherson#**[78,465,476], Treasa A McPherson[472], Jessica K Miller[78], Malcolm J Moore[471], Faiyaz Notta[465,477], Danielle Pasternack[78], Gloria M Petersen[478], Michael H A Roehrl[130,465,479,480,481], Michelle Sam[78], Iris Selander[472], Stefano Serra[253], Sagedeh Shahabi[474], **Lincoln D Stein#**[9,10], Morgan L Taschuk[45], Sarah P Thayer[104], Lee E Timms[78], Gavin W Wilson[9,465], Julie M Wilson[465] and Bradly G Wouters[482]

Tumor Specific Providers – Canada (Prostate cancer)

Timothy A Beck[45], Vinayak Bhandari[9], Paul C Boutros[9,130,139,140], **Robert G Bristow#**[130,483,484,485,486], Colin C Collins[148], Shadrielle MG Espiritu[9], Neil E Fleshner[487], Natalie S Fox[9], Michael Fraser[9], Syed Haider[9], Lawrence E Heisler[488], Vincent Huang[9], Emilie Lalonde[9], Julie Livingstone[9], John D McPherson[78,465,476], Alice Meng[489], Veronica Y Sabelnykova[9], Adriana Salcedo[9], Yu-Jia Shiah[9], Theodorus Van der Kwast[490] and Takafumi N Yamaguchi[9]

Tumor Specific Providers – China (Gastric cancer)

Shuai Ding[491], Daiming Fan[492], Yong Hou[39,249], Yi Huang[150,151], Lin Li[39], Siliang Li[39,249], Dongbing Liu[39,249], Xingmin Liu[39,249], **Youyong Lu#**[28,29,30], Yongzhan Nie[492,493], Hong Su[39,249], Jian Wang[39], Kui Wu[39,249], Xiao Xiao[151], Rui Xing[29,494], **Huanming Yang#**[39], Shanlin Yang[491], Yingyan Yu[495], [229], Xiuqing Zhang[39], Yong Zhou[39] and Shida Zhu[39,249]

Tumor Specific Providers – EU: France (Renal cancer)

Rosamonde E Banks[496], Guillaume Bourque[497,498], Alvis Brazma[7], Paul Brennan[499], **Mark Lathrop#**[498], Louis Letourneau[500], Yasser Riazalhosseini[498], Ghislaine Scelo[499], **Jörg Tost#**[501], Naveen Vasudev[502] and Juris Viksna[503]

Tumor Specific Providers – EU: United Kingdom (Breast cancer)

Sung-Min Ahn[504], Ludmil B Alexandrov[1,316], Samuel Aparicio[505], Laurent Arnould[506], MR Aure[507], Shriram G Bhosle[1], E Birney[7], Ake Borg[508], S Boyault[509], AB Brinkman[510], JE Brock[511], A Broeks[512], Adam P Butler[1], AL Børresen-Dale[507], C Caldas[513,514], Peter J Campbell[1,2], Suet-Feung Chin[513,514], Helen Davies[1], C Desmedt[515], L Dirix[516], S Dronov[1], Anna Ehinger[517], JE Eyfjord[518], GG Van den Eynden[519], A Fatima[216], Jorge Reis Filho[520], JA Foekens[521], PA Futreal[522], Øystein Garred[523,524], Moritz Gerstung[7,8], Dilip D Giri[520], D Glodzik[1], Dorthe Grabau[525], Holmfridur Hilmarsdottir[518], GK Hooijer[526], Jocelyne Jacquemier[527], SJ Jang[528], Jon G Jonasson[518], Jos Jonkers[529], HY Kim[527], Tari A King[530,531], Stian Knappskog[1], G Kong[527], S Krishnamurthy[532], S Van Laere[516], SR

Lakhani[533], A Langerød[507], Denis Larsimont[534], HJ Lee[528], JY Lee[535], Ming Ta Michael Lee[522], Yilong Li[1], Ole Christian Lingjærde[536], Gaetan MacGrogan[537], JW Martens[538], Sancha Martin[1,371], Iñigo Martincorena[1], Andrew Menzies[1], Sandro Morganella[1], Ville Mustonen[319,320,321], Serena Nik-Zainal[1,323,324,325], Sarah O'Meara[1], I Pauporté[18], Sarah Pinder[539], X Pivot[540], Elena Provenzano[541], CA Purdie[542], Keiran M Raine[1], M Ramakrishna[1], K Ramakrishnan[1], AL Richardson[216], M Ringnér[508], Javier Bartolomé Rodriguez[46], FG Rodríguez-González[174], G Romieu[543], Roberto Salgado[253], Torill Sauer[536], R Shepherd[1], AM Sieuwerts[177], PT Simpson[533], M Smid[544], C Sotiriou[55], PN Span[545], J Staaf[508], Lucy Stebbings[1], Ólafur Andri Stefánsson[546], Alasdair Stenhouse[547], **Michael Rudolf Stratton#**[1], HG Stunnenberg[249,548], Fred Sweep[549], BK Tan[550], Jon W Teague[1], Gilles Thomas[551], AM Thompson[547], S Tommasi[552], I Treilleux[553,554], Andrew Tutt[216], NT Ueno[395], Peter Van Loo[63,64], P Vermeulen[516], Alain Viari[430], MJ van de Vijver[253], A Vincent-Salomon[548], David C Wedge[1,356,357], Bernice Huimin Wong[555], Lucy Yates[1], X Zou[1], CHM van Deurzen[538] and L van't Veer[556,557]

Tumor Specific Providers – Germany (Malignant lymphoma)

Ole Ammerpohl[558,559], Sietse Aukema[560,561], Anke K Bergmann[562], Stephan H Bernhart[276,277,280], Hans Binder[276,277], Arndt Borkhardt[563], Christoph Borst[564], Benedikt Brors[81,117,278], Birgit Burkhardt[565], Alexander Claviez[566], Roland Eils[52,54,66,67], Maria Elisabeth Goebler[567], Andrea Haake[558], Siegfried Haas[564], Martin Hansmann[568], Jessica I Hoell[563], Steve Hoffmann[276,277,279,280], Michael Hummel[569], Daniel Hübschmann[54,66,82,83,84], Dennis Karsch[570], Wolfram Klapper[561], Kortine Kleinheinz[52,54], Michael Kneba[570], Jan O Korbel[7,8], Helene Kretzmer[277,280], Markus Kreuz[571], Dieter Kube[572], Ralf Küppers[573], Chris Lawerenz[67], Dido Lenze[569], Peter Lichter[79,410], Markus Loeffler[571], Cristina López[262,558], Luisa Mantovani-Löffler[574], Peter Möller[575], German Ott[576], Bernhard Radlwimmer[410], Julia Richter[558,561], Marius Rohde[577], Philip C Rosenstiel[578], Andreas Rosenwald[579], Markus B Schilhabel[578], Matthias Schlesner[52,108], Stefan Schreiber[580], **Reiner Siebert#**[261,262], Peter F Stadler[276,277,280], Peter Staib[581], Stephan Stilgenbauer[582], Stephanie Sungalee[8], Monika Szczepanowski[561], Umut H Toprak[54,583], Lorenz HP Trümper[572], Rabea Wagener[262,558] and Thorsten Zenz[81]

Tumor Specific Providers – Germany (Pediatric Brain cancer)

Ivo Buchhalter[52,53,54], Juergen Eils[66,67], Roland Eils[52,54,66,67], Volker Hovestadt[410], Barbara Hutter[79,80,81], David TW Jones[300,301], Natalie Jäger[52], Christof von Kalle[83], Marcel Kool[97,300], Jan O Korbel[7,8], Andrey Korshunov[97], Pablo Landgraf[584], Chris Lawerenz[67], Hans Lehrach[585], **Peter Lichter#**[79,410], Paul A Northcott[586], Stefan M Pfister[97,300,587], Bernhard Radlwimmer[410], Guido Reifenberger[584], Matthias Schlesner[52,108], Hans-Jörg Warnatz[585], Joachim Weischenfeldt[8,111,112], Stephan Wolf[588], Marie-Laure Yaspo[585] and Marc Zapatka[410]

Tumor Specific Providers – Germany (Prostate cancer)

Yassen Assenov[589], Benedikt Brors[81,117,278], Juergen Eils[66,67], Roland Eils[52,54,66,67], Lars Feuerbach[117], Clarissa Gerhauser[284], Jan O Korbel[7,8], Chris Lawerenz[67], Hans Lehrach[585], Sarah Minner[590], Christoph Plass[284], **Guido Sauter#**[591], Thorsten Schlomm[112,592], Nikos Sidiropoulos[111], Ronald Simon[591], **Holger Sültmann#**[81,593], Hans-Jörg Warnatz[585], Dieter Weichenhan[284], Joachim Weischenfeldt[8,111,112] and Marie-Laure Yaspo[585]

Tumor Specific Providers – India (Oral cancer)

Nidhan K Biswas[594], Luca Landoni[426], Arindam Maitra[594], **Partha P Majumder#**[594] and **Rajiv Sarin#**[595]

Tumor Specific Providers – Italy (Pancreatic cancer)

Davide Antonello[426], Stefano Barbi[433], Claudio Bassi[426], Samantha Bersani[429], Giada Bonizzato[430], Cinzia Cantù[430], Ivana Cataldo[429,430], Sara Cingarlini[37], Vincenzo Corbo[430,433], [434], Angelo

P Dei Tos[596], Matteo Fassan[597], Sonia Grimaldi[430], Luca Landoni[426], Rita T Lawlor[430], Claudio Luchini[429], Andrea Mafficini[430], Giuseppe Malleo[426], Giovanni Marchegiani[426], Michele Milella[37], Marco Miotto[426], Salvatore Paiella[426], Antonio Pea[426], Paolo Pederzoli[426], Borislav C Rusev[430], Andrea Ruzzenente[426], Roberto Salvia[426], Maria Scardoni[429], **Aldo Scarpa#**[430], Elisabetta Sereni[426], Michele Simbolo[433], Nicola Sperandio[430], Giampaolo Tortora[37,38] and Caterina Vicentini[430]

Tumor Specific Providers – Japan (Biliary tract cancer)

Yasuhito Arai[33], Natsuko Hama[33], Nobuyoshi Hiraoka[598], Fumie Hosoda[33,599], Mamoru Kato[368], Hiromi Nakamura[33], Hidenori Ojima[600], Takuji Okusaka[601], **Tatsuhiro Shibata#**[33,34], Yasushi Totoki[33] and Tomoko Urushidate[34]

Tumor Specific Providers – Japan (Gastric cancer)

Hiroyuki Aburatani#[272], Yasuhito Arai[33], Masashi Fukayama[602], Natsuko Hama[33], Fumie Hosoda[33,599], Shumpei Ishikawa[603], Hitoshi Katai[604], Mamoru Kato[368], Hiroto Katoh[605], Daisuke Komura[603], Genta Nagae[272,283], Hiromi Nakamura[33], Hirofumi Rokutan[606], Mihoko Saito-Adachi[33], **Tatsuhiro Shibata#**[33,34], Akihiro Suzuki[272,607], Hirokazu Taniguchi[608], Kenji Tatsuno[272], Yasushi Totoki[33], Tetsuo Ushiku[602], Shinichi Yachida[33,609] and Shogo Yamamoto[272]

Tumor Specific Providers – Japan (Liver cancer)

Hiroyuki Aburatani[272], Hiroshi Aikata[610], Koji Arihiro[610], Shun-ichi Ariizumi[611], Keith A Boroevich[47,48], Kazuaki Chayama[610], Akihiro Fujimoto[48], Masashi Fujita[48], Mayuko Furuta[48], Kunihito Gotoh[612], Natsuko Hama[33], Takanori Hasegawa[86], Shinya Hayami[613], Shuto Hayashi[86], Satoshi Hirano[614], Seiya Imoto[85,86], Mamoru Kato[368], Yoshiiku Kawakami[610], Kazuhiro Maejima[48], Satoru Miyano[86], Genta Nagae[272,283], **Hidewaki Nakagawa#**[48], Hiromi Nakamura[33], Toru Nakamura[614], Kaoru Nakano[48], Hideki Ohdan[610], Aya Sasaki-Oku[48], **Tatsuhiro Shibata#**[33,34], Yuichi Shiraishi[86], Hiroko Tanaka[86], Yasushi Totoki[33], Tatsuhiko Tsunoda[47,219,220,221], Masaki Ueno[613], Rui Yamaguchi[86], Masakazu Yamamoto[611] and Hiroki Yamaue[613]

Tumor Specific Providers – Singapore (Biliary tract cancer)

Su Pin Choo[615], Ioana Cutcutache[267,318], Narong Khuntikeo[426,616], John R McPherson[267,318], Choon Kiat Ong[617], Chawalit Pairojkul[253], Irinel Popescu[618], **Steven G Rozen#**[267,268,318], **Patrick Tan#**[254,266,267,268] and **Bin Tean Teh#**[266,267,268,269,270]

Tumor Specific Providers – South Korea (Blood cancer)

Keun Soo Ahn[619], Hyung-Lae Kim[27], Youngil Koh[306,307] and **Sung-Soo Yoon#**[307]

Tumor Specific Providers – Spain (Chronic Lymphocytic Leukemia)

Marta Aymerich[620], **Elias Campo#**[621,622], Josep Ll Gelpi[46,70], Ivo G Gut[132,133], Marta Gut[132,133], Armando Lopez-Guillermo[623], Carlos López-Otín[624], Xose S Puente[625], Romina Royo[46] and David Torrents[46,109]

Tumor Specific Providers – United Kingdom (Bone cancer)

Fernanda Amary[626], Daniel Baumhoer[627], Sam Behjati[1], Bodil Bjerkehagen[628], **Peter J Campbell#**[1,2], **Adrienne M Flanagan#**[629], PA Futreal[522], Ola Myklebost[630], Nischalan Pillay[631], Patrick Tarpey[632], Roberto Tirabosco[633] and Olga Zaikova[634]

Tumor Specific Providers – United Kingdom (Chronic myeloid disorders)

Jacqueline Boultwood[635], David T Bowen[1], Adam P Butler[1], **Peter J Campbell#**[1,2], Mario Cazzola[636], Carlo Gambacorti-Passerini[186], Anthony R Green[294], Eva Hellstrom-Lindberg[637], Luca Malcovati[636], Sancha Martin[1,371], Jyoti Nangalia[638], Elli Papaemmanuil[1] and Paresh Vyas[310,639]

Tumor Specific Providers – United Kingdom (Esophageal cancer)

Yeng Ang[640], Hugh Barr[641], Duncan Beardsmore[642], Matthew Eldridge[293], **Rebecca C Fitzgerald#**[324], James Gossage[643], Nicola Grehan[324], George B Hanna[644], Stephen J Hayes[645,646], Ted R Hupp[647], David Khoo[648], Jesper Lagergren[637,649], Laurence E Lovat[251], Shona MacRae[397], Maria O'Donovan[324], J Robert O'Neill[650], Simon L Parsons[651], Shaun R Preston[652], Sonia Puig[653], Tom Roques[654], Grant Sanders[248], Sharmila Sothi[655], Simon Tavaré[293], Olga Tucker[656], Richard Turkington[657], Timothy J Underwood[658] and Ian Welch[659]

Tumor Specific Providers – United Kingdom (Prostate cancer)

Nicholas Van As[660], Daniel M Berney[661], Johann S De Bono[407], G Steven Bova[330], Daniel S Brewer[405,406], Adam P Butler[1], Declan Cahill[660], Niedzica Camacho[407], **Colin S Cooper#**[406,407,408], Nening M Dennis[660], Tim Dudderidge[660], Sandra E Edwards[407], **Rosalind A Eeles#**[407,660], Cyril Fisher[660], Christopher S Foster[662,663], Mohammed Ghori[1], Pelvender Gill[639], Vincent J Gnanapragasam[385,664], Gunes Gundem[1], Freddie C Hamdy[665], Steve Hawkins[293], Steven Hazell[660], William Howat[385], William B Isaacs[291], Katalin Karaszi[639], Jonathan D Kay[251], Vincent Khoo[660], Zsofia Kote-Jarai[407], Barbara Kremeyer[1], Pardeep Kumar[660], Adam Lambert[639], Daniel A Leongamornlert[1,407], Naomi Livni[660], Hayley J Luxton[251], Andy G Lynch[293,294,309], Luke Marsden[639], Charlie E Massie[293], Lucy Matthews[407], Erik Mayer[660,666], Ultan McDermott[1], Sue Merson[407], Thomas J Mitchell[1,294,385], David E Neal[293,385], Anthony Ng[667], David Nicol[660], Christopher Ogden[660], Edward W Rowe[660], Nimish C Shah[385], Jon W Teague[1], Sarah Thomas[660], Alan Thompson[660], Peter Van Loo[63,64], Clare Verrill[639,668], Tapio Visakorpi[330], Anne Y Warren[385,669], David C Wedge[1,356,357], Hayley C Whitaker[251], Yong-Jie Yu[661], Yongwei Yu[229], Jorge Zamora[1,286,287,288] and Hongwei Zhang[229]

Tumor Specific Providers – United States (TCGA)

Adam Abeshouse[197], Nishant Agrawal[71], Rehan Akbani[324,670], Hikmat Al-Ahmadie[197], Monique Albert[464], Kenneth Aldape[253,648,671], Adrian Ally[672], Yeng Ang[640], Elizabeth L Appelbaum[136,251], Joshua Armenia[673], Sylvia Asa[651,674], J Todd Auman[675], Matthew H Bailey[135,136], Miruna Balasundaram[672], Saianand Balu[248], Jill Barnholtz-Sloan[676,677], Hugh Barr[641], John Bartlett[463,464], Oliver F Bathe[678,679], Stephen B Baylin[658,680], Duncan Beardsmore[642], Christopher Benz[681], Andrew Berchuck[682], Benjamin P Berman[273,274,275], Rameen Beroukhim[3,6,171], Mario Berrios[683], Darell Bigner[293,684], Michael Birrer[104], Tom Bodenheimer[248], Lori Boice[653], Moiz S Bootwalla[685], Marcus Bosenberg[686], Reanne Bowlby[672], Jeffrey Boyd[687], Russell R Broaddus[671], Malcolm Brock[688], Denise Brooks[672], Susan Bullman[3,171], Samantha J Caesar-Johnson[40], Thomas E Carey[689], Rebecca Carlsen[672], Robert Cerfolio[690], Vishal S Chandan[691], Hsiao-Wei Chen[640,673], Andrew D Cherniack[3,3,49,171], Jeremy Chien[692], Juok Cho[3], Eric Chuah[672], Carrie Cibulskis[3], Kristian Cibulskis[3], Leslie Cope[693], Matthew G Cordes[136,654], Kyle Covington[162], Erin Curley[694], Bogdan Czerniak[648,671], Ludmila Danilova[693], Ian J Davis[695], Timothy Defreitas[3], John A Demchok[40], Noreen Dhalla[672], Rajiv Dhir[696], Li Ding[135,136,143], HarshaVardhan Doddapaneni[162], Adel El-Naggar[648,671], Ina Felau[40], Martin L Ferguson[697], Gaetano Finocchiaro[698], Kwun M Fong[699], Scott Frazer[3], William Friedman[700], Catrina C Fronick[136,654], Lucinda A Fulton[136], Robert S Fulton[135,136,143], Stacey B Gabriel[3], Jianjiong Gao[673], Nils Gehlenborg[3,701], Jeffrey E Gershenwald[702,703], Gad Getz[3,4,5,6], Ronald Ghossein[520], Nasra H Giama[704], Richard A Gibbs[162], Carmen Gomez[705], James Gossage[643], Ramaswamy Govindan[135], Nicola Grehan[324], George B Hanna[644], D Neil Hayes[248,706,707], Stephen J Hayes[645,646], Apurva M Hegde[397,670],

David I Heiman[3], Zachary Heins[197], Austin J Hepperla[248], Katherine A Hoadley[247,248], Andrea Holbrook[708], Robert A Holt[672], Alan P Hoyle[248], Ralph H Hruban[709], Jianhong Hu[162], Mei Huang[653], David Huntsman[710], Ted R Hupp[647], Jason Huse[197], **Carolyn M Hutter#**[21], Christine A Iacobuzio-Donahue[520], Michael Ittmann[711,712], Joy C Jayaseelan[162], Stuart R Jefferys[248], Corbin D Jones[713], Steven JM Jones[714], Hartmut Juhl[715], Koo Jeong Kang[716], Beth Karlan[717], Katayoon Kasaian[718], Electron Kebebew[719,720], David Khoo[648], Hark Kim[31], Jaegil Kim[3], Tari A King[530,531], Viktoriya Korchina[162], Ritika Kundra[640,673], Jesper Lagergren[637,649], Phillip H Lai[708], Peter W Laird[281], Eric Lander[3], Michael S Lawrence[3,47,104], Alexander J Lazar[369], Xuan Le[721], Darlene Lee[672], Douglas A Levine[197,722], Lora Lewis[162], Tim Ley[723], Haiyan Irene Li[672], Pei Lin[3], W Marston Linehan[724], Eric Minwei Liu[115,116,197], Fei Fei Liu[386], Laurence E Lovat[251], Yiling Lu[725], Lisa Lype[726], Yussanne Ma[672], Shona MacRae[397], Dennis T Maglinte[708], Elaine R Mardis[136,687,727], Jeffrey Marks[426,728], Marco A Marra[672], Thomas J Matthew[50], Michael Mayo[672], Karen McCune[729], Michael D McLellan[135,136,143], Samuel R Meier[3], Shaowu Meng[248], Matthew Meyerson[3,6,49,177,253], Piotr A Mieczkowski[247], Tom Mikkelsen[730], Christopher A Miller[136], Gordon B Mills[370,397,670], Richard A Moore[672], Carl Morrison[253,731], Lisle E Mose[248], Catherine D Moser[704], Andrew J Mungall[672], Karen Mungall[672], David Mutch[732], Donna M Muzny[162], Jerome Myers[733], Yulia Newton[50], Michael S Noble[3], Peter O'Donnell[734], Brian Patrick O'Neill[735], Angelica Ochoa[197], Akinyemi I Ojesina[255,256,257], Joong Won Park[31], Joel S Parker[736], Simon L Parsons[651], Harvey Pass[737], Alessandro Pastore[89], Chandra Sekhar Pedamallu[3,6,171], Nathan A Pennell[738], Charles M Perou[739], Gloria M Petersen[478], Nicholas Petrelli[740], Olga Potapova[741], Shaun R Preston[652], Sonia Puig[653], Janet S Rader[742], Suresh Ramalingam[743], W Kimryn Rathmell[744], Victor Reuter[253,520], Sheila M Reynolds[726], Matthew Ringel[745], Jeffrey Roach[746], Lewis R Roberts[704], A Gordon Robertson[672], Tom Roques[654], Mark A Rubin[128,192,210,211,212], Sara Sadeghi[672], Gordon Saksena[3], Charles Saller[747], Francisco Sanchez-Vega[640,673], Chris Sander[49,89,214,215], Grant Sanders[248], Dirk Schadendorf[79,748], Jacqueline E Schein[672], Heather K Schmidt[136], Nikolaus Schultz[673], Steven E Schumacher[3,216], Richard A Scolyer[424,454,459,460], Raja Seethala[749], Yasin Senbabaoglu[89], Troy Shelton[694], Yan Shi[248], Juliann Shih[3,171,177], Ilya Shmulevich[726], Craig Shriver[750], Sabina Signoretti[171,177,751], Janae V Simons[248], Samuel Singer[426,752], Payal Sipahimalani[672], Tara J Skelly[247], Karen Smith-McCune[729], Nicholas D Socci[89], Heidi J Sofia[21], Matthew G Soloway[736], Anil K Sood[753], Sharmila Sothi[655], Angela Tam[672], Donghui Tan[247], Roy Tarnuzzer[40], Nina Thiessen[672], R Houston Thompson[754], Leigh B Thorne[653], Ming Tsao[651,674], Olga Tucker[656], Richard Turkington[657], Christopher Umbricht[642,755], Timothy J Underwood[658], David J Van Den Berg[683], Erwin G Van Meir[756], Umadevi Veluvolu[247], Douglas Voet[3], Jiayin Wang[136,151,158], Linghua Wang[162], Zhining Wang[40], Paul Weinberger[757], John N Weinstein[397,398], Daniel J Weisenberger[708], Ian Welch[659], David A Wheeler[161,162], Dennis Wigle[758], Matthew D Wilkerson[247], Richard K Wilson[136,759], Boris Winterhoff[760], Maciej Wiznerowicz[761,762], Tina Wong[136,672], Winghing Wong[136], Liu Xi[162], Liming Yang[40], Christina Yau[293,681,682], Venkata D Yellapantula[164,165], **Jean C Zenklusen#**[40], Hailei Zhang[3], Hongxin Zhang[673] and Jiashan Zhang[40]

# Denotes **working group or project co-leader**

Author Affiliations

1. Wellcome Sanger Institute, Wellcome Genome Campus, Hinxton, Cambridge, CB10 1SA, UK.

2. Department of Haematology, University of Cambridge, Cambridge CB2 2XY, UK.

3. Broad Institute of MIT and Harvard, Cambridge, MA 02142, USA.

4. Center for Cancer Research, Massachusetts General Hospital, Boston, MA 02129, USA.

5. Department of Pathology, Massachusetts General Hospital, Boston, MA 02115, USA.

6. Harvard Medical School, Boston, MA 02115, USA.

7. European Molecular Biology Laboratory, European Bioinformatics Institute (EMBL-EBI), Wellcome Genome Campus, Hinxton, Cambridge, CB10 1SD, UK.

8. Genome Biology Unit, European Molecular Biology Laboratory (EMBL), Heidelberg 69117, Germany.

9. Computational Biology Program, Ontario Institute for Cancer Research, Toronto, ON M5G 0A3, Canada.

10. Department of Molecular Genetics, University of Toronto, Toronto, ON M5S 1A8, Canada.

11. Biomolecular Engineering Department, University of California, Santa Cruz, Santa Cruz, CA 95064, USA.

12. King Faisal Specialist Hospital and Research Centre, Al Maather, Riyadh 12713, Saudi Arabia.

13. DLR Project Management Agency, Bonn 53227, Germany.

14. Genome Canada, Ottawa, ON K2P 1P1, Canada.

15. Instituto Carlos Slim de la Salud, Mexico City, Mexico.

16. Federal Ministry of Education and Research, Berlin 10117, Germany.

17. Institut Gustave Roussy, Villejuif 94805, France.

18. Institut National du Cancer (INCA), Boulogne-Billancourt 92100, France.

19. The Wellcome Trust, London NW1 2BE, UK.

20. Prostate Cancer Canada, Toronto, ON M5C 1M1, Canada.

21. National Human Genome Research Institute, National Institutes of Health, Bethesda, MD 20892, USA.

22. Department of Biotechnology, Ministry of Science & Technology, Government of India, New Delhi, Delhi 110003, India.

23. Science Writer, Garrett Park, MD 20896, USA.

24. International Cancer Genome Consortium (ICGC)/ICGC Accelerating Research in Genomic Oncology (ARGO) Secretariat, Toronto, ON M5G 0A3, Canada.

25. Adaptive Oncology Initiative, Ontario Institute for Cancer Research, Toronto, ON M5G 0A3, Canada.

26. Cancer Research UK, London EC1V 4AD, UK.

27. Department of Biochemistry, College of Medicine, Ewha Womans University, Seoul 07895, South Korea.

28. Chinese Cancer Genome Consortium, Shenzhen 518083, China.

29. Laboratory of Molecular Oncology, Beijing,100142, China.

30. Peking University Cancer Hospital & Institute, Key Laboratory of Carcinogenesis and Translational Research (Ministry of Education), Peking University Cancer Hospital & Institute, Beijing 100142, China.

31. National Cancer Center, Tokyo 104-0045, Japan.

32. German Cancer Aid, Bonn 53113, Germany.

33. Division of Cancer Genomics, National Cancer Center Research Institute, Tokyo 104-0045, Japan.

34. Laboratory of Molecular Medicine, Human Genome Center, The Institute of Medical Science, The University of Tokyo, Minato-ku, Tokyo 108-8639, Japan.

35. Japan Agency for Medical Research and Development, Chiyoda-ku, Tokyo 100-0004 Japan.

36. Japan Agency for Medical Research and Development, Chiyoda-ku, Tokyo 100-0004, Japan.

37. Medical Oncology, University and Hospital Trust of Verona, Verona 37134, Italy.

38. University of Verona, Verona 37129, Italy.

39. BGI-Shenzhen, Shenzhen 518083, China.

40. National Cancer Institute, National Institutes of Health, Bethesda, MD 20892, USA.

41. Centre for Law and Genetics, University of Tasmania, Sandy Bay Campus, Hobart, Tasmania 7001 Australia.

42. Centre of Genomics and Policy, McGill University and Génome Québec Innovation Centre, Montreal, QC H3A 1A4, Canada.

43. Heidelberg Academy of Sciences and Humanities, Heidelberg 69120, Germany.

44. CAPHRI Research School, Maastricht University, Maastricht, ER 6229, The Netherlands.

45. Genome Informatics Program, Ontario Institute for Cancer Research, Toronto, ON M5G 0A3, Canada.

46. Barcelona Supercomputing Center (BSC), Barcelona 08034, Spain.

47. Laboratory for Medical Science Mathematics, RIKEN Center for Integrative Medical Sciences, Yokohama, Kanagawa 230-0045, Japan.

48. RIKEN Center for Integrative Medical Sciences, Yokohama, Kanagawa 230-0045, Japan.

49. Dana-Farber Cancer Institute, Boston, MA 02215, USA.

50. University of California Santa Cruz, Santa Cruz, CA 95064, USA.

51. Oregon Health and Science University, Portland, OR 97239, USA.

52. Division of Theoretical Bioinformatics, German Cancer Research Center (DKFZ), Heidelberg 69120, Germany.

53. Heidelberg Center for Personalized Oncology (DKFZ-HIPO), German Cancer Research Center, Heidelberg 69120, Germany.

54. Institute of Pharmacy and Molecular Biotechnology and BioQuant, Heidelberg University, Heidelberg 69120, Germany.

55. University of California San Diego, San Diego, CA 92093, USA.

56. PDXen Biosystems Inc, Seoul 4900, South Korea.

57. Electronics and Telecommunications Research Institute, Daejoen 34129, South Korea.

58. Seven Bridges Genomics, Charlestown, MA 02129, USA.

59. Annai Systems, Inc, Carlsbad, CA 92013, USA.

60. Department of Biomedical Data Science, Stanford University School of Medicine, Stanford, CA 94305, USA.

61. Department of Genetics, Stanford University School of Medicine, Stanford, CA 94305, USA.

62. Departments of Genetics and Biomedical Data Science, Stanford University School of Medicine, Stanford, CA 94305, USA.

63. University of Leuven, Leuven B-3000, Belgium.

64. The Francis Crick Institute, London NW1 1AT, UK.

65. The Hospital for Sick Children, Toronto, ON M5G 0A4, Canada.

66. Heidelberg University, Heidelberg 69120, Germany.

67. New BIH Digital Health Center, Berlin Institute of Health (BIH) and Charité - Universitätsmedizin Berlin, Berlin 10117, Germany.

68. Department of Biochemistry and Molecular Medicine, University of Montreal, Montreal, QC H3C 3J7, Canada.

69. CIBIO/InBIO - Research Center in Biodiversity and Genetic Resources, Universidade do Porto, Vairão 4485-601, Portugal.

70. Department Biochemistry and Molecular Biomedicine, University of Barcelona, Barcelona 08028, Spain.

71. University of Chicago, Chicago, IL 60637, USA.

72. Division of Biomedical Informatics, Department of Medicine, & Moores Cancer Center, UC San Diego School of Medicine, San Diego, CA 92093, USA.

73. Children's Hospital of Philadelphia, Philadelphia, PA 19146, USA.

74. Massachusetts General Hospital Center for Cancer Research, Charlestown, MA 02129, USA.

75. University of Melbourne Centre for Cancer Research, University of Melbourne, Melbourne, VIC 3010, Australia.

76. Syntekabio Inc, Daejon 34025, South Korea.

77. AbbVie, North Chicago, IL 60064, USA.

78. Genomics Program, Ontario Institute for Cancer Research, Toronto, ON M5G 0A3, Canada.

79. German Cancer Consortium (DKTK), Heidelberg 69120, Germany.

80. Heidelberg Center for Personalized Oncology (DKFZ-HIPO), German Cancer Research Center (DKFZ), Heidelberg 69120, Germany.

81. National Center for Tumor Diseases (NCT) Heidelberg, Heidelberg 69120, Germany.

82. Department of Pediatric Immunology, Hematology and Oncology, University Hospital, Heidelberg 69120, Germany.

83. German Cancer Research Center (DKFZ), Heidelberg 69120, Germany.

84. Heidelberg Institute for Stem Cell Technology and Experimental Medicine (HI-STEM), Heidelberg 69120, Germany.

85. Institute of Medical Science, University of Tokyo, Tokyo 108-8639, Japan.

86. The Institute of Medical Science, The University of Tokyo, Tokyo 108-8639, Japan.

87. Seven Bridges, Charlestown, MA 02129, USA.

88. Genome Integration Data Center, Syntekabio, Inc, Daejon, 34025, South Korea.

89. Computational Biology Center, Memorial Sloan Kettering Cancer Center, New York, NY 10065, USA.

90. ETH Zurich, Department of Biology, Zürich 8093, Switzerland.

91. ETH Zurich, Department of Computer Science, Zurich 8092, Switzerland.

92. SIB Swiss Institute of Bioinformatics, Lausanne 1015, Switzerland.

93. University Hospital Zurich, Zurich, 8091, Switzerland.

94. Health Sciences Department of Biomedical Informatics, University of California San Diego, La Jolla, CA 92093, USA.

95. Department of Health Sciences and Technology, Sungkyunkwan University School of Medicine, Seoul 06351, South Korea.

96. Samsung Genome Institute, Seoul 06351, South Korea.

97. Functional and Structural Genomics, German Cancer Research Center (DKFZ), Heidelberg 69120, Germany.

98. Leidos Biomedical Research, Inc, McLean, VA 22102, USA.

99. Sage Bionetworks, Seattle WA 98109, USA.

100. Genome Informatics, Ontario Institute for Cancer Research, Toronto, ON M5G 2C4, Canada.

101. Department of Cell and Systems Biology, University of Toronto, Toronto, ON M5S 3G5, Canada.

102. Department of Radiation Oncology, University of California San Francisco, San Francisco, CA 94518, USA.

103. CSRA Incorporated, Fairfax, VA 22042, USA.

104. Massachusetts General Hospital, Boston, MA 02114, USA.

105. Department of Biology, ETH Zurich, Wolfgang-Pauli-Strasse 27, 8093 Zürich, Switzerland.

106. Department of Computer Science, ETH Zurich, Zurich 8092, Switzerland.

107. Weill Cornell Medical College, New York, NY 10065, USA.

108. Bioinformatics and Omics Data Analytics, German Cancer Research Center (DKFZ), Heidelberg 69120, Germany.

109. Institució Catalana de Recerca i Estudis Avançats (ICREA), Barcelona 08010, Spain.

110. Department of Clinical and Molecular Medicine, Faculty of Medicine and Health Sciences, Norwegian University of Science and Technology, Trondheim 7030, Norway.

111. Finsen Laboratory and Biotech Research & Innovation Centre (BRIC), University of Copenhagen, Copenhagen 2200, Denmark.

112. Department of Urology, Charité Universitätsmedizin Berlin, Berlin 10117, Germany.

113. Department of Biological Oceanography, Leibniz Institute of Baltic Sea Research, Seestraße 15, Rostock 18119, Germany.

114. Ontario Institute for Cancer Research, Toronto, ON M5G 0A3, Canada.

115. Department of Physiology and Biophysics, Weill Cornell Medicine, New York, NY 10065, USA.

116. Institute for Computational Biomedicine, Weill Cornell Medicine, New York, NY 10021, USA.

117. Division of Applied Bioinformatics, German Cancer Research Center (DKFZ), Heidelberg 69120, Germany.

118. Department of Computer Science, Yale University, New Haven, CT 06520, USA.

119. Department of Molecular Biophysics and Biochemistry, Yale University, New Haven, CT 06520, USA.

120. Program in Computational Biology and Bioinformatics, Yale University, New Haven, CT 06520, USA.

121. Department of Internal Medicine, Stanford University, Stanford, CA 94305, USA.

122. Department of Molecular Medicine (MOMA), Aarhus University Hospital, Aarhus N 8200, Denmark.

123. Clinical Bioinformatics, Swiss Institute of Bioinformatics, Geneva 1202, Switzerland.

124. Institute for Pathology and Molecular Pathology, University Hospital Zurich, Zurich 8091, Switzerland.

125. Institute of Molecular Life Sciences, University of Zurich, Zurich 8057, Switzerland.

126. MIT Computer Science and Artificial Intelligence Laboratory, Massachusetts Institute of Technology, Cambridge, MA 02139, USA.

127. Englander Institute for Precision Medicine, Weill Cornell Medicine, New York, NY 10065, USA.

128. Meyer Cancer Center, Weill Cornell Medicine, New York, NY 10065, USA.

129. Bioinformatics Research Centre (BiRC), Aarhus University, Aarhus 8000, Denmark.

130. Department of Medical Biophysics, University of Toronto, Toronto, ON M5S 1A8, Canada.

131. Institute of Molecular Life Sciences and Swiss Institute of Bioinformatics, University of Zurich, Zurich 8057, Switzerland.

132. CNAG-CRG, Centre for Genomic Regulation (CRG), Barcelona Institute of Science and Technology (BIST), Barcelona 08028, Spain.

133. Universitat Pompeu Fabra (UPF), Barcelona 08003, Spain.

134. Office of Cancer Genomics, National Cancer Institute, US National Institutes of Health, Bethesda, MD 20892, USA.

135. Alvin J. Siteman Cancer Center, Washington University School of Medicine, St Louis, MO 63110, USA.

136. The McDonnell Genome Institute at Washington University, St Louis, MO 63108, USA.

137. Computer Network Information Center,Chinese Academy of Sciences, Beijing 100190, China.

138. Center for Digital Health, Berlin Institute of Health and Charitè - Universitätsmedizin Berlin, Berlin 10117, Germany.

139. Department of Pharmacology, University of Toronto, Toronto, ON M5S 1A8, Canada.

140. University of California Los Angeles, Los Angeles, CA 90095, USA.

141. University of Texas MD Anderson Cancer Center, Houston, TX 77030, USA.

142. Department of Genetics and Informatics Institute, University of Alabama at Birmingham, Birmingham, AL 35294, USA.

143. Department of Genetics, Department of Medicine, Washington University in St Louis, St Louis, MO 63110, USA.

144. Centre for Genomic Regulation (CRG), The Barcelona Institute of Science and Technology, Barcelona 08003, Spain.

145. Department of Bioinformatics and Computational Biology, The University of Texas MD Anderson Cancer Center, Houston, TX 77030, USA.

146. Beijing Genomics Institute, Shenzhen 518083, China.

147. Department of Urologic Sciences, University of British Columbia, Vancouver, BC V5Z 1M9, Canada.

148. Vancouver Prostate Centre, Vancouver, BC V6H 3Z6, Canada.

149. Division of Life Science and Applied Genomics Center, Hong Kong University of Science and Technology, Clear Water Bay, Hong Kong, China.

150. Geneplus-Shenzhen, Shenzhen 518122, China.

151. School of Computer Science and Technology, Xi'an Jiaotong University, Xi'an 710048, China.

152. Biobyte solutions GmbH, Heidelberg 69126, Germany.

153. Division of Oncology, Washington University School of Medicine, St Louis, MO 63110, USA.

154. Institute of Medical Genetics and Applied Genomics, University of Tübingen, Tübingen 72074, Germany.

155. Indiana University, Bloomington, IN 47405, USA.

156. Simon Fraser University, Burnaby, BC V5A 1S6, Canada.

157. Department of Computer Science, University of Toronto, Toronto, ON M5S 1A8, Canada.

158. School of Electronic and Information Engineering, Xi'an Jiaotong University, Xi'an 710048, China.

159. Department of Genetics, Washington University School of Medicine, St Louis, MO 63110, USA.

160. Department of Mathematics, Washington University in St Louis, St Louis, MO 63130, USA.

161. Department of Molecular and Human Genetics, Baylor College of Medicine, Houston, TX 77030, USA.

162. Human Genome Sequencing Center, Baylor College of Medicine, Houston, TX 77030, USA.

163. The First Affiliated Hospital, Xi'an Jiaotong University, Xi'an 710049, China.

164. Department of Epidemiology and Biostatistics, Memorial Sloan Kettering Cancer Center, New York, NY 10065, USA.

165. The McDonnell Genome Institute at Washington University, Department of Genetics, Department of Medicine, Siteman Cancer Center, Washington University in St Louis, St Louis, MO 63108, USA.

166. Department of Genomic Medicine, The University of Texas MD Anderson Cancer Center, Houston, TX 77030, USA.

167. The Jackson Laboratory for Genomic Medicine, Farmington, CT 06032, USA.

168. Quantitative & Computational Biosciences Graduate Program, Baylor College of Medicine, Houston, TX 77030, USA.

169. Dana-Farber/Boston Children's Cancer and Blood Disorders Center, Boston, MA 02215, USA.

170. Department of Pediatrics, Harvard Medical School, Boston, MA 02115, USA.

171. Department of Medical Oncology, Dana-Farber Cancer Institute, Boston, MA 02115, USA.

172. Department of Mathematics, Aarhus University, Aarhus 8000, Denmark.

173. Technical University of Denmark, Lyngby 2800, Denmark.

174. University of Copenhagen, Copenhagen 2200, Denmark.

175. Foundation Medicine, Inc, Cambridge, MA 02141, USA.

176. Department for BioMedical Research, University of Bern, Bern 3008, Switzerland.

177. Department of Medical Oncology, Inselspital, University Hospital and University of Bern, Bern 3010, Switzerland.

178. Graduate School for Cellular and Biomedical Sciences, University of Bern, Bern 3012, Switzerland.

179. Department of Genitourinary Medical Oncology - Research, Division of Cancer Medicine, The University of Texas MD Anderson Cancer Center, Houston, TX 77030, USA.

180. Faculty of Biosciences, Heidelberg University, Heidelberg 69120, Germany.

181. Korea Advanced Institute of Science and Technology, Daejeon 34141, South Korea.

182. Institute for Research in Biomedicine (IRB Barcelona), The Barcelona Institute of Science and Technology, Barcelona 8003, Spain.

183. Research Program on Biomedical Informatics, Universitat Pompeu Fabra, Barcelona 08002, Spain.

184. Science for Life Laboratory, Department of Cell and Molecular Biology, Uppsala University, Uppsala SE-75124, Sweden.

185. Queensland Centre for Medical Genomics, Institute for Molecular Bioscience, The University of Queensland, St Lucia, QLD 4072, Australia.

186. University of Milano Bicocca, Monza 20052, Italy.

187. Peter MacCallum Cancer Centre, Melbourne, VIC 3000, Australia.

188. Sir Peter MacCallum Department of Oncology, The University of Melbourne, Melbourne, VIC 3052, Australia.

189. Center for Precision Health, School of Biomedical Informatics, University of Texas Health Science Center, Houston, TX 77030, USA.

190. The Donnelly Centre, University of Toronto, Toronto, ON M5S 3E1, Canada.

191. Health Data Science Unit, University Clinics, Heidelberg 69120, Germany.

192. Department for Biomedical Research, University of Bern, Bern 3008, Switzerland.

193. Research Core Center, National Cancer Centre Korea, Goyang-si 410-769, South Korea.

194. Institute of Computer Science, Polish Academy of Sciences, Warsawa 01-248, Poland.

195. ETH Zurich, Department of Biology, Wolfgang-Pauli-Strasse 27, 8093 Zürich, Switzerland.

196. Harvard University, Cambridge, MA 02138, USA.

197. Memorial Sloan Kettering Cancer Center, New York, NY 10065, USA.

198. Department of Molecular Biophysics and Biochemistry, New Haven, CT 06520, USA.

199. Program in Computational Biology and Bioinformatics, New Haven, CT 06520, USA.

200. Yale University, New Haven, CT 06520, USA.

201. Department of Information Technology, Ghent University, Ghent B-9000, Belgium.

202. Department of Plant Biotechnology and Bioinformatics, Ghent University, Ghent B-9000, Belgium.

203. Yale School of Medicine, Yale University, New Haven, CT 06520, USA.

204. Institute for Research in Biomedicine (IRB Barcelona), Barcelona 08028, Spain.

205. Division of Hematology-Oncology, Samsung Medical Center, Sungkyunkwan University School of Medicine, Seoul, 06351, South Korea.

206. Samsung Advanced Institute for Health Sciences and Technology, Sungkyunkwan University School of Medicine, Seoul 06351, South Korea.

207. Cheonan Industry-Academic Collaboration Foundation, Sangmyung University, Cheonan 31066, South Korea.

208. Spanish National Cancer Research Centre, Madrid 28029, Spain.

209. Department of Computer Science, Princeton University, Princeton, NJ 08540, USA.

210. Bern Center for Precision Medicine, University Hospital of Bern, University of Bern, Bern 3008, Switzerland.

211. Englander Institute for Precision Medicine, Weill Cornell Medicine and NewYork Presbyterian Hospital, New York, NY 10021, USA.

212. Pathology and Laboratory, Weill Cornell Medical College, New York, NY 10021, USA.

213. Vall d'Hebron Institute of Oncology: VHIO, Barcelona 08035, Spain.

214. cBio Center, Dana-Farber Cancer Institute, Harvard Medical School, Boston, MA 02115, USA.

215. Department of Cell Biology, Harvard Medical School, Boston, MA 02115, USA.

216. Department of Cancer Biology, Dana-Farber Cancer Institute, Boston, MA 02215, USA.

217. Peter MacCallum Cancer Centre and University of Melbourne, Melbourne, VIC 3000, Australia.

218. cBio Center, Dana-Farber Cancer Institute, Boston, MA 02215, USA.

219. CREST, Japan Science and Technology Agency, Tokyo 113-0033, Japan.

220. Department of Medical Science Mathematics, Medical Research Institute, Tokyo Medical and Dental University, Bunkyo-ku, Tokyo 113-8510, Japan.

221. Laboratory for Medical Science Mathematics, Department of Biological Sciences, Graduate School of Science, The University of Tokyo, Bunkyo-ku, Tokyo 113-0033, Japan.

222. Science for Life Laboratory, Department of Oncology-Pathology, Karolinska Institutet, Stockholm 17121, Sweden.

223. Department of Gene Technology, Tallinn University of Technology, Tallinn 12616, Estonia.

224. Genetics & Genome Biology Program, SickKids Research Institute, The Hospital for Sick Children, Toronto, ON M5G 1X8, Canada.

225. Department of Information Technology, Ghent University, Interuniversitair Micro-Electronica Centrum (IMEC), Ghent B-9000, Belgium.

226. Science for Life Laboratory, Department of Immunology, Genetics and Pathology, Uppsala University, Uppsala SE-75108, Sweden.

227. Oregon Health & Sciences University, Portland, OR 97239, USA.

228. Department of Medicine and Therapeutics, The Chinese University of Hong Kong, Shatin, NT, Hong Kong, China SAR.

229. Second Military Medical University, Shanghai 200433, China.

230. The University of Texas Health Science Center at Houston, Houston, TX 77030, USA.

231. Department of Biomedical Informatics, College of Medicine, The Ohio State University, Columbus, OH 43210, USA.

232. The Ohio State University Comprehensive Cancer Center (OSUCCC – James), Columbus, OH 43210, USA.

233. School of Biomedical Informatics, The University of Texas Health Science Center at Houston, Houston, TX 77030, USA.

234. Department of Biochemistry and Molecular Genetics, Feinberg School of Medicine, Northwestern University, Chicago, IL 60637, USA.

235. University of Glasgow, CRUK Beatson Institute for Cancer Research, Bearsden, Glasgow G61 1BD, UK.

236. Centre for Molecular Science Informatics, Department of Chemistry, University of Cambridge, Cambridge CB2 1EW, UK.

237. Department of Biomedical Informatics, Harvard Medical School, Boston, MA 02115, USA.

238. Ludwig Center, Harvard Medical School, Boston, MA 02115, USA.

239. UC Santa Cruz Genomics Institute, University of California Santa Cruz, Santa Cruz, CA 95064, USA.

240. Massachusetts General Hospital, Physics Division, Optimization and Systems Biology Lab, Boston, MA 02114, USA.

241. Department of Medicine, Baylor College of Medicine, Houston, TX 77030, USA.

242. Computational and Systems Biology, Genome Institute of Singapore, Singapore 138672, Singapore.

243. School of Computing, National University of Singapore, Singapore 117417, Singapore.

244. The Azrieli Faculty of Medicine, Bar-Ilan University, Safed 13195, Israel.

245. National Cancer Centre Singapore, Singapore 169610, Singapore.

246. Peking University, Beijing 100871, China.

247. Department of Genetics, University of North Carolina at Chapel Hill, Chapel Hill, NC 27599, USA.

248. Lineberger Comprehensive Cancer Center, University of North Carolina at Chapel Hill, Chapel Hill, NC 27599, USA.

249. China National GeneBank-Shenzhen, Shenzhen 518083, China.

250. Berlin Institute for Medical Systems Biology, Max Delbrück Center for Molecular Medicine, Berlin 13125, Germany.

251. University College London, London WC1E 6BT, UK.

252. School of Life Sciences, Peking University, Beijing 100180, China.

253. Department of Pathology, The University of Melbourne, Melbourne, VIC 3052, Australia.

254. Genome Institute of Singapore, Singapore 138672, Singapore.

255. Department of Epidemiology, University of Alabama at Birmingham, Birmingham, AL 35294, USA.

256. HudsonAlpha Institute for Biotechnology, Huntsville, AL 35806, USA.

257. O'Neal Comprehensive Cancer Center, University of Alabama at Birmingham, Birmingham, AL 35294, USA.

258. Department of Biosciences and Nutrition, Karolinska Institutet, Stockholm 14183, Sweden.

259. Ludwig Center at Harvard, Boston, MA 02115, USA.

260. German Cancer Consortium (DKTK), Partner site Berlin.

261. Human Genetics, University of Kiel, Kiel 24118, Germany.

262. Institute of Human Genetics, Ulm University and Ulm University Medical Center, Ulm 89081, Germany.

263. Computational & Systems Biology Program, Memorial Sloan Kettering Cancer Center, New York, NY 10065, USA.

264. Korea University, Seoul 02481, South Korea.

265. Division of Computational Genomics and Systems Genetics, German Cancer Research Center (DKFZ), Heidelberg 69120, Germany.

266. Cancer Science Institute of Singapore, National University of Singapore, Singapore 169609, Singapore.

267. Programme in Cancer & Stem Cell Biology, Duke-NUS Medical School, Singapore 169857, Singapore.

268. SingHealth, Duke-NUS Institute of Precision Medicine, National Heart Centre Singapore, Singapore 169609, Singapore.

269. Institute of Molecular and Cell Biology, Singapore 169609, Singapore.

270. Laboratory of Cancer Epigenome, Division of Medical Science, National Cancer Centre Singapore, Singapore 169610, Singapore.

271. BIOPIC, ICG and College of Life Sciences, Peking University, Beijing 100871, China.

272. Genome Science Division, Research Center for Advanced Science and Technology, The University of Tokyo, Tokyo 153-8904, Japan.

273. Center for Bioinformatics and Functional Genomics, Cedars-Sinai Medical Center, Los Angeles, CA 90048, USA.

274. Department of Biomedical Sciences, Cedars-Sinai Medical Center, Los Angeles, CA 90048, USA.

275. The Hebrew University Faculty of Medicine, Jerusalem 91120, Israel.

276. Bioinformatics Group, Department of Computer Science, University of Leipzig, Leipzig 04109, Germany.

277. Interdisciplinary Center for Bioinformatics, University of Leipzig, Leipzig 04109, Germany.

278. German Cancer Consortium (DKTK), German Cancer Research Center (DKFZ), Heidelberg 69120, Germany.

279. Computational Biology, Leibniz Institute on Aging - Fritz Lipmann Institute (FLI), Jena 07745, Germany.

280. Transcriptome Bioinformatics, LIFE Research Center for Civilization Diseases, University of Leipzig, Leipzig 04109, Germany.

281. Center for Epigenetics, Van Andel Research Institute, Grand Rapids, MI 49503, USA.

282. Institut d'Investigacions Biomèdiques August Pi i Sunyer (IDIBAPS), Barcelona 08036, Spain.

283. Research Center for Advanced Science and Technology, The University of Tokyo, Minato-ku, Tokyo 108-8639, Japan.

284. Cancer Epigenomics, German Cancer Research Center (DKFZ), Heidelberg 69120, Germany.

285. Van Andel Research Institute, Grand Rapids, MI 49503, USA.

286. Department of Zoology, Genetics and Physical Anthropology, Universidade de Santiago de Compostela, Santiago de Compostela 15706, Spain.

287. Centre for Research in Molecular Medicine and Chronic Diseases (CIMUS), Universidade de Santiago de Compostela, Santiago de Compostela 15706, Spain.

288. The Biomedical Research Centre (CINBIO), Universidade de Vigo, Vigo 36310, Spain.

289. Transmissible Cancer Group, Department of Veterinary Medicine, University of Cambridge, Cambridge CB3 0ES, UK.

290. Sir Peter MacCallum Department of Oncology, University of Melbourne, Melbourne, VIC 3052, Australia.

291. Johns Hopkins School of Medicine, Baltimore, MD 21205, USA.

292. University of Ottawa Faculty of Medicine, Department of Biochemistry, Microbiology and Immunology, Ottawa, ON K1H 8M5, Canada.

293. Cancer Research UK Cambridge Institute, University of Cambridge, Cambridge CB2 0RE, UK.

294. University of Cambridge, Cambridge CB2 1TN, UK.

295. Quantitative Genomics Laboratories (qGenomics), Barcelona 08950, Spain.

296. Genome Integrity and Structural Biology Laboratory, National Institute of Environmental Health Sciences (NIEHS), Durham, NC 27709, USA.

297. Brandeis University, Waltham, MA 02254, USA.

298. New York Genome Center, New York, NY 10013, USA.

299. Weill Cornell Medicine, New York, NY 10065, USA.

300. Hopp Children's Cancer Center (KiTZ), Heidelberg 69120, Germany.

301. Pediatric Glioma Research Group, German Cancer Research Center (DKFZ), Heidelberg 69120, Germany.

302. Skolkovo Institute of Science and Technology, Moscow 121205, Russia.

303. A.A.Kharkevich Institute of Information Transmission Problems, Moscow 127051, Russia.

304. Dmitry Rogachev National Research Center of Pediatric Hematology, Oncology and Immunology, Moscow, 117997, Russia.

305. Integrative Bioinformatics Support Group, National Institute of Environmental Health Sciences (NIEHS), Durham, NC 27709, USA.

306. Center For Medical Innovation, Seoul National University Hospital, Seoul 03080, South Korea.

307. Department of Internal Medicine, Seoul National University Hospital, Seoul 03080, South Korea.

308. Division of Genetics and Genomics, Boston Children's Hospital and Harvard Medical School, Boston, MA 02115, USA.

309. School of Medicine/School of Mathematics and Statistics, University of St Andrews, St Andrews, Fife KY16 9SS, UK.

310. Department of Genetics and Computational Biology, QIMR Berghofer Medical Research Institute, Brisbane 4006, Australia.

311. Institute for Molecular Bioscience, University of Queensland, St Lucia, Brisbane, QLD 4072, Australia.

312. School of Molecular Biosciences and Center for Reproductive Biology, Washington State University, Pullman, WA 99164, USA.

313. Cancer Research Institute, Beth Israel Deaconess Medical Center, Boston, MA 02215, USA.

314. Ben May Department for Cancer Research, Department of Human Genetics, The University of Chicago, Chicago, IL 60637, USA.

315. Tri-institutional PhD program of computational biology and medicine, Weill Cornell Medicine, New York, NY 10065, USA.

316. Department of Cellular and Molecular Medicine and Department of Bioengineering and Moores Cancer Center, University of California San Diego, La Jolla, California 92093, USA.

317. Department of Cellular and Molecular Medicine and Department of Bioengineering and Moores Cancer Center, University of California, San Diego, La Jolla, California 92093, USA.

318. Centre for Computational Biology, Duke-NUS Medical School, Singapore 169857, Singapore.

319. Department of Computer Science, University of Helsinki, Helsinki 00014, Finland.

320. Institute of Biotechnology, University of Helsinki, Helsinki 00014, Finland.

321. Organismal and Evolutionary Biology Research Programme, University of Helsinki, Helsinki 00014, Finland.

322. Programme in Cancer & Stem Cell Biology, Centre for Computational Biology, Duke-NUS Medical School, Singapore 169857, Singapore.

323. Academic Department of Medical Genetics, University of Cambridge, Addenbrooke's Hospital, Cambridge CB2 0QQ, UK.

324. MRC Cancer Unit, University of Cambridge, Cambridge CB2 0XZ, UK.

325. The University of Cambridge School of Clinical Medicine, Cambridge CB2 0SP, UK.

326. National Centre for Biological Sciences, Tata Institute of Fundamental Research, Bangalore 560065, India.

327. Department of Applied Mathematics and Theoretical Physics, Centre for Mathematical Sciences, University of Cambridge, Cambridge CB3 0WA, UK.

328. Department of Statistics, Columbia University, New York, NY 10027, USA.

329. Duke-NUS Medical School, Singapore 169857, Singapore.

330. Faculty of Medicine and Health Technology, Tampere University and Tays Cancer Center, Tampere University Hospital, Tampere Fl-33014, Finland.

331. Institute for Computational Health Sciences and Department of Pediatrics, University of California, San Francisco, CA USA.

332. Division of Cancer Epidemiology and Genetics, National Cancer Institute, National Institutes of Health, Bethesda, MD 20892, USA.

333. Department of Biostatistics, Bloomberg School of Public Health, Johns Hopkins University, Baltimore, MD 21287, USA.

334. Department of Oncology, School of Medicine, Johns Hopkins University, Baltimore, MD 21230, USA.

335. Integrated Graduate Program in Physical and Engineering Biology, Yale University, New Haven, CT 06520, USA.

336. Department of Computational Biology, University of Lausanne, Lausanne 1015, Switzerland.

337. Department of Genetic Medicine and Development, University of Geneva Medical School, Geneva CH1211, Switzerland.

338. Swiss Institute of Bioinformatics, University of Geneva, Geneva CH1211, Switzerland.

339. Institute of Medical Genetics and Applied Genomics, University of Tübingen, Tübingen 72076, Germany.

340. Independent Consultant, Wellesley 02481, USA.

341. Centre for Cancer Genetic Epidemiology, Department of Oncology, University of Cambridge, Cambridge CB1 8RN, UK.

342. Centre for Cancer Genetic Epidemiology, Department of Public Health and Primary Care, University of Cambridge, Cambridge CB1 8RN, UK.

343. CIBER Epidemiología y Salud Pública (CIBERESP), Madrid 28029, Spain.

344. Research Group on Statistics, Econometrics and Health (GRECS), UdG, Barcelona 8041, Spain.

345. Oxford Nanopore Technologies, New York, NY 10013, USA.

346. Department of Medical Genetics, College of Medicine, Hallym University, Chunchoen 24252, South Korea.

347. Institute of Evolutionary Biology (UPF-CSIC), Department of Experimental and Health Sciences, Universitat Pompeu Fabra, Barcelona 08003, Spain.

348. Icahn School of Medicine at Mount Sinai, New York, NY 10029, USA.

349. Laboratory of Translational Genomics, Division of Cancer Epidemiology and Genetics, National Cancer Institute, National Institutes of Health, Bethesda, MD 20892, USA.

350. Institut Català de Paleontologia Miquel Crusafont, Universitat Autònoma de Barcelona, Barcelona 08193, Spain.

351. Applications Department, Oxford Nanopore Technologies, Oxford OX4 4DQ, UK.

352. Institut de Recerca Sant Joan de Déu; Institut de Biomedicina de la Universitat de Barcelona (IBUB) & Department of Genetics, Microbiology & Statistics, Faculty of Biology, University of Barcelona, Barcelona 08028, Spain.

353. Institute for Research in Biomedicine (IRB Barcelona), The Barcelona Institute of Science and Technology, Barcelona 08028, Spain.

354. Department of Ophthalmology and Ocular Genomics Institute, Massachusetts Eye and Ear, Harvard Medical School, Boston, MA 02114, USA.

355. Department of Medical and Clinical Genetics, Genome-Scale Biology Research Program, University of Helsinki, Helsinki 00100, Finland.

356. Big Data Institute, Li Ka Shing Centre, University of Oxford, Oxford OX3 7LF, UK.

357. Oxford NIHR Biomedical Research Centre, University of Oxford, Oxford OX4 2PG, UK.

358. School of Electronic Information and Communications, Huazhong University of Science and Technology, Wuhan, Hubei 430074, China.

359. Bioinformatics Unit, Spanish National Cancer Research Centre (CNIO), Madrid 28029, Spain.

360. Vector Institute, Toronto, ON M5G 0A3, Canada.

361. South Western Sydney Clinical School, Faculty of Medicine, University of NSW, Liverpool, NSW 2170, Australia.

362. The Kinghorn Cancer Centre, Cancer Division, Garvan Institute of Medical Research, University of NSW, Sydney, NSW 2010, Australia.

363. West of Scotland Pancreatic Unit, Glasgow Royal Infirmary, Glasgow G31 2ER, UK.

364. Wolfson Wohl Cancer Research Centre, Institute of Cancer Sciences, University of Glasgow, Bearsden, Glasgow G61 1QH, UK.

365. University of Melbourne Centre for Cancer Research, The University of Melbourne, Melbourne, VIC 3052, Australia.

366. MRC Human Genetics Unit, MRC IGMM, University of Edinburgh, Edinburgh EH4 2XU, UK.

367. Bioinformatics Group, Division of Molecular Biology, Department of Biology, Faculty of Science, University of Zagreb, Zagreb 10000, Croatia.

368. Department of Bioinformatics, Research Institute, National Cancer Center Japan, Tokyo 104-0045, Japan.

369. Departments of Pathology, Genomic Medicine, and Translational Molecular Pathology, The University of Texas MD Anderson Cancer Center, Houston, TX 77030, USA.

370. Oregon Health & Science University, Portland, OR 97239, USA.

371. University of Glasgow, Glasgow G61 1BD, UK.

372. MRC-University of Glasgow Centre for Virus Research, Glasgow G61 1QH, UK.

373. Wolfson Wohl Cancer Research Centre, Institute of Cancer Sciences, University of Glasgow, Bearsden G61 1QH, United Kingdom.

374. School of Computing Science, University of Glasgow, Glasgow G12 8RZ, UK.

375. Molecular and Medical Genetics, Oregon Health and Science University, Portland, OR 97201, USA.

376. Department of Surgery, University of Melbourne, Parkville VIC 3010, Australia.

377. The Murdoch Children's Research Institute, Royal Children's Hospital, Parkville, VIC 3052, Australia.

378. Walter + Eliza Hall Institute, Parkville, VIC 3052, Australia.

379. University of Cologne, Cologne 50931, Germany.

380. The Edward S. Rogers Sr. Department of Electrical and Computer Engineering, University of Toronto, Toronto, ON M5S 3G4, Canada.

381. University of Ljubljana, Ljubljana 1000, Slovenia.

382. Research Institute, NorthShore University HealthSystem, Evanston, IL 60201, USA.

383. Department of Public Health Sciences, The University of Chicago, Chicago IL 60637.

384. Department of Statistics, University of California Santa Cruz, Santa Cruz, CA 95064, USA.

385. Cambridge University Hospitals NHS Foundation Trust, Cambridge CB2 0QQ, UK.

386. University of Toronto, Toronto, ON M5G 2M9, Canada.

387. Department of Computer Science, Carleton College, Northfield, MN 55057, USA.

388. Molecular and Medical Genetics, Oregon Health & Science University, Portland, OR 97239, USA.

389. Center for Psychiatric Genetics, NorthShore University HealthSystem, Evanston, IL 60201, USA.

390. Molecular and Medical Genetics, Knight Cancer Institute, Oregon Health & Science University, Portland, OR 97219, USA.

391. Argmix Consulting, North Vancouver BC V7M 2J5, Canada.

392. University of Toronto, Department of Computer Science, Toronto, ON M5S 2E4, Canada.

393. Department of Biostatistics, The University of Texas MD Anderson Cancer Center, Houston, TX 77030, USA.

394. Department of Biostatistics, University of North Carolina at Chapel Hill, Chapel Hill, NC 27599, USA.

395. The University of Texas MD Anderson Cancer Center, Houston, TX 77030, USA.

396. Howard Hughes Medical Institute, University of California Santa Cruz, Santa Cruz, CA 95065, USA.

397. Cancer Unit, MRC University of Cambridge, Cambridge CB2 0XZ, UK.

398. Department of Bioinformatics and Computational Biology and Department of Systems Biology, The University of Texas MD Anderson Cancer Center, Houston, TX 77030, USA.

399. Department of Bioinformatics and Computational Biology, Department of Systems Biology, The University of Texas MD Anderson Cancer Center, Houston, TX 77030, USA.

400. Department of Health Sciences, Faculty of Medical Sciences, Kyushu University, Fukuoka 812-8582, Japan.

401. Baylor College of Medicine, Houston, TX 77030, USA.

402. Department of Applied Mathematics and Statistics, Johns Hopkins University, Baltimore, MD 21218, USA.

403. Heinrich Pette Institute, Leibniz Institute for Experimental Virology, Hamburg 20251, Germany.

404. University Medical Center Hamburg-Eppendorf, Bioinformatics Core, Hamburg 20246, Germany.

405. Earlham Institute, Norwich NR4 7UZ, UK.

406. Norwich Medical School, University of East Anglia, Norwich NR4 7TJ, UK.

407. The Institute of Cancer Research, London SW7 3RP, UK.

408. University of East Anglia, Norwich NR4 7TJ, UK.

409. German Center for Infection Research (DZIF), Partner Site Hamburg-Borstel-Lübeck-Riems, Hamburg, Germany.

410. Division of Molecular Genetics, German Cancer Research Center (DKFZ), Heidelberg 69120, Germany.

411. Peter MacCallum Cancer Centre, Melbourne, Victoria 3000, Australia.

412. Sir Peter MacCallum Department of Oncology, The University of Melbourne, Melbourne VIC 3000, Australia.

413. QIMR Berghofer Medical Research Institute, Brisbane, QLD 4006, Australia.

414. Victorian Institute of Forensic Medicine, Southbank, Victoria 3006, Australia.

415. University of Pennsylvania, Philadelphia, PA 19104, USA.

416. Peter MacCallum Cancer Centre, Melbourne VIC 3000, Australia.

417. Centre for Cancer Research, The Westmead Institute for Medical Research, Sydney 2145, Australia.

418. Department of Gynaecological Oncology, Westmead Hospital, Sydney 2145, Australia.

419. Genetics and Molecular Pathology, SA Pathology, Adelaide, SA 5000, Australia.

420. Centre for Cancer Research, The Westmead Institute for Medical Research, The University of Sydney, Sydney, NSW 2145, Australia.

421. Department of Gynaecological Oncology, Westmead Hospital, Sydney, NSW 2006, Australia.

422. Garvan Institute of Medical Research, Darlinghurst, NSW 2010 Australia.

423. Centre for Cancer Research, The Westmead Institute for Medical Research, and Department of Gynaecological Oncology, Westmead Hospital, Sydney, NSW 2145, Australia.

424. The University of Sydney, Sydney, NSW 2006, Australia.

425. The Westmead Institute for Medical Research. The University of Sydney. The Department of Gynaecological Oncology, Westmead Hospital, Westmead, NSW 2145, Australia.

426. Department of Surgery, Pancreas Institute, University and Hospital Trust of Verona, Verona 37134, Italy.

427. Department of Surgery, Princess Alexandra Hospital, Woolloongabba QLD 4102, Australia.

428. Surgical Oncology Group, Diamantina Institute, The University of Queensland, Woolloongabba, Brisbane, QLD 4102, Australia.

429. Department of Diagnostics and Public Health, University and Hospital Trust of Verona, Verona 37134, Italy.

430. ARC-Net Centre for Applied Research on Cancer, University and Hospital Trust of Verona, Verona 37134, Italy.

431. Department of Anatomical Pathology, St Vincent's Hospital, Sydney NSW 2010, Australia.

432. School of Biological Sciences, The University of Auckland, Auckland 1010, New Zealand.

433. Department of Pathology and Diagnostics, University and Hospital Trust of Verona, Verona 37134, Italy.

434. Department of Medicine, Section of Endocrinology, University and Hospital Trust of Verona, Verona 37134, Italy.

435. Department of Pathology, Queen Elizabeth University Hospital, Glasgow G51 4TF, UK.

436. Wolfson Wohl Cancer Research Centre, Bearsden, Glasgow G61 1QH, UK.

437. University of Sydney, Sydney, NSW 2006, Australia.

438. Department of Medical Oncology, Beatson West of Scotland Cancer Centre, Glasgow G12 0YN, UK.

439. Academic Unit of Surgery, School of Medicine, College of Medical, Veterinary and Life Sciences, University of Glasgow, Glasgow Royal Infirmary, Glasgow G4 OSF, UK.

440. Tissue Pathology and Diagnostic Oncology, Royal Prince Alfred Hospital, Camperdown, NSW 2050, Australia.

441. Discipline of Surgery, Western Sydney University, Penrith NSW 2751, Australia.

442. Institute of Cancer Sciences, College of Medical Veterinary and Life Sciences, University of Glasgow, Glasgow G12 8QQ, UK.

443. School of Environmental & Life Sciences, University of Newcastle, Ourimbah, NSW 2258, Australia.

444. School of Surgery M507, University of Western Australia, Nedlands 6009, Australia.

445. Applied Tumor Genomics Research Program, Research Programs Unit, University of Helsinki, Helsinki 00290, Finland.

446. Olivia Newton-John Cancer Research Institute, La Trobe University, Heidelberg, Victoria 3084, Australia.

447. Melanoma Institute Australia, The University of Sydney, Wollstonecraft NSW 2065, Australia.

448. Children's Hospital at Westmead, The University of Sydney, Westmead, NSW 2145, Australia.

449. Melanoma Institute Australia, The University of Sydney, Sydney 2065, Australia.

450. Australian Institute of Tropical Health and Medicine, James Cook University, Douglas QLD 4814, Australia.

451. Bioplatforms Australia, North Ryde, NSW 2109, Australia.

452. Melanoma Institute Australia, Macquarie University, Wollstonecraft NSW, 2109, Australia.

453. Children's Medical Research Institute, Westmead, NSW 2145 Australia.

454. Melanoma Institute Australia, The University of Sydney, Wollstonecraft 2065, NSW, Australia.

455. Westmead Institute for Medical Research, University of Sydney, Westmead, NSW 2145 Australia.

456. Melanoma Institute Australia, The University of Sydney, Wollstonecraft, NSW 2065, Australia.

457. Centre for Cancer Research, The Westmead Millennium Institute for Medical Research, University of Sydney, Westmead Hospital, Westmead NSW 2145, Australia.

458. Centre for Cancer Research, Westmead Institute for Medical Research, Westmead, NSW 2145, Australia.

459. Discipline of Pathology, Sydney Medical School, The University of Sydney, Sydney 2065, Australia.

460. Royal Prince Alfred Hospital, Sydney, NSW 2050, Australia.

461. Bioplatforms Australia, North Ryde, NSW 2109 Australia.

462. School of Mathematics and Statistics, The University of Sydney, Sydney, NSW 2006 Australia.

463. Diagnostic Development, Ontario Institute for Cancer Research, Toronto, ON M5G 0A3, Canada.

464. Ontario Tumour Bank, Ontario Institute for Cancer Research, Toronto, ON M5G 0A3, Canada.

465. PanCuRx Translational Research Initiative, Ontario Institute for Cancer Research, Toronto, ON M5G 0A3, Canada.

466. BioSpecimen Sciences Program, University Health Network, Toronto, ON M5G 2C4, Canada, Toronto, ON M5G 2C4, Canada.

467. Hepatobiliary/Pancreatic Surgical Oncology Program, University Health Network, Toronto, ON M5G 2C4, Canada.

468. Hepatobiliary/pancreatic Surgical Oncology Program, University Health Network, Toronto, ON M5G 2C4, Canada.

469. Lunenfeld-Tanenbaum Research Institute, Mount Sinai Hospital, Toronto, ON M5G 1X5, Canada.

470. Genomics, Ontario Institute for Cancer Research, Toronto, ON M5G 0A3, Canada.

471. Division of Medical Oncology, Princess Margaret Cancer Centre, Toronto, ON M5G 2M9, Canada.

472. Lunenfeld-Tanenbaum Research Institute, Toronto, ON M5G 1X5, Canada.

473. University of Nebraska Medical Centre, Omaha, NE 68198, USA.

474. BioSpecimen Sciences Program, University Health Network, Toronto, ON M5G 2C4, Canada.

475. Transformative Pathology, Ontario Institute for Cancer Research, Toronto, ON M5G 0A3, Canada.

476. Department of Biochemistry and Molecular Medicine, University California at Davis, Sacramento, CA 95817 USA.

477. University Health Network, Princess Margaret Cancer Centre, Toronto, ON M5G 1L7, Canada.

478. Department of Health Sciences Research, Mayo Clinic, Rochester, MN 55905, USA.

479. Department of Pathology, Human Oncology and Pathogenesis Program, Memorial Sloan Kettering Cancer Center, New York, NY 10053, USA.

480. Department of Laboratory Medicine and Pathobiology, University of Toronto, Toronto, ON M5S 1A8, Canada.

481. BioSpecimen Sciences, Laboratory Medicine (Toronto), Medical Biophysics, PanCuRX, Toronto, ON M5S 1A8, Canada.

482. Department of Medical Biophysics, University of Toronto, Toronto, ON M5G 1L7, Canada.

483. CRUK Manchester Institute and Centre, Manchester M204GJ, UK.

484. Department of Radiation Oncology, University of Toronto, Toronto, ON M5S 1A8, Canada.

485. Manchester Cancer Research Centre, Cancer Division, FBMH, University of Manchester, Manchester M204GJ, UK.

486. Radiation Medicine Program, Princess Margaret Cancer Centre, Toronto, ON M5G 2M9, Canada.

487. Department of Surgical Oncology, Princess Margaret Cancer Centre, Toronto, ON M5G 2M9, Canada.

488. Genome Informatics Program, Ontario Institute for Cancer Research, Toronto, ON M5G 2C4, Canada.

489. STTARR Innovation Facility, Princess Margaret Cancer Centre, Toronto, ON M5G 1L7, Canada.

490. Department of Pathology, Toronto General Hospital, Toronto, ON M5G 2C4, Canada.

491. Hefei University of Technology, Anhui 230009, China.

492. State key Laboratory of Cancer Biology, and Xijing Hospital of Digestive Diseases, Fourth Military Medical University, Shaanxi 710032, China.

493. Fourth Military Medical University, Shaanxi 710032, China.

494. Peking University Cancer Hospital & Institute, Beijing 100142, China.

495. Department of Surgery, Ruijin Hospital, Shanghai Jiaotong University School of Medicine, Shanghai, 200025, China.

496. Leeds Institute of Medical Research @ St James's, University of Leeds, St James's University Hospital, Leeds LS9 7TF, UK.

497. Canadian Center for Computional Genomics, McGill University, Montreal, QC H3A 0G1, Canada.

498. Department of Human Genetics, McGill University, Montreal, QC H3A 1B1, Canada.

499. International Agency for Research on Cancer, Lyon 69008, France.

500. McGill University and Genome Quebec Innovation Centre, Montreal, QC H3A 0G1, Canada.

501. Centre National de Génotypage, CEA - Institute de Génomique, Evry 91000, France.

502. Leeds Institute of Medical Research @ St James's, University of Leeds, St James's University Hospital, Leeds LS9 7TF, UK.

503. Institute of Mathematics and Computer Science, University of Latvia, Riga LV1459, Latvia.

504. Department of Oncology, Gil Medical Center, Gachon University, Incheon, South Korea.

505. Department of Molecular Oncology, BC Cancer Agency, Vancouver, BC V5Z 1L3, Canada.

506. Los Alamos National Laboratory, Los Alamos, NM 87545, USA.

507. Department of Genetics, Institute for Cancer Research, Oslo University Hospital, The Norwegian Radium Hospital, Oslo O310, Norway.

508. Lund University, Lund 223 62, Sweden.

509. Translational Research Lab, Centre Léon Bérard, Lyon 69373, France.

510. Radboud University, Department of Molecular Biology, Faculty of Science, Nijmegen Centre for Molecular Life Sciences, Nijmegen 6500 HB, The Netherlands.

511. Department of Pathology, Brigham and Women's Hospital, Harvard Medical School, Boston, MA 02115, USA.

512. Department Experimental Therapy, The Netherlands Cancer Institute, Amsterdam 1066 CX, The Netherlands.

513. Cancer Research UK Cambridge Institute, University of Cambridge, Li Ka Shing Centre, Cambridge CB2 0RE, UK.

514. Department of Oncology, University of Cambridge, Cambridge CB2 1TN, UK.

515. Breast Cancer Translational Research Laboratory JC Heuson, Institut Jules Bordet, Brussels 1000, Belgium.

516. Translational Cancer Research Unit, Center for Oncological Research, Faculty of Medicine and Health Sciences, University of Antwerp, Antwerp 2000, Belgium.

517. Department of Gynecology & Obstetrics, Department of Clinical Sciences, Skåne University Hospital, Lund University, Lund SE-221 85, Sweden.

518. Icelandic Cancer Registry, Icelandic Cancer Society, Reykjavik 125, Iceland.

519. Translational Cancer Research Unit, GZA Hospitals St.-Augustinus, Antwerp 2000, Belgium.

520. Department of Pathology, Memorial Sloan Kettering Cancer Center, New York, NY 10065, USA.

521. Department of Medical Oncology, Josephine Nefkens Institute and Cancer Genomics Centre, Erasmus Medical Center, Rotterdam 3015CN, The Netherlands.

522. National Genotyping Center, Institute of Biomedical Sciences, Academia Sinica, Taipei 115, Taiwan.

523. Department of Pathology, Oslo University Hospital Ulleval, Oslo 0450, Norway.

524. Faculty of Medicine and Institute of Clinical Medicine, University of Oslo, Oslo NO-0316, Norway.

525. Department of Pathology, Skåne University Hospital, Lund University, Lund SE-221 85, Sweden.

526. Department of Pathology, Academic Medical Center, Amsterdam 1105 AZ, The Netherlands.

527. Department of Pathology, College of Medicine, Hanyang University, Seoul 133-791, South Korea.

528. Department of Pathology, Asan Medical Center, College of Medicine, Ulsan University, Songpa-gu, Seoul 05505, South Korea.

529. Netherlands Cancer Institute, Lund University, Lund 223 62, Sweden.

530. Department of Surgery, Memorial Sloan-Kettering Cancer Center, New York, NY 10065, USA.

531. Department of Surgery, Brigham and Women's Hospital/Dana Farber Cancer Insititute, Boston, MA 02115, USA.

532. Morgan Welch Inflammatory Breast Cancer Research Program and Clinic, The University of Texas MD Anderson Cancer Center, Houston, TX 77030, USA.

533. The University of Queensland Centre for Clinical Research, The Royal Brisbane & Women's Hospital, Herston, QLD 4029, Australia.

534. Department of Pathology, Jules Bordet Institute, Brussels 1000, Belgium.

535. Institute for Bioengineering and Biopharmaceutical Research (IBBR), Hanyang University, Seoul, South Korea.

536. University of Oslo, Oslo 0316, Norway.

537. Institut Bergonié, Bordeaux 33076, France.

538. Department of Pathology, Erasmus Medical Center Rotterdam, Rotterdam 3015 GD, The Netherlands.

539. Department of Research Oncology, Guy's Hospital, King's Health Partners AHSC, King's College London School of Medicine, London SE1 9RT, UK.

540. University Hospital of Minjoz, INSERM UMR 1098, Besançon 25000, France.

541. Cambridge Breast Unit, Addenbrooke's Hospital, Cambridge University Hospital NHS Foundation Trust and NIHR Cambridge Biomedical Research Centre, Cambridge CB2 2QQ, UK.

542. East of Scotland Breast Service, Ninewells Hospital, Aberdeen AB25 2XF, UK.

543. Oncologie Sénologie, ICM Institut Régional du Cancer, Montpellier 34298, France.

544. Los Almos National Laboratory, Los Alamos, NM 87545, USA.

545. Department of Radiation Oncology, Radboud University Medical Centre, Nijmegen 6525 GA, The Netherlands.

546. University of Iceland, Reykjavik 101, Iceland.

547. Dundee Cancer Centre, Ninewells Hospital, Dundee DD2 1SY, UK.

548. Institut Curie, INSERM Unit 830, Paris 75248, France.

549. Department of Laboratory Medicine, Radboud University Nijmegen Medical Centre, Nijmegen GA 6525, The Netherlands.

550. Department of General Surgery, Singapore General Hospital, Outram Rd, Singapore 169608, Singapore.

551. Universite Lyon, INCa-Synergie, Centre Léon Bérard, Lyon 69008, France.

552. Giovanni Paolo II / I.R.C.C.S. Cancer Institute, Bari BA 70124, Italy.

553. Department of Biopathology, Centre Léon Bérard, Lyon 69008, France.

554. Université Claude Bernard Lyon 1, Villeurbanne 69100, France.

555. NCCS-VARI Translational Research Laboratory, National Cancer Centre Singapore, Singapore 169610, Singapore.

556. Division of Molecular Carcinogenesis, The Netherlands Cancer Institute, Amsterdam 1066 CX, The Netherlands.

557. Division of Molecular Carcinogenesis, The Netherlands Cancer Institute, Amsterdam, The Netherlands.

558. Institute of Human Genetics, Christian-Albrechts-University, Kiel 24118, Germany.

559. Institute of Human Genetics, Ulm University and Ulm University Medical Center of Ulm, Ulm 89081, Germany.

560. Institute of Human Genetics, University of Ulm and University Hospital of Ulm, Ulm 89081, Germany.

561. Hematopathology Section, Institute of Pathology, Christian-Albrechts-University, Kiel 24118, Germany.

562. Department of Human Genetics, Hannover Medical School, Hannover 30625, Germany.

563. Department of Pediatric Oncology, Hematology and Clinical Immunology, Heinrich-Heine-University, Düsseldorf 40225, Germany.

564. Department of Internal Medicine/Hematology, Friedrich-Ebert-Hospital, Neumünster 24534, Germany.

565. University Hospital Muenster - Pediatric Hematology and Oncology, Muenster 24534, Germany.

566. Department of Pediatrics, University Hospital Schleswig-Holstein, Kiel 24105, Germany.

567. Department of Medicine II, University of Würzburg, Würzburg, Germany.

568. Senckenberg Institute of Pathology, University of Frankfurt Medical School, Frankfurt 60596, Germany.

569. Institute of Pathology, Charité – University Medicine Berlin, Berlin 10117, Germany.

570. Department for Internal Medicine II, University Hospital Schleswig-Holstein, Kiel 24105, Germany.

571. Institute for Medical Informatics Statistics and Epidemiology, University of Leipzig, Leipzig 04109, Germany.

572. Department of Hematology and Oncology, Georg-Augusts-University of Göttingen, Göttingen 37073, Germany.

573. Institute of Cell Biology (Cancer Research), University of Duisburg-Essen, Essen D-45147, Germany.

574. MVZ Department of Oncology, PraxisClinic am Johannisplatz, Leipzig 04109, Germany.

575. Institute of Pathology, Ulm University and University Hospital of Ulm, Ulm 89081, Germany.

576. Department of Pathology, Robert-Bosch-Hospital, Stuttgart, Germany, Stuttgart 70376, Germany.

577. University Hospital Giessen, Pediatric Hematology and Oncology, Giessen 35392, Germany.

578. Institute of Clinical Molecular Biology, Christian-Albrechts-University, Kiel 24118, Germany.

579. Institute of Pathology, University of Wuerzburg, Wuerzburg 97070, Germany.

580. Department of General Internal Medicine, University Kiel, Kiel 24118, Germany.

581. Clinic for Hematology and Oncology, St.-Antonius-Hospital, Eschweiler D-52249, Germany.

582. Department for Internal Medicine III, University of Ulm and University Hospital of Ulm, Ulm 89081, Germany.

583. Neuroblastoma Genomics, German Cancer Research Center (DKFZ), Heidelberg 69120, Germany.

584. University of Düsseldorf, Düsseldorf 40225, Germany.

585. Department of Vertebrate Genomics/Otto Warburg Laboratory Gene Regulation and Systems Biology of Cancer, Max Planck Institute for Molecular Genetics, Berlin 14195, Germany.

586. St. Jude Children's Research Hospital, Memphis, TN 38105-3678, USA.

587. Heidelberg University Hospital, Heidelberg 69120, Germany.

588. Genomics and Proteomics Core Facility High Throughput Sequencing Unit, German Cancer Research Center (DKFZ), Heidelberg 69120, Germany.

589. Epigenomics and Cancer Risk Factors, German Cancer Research Center (DKFZ), Heidelberg 69120, Germany.

590. University Medical Center Hamburg-Eppendorf, Hamburg 20251, Germany.

591. Institute of Pathology, University Medical Center Hamburg-Eppendorf, Hamburg 20251, Germany.

592. Martini-Clinic, Prostate Cancer Center, University Medical Center Hamburg-Eppendorf, Hamburg 20095, Germany.

593. Division of Cancer Genome Research, German Cancer Research Center (DKFZ), Heidelberg 69120, Germany.

594. National Institute of Biomedical Genomics, Kalyani 741235, West Bengal, India.

595. Advanced Centre for Treatment Research & Education in Cancer, Tata Memorial Centre, Navi Mumbai, Maharashtra 410210, India.

596. Department of Pathology, General Hospital of Treviso, Department of Medicine, University of Padua, Italy, Treviso 31100, Italy.

597. Department of Medicine (DIMED), Surgical Pathology Unit, University of Padua, Padua 35121, Italy.

598. Division of Pathology and Clinical Laboratories, Department of Hepatobiliary and Pancreatic Oncology, Hepatobiliary and Pancreatic Surgery Division, National Cancer Center Hospital, Chuo-ku, Tokyo, 104-0045, Japan.

599. Division of Cancer Genomics, Department of Bioinformatics, National Cancer Center, Tokyo 104-0045, Japan.

600. Department of Pathology, Keio University School of Medicine, Tokyo 160-8582, Japan.

601. Department of Hepatobiliary and Pancreatic Oncology, National Cancer Center Hospital (NCCH), Tokyo, 104-0045 Japan.

602. Department of Pathology, Graduate School of Medicine, The University of Tokyo, Bunkyo-ku, Tokyo 113-0033, Japan.

603. Preventive Medicine, Graduate School of Medicine, The University of Tokyo, Tokyo 113-0033, Japan.

604. Gastric Surgery Division, Division of Pathology and Clinical Laboratories, National Cancer Center Hospital, Tokyo 104-0045, Japan.

605. Department of Preventive Medicine, Graduate School of Medicine, The University of Tokyo, Tokyo 113-0033, Japan.

606. Division of Cancer Genomics, Department of Bioinformatics, National Cancer Center Research Institute, National Cancer Center, Tokyo 104-0045, Japan.

607. Department of Gastroenterology and Hepatology, Yokohama City University Graduate School of Medicine, Kanagawa 236-0004, Japan.

608. Laboratory of Molecular Medicine, Human Genome Center, The Institute of Medical Science, University of Tokyo, Shirokane-dai, Minato-ku, Tokyo 108-8639, Japan.

609. Department of Cancer Genome Informatics, Graduate School of Medicine, Osaka University, Osaka 565-0871, Japan.

610. Hiroshima University, Hiroshima 734-8553, Japan.

611. Tokyo Women's Medical University, Tokyo 162-8666, Japan.

612. Osaka International Cancer Center, Osaka 541-8567, Japan.

613. Wakayama Medical University, Wakayama 641-8509, Japan.

614. Hokkaido University, Sapporo 060-8648, Japan.

615. Division of Medical Oncology, National Cancer Centre, Singapore 169610, Singapore.

616. Cholangiocarcinoma Screening and Care Program and Liver Fluke and Cholangiocarcinoma Research Centre, Faculty of Medicine, Khon Kaen University, Khon Kaen, Thailand.

617. Lymphoma Genomic Translational Research Laboratory, National Cancer Centre, Singapore 169610, Singapore.

618. Center of Digestive Diseases and Liver Transplantation, Fundeni Clinical Institute, Bucharest 022328, Romania.

619. Division of Hepatobiliary and Pancreatic Surgery, Department of Surgery, School of Medicine, Keimyung University Dongsan Medical Center, Daegu 41931, South Korea.

620. Pathology, Hospital Clinic, Institut d'Investigacions Biomèdiques August Pi i Sunyer (IDIBAPS), Universiy of Barcelona, Barcelona 8034, Spain.

621. Anatomia Patológica, Hospital Clinic, Institut d'Investigacions Biomèdiques August Pi i Sunyer (IDIBAPS), Universiy of Barcelona, Barcelona 8036, Spain.

622. Spanish Ministry of Science and Innovation, Madrid 28046, Spain.

623. Hematology, Hospital Clinic, Institut d'Investigacions Biomèdiques August Pi i Sunyer (IDIBAPS), Universiy of Barcelona, Barcelona 8034, Spain.

624. Departamento de Bioquımicay Biologıa Molecular, Facultad de Medicina, Instituto Universitario de Oncologıa-IUOPA, Oviedo 33006, Spain.

625. Departamento de Bioquímica y Biología Molecular, Instituto Universitario de Oncología (IUOPA), Universidad de Oviedo, Oviedo 33006, Spain.

626. Royal National Orthopaedic Hospital - Bolsover, London W1W 5AQ, UK.

627. Department of Pathology, Oslo University Hospital, Oslo O310, Norway.

628. Department of Pathology, Oslo University Hospital, Norway and University of Oslo, Norway, Oslo O310, Norway.

629. Department of Pathology (Research), University College London Cancer Institute, London WC1E 6BT, UK.

630. Department for Clinical Science, University of Bergen, Bergen 5020, Norway.

631. Research Department of Pathology, University College London Cancer Institute, 72 Huntley Street, London, WC1E 6BT.

632. East Anglian Medical Genetics Service, Cambridge University Hospitals NHS Foundation Trust, Cambridge CB2 0QQ, UK.

633. Royal National Orthopaedic Hospital - Stanmore, Stanmore, Middlesex HA7 4LP, UK.

634. Division of Orthopaedic Surgery, Oslo University Hospital, Oslo 0379, Norway.

635. Radcliffe Department of Medicine, University of Oxford, Oxford OX3 9DU, UK.

636. University of Pavia, Pavia 27100, Italy.

637. Karolinska Institute, Stockholm SE-171 76, Sweden.

638. Wellcome Sanger Institute, Wellcome Genome Campus, Hinxton, Cambridge, CB10 1SD, UK.

639. University of Oxford, Oxford OX3 9DU, UK.

640. Salford Royal NHS Foundation Trust, Salford M6 8HD, UK.

641. Gloucester Royal Hospital, Gloucester, GL1 3NL, UK.

642. Royal Stoke University Hospital, Stoke-on-Trent ST4 6QG, UK.

643. St Thomas's Hospital, London SE1 7EH, UK.

644. Imperial College NHS Trust, Imperial College, London W2 INY, UK.

645. Department of Histopathology, Salford Royal NHS Foundation Trust, Salford M6 8HD, UK.

646. Faculty of Biology, Medicine and Health, University of Manchester, Salford M6 8HD, UK.

647. Edinburgh Royal Infirmary, Edinburgh EH16 4SA, UK.

648. Barking Havering and Redbridge University Hospitals NHS Trust, Romford, RM7 0AG, UK.

649. King's College London and Guy's and St Thomas' NHS Foundation Trust, London SE1 7EH, UK.

650. Cambridge Oesophagogastric Centre, Cambridge University Hospitals NHS Foundation Trust, Cambridge, CB2 0QQ.

651. Nottingham University Hospitals NHS Trust, Nottingham NG7 2UH, UK.

652. St Luke's Cancer Centre, Royal Surrey County Hospital NHS Foundation Trust, Guildford GU2 7XX, UK.

653. University of North Carolina at Chapel Hill, Chapel Hill, NC 27599, USA.

654. Norfolk and Norwich University Hospital NHS Trust, Norwich NR4 7UY, UK.

655. University Hospitals Coventry and Warwickshire NHS Trust, Coventry CV2 2DX, UK.

656. University Hospitals Birmingham NHS Foundation Trust, Birmingham B15 2GW, UK.

657. Centre for Cancer Research and Cell Biology, Queen's University, Belfast BT9 7AB, UK.

658. University Hospital Southampton NHS Foundation Trust, Southampton, SO16 6YD, UK.

659. Wythenshawe Hospital, Manchester M23 9LT, UK.

660. Royal Marsden NHS Foundation Trust, London and Sutton SW3 6JJ, UK.

661. Barts Cancer Institute, Barts and the London School of Medicine and Dentistry, Queen Mary University of London, London EC1M 6BQ, UK.

662. HCA Laboratories, London W1G 8AQ, UK.

663. University of Liverpool, Liverpool L69 3BX, UK.

664. Academic Urology Group, Department of Surgery, University of Cambridge, Cambridge CB2 0QQ, UK.

665. University of Oxford, Oxford, OX3 9DU, UK.

666. Department of Surgery and Cancer, Imperial College, London W2 INY, UK.

667. The Chinese University of Hong Kong, Shatin, Hong Kong, China.

668. Nuffield Department of Surgical Sciences, John Radcliffe Hospital, University of Oxford, Headington, Oxford OX3 9DU, UK.

669. Department of Histopathology, Cambridge University Hospitals NHS Foundation Trust, Cambridge CB2 0QQ, UK.

670. Department of Bioinformatics and Computational Biology / Department of Systems Biology, The University of Texas MD Anderson Cancer Center, Houston, TX 77030, USA.

671. Department of Pathology, The University of Texas MD Anderson Cancer Center, Houston, TX 77030, USA.

672. Canada's Michael Smith Genome Sciences Center, BC Cancer Agency, Vancouver, BC V5Z 4S6, Canada.

673. Center for Molecular Oncology, Memorial Sloan Kettering Cancer Center, New York, NY 10065, USA.

674. University Health Network, Toronto, ON M5G 2C4, Canada.

675. Department of Pathology and Laboratory Medicine, School of Medicine, University of North Carolina at Chapel Hill, Chapel Hill, NC 27599, USA.

676. Department of Population and Quantitative Health Sciences, Case Western Reserve University School of Medicine, Cleveland, OH 44016, USA.

677. Research Health Analytics and Informatics, University Hospitals Cleveland Medical Center, Cleveland, OH 44106, USA.

678. Arnie Charbonneau Cancer Institute, University of Calgary, Calgary, AB T2N 4N2, Canada.

679. Centre for Cancer Research and Cell Biology, Queens University, Belfast BT9 7AB, UK.

680. Sidney Kimmel Cancer Center, The Johns Hopkins Medical Institutions, Baltimore, MD 21230, USA.

681. Buck Institute for Research on Aging, Novato, CA 94945, USA.

682. Duke University Medical Center, Durham, NC 27710, USA.

683. Norris Comprehensive Cancer Center, University of Southern California, Los Angeles, CA 90033, USA.

684. The Preston Robert Tisch Brain Tumor Center, Duke University Medical Center, Durham, NC 27710, USA.

685. University of Southern California, USC/Norris Comprehensive Cancer Center, Los Angeles, CA 90033, USA.

686. Departments of Dermatology and Pathology, Yale University, New Haven, CT 06510, USA.

687. Fox Chase Cancer Center, Philadelphia, PA 19111, USA.

688. Johns Hopkins University, Baltimore, MD 21287, USA.

689. University of Michigan Comprehensive Cancer Center, Ann Arbor, MI 48109, USA.

690. University of Alabama at Birmingham, Birmingham, AL 35294, USA.

691. Division of Anatomic Pathology, Mayo Clinic, Rochester, MN 55905, USA.

692. Division of Experimental Pathology, Mayo Clinic, Rochester, MN 55905 USA.

693. The Sidney Kimmel Comprehensive Cancer Center at Johns Hopkins University, Baltimore, MD 21287, USA.

694. International Genomics Consortium, Phoenix, AZ 85004, USA.

695. Departments of Pediatrics and Genetics, University of North Carolina at Chapel Hill, Chapel Hill, NC 27599, USA.

696. Department of Pathology, UPMC Shadyside, Pittsburgh, PA 15232, USA.

697. Center for Cancer Genomics, National Cancer Institute, National Institutes of Health, Bethesda, MD 20892, USA.

698. Istituto Neurologico Besta, Department of Neuro-Oncology, Milano 20133, Italy.

699. University of Queensland Thoracic Research Centre, The Prince Charles Hospital, Brisbane, QLD 4032, Australia.

700. Department of Neurosurgery, University of Florida, Gainesville, FL 32610, USA.

701. Center for Biomedical Informatics, Harvard Medical School, Boston, MA 02115, USA.

702. Department of Cancer Biology, The University of Texas MD Anderson Cancer Center, Houston, TX 77030, USA.

703. Department of Surgical Oncology, The University of Texas MD Anderson Cancer Center, Houston, TX 77030, USA.

704. Division of Gastroenterology and Hepatology, Mayo Clinic, Rochester, MN 55905, USA.

705. University of Miami, Sylvester Comprehensive Cancer Center, Miami, FL 33136, USA.

706. Department of Internal Medicine, Division of Medical Oncology, University of North Carolina at Chapel Hill, Chapel Hill, NC 27599, USA.

707. Department of Internal Medicine, Division of Medical Oncology, Lineberger Comprehensive Cancer Center, University of North Carolina at Chapel Hill, Chapel Hill, NC 27599, USA.

708. University of Southern California, Norris Comprehensive Cancer Center, Los Angeles, CA 90033, USA.

709. The Sol Goldman Pancreatic Cancer Research Center, Department of Pathology, Johns Hopkins Hospital, Baltimore, MD 21287, USA.

710. Centre for Translational and Applied Genomics, British Columbia Cancer Agency, Vancouver, BC V5Z 1L3, Canada.

711. Department of Pathology & Immunology, Baylor College of Medicine, Houston, TX 770230, USA.

712. Michael E. DeBakey Veterans Affairs Medical Center, Houston, TX 770230, USA.

713. Carolina Center for Genome Sciences, University of North Carolina at Chapel Hill, Chapel Hill, NC 27599, USA.

714. Canada's Michael Smith Genome Sciences Centre, BC Cancer, Vancouver, BC V5Z 4S6, Canada.

715. Indivumed GmbH, Hamburg 20251, Germany.

716. Division of Hepatobiliary and Pancreatic Surgery, Department of Surgery, School of Medicine, Keimyung University Dong-san Medical Center, Daegu 41931, South Korea.

717. Women's Cancer Program at the Samuel Oschin Comprehensive Cancer Institute, Cedars-Sinai Medical Center, Los Angeles, CA 90048, USA.

718. Canada's Michael Smith Genome Sciences Centre, BC Cancer Agency, Vancouver, BC V5Z 4S6, Canada.

719. Department of Surgery, The George Washington University, School of Medicine and Health Science, Washington, DC 20052, USA.

720. Endocrine Oncology Branch, Center for Cancer Research, National Cancer Institute, National Institutes of Health, Bethesda, MD 20892, USA.

721. ILSbio, LLC Biobank, Chestertown, MD 21620, USA.

722. Gynecologic Oncology, NYU Laura and Isaac Perlmutter Cancer Center, New York University, New York, NY 10016, USA.

723. Division of Oncology, Stem Cell Biology Section, Washington University School of Medicine, St. Louis, MO 63110, USA.

724. Urologic Oncology Branch, Center for Cancer Research, National Cancer Institute, National Institutes of Health, Bethesda, MD 20892, USA.

725. Department of Systems Biology, University of Texas MD Anderson Cancer Center, Houston, TX 77030, USA.

726. Institute for Systems Biology, Seattle, WA 98109, USA.

727. Institute for Genomic Medicine, Nationwide Children's Hospital, Columbus, OH 43215, USA.

728. Department of Surgery, Duke University, Durham, NC 27710, USA.

729. Department of Obstetrics, Gynecology and Reproductive Services, University of California San Francisco, San Francisco, CA 94143, USA.

730. Departments of Neurology and Neurosurgery, Henry Ford Hospital, Detroit, MI 48202, USA.

731. Department of Pathology, Roswell Park Cancer Institute, Buffalo, NY 14263, USA.

732. Department of Obstetrics and Gynecology, Division of Gynecologic Oncology, Washington University School of Medicine, St. Louis, MO 3110, USA.

733. Penrose St. Francis Health Services, Colorado Springs, CO 80907, USA.

734. The University of Chicago, Chicago, IL 60637, USA.

735. Department of Neurology, Mayo Clinic, Rochester, MN 55905, USA.

736. Department of Genetics and Lineberger Comprehensive Cancer Center, University of North Carolina at Chapel Hill, Chapel Hill, NC 27599, USA.

737. NYU Langone Medical Center, New York, NY 10016, USA.

738. Department of Hematology and Medical Oncology, Cleveland Clinic, Cleveland, OH 44195, USA.

739. Department of Genetics, Department of Pathology and Laboratory Medicine, School of Medicine, University of North Carolina at Chapel Hill, Chapel Hill, NC 27599, USA.

740. Helen F. Graham Cancer Center at Christiana Care Health Systems, Newark, DE 19713, USA.

741. Cureline, Inc, South San Francisco, CA 94080, USA.

742. Department of Obstetrics and Gynecology, Medical College of Wisconsin, Milwaukee, WI 53226, USA.

743. Emory University, Atlanta, GA 30322, USA.

744. Vanderbilt University, Vanderbilt Ingram Cancer Center, Nashville, TN 37232, USA.

745. Ohio State University College of Medicine and Arthur G. James Comprehensive Cancer Center, Columbus, OH 43210, USA.

746. Research Computing Center, University of North Carolina at Chapel Hill, Chapel Hill, NC 27599, USA.

747. Analytical Biological Services, Inc, Wilmington, DE 19801, USA.

748. Department of Dermatology, University Hospital Essen, Westdeutsches Tumorzentrum & German Cancer Consortium, Essen 45122, Germany.

749. University of Pittsburgh, Pittsburgh, PA 15213, USA.

750. Murtha Cancer Center, Walter Reed National Military Medical Center, Bethesda, MD 20889, USA.

751. Brigham and Women's Hospital, Harvard Medical School, Boston, MA 02115, USA.

752. Department of Surgery, Memorial Sloan Kettering Cancer Center, New York, NY 10065, USA.

753. Department of Gynecologic Oncology & Reproductive Medicine, and Center for RNA Interference and Non-Coding RNA, the University of Texas MD Anderson Cancer Center, Houston, TX 77030, USA.

754. Department of Urology, Mayo Clinic, Rochester, MN 55905, USA.

755. Johns Hopkins Medical Institutions, Baltimore, MD 21205, USA.

756. Departments of Neurosurgery and Hematology and Medical Oncology, Winship Cancer Institute and School of Medicine, Emory University, Atlanta, GA 30322, USA.

757. Georgia Regents University Cancer Center, Augusta, GA 30912, USA.

758. Thoracic Oncology Laboratory, Mayo Clinic, Rochester, MN 55905, USA.

759. Institute for Genomic Medicine, Nationwide Children's Hospital, Columbus, OH 43205, USA.

760. Department of Obstetrics & Gynecology, Division of Gynecologic Oncology, Mayo Clinic, Rochester, MN 55905, USA.

761. International Institute for Molecular Oncology, Poznań 60-203, Poland.

762. Poznan University of Medical Sciences, Poznań 61-701, Poland.
