# Supplementary material for: Inferring structural variant cancer cell fraction
Source: Nat Commun. 2020 Feb 5;11:730. doi: 10.1038/s41467-020-14351-8 (PMC7002525; doi:10.1038/s41467-020-14351-8)
Supplement: Supplementary file 3 — Reporting Summary [file 41467_2020_14351_MOESM3_ESM.pdf]

nature research

Corresponding author(s): Geoff Macintyre, Marek Cmero

Last updated by author(s): Dec 9, 2019

# Reporting Summary

Nature Research wishes to improve the reproducibility of the work that we publish. This form provides structure for consistency and transparency in reporting. For further information on Nature Research policies, seeAuthors & Referees and theEditorial Policy Checklist .

## Statistics

For all statistical analyses, confirm that the following items are present in the figure legend, table legend, main text, or Methods section.

| n/a | Confirmed | |
|---|---|---|
| ☐ | ☒ | The exact sample size (*n*) for each experimental group/condition, given as a discrete number and unit of measurement |
| ☒ | ☐ | A statement on whether measurements were taken from distinct samples or whether the same sample was measured repeatedly |
| ☐ | ☒ | The statistical test(s) used AND whether they are one- or two-sided<br>*Only common tests should be described solely by name; describe more complex techniques in the Methods section.* |
| ☐ | ☒ | A description of all covariates tested |
| ☐ | ☒ | A description of any assumptions or corrections, such as tests of normality and adjustment for multiple comparisons |
| ☐ | ☒ | A full description of the statistical parameters including central tendency (e.g. means) or other basic estimates (e.g. regression coefficient) AND variation (e.g. standard deviation) or associated estimates of uncertainty (e.g. confidence intervals) |
| ☐ | ☒ | For null hypothesis testing, the test statistic (e.g. *F*, *t*, *r*) with confidence intervals, effect sizes, degrees of freedom and *P* value noted<br>*Give P values as exact values whenever suitable.* |
| ☐ | ☒ | For Bayesian analysis, information on the choice of priors and Markov chain Monte Carlo settings |
| ☒ | ☐ | For hierarchical and complex designs, identification of the appropriate level for tests and full reporting of outcomes |
| ☐ | ☒ | Estimates of effect sizes (e.g. Cohen's *d*, Pearson's *r*), indicating how they were calculated |

*Our web collection on statistics for biologists contains articles on many of the points above.*

## Software and code

Policy information about availability of computer code

| | |
|---|---|
| Data collection | This manuscript involves analysis of existing data which is described in the following manuscripts:<br>Dentro, S. C., Leshchiner, I., Haase, K. & Tarabichi, M. Portraits of genetic intra-tumour heterogeneity and subclonal selection across cancer types. bioRxiv (2018).<br>Campbell, P. J. et al. Pan-cancer analysis of whole genomes. bioRxiv 162784 (2017). doi:10.1101/162784<br>Gerstung, M. et al. The evolutionary history of 2,658 cancers. bioRxiv 161562 (2017). doi:10.1101/161562<br>Hong, M. K. H. et al. Tracking the origins and drivers of subclonal metastatic expansion in prostate cancer. Nat. Commun. 6, 1–12 (2015). |
| Data analysis | The SVclone software, user documentation, and example data can be downloaded from https://github.com/mcmero/SVclone. Ccube clustering code can be found under https://github.com/keyuan/ccube. Code for generating all figures in the manuscript and the in silico mixture samples can be found under https://github.com/mcmero/SVclone_Rmarkdown. Code for simulating SVs can be found under https://github.com/mcmero/sv_simu_pipe.<br><br>The core computational pipelines used by the PCAWG Consortium for alignment, quality control and variant calling are available to the public at https://dockstore.org/search?search=pcawg under the GNU General Public License v3.0, which allows for reuse and distribution. |

For manuscripts utilizing custom algorithms or software that are central to the research but not yet described in published literature, software must be made available to editors/reviewers. We strongly encourage code deposition in a community repository (e.g. GitHub). See the Nature Research guidelines for submitting code & software for further information.

## Data

Policy information about availability of data

All manuscripts must include a data availability statement. This statement should provide the following information, where applicable:

- Accession codes, unique identifiers, or web links for publicly available datasets
- A list of figures that have associated raw data
- A description of any restrictions on data availability

in silico sample mixtures were generated from patient data derived from patient 001 from the Hong et al. study23. The data are available in the EGA Sequence Read Archive under accession EGAS00001000942 [https://www.ebi.ac.uk/ega/studies/EGAS00001000942].
Somatic and germline variant calls, mutational signatures, subclonal reconstructions, transcript abundance, splice calls and other core data generated by the ICGC/TCGA Pan-cancer Analysis of Whole Genomes Consortium is described here41 and available for download at https://dcc.icgc.org/releases/PCAWG. Additional information on accessing the data, including raw read files, can be found at https://docs.icgc.org/pcawg/data/. In accordance with the data access policies of the ICGC and TCGA projects, most molecular, clinical and specimen data are in an open tier which does not require access approval. To access potentially identification information, such as germline alleles and underlying sequencing data, researchers will need to apply to the TCGA Data Access Committee (DAC) via dbGaP (https://dbgap.ncbi.nlm.nih.gov/aa/wga.cgi?page=login) for access to the TCGA portion of the dataset, and to the ICGC Data Access Compliance Office (DACO; http://icgc.org/daco) for the ICGC portion. In addition, to access somatic single nucleotide variants derived from TCGA donors, researchers will also need to obtain dbGaP authorisation. Derived data sets described specifically in this manuscript can be found at these locations:
https://www.synapse.org/#!Synapse:syn7596712 (consensus SVs)
https://www.synapse.org/#!Synapse:syn7357330 (consensus SNVs and INDELs)
https://www.synapse.org/#!Synapse:syn8042880 (consensus copy-numbers)
All the other data supporting the findings of this study are available within the article and its supplementary information files and from the corresponding author upon reasonable request.

# Field-specific reporting

Please select the one below that is the best fit for your research. If you are not sure, read the appropriate sections before making your selection.

[x] Life sciences  [ ] Behavioural & social sciences  [ ] Ecological, evolutionary & environmental sciences

For a reference copy of the document with all sections, see nature.com/documents/nr-reporting-summary-flat.pdf

# Life sciences study design

All studies must disclose on these points even when the disclosure is negative.

| | |
|---|---|
| Sample size | Sample size was determined by the number of samples available in the original studies. Appropriate statistical tests and multiple correction was applied to ensure that all reported results were well powered given the sample numbers. |
| Data exclusions | Samples were excluded from analysis based on data quality. Details on exclusion criteria can be found in the methods. |
| Replication | As analyses were based on single primary tissue samples, replication was not possible. |
| Randomization | Randomisation was not applicable as all analyses were discovery based. |
| Blinding | Blinding was not applicable as no classification was performed. |

# Reporting for specific materials, systems and methods

We require information from authors about some types of materials, experimental systems and methods used in many studies. Here, indicate whether each material, system or method listed is relevant to your study. If you are not sure if a list item applies to your research, read the appropriate section before selecting a response.

## Materials & experimental systems

| n/a | Involved in the study | |
|---|---|---|
| [x] | [ ] | Antibodies |
| [x] | [ ] | Eukaryotic cell lines |
| [x] | [ ] | Palaeontology |
| [x] | [ ] | Animals and other organisms |
| [x] | [ ] | Human research participants |
| [x] | [ ] | Clinical data |

## Methods

| n/a | Involved in the study | |
|---|---|---|
| [x] | [ ] | ChIP-seq |
| [x] | [ ] | Flow cytometry |
| [x] | [ ] | MRI-based neuroimaging |
